# Global health burdens of plastics: a lifecycle assessment model from 2016 to 2040

**DOI:** 10.1016/j.lanplh.2025.101406

**Published:** 2026-01-26

**Authors:** Megan Deeney, Lorie Hamelin, Claire Vialle, Xiaoyu Yan, Rosemary Green, Joe Yates, Suneetha Kadiyala

**Affiliations:** a London School of Hygiene & Tropical Medicine, London, UK; b Toulouse Biotechnology Institute, Université de Toulouse, Centre National de la Recherche Scientifique, Institut National de la Recherche Agronomique, Institut National des Sciences Appliqués, Toulouse, France; c Université de Toulouse, Institut National Polytechnique de Toulouse, Institut National de la Recherche Agronomique, Laboratoire de Chimie Agro-Industrielle, Toulouse, France; d University of Exeter, Exeter, UK

## Abstract

**Background:**

Quantifying human health impacts throughout plastics lifecycles can inform global action against pollution that promotes sustainability across environmental, economic, and health concerns.

**Methods:**

We combined material flow analysis, using the Plastics-to-Ocean model, with lifecycle assessment (LCA) to quantitatively compare disability-adjusted life-years (DALYs) associated with plastics lifecycles, under different global consumption and waste management scenarios between 2016 and 2040. We estimated global health effects of greenhouse gases, particulate matter, and specific chemical emissions associated with plastics commonly found in municipal solid waste (approximately 64% of global plastics production), from their production, transportation, recycling, end-of-life fates, and those associated with illustrative alternative single-use materials and glass reuse systems. Direct health effects of exposure to chemicals during product use and microplastics and nanoplastics pollution were not included due to the absence of available inventory data. We conducted the LCA using Ecoinvent version 3.8 and 3.10 (cutoff) data and ReCiPe 2016 impact assessment method, updated with Intergovernmental Panel on Climate Change 2021 global warming characterisation factors.

**Findings:**

We estimated a cumulative 83 million DALYs associated with business as usual (BAU) projections of the global plastics system (2016–40), mainly due to the health burdens of global warming, air pollution, and chemical toxic effect-related disease and premature mortality. Compared with BAU, reducing total global primary plastics production, combined with improving waste collection and disposal, increasing recycling, and replacing specific plastics with alternative materials and reuse systems reduced annual DALYs by 43% (46–23% in material substitution ratio sensitivity analyses) in 2040, but still indicated rising global health burdens over time. Reducing primary plastics production, without material substitution, was the most effective single lever for reducing emissions and alleviating associated health burdens.

**Interpretation:**

Adverse health effects are associated with emissions throughout plastics lifecycles, particularly from production, though the non-disclosure of plastics chemical composition is severely limiting LCA capacity to inform effective policy. Deep reductions in primary production are needed in leading plastics transition roadmaps, with assessments that account for plastics’ functions across sectors, including exposure and health impacts from plastics’ use, from chemicals, microplastics, and nanoplastics, facilitated by mandatory transparency and reporting. Globally coordinated policy that addresses these upstream impacts through a full lifecycle approach is crucial to protecting human health.

**Funding:**

Innovative Methods and Metrics for Agriculture and Nutrition Actions programme, UK Foreign Commonwealth and Development Office, Bill & Melinda Gates Foundation, French National Research Agency and Region Occitanie.

## Introduction

Global mismanagement of plastics is breaching planetary boundaries^1^^,^^2^ and the human right to a safe, clean, healthy, and sustainable environment.^3^ Plastic pollution and its lifecycle emissions are damaging the lives and wellbeing of populations worldwide, but the magnitude of manifold health impacts have not yet been fully quantified.^4^^,^^5^ Demonstrating the scale, and making apparent less visible sources, of plastics’ health effects could propel more ambitious action to end pollution, and shape safer, more sustainable solutions across environmental, economic, and human health concerns.^6^^,^^7^

**Research in context**

**Evidence before this study**

Existing scientific evidence has indicated adverse human health effects at every stage of the plastics lifecycle, associated with greenhouse gases and different forms of plastics pollution including hazardous chemicals, air pollutants, and environmental leakage of plastics materials and particles. Nevertheless, aggregate health effects across plastics lifecycles have yet to be fully quantified. We previously systematically reviewed the extent, range, and nature of evidence on the effects of plastics used throughout food systems (evidence published between 2000 and 2018), which identified opportunities to assess health effects through lifecycle assessment (LCA). We conducted a meta-analysis of LCA studies (published up to 2021) that indicated recycling and reusing plastics could reduce greenhouse gases and air pollution relative to linear economies of plastics, but identified major data limitations. We searched PubMed for literature published from Jan 1, 2021, to Oct 28, 2025, with no geographical or publication date restrictions, in English, using key search terms for plastics and health: ("plastic"∗ OR "polymer") AND ("human health" OR “public health” OR "global health" OR "sustainab"∗ OR "planetary"). Research on the presence and potential harms associated with microplastics and nanoplastics and plastics-related chemicals in the environment, foods, and human bodies has proliferated in recent years, alongside evidence of plastics’ adverse effects across planetary boundaries. Global scale modelling of plastics lifecycles, based on combinations of LCA, material flow analysis, and the Planetary Boundaries Framework, have assessed a range of environmental effects. LCAs of the Organisation for Economic Co-operation and Development projections estimated human toxic effects and the health impacts of ozone formation associated with the global plastics system for the years 2019 and 2060. However, none have quantitatively assessed global health outcomes using all relevant LCA indicators.

**Added value of this study**

Building on an existing material flow analysis, the Plastics-to-Ocean model, our study used LCA to provide the first global-scale quantitative assessment of disability-adjusted life-years associated with greenhouse gases, air pollutants, and specific chemicals emitted across the lifecycle of the most common, predominantly single-use plastics under six different global scenarios between 2016 and 2040. From raw material extraction and primary polymer production, to post-consumer waste collection, recycling technologies, industrial waste management, dumpsites, open burning, and macroplastics environmental pollution, the product lifecycles included in our analyses represent approximately 64% of global plastics production and most land-based plastics leakage to the ocean.

We showed that adverse health effects are associated with these types of emissions throughout the plastics lifecycle, most crucially from plastics production, including the oil and gas extraction for petrochemical feedstocks, which contributed to the health effects of global warming, air pollution-induced respiratory disease, and toxic effects from waste chemicals. However, the direct health effects of exposure to chemicals during product use and microplastics and nanoplastics pollution across the plastics lifecycle were not accounted for because of the absence of available inventory data. Similarly, it is unclear to what extent chemicals contained in plastics are captured at other stages of the plastics lifecycle because of a crucial absence of transparency in available data inventories. This represents a considerable gap across LCA studies, which could result in underestimating plastics’ health impacts by at least an order of magnitude. Yet, even without these major considerations, our analyses indicated growing human health burdens associated with the current and alternative trajectories of the global plastics system, suggesting far more substantial changes will be needed at scale to create safe and sustainable systems. These findings reinforce calls for strong policy action to mitigate plastics pollution and its lifecycle emissions, not just plastic waste and leakage.

Our model contributes a flexible framework that can be readily developed with new data and methods to increase the scope and precision of health estimates associated with plastics and their alternatives and substitute materials, technologies, systems, and services, in the rapidly changing policy landscape. To build on this model and to support urgent and contextualised policy decisions, we highlight priorities including mandatory access to transparent, geographically representative data on the chemical composition of plastics and their existing or potential alternatives, validated measures of industrial process emissions, and international flows of feedstocks, materials, products, and waste; incorporating and developing LCA methods to advance health impact assessments of the use stage of products and plastics chemicals, and the effects of microplastics and nanoplastics; and prospective, spatiotemporally explicit LCA integrated with human rights and social justice considerations to inform localised and international decision making within a globally co-ordinated strategy to end plastics pollution and protect human health.

**Implications of all the available evidence**

Scientific evidence of widespread global health burdens associated with plastics underscores the urgent need for ambitious global policy and plastics regulation, and a precautionary approach to mitigate emerging hazards associated with plastics and their alternatives, to protect human health. Resolution 5/14 to develop an internationally, legally binding instrument to end plastics pollution was adopted in March, 2022, by the UN Environment Assembly, which recognised the need to adopt a full lifecycle approach to reduce pollution and its harms. As of 2026, the future Global Plastics Treaty is under negotiation by more than 175 member states. Scientific evidence shows ending plastics pollution, according to the agreed goals of resolution 5/14, requires global, legally binding obligations to substantially reduce total plastics production, ensure chemical simplification and elimination of hazardous chemicals in plastics, and safe and sustainable design of plastics within an international framework of transparency, traceability, and reporting. An effective treaty would be guided by the principles of prevention, precaution, polluter pays, and non-regression, with the protection of human rights at its core, including the human right to health and the human right to a safe, clean, healthy, and sustainable environment.

Plastics lifecycles emit a range of gases and pollutants that contribute to the global burden of disease, including greenhouse gases that drive climate change, air pollutants linked to respiratory illnesses, and hazardous chemicals associated with cancers and other non-communicable diseases.^4^^,^^5^^,^^8^ These emissions occur across all stages of the plastics value chain: from oil and gas extraction, which provides the feedstocks for more than 90% of global plastics;^9^ to polymer production and product manufacturing, global transportation, recycling, and formal or informal waste management and mismanagement; to the gradual degradation of plastics in the environment.^4^ Between 2026 and 2050, the number of deaths caused by climate change could reach between 14·5 and 15·6 million in low-income and middle-income countries.^10^ Air pollution currently causes more than 6·5 million premature deaths each year globally, and chemical pollution adds a further 1·8 million deaths, both rising over time.^11^ Plastics’ contribution to these anthropogenic environmental pressures and the associated global burden of disease is underexplored. Meanwhile, efforts to address plastics pollution through waste management alone overlook or understate health risks related to climate change, air pollution, and chemical toxicity, perpetuating adverse consequences for health.^12^ Global plastics demand is set to double by 2050,^13^ exacerbating challenges for eliminating pollution, with mounting environmental and global health risks across plastics lifecycles.^5^

Systems-based modelling can help to capture the complexity of the plastics system and its effects, particularly where epidemiological data might be limited.^14^^,^^15^ Material flow analysis (MFA) quantifies plastics flows through sectors and regions as a spatially and temporally dynamic exposure, and lifecycle assessment (LCA) offers a systematic approach to assessing associated environmental and health effects across plastics lifecycles.^16^^,^^17^ Existing analyses have used elements of MFA and LCA to assess plastics leakage,^18^ or climate change associated with plastics,^19^^,^^20^ some with energy use and economic considerations,^21^^,^^22^ and one analysis based on broader planetary boundaries.^23^ An LCA of the Organisation for Economic Co-operation and Development ENV-Linkages model^13^^,^^24^ assessed the health effects of ozone formation as well as carcinogenic and non-carcinogenic toxicity associated with global plastics management in 2019 and projections for 2060.^13^ However, analyses were not designed to compare alternative global scenarios for plastics and did not include health risks from particulate matter formation and climate change.^13^ As yet, no model has accounted for the full range of lifecycle human health effects associated with different scenarios of the global plastics system.

Using a hybrid MFA and LCA approach, our study quantitatively estimates and compares the human health effects in disability-adjusted life-years (DALYs) associated with greenhouse gases, air pollutants, and chemical emissions across the lifecycle of the most common, predominantly single-use plastics under different global scenarios between 2016 and 2040.

## Methods

### Overview of existing material flow analysis: the Plastics-to-Ocean model

Our study built on an existing MFA, the Plastics-to-Ocean (P_2_O) model^18^^,^^25^ of global flows of plastics commonly found in municipal solid waste, from production to end-of-life. Within six global scenarios, P_2_O tested the capacity of different plastics pollution mitigation levers (eg, reducing production, increasing recycling, and substituting plastics) to reduce plastics leakage to the environment between 2016 and 2040, accounting for projected increases in plastics production and geographical differences in waste generation and management (panel 1).^18^^,^^25^

Panel 1 **Key definitions and summary of Plastics-to-Ocean (P2O) system scenarios, geographical archetypes, and plastics categories**

**Plastics terms**

• Plastics pollution: plastic objects, materials, particles, and chemicals contained in plastics, released to the environment at any stage and throughout the entire plastics lifecycle
• Lifecycle emissions: all substances emitted from any stage and throughout the entire plastics lifecycle including water vapour, gases, chemical elements, and compounds, including those emitted to any environmental compartment including air, land, groundwater, rivers, lakes, and oceans. In the context of this analysis, the term emissions is not used to refer to plastic objects, items, materials, or particles; it does include all greenhouse gases, particulate matter, chemical elements, and compounds
• Plastics chemicals: all chemicals identified in plastics or associated with processes in their lifecycles, including monomers, oligomers, processing aids, catalysts, additives, and non-intentionally added substances including degradation products, reaction byproducts, and contaminants
• Plastics material losses: plastic materials generated or released from lifecycle processes that are not incorporated in the subsequent stage of the plastics value chain, which might be collected, managed, or leaked to the environment
• Plastics leakage: the intentional or unintentional release of plastics to the natural environment at any stage of the lifecycle
• Plastics littering: the intentional or unintentional release of plastic objects, items, or materials to the natural environment by individuals during the consumer use stage
• Plastics litter: plastic objects, items, and materials present without purpose in the natural environment
• Plastics waste: plastic items disposed of, discarded, or lost after their intended use stage has ended
• Plastics alternatives: plastics that are not made from fossil-fuel based polymers—namely, bio-based plastics
• Plastics substitutes: non-plastic materials that might be used to replace synthetic fossil fuel-based polymers and bio-based plastics. Examples include glass, leather, wood, silk, paper, cotton, wool, stone, ceramic, and aluminium

**P_2_O model plastics system scenarios**

1. Business as usual: no change to current plastics-related policy, economics, infrastructure, materials, cultural norms, or consumer behaviours
2. Current commitments: assumes all existing major commitments (2016–19) made by the public and private sectors are implemented and enforced, including product bans and recycling targets
3. Collect and dispose: assumes global expansion of waste collection and increases in the global capacity of engineered and managed landfills and incineration facilities
4. Recycling: assumes expansion and investment into collection, sorting, mechanical recycling, and plastic-to-plastic chemical conversion infrastructure
5. Reduce and substitute: assumes reduction of plastics use through elimination, introduction of reuse and new delivery models, and plastics substitutes including paper, coated paper, and compostables
6. System change scenario: assumes all system interventions are applied concurrently

**P_2_O model geographical archetypes**

Geographical archetypes were designed on the basis of: (1) World Bank country income classification; (2) population density to account for geographical differences in plastics consumption, waste generation, and post-consumption waste management pathways; (3) population (%) residing near a body of water to account for differences in the quantity of mismanaged plastics waste leaking into aquatic sources. These factors established flows of plastics within archetypes, under different scenarios, and over time.

1. High-income rural
2. High-income urban
3. Upper middle-income rural
4. Upper middle-income urban
5. Lower middle-income rural
6. Lower middle-income urban
7. Low-income rural
8. Low-income urban

**P_2_O model plastics categories**

Based on municipal solid waste data which included: plastic packaging, single-use products, nappies and sanitary waste, cigarette butts, durable consumer products, household products, and business-to-business packaging. According to P_2_O, this represented approximately 64% of global plastics production and the majority of land-based plastics leakage to the ocean at the time of publication (2020). These plastics categories were assessed in isolation from all other materials found in municipal solid waste and did not include plastics under the categories of medical waste, hazardous waste, electronics, textiles and furnishings, agricultural waste, transportation and construction waste, and other industrial waste. Thermoset plastics and biodegradable plastics were excluded from P_2_O plastics categories within the main system.

1. Rigid monomaterials
2. Flexible monomaterials
3. Multilayer and multimaterials

Adapted from Lau and colleagues.^18^

P_2_O provides annual (2016–40) mass flows for the six system scenarios and distinguishes three broad categories of plastics at every lifecycle stage of the MFA system, by geographical archetype.^18^^,^^25^ These are described in panel 1. Our study paired these data with LCA to estimate the human health effects associated with each global system scenario. We conducted our analyses according to two sub-objectives: to generate static modular unitary building blocks to represent the DALYs (and related midpoint effects, such as global warming or particulate matter formation) associated with one million metric tonnes (Mt) of each plastics category for each lifecycle stage in the P_2_O model; and to combine these building blocks to represent each of the six global system scenarios between 2016 and 2040, comparing their relative effects on human health over time.

Ethical approval was not required for this research. All data used were secondary data related to industrial and environmental emissions and related open-access characterisation factors for lifecycle impact assessment.

### LCA

**Figure 1:**
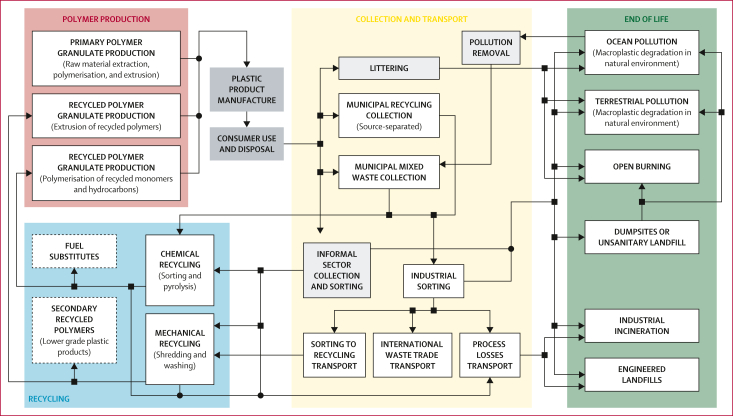
LCA system boundaries based on the P_2_O model Diagram adapted from the system map in P_2_O.^18^^,^^24^ Arrows describe P_2_O material flows, round joiners represent converging flows, squares represent diverging flows, and where there is no joiner the flows do not combine and only overlap for the purposes of visualising the map. Each box represents a modelled lifecycle stage. Boxes with solid outlines represent induced processes, and boxes with dotted outlines represent avoided processes. Plastic product manufacture and consumer use (dark grey boxes) were not included in the P_2_O material flow analyses and are outside of the LCA system boundary. Littering, informal sector collection and sorting, and environmental remediation of plastic litter (ie, organised beach clean-ups) were modelled as zero effect processes (light grey boxes), assuming human energy as the input with no emissions. Subsequent effects of these plastics are accounted for by their transferral to other processes. Avoided burdens of fuel production (fuel substitutes) and virgin plastic production (secondary recycled polymers) as a result of chemical and open-loop mechanical recycling were included. LCA=lifecycle assessment. P_2_O=Plastics-to-Ocean.

Our LCA was conducted in accordance with the International Organization for Standardization 14040 and 14044 standards.^26^^,^^27^ The goal was to estimate DALYs associated with 1 Mt of each of the three plastics categories included in P_2_O, for every lifecycle stage and for each geographical archetype in the global model (panel 1). Broad shifts over time in the global consumption of plastics categories were accounted for in P_2_O flows, but within each of the plastics categories we maintained a static polymer composition throughout lifecycle stages and the modelled timeframe (2016–40). The scope of our assessment for plastics included raw material extraction, monomer and primary polymer production, waste collection, industrial sorting, mechanical and chemical recycling (pyrolysis), incineration, landfill (engineered and dumpsites), open burning, aquatic and terrestrial macroplastics pollution, and the consequences of open-loop recycling (eg, secondary plastics and waste-to-fuel substitution). Product manufacturing and consumer use were not included in P_2_O and were therefore outside of our system boundary (figure 1). For each of the lifecycle stages, we included process-specific resources, energy and emissions, infrastructure, transportation, material losses, and waste management.

P_2_O considered three broad categories of plastics without distinguishing specific polymers (panel 1) but provided the proportions of product types included in each category (eg, water bottles, serviceware, and sachets).^18^^,^^25^ We estimated polymer profiles for each product on the basis of published literature (appendix pp 6–11), thereby converting P_2_O product-based categories to polymer-based categories of plastics (appendix p 12).

We compiled inventories accounting for short-term and long-term emissions for each polymer at each lifecycle stage (figure 1), using data from the Ecoinvent database versions 3.8 and 3.10^28^ (inventory model “allocation cut-off by classification”^28^) and published literature (appendix pp 14–30), where no dataset was available. Detailed descriptions of the inventories, assumptions, and modifications for each lifecycle stage are provided in the appendix (pp 14–30). Inventories for P_2_O geographical archetypes were adjusted with regional electricity mixes (ie, proportional reliance on fossil fuels and nuclear and renewable energy; appendix pp 52–57), and by climatic conditions where possible (appendix pp 27–29, 57). Polymer-specific inventories were combined to reflect 1 Mt of each P_2_O plastic category for each process and archetype (appendix p 12).

Detailed modelling of plastic material alternatives and substitutes was outside of the scope of P_2_O but the authors of this model provided estimates of the total plastic mass perceived to be substitutable by single-use paper, coated paper, and compostables; and by non-plastic household reuse and refill delivery schemes.^18^^,^^25^ Using expert stakeholder engagement, P_2_O assessed the quantity of plastics that could be substituted by scoring product categories according to perceived technology readiness level, performance, convenience, and cost within geographical archetypes and using time-based limits to reflect market penetration lags (appendix pp 30, 42).^18^^,^^25^ Full details of substitution by product category and geographical archetype are provided in the appendix of Lau and colleagues.^18^ Detailed descriptions of our lifecycle inventories, assumptions, modifications, and effects associated with each substitute system are provided in our appendix (pp 30–52).

We created illustrative lifecycle inventories for each single-use substitute on the basis of equivalent system boundaries. The influence of paper recycling rates and polylactide end-of-life management was explored in preliminary sensitivity analyses (appendix pp 34–42). In the assessment of P_2_O global system scenarios, we included alternative and substitute lifecycle variables resulting in lower adverse effects (75% recycling rate for paper and coated paper and 100% incineration for polylactide) to examine greater potential reductions in plastics’ adverse health effects at scale. Data on the equivalent mass of alternative materials required to substitute plastics vary substantially across the literature, even for the same product types (appendix p 33). We used a 1:1 mass substitution ratio to assess P_2_O global scenarios and conducted a further sensitivity analysis of this variable for the year 2040 (appendix pp 32–34, 80).

Drawing on published literature, we designed illustrative reusable glass systems, including reuse within households (appendix p 47) and door-to-door refill delivery models (appendix p 50), on the basis of providing equivalent food packaging service during 1 year. We accounted for the effects of glass production, household and industrial washing, distribution, and end-of-life management, and explored sensitivities to assumptions on container lifespans and delivery scheme scale and transport distances (appendix pp 42–52). We regionalised electricity inputs for all single-use and reuse substitute systems to account for differences between P_2_O geographical archetypes, and reflected regional differences in household dishwashing practices (appendix pp 37, 42, 52).

We reviewed impact assessment methods with human health endpoint characterisation factors. LC-Impact^29^ and Impact World+^30^ regionalise impact assessment, and USEtox 2.0^31^ characterises the broadest scope of chemicals but only for toxic effects. Given the limited regionalisation of our inventory datasets and our goal of accounting for multiple effect pathways, ReCiPe 2016^32^ (“hierarchic perspective”^32^) was considered the most appropriate, using a 100-year time horizon. We calculated all health-related midpoint effects (panel 2) and total endpoint human health DALYs associated with 1 Mt of each plastics category, for each process, in each geographical archetype, accounting for short-term and long-term emissions from all lifecycle stages. These lifecycle impact assessment results were used as characterisation factors to translate P_2_O plastics mass flows to equivalent health effects. The same approach was used to calculate DALYs and midpoint effects associated with the lifecycles of single-use substitutes and glass reuse systems.

Panel 2 **Human health impacts modelled in ReCiPe 2016 impact assessment method32**

**ReCiPe 2016 human health endpoint effects**

**Global warming**

• Impact pathway: greenhouse gas emissions drive increase in global mean temperature
• Human health impacts: morbidity and mortality due to increase in malnutrition, malaria, diarrhoea, and flooding

**Stratospheric ozone depletion**

• Impact pathway: emissions of ozone depleting substances drive decrease in atmospheric total ozone and increase in erythema from ultraviolet B
• Human health impacts: morbidity and mortality due to increase in skin cancers including malignant melanoma, basal cell carcinoma, and squamous cell carcinoma due to ultraviolet B exposure

**Photochemical ozone formation**

• Impact pathway: emission and atmospheric fate of nitrogen oxides (NOx) or non-methane volatile organic compounds
• Human health impacts: mortality due to increase in respiratory diseases such as asthma and chronic obstructive pulmonary disease

**Ionising radiation**

• Impact pathway: emission and dispersion of radionuclide
• Human health impacts: morbidity and mortality due to increase in cancers of the thyroid, bone marrow, lung, breast, bladder, colon, ovary, skin, liver, oesophagus, and stomach, and increase in severe hereditary effects

**Fine particulate matter formation**

• Impact pathway: emission and atmospheric fate of NOx, ammonia, sulphur dioxide, or fine particulate matter with aerodynamic diameter ≤2·5 μm
• Human health impacts: mortality due to increase in cardiopulmonary disease and lung cancer

**Human carcinogenic toxic effects**

• Impact pathway: emission and increased concentration of chemicals in the environment
• Human health impacts: morbidity and mortality due to increase in cancer incidence: mouth and oropharynx, oesophagus, stomach, colon and rectum, liver, pancreas, trachea, bronchus and lung, melanoma, and other skin cancer, breast, cervix uteri, corpus uteri, ovary, prostate, bladder, lymphomas and multiple myeloma, leukaemia^33^^,^^34^

**Human non-carcinogenic toxic effects**

• Impact pathway: emission and increased concentration of chemicals in the environment
• Human health impacts: morbidity and mortality due to increase in non-cancer incidence: (1) neuropsychiatric conditions: bipolar disorder, schizophrenia, epilepsy, dementia, Parkinson’s disease, multiple sclerosis, obsessive-compulsive disorder, and panic disorder; (2) sense-organ diseases: glaucoma and cataract; (3) cardiovascular diseases: rheumatic heart disease, ischaemic heart disease, and inflammatory heart disease; (4) respiratory diseases: chronic obstructive pulmonary disease and asthma; (5) diabetes; (6) digestive diseases: peptic ulcer and liver cirrhosis; (7) genitourinary diseases: nephritis and nephrosis and benign prostate hypertrophy; (8) musculoskeletal diseases: rheumatoid arthritis and osteoarthritis; (9) congenital anomalies: abdominal wall defect, anencephaly, anorectal atresia, cleft lip, cleft palate, oesophageal atresia, renal agenesis, Down syndrome, congenital heart anomalies, and spina bifida^33^^,^^34^

**Water use**

• Impact pathway: consumption of water drives reduced availability and water shortage for crop irrigation
• Human health impacts: morbidity and mortality due to increase in malnutrition and vulnerability of population

Adapted from ReCiPe 2016 version 1.1 (2017).^32^ Lifecycle impact assessment modelling pathways from specific emissions to health-related midpoint impact categories, and from midpoint impact categories to endpoint human health disability-adjusted life-years.^32^ Detailed description of toxicity-related health conditions were taken from van Zelm and colleagues^33^ and Huijbregts and colleagues.^34^

### Combining LCA with the Plastics-to-Ocean model

The P_2_O dataset^35^ was processed to multiply mass flows (Mt) by our LCA results for human health (DALYs per Mt). This included all plastics flows and those indicating the quantity of plastics substituted by alternative and substitute materials and reuse systems. The combined modelling accounts for avoided burdens of primary plastics production through closed and open loop mechanical and chemical recycling, and fuel production via waste-to-fuel pyrolysis (appendix pp 21–25). Total DALYs were calculated for each year of the global system (2016–40) under each scenario, by plastics category, geographical archetype, and midpoint indicator. We compared global DALYs between scenarios and over time, accounting for relative contributions of midpoint effects, processes, activities, and specific substance emissions.

### Sensitivity and uncertainty analyses

We tested the influence of key variables in the modelled lifecycle systems for single-use alternatives and substitutes and glass reusables through preliminary LCA sensitivity analyses (appendix pp 34–52). We conducted a sensitivity analysis of DALYs associated with P_2_O global system scenarios in 2040 on the basis of an evidence-based range of substitution ratios for paper-based alternatives (0·4–9·0), and for compostables (0·8–1·4); appendix pp 32–34, 80). Uncertainty was high across modelling stages, including within original P_2_O plastics flows, lifecycle inventory data, assumptions and extrapolations, and impact assessment methods. Statistical evaluation of summative uncertainty was not feasible or meaningful for these illustrative analyses. Instead, we provided transparent documentation and description of our inventories (appendix pp 6–58), narratively discussed impact assessment uncertainties, and quantitatively incorporated the original P_2_O Monte Carlo uncertainty analyses in our estimates of DALYs. We reported DALYs as the mean average and CIs of 300 simulations of each scenario, for each archetype, over the years 2016–40, conducted by P_2_O.^18^

### Statistical analyses

DALYs are a measure of population-level disease burden; one DALY represents 1 year of healthy life lost in a population due to the sum of morbidity and premature mortality associated with a given disease.^36^ We estimated DALYs associated with short-term and long-term emissions from plastics lifecycle stages, using a 100-year time horizon for impacts from the point of emission. Therefore, annual global health burdens refer to all health impacts associated with current and future substance emissions associated with a single year of plastics and alternative material flows in the global system, which might include short-term emissions with relatively near-term effects (eg, respiratory disease caused by industrial air pollution) and long-term emissions with possible latent effects (eg, cancers associated with toxic substances released over tens, hundreds, or even thousands of years from plastics gradual degradation in landfill and the natural environment). We did not quantitatively account for the benefits of plastics or their alternatives in our DALY estimates, but modelled comparisons on the basis of equivalent product function and service provision.

We used Simapro version 9.6.0.1 to compile life cycle inventories and conduct all LCA analyses and Stata version 17 to combine lifecycle impact assessment with P_2_O plastic flows. Global scenario results were further analysed and visualised in Excel.

### Role of the funding source

The funders of the study had no role in study design, data collection, data analysis, data interpretation, or writing of the report.

## Results

All plastics lifecycle stages generated emissions associated with adverse human health effects, mainly through: (1) global warming-related morbidity and mortality due to malnutrition, malaria, diarrhoea, and flooding; (2) fine particulate matter formation increasing cardiopulmonary disease and lung cancer mortality; and (3) human toxic effects encompassing morbidity and mortality from cancers and other non-communicable diseases.^32^ All unitary impact assessment results are available in the appendix (pp 59–65). For the same mass of plastics, DALYs varied substantially between stages, plastics categories, and geographical archetypes, particularly due to differences in emissions associated with our regionalised electricity production mixes (appendix p 55).

We present the results of combining LCA with P_2_O mass flows to estimate short-term and long-term human health effects associated with six global plastics scenarios between 2016 and 2040. All effect results are provided in the appendix (pp 66–88), including data by year, global system scenario, geographical archetype, midpoint impacts, lifecycle, stage, and substance contributions.

**Figure 2:**
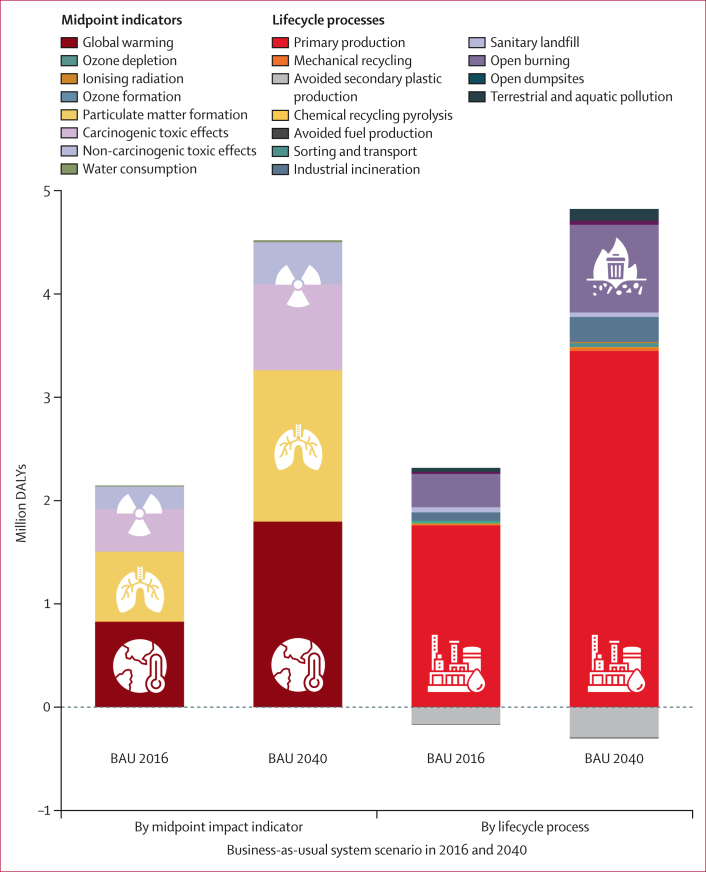
DALYs associated with 1 year of the P_2_O BAU global plastics system in 2016 and 2040 by contributing midpoint indicators and lifecycle processes Total DALYs associated with each year of the BAU scenario (2016 and 2040) are the sum of DALYs across all plastics lifecycle stages from health-related midpoint effects including global warming, ozone depletion, ionising radiation, ozone formation, fine particulate matter formation, carcinogenic toxic effects, non-carcinogenic toxic effects, and water consumption calculated using the ReCiPe 2016 lifecycle impact assessment method.^32^ DALYs by midpoint indicator represent the sum total DALYs after accounting for avoided effects from secondary plastics and fuel production (ie, the sum of all adverse [>0] and avoided [<0] effects). DALYs by lifecycle process appear greater overall because adverse effects associated with all plastics lifecycle stages (>0) are shown separately from avoided effects (<0). The BAU system scenario includes projected increases in the global human population and associated plastics demand between 2016 and 2040 with no change to current plastics-related policy, economics, infrastructure, materials, cultural norms, or consumer behaviours. Icons are additional illustrations of the midpoint indicators and life processes represented in the chart and described in the key. BAU=business as usual. DALY=disability-adjusted life-year. P_2_O=Plastics-to-Ocean.

In 2016, the global plastics system was associated with 2·1 million DALYs (95% CI 2·1–2·2 million DALYs based on P_2_O Monte Carlo uncertainty analyses for plastics flows), predominantly due to global warming (0·83 million DALYs, 0·79–0·86, 100-year timeframe) and air pollution (0·68 million DALYs, 0·65–0·70; figure 2). Primary plastics production resulted in 82% (1·8 million DALYs) of the total 2·1 million DALYs , with a further 15% (0·32 million DALYs) caused by open burning (or 76% and 14% of a total 2·3 million DALYs, respectively, if the avoided burdens of open-loop recycling and waste-to-fuel are not considered). Toxic chemicals released across the plastics lifecycle stages assessed were associated with 0·41 million DALYs (0·40–0·43) from cancers and 0·22 million DALYs (0·21–0·22) from other non-communicable diseases, including disease associated with short-term and long-term emissions. The combined effects of ozone depletion, ionising radiation, ozone formation, and water consumption contributed 0·5% (0·012 million DALYs) to total DALYs associated with the business-as-usual (BAU) scenario in 2016 (figure 2).

Between 2016 and 2040, P_2_O BAU projections resulted in a cumulative 83 million associated DALYs globally, increasing annually with 4·5 million DALYs (95% CI 4·4–4·7) associated with plastics produced and discarded in 2040. Rising health impacts were a function of the projected increase in plastics demand in P_2_O with no modelled changes to policy, infrastructure, economics, materials, or behaviours. Therefore, the proportional contributions of lifecycle processes and midpoint indicators to total DALYs were similar in 2016 and 2040 (figure 2).

Relative to BAU, alternative global scenarios including implementing government and industry commitments (2016–19) to address plastics pollution (current commitments) reduced cumulative health burdens associated with the global plastics system 2016–40 by just 4% (79 *vs* 83 million DALYs), increasing collection and disposal by 8% (76 *vs* 83 million DALYs), increasing recycling by 10% (74 *vs* 83 million DALYs), and reducing plastics use, partly through single-use material substitutions and glass reuse systems, reduced health effects by 17% (69 *vs* 83 million DALYs). The system change scenario, which combined all measures, provided the greatest reduction in cumulative global health burdens compared with BAU, with 21% (65 *vs* 83 million DALYs) less years of healthy life lost due to the global plastics system from 2016 to 2040.

**Figure 3:**
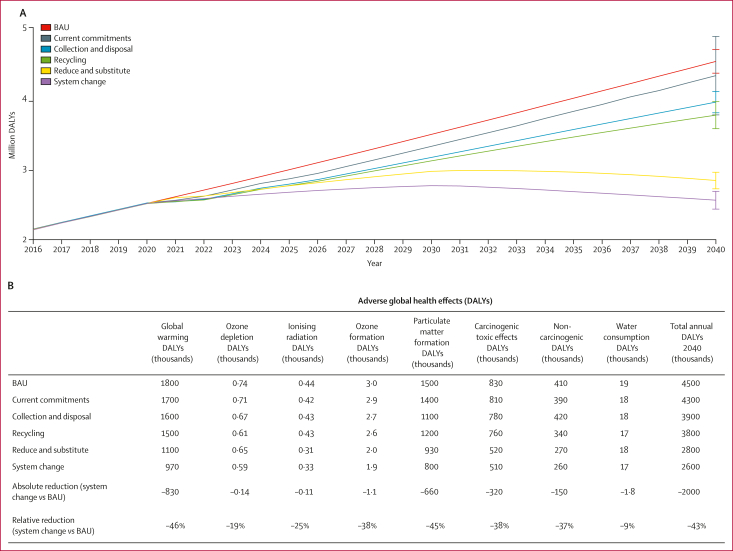
DALYs associated with six P_2_O model global plastics system scenarios for each year from 2016 to 2040 (A) Total DALYs associated with each year of P_2_O system scenarios between 2016 and 2040. (B) Total DALYs associated with P_2_O system scenarios in 2040 by midpoint indicators. In (B), absolute values are presented in thousands of DALYs and rounded to two significant figures. As a result, row totals and inter-column differences might have minor discrepancies due to rounding. Underlying data are available in the appendix (pp 66–80). Total DALYs are the sum of DALYs from health-related midpoint indicators calculated using ReCiPe 2016 lifecycle impact assessment method.^32^ BAU=business as usual. DALY=disability-adjusted life-year. P_2_O=Plastics-to-Ocean.

Nevertheless, all system scenarios were associated with rising health effects over time (figure 3A). By 2040, annual DALYs doubled under the current commitments scenario relative to 2016 (+2·2 million annual DALYs), increased by 84% (+1·8 million annual DALYs) in collection and disposal and by 75% (+1·6 million annual DALYs) in the recycling scenario. Reduce and substitute and system change scenarios were the only scenarios to show declining emissions and effects after a peak between 2030 and 2032. By 2040, health effects returned to levels associated with the years 2026–27 in the reduce and substitute scenario, and to those between 2021 and 2022 in the system change scenario (figure 3A). Sensitivity analyses revealed global DALYs under reduce and substitute and system change scenarios were strongly influenced by the ratio of plastics substitution by paper-based and polylactide alternatives (reduce and substitute 2040: 2·7–3·6 million DALYs; system change scenario 2040: 2·4–3·3 million DALYs). Based on these substitution ratios, the system change scenario reduced adverse health effects associated with BAU in 2040 by 46–28% (reduction of 2·1–1·3 million DALYs) and was favourable in comparison with other system scenarios in 2040 (appendix p 80).

Health burdens associated with the global plastics system under BAU in 2040 were reduced by between 0·2 and 2·0 million DALYs (–4 to 43%) under alternative system scenarios. This reduction included reductions in all health-related midpoint indicators, most substantially for global warming and particulate matter, followed by carcinogenic and non-carcinogenic toxic effects (figure 3B).

**Figure 4:**
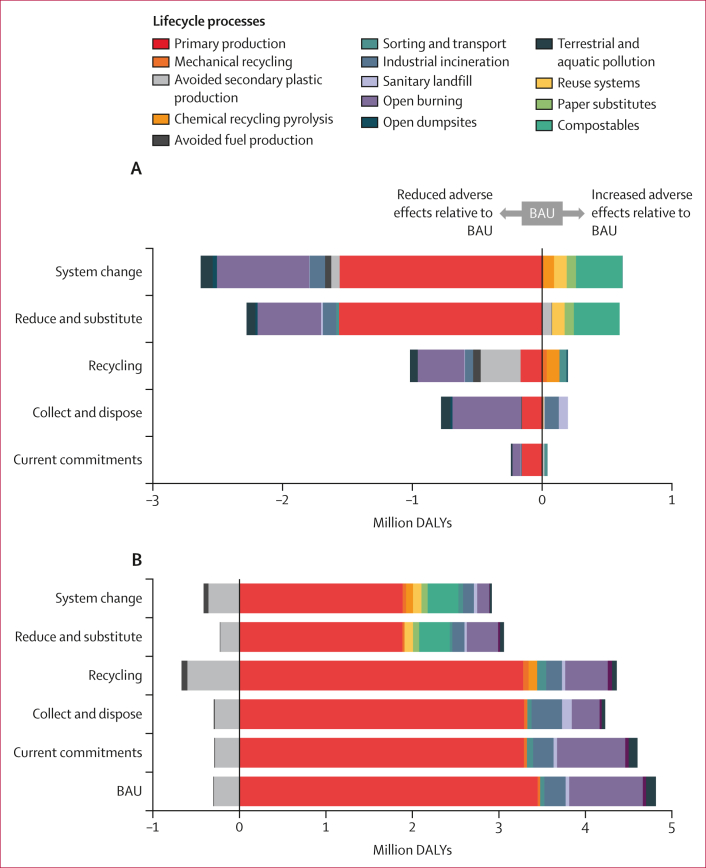
Lifecycle contributions to DALYs associated with P_2_O system scenarios in 2040 (A) Absolute difference in total system scenario DALYs by lifecycle stage relative to the BAU scenario for the year 2040. (B) Total DALYs associated with each P_2_O system scenario for the year 2040. Total DALYs are the sum of global DALYs in 2040 from health-related midpoint indicators calculated using the ReCiPe 2016 lifecycle impact assessment method.^32^ Absolute DALYs by lifecycle process in (B) show adverse effects associated with plastics lifecycle stages (>0) separately from avoided effects (<0). The total DALYs associated with each scenario is the sum of adverse and avoided effects. BAU=business as usual. DALY=disability-adjusted life-year. P_2_O=Plastics-to-Ocean.

The scale of absolute reductions in adverse health effects between scenarios was predominantly a function of the ratio of P_2_O modelled reductions in plastics production and open burning, relative to emissions introduced by increasing recycling technologies, plastics alternatives, and substitute systems (figure 4). Relative to BAU, reducing primary plastics production facilitated two-thirds of the associated alleviation of health burdens (0·2–1·6 million DALYs) in the current commitments, reduce and substitute, and system change scenarios in 2040, with reductions in open burning resulting in a further 22–28% (0·1–0·7 million DALYs) alleviated under the same scenarios. Within the overarching benefits of these scenarios, specific emissions increased relative to BAU as a result of increasing transport for collection (+0·02 million DALYs, current commitments scenario), increasing mechanical recycling (+0·005–0·013 million DALYs, current commitments and system change scenarios), chemical recycling (+0·1 million DALYs, system change scenario) and from all plastics alternatives and substitutes, particularly polylactide (+0·4 million DALYs in the reduce and substitute and system change scenarios). For recycling and collection and disposal, which maintained higher levels of primary plastics production, reductions in total DALYs relative to BAU were primarily due to reductions in open burning (39–68%, 0·4–0·5 million DALYs), although trade-offs included increased burdens from chemical recycling in recycling (+0·1 million DALYs) and from redirecting plastics to incineration (+0·1 million DALYs) and landfill (+0·07 million DALYs) in collection and disposal (although the health effects from landfills might only occur over the long term; figure 4).

**Figure 5:**
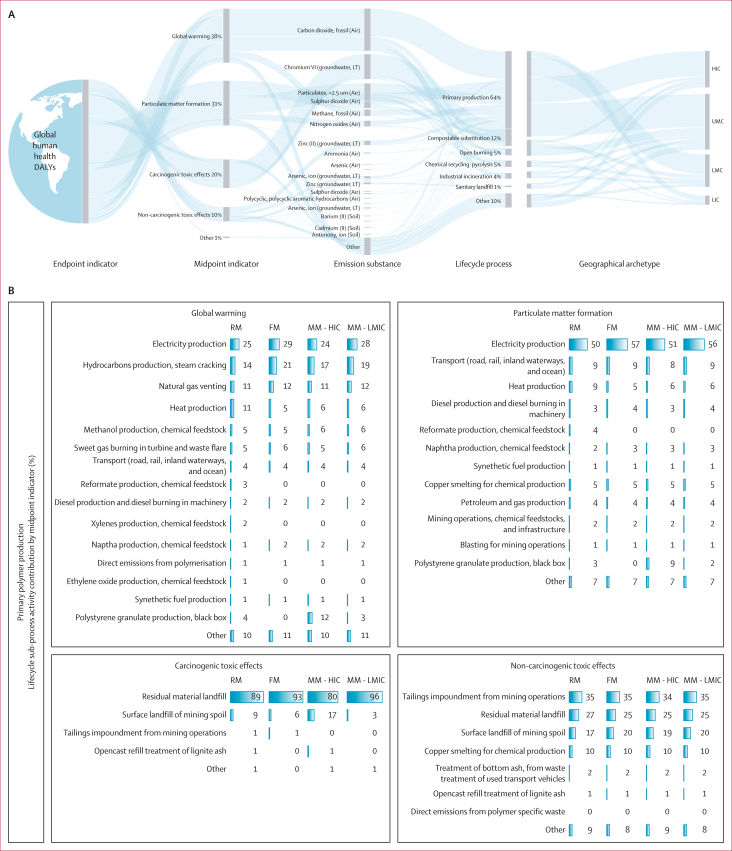
Contributions to total DALYs associated with the P_2_O system change scenario in 2040 (A) Sankey diagram of midpoint indicators, emission substance, lifecycle stage, and geographical archetype contributions to total DALYs in the system change scenario in 2040. Total DALYs shown by midpoint indicators contributing more than 5% of total global DALYs, by the substance emissions contributing more than 1% to each midpoint, and their provenance from lifecycle processes contributing more than 5% to any health-related midpoint indicator. All percentages in (A) reflect proportional contributions to total DALYs associated with the global system in 2040. Geographical archetypes indicate regional differences in lifecycle processes. These are shown separately to the main system because the location of primary polymer production and polylactide was not in scope of the P_2_O model. Links to geographical archetypes therefore reflect demand for these polymers rather than production. (B) Sub-process activity percentage contributions to primary polymer production by plastics category. DALY=disability-adjusted life-year. FM=flexible monomaterials. HIC=high-income countries. LIC=low-income countries. LMC=lower middle income countries. LMIC=low- and middle-income countries. LT=long-term emissions. MM=multilayer and multimaterials. P_2_O=Plastics-to-Ocean. RM=rigid monomaterials. UMC=upper middle-income countries.

The system change scenario was the most optimistic for health but the global system in 2040 was still associated with 2·6 million DALYs (95% CI 2·4–2·7 based on P_2_O Monte Carlo uncertainty analyses for plastics flows). Primary petrochemical plastics production were the leading source of adverse health effects (63% of total DALYs associated with system change scenario in 2040, 1·9 million DALYs), followed by polylactide alternatives (12%, 0·4 million DALYs), open burning (5%, 0·2 million DALYs), chemical recycling (5%, 0·1 million DALYs), and industrial incineration (4%, 0·1 million DALYs; figure 5). Electricity, predominantly produced from fossil fuels at the global level, resulted in a quarter (0·2 million DALYs) of greenhouse gas emissions associated with plastics production, followed by steam cracking operations for hydrocarbon production (14–21%, 0·1 million DALYs) and natural gas venting during raw material extraction (11–12%, 0·1 million DALYs). Electricity production was also the primary source of greenhouse gases and particulate matter associated with polylactide production (0·05–0·07 million DALYs respectively) and chemical recycling of petrochemical plastics (0·02–0·51 million DALYs respectively), although heat energy inputs and direct emissions from maize production and pyrolysis were also important (appendix p 88). Regionally differentiated electricity resulted in greater adverse effects in lower middle-income country archetypes for the same processes (appendix pp 59–65).

Material substitutes including paper, coated paper, and two systems of reusable glass containers each contributed 1–2% (0·03–0·05 million DALYs) of system change scenario 2040 effects. However, at higher substitution ratios, paper-based substitutes could account for 11% (0·4 million DALYs) of global effects under the system change scenario. These effects varied by geographical archetype due to regional electricity mixes and modelled differences in household practices for washing reusable containers by hand or electric dishwasher (appendix pp 59–65). Household washing resulted in 94–98% (0·04 million DALYs) of health effects in the consumer reuse system in different geographical archetypes. Relatively fewer DALYs were associated with industrial washing processes in the door-to-door refill delivery system (appendix pp 48–52) because of our assumed efficiency and capacity of industrial washing facilities, despite additional emissions from transportation (<10% of total effect) and greater material inputs.

Direct emissions of carbon dioxide were the main source of global warming-related DALYs from incineration and open burning (figure 5). Open burning also produced particulates of less than 2·5 μm and nitrogen oxides contributing to the respiratory effects of air pollution, and dioxins linked to carcinogenic toxic effects, particularly for P_2_O multilayer plastics, which included proportionally more polyvinyl chloride than other plastics categories. Groundwater contamination of chromium VI from treating residual and waste products from primary plastics production was the leading driver of carcinogenic toxic effects. Zinc, vanadium, and arsenic were the highest sources of non-carcinogenic toxic effects, linked to direct plastics landfill and the landfill of residual materials and bottom ash from producing petrochemical and polylactide plastics, pyrolysis, and incineration, although these releases might occur over long timeframes (figure 5; appendix pp 83–87).

## Discussion

We found that emissions throughout plastics lifecycles contributed to human health burdens of global warming, air pollution, toxicity-related cancers, and non-communicable diseases, with greatest harms from primary plastics production and open burning. Adverse health effects associated with the global plastics system more than doubled under P_2_O BAU projections for 2016–40. Alternative global scenarios centring on improving plastics waste management or increasing recycling alone were substantially less effective in reducing emissions and associated health burdens than scenarios that incorporated greater reductions in primary plastics production, underscoring the need for a full lifecycle approach to plastics pollution and harmful emissions. Globally implemented primary plastics production reduction, combined with improvements to waste collection and disposal, increased recycling, and replacing some plastics with alternative materials and reuse systems, reduced cumulative DALYs between 2016 and 2040 by 21% (18 million DALYs) relative to BAU projections, but still showed rising health burdens over time. Safer and more sustainable systems require deeper reductions in primary plastics production, alongside careful navigation of the effects of any alternatives or substitutes for plastics, which can be supported by using and developing LCA capacity to account for human health effects across material lifecycles.

Emissions from primary plastics production were the leading cause of health effects in all scenarios, including the system change scenario, which modelled a 45% reduction in primary production relative to BAU in 2040 (227 Mt *vs* 416 Mt in 2040, as shown in the original P_2_O plastics flow data^35^). Although transitions to renewable energy could alleviate some associated effects in terms of global warming and air pollution, this would not eliminate direct emissions from monomer production including natural gas venting, nor alleviate effects from plastics downstream waste management. More substantial reductions in primary polymer production would yield further reductions in emissions and associated health effects. An analysis by Chen and colleagues^37^ suggested that reducing global plastics production by 75% would reduce associated greenhouse gases emissions by 71%, with further reductions made possible by eliminating incineration and 100% greening of electricity supplies.

The absolute benefits of reducing primary plastics production in 2040 were partly constrained in P_2_O scenarios by the effects of increasing plastics recycling and replacing plastics with other materials. Mechanical recycling provided net benefits based on the P_2_O assumption that 17% of 2040 plastics demand could be fulfilled by recycled feedstocks and our crediting of open-loop recycling with averting production for lower grade plastics in other sectors. Both assumptions are debatable and require further analytical exploration. Chemical recycling was associated with net adverse effects due to high energy demand, despite the waste-to-fuel fraction credits for avoided fuel production. Other models have suggested that increasing recycling rates substantially and safely would require major advances and investment in technology and infrastructure, product redesign, and chemical simplification,^38^ and plastics might still breach planetary boundaries.^23^

Single-use material substitutes, including paper, coated paper, and polylactide each introduced their own lifecycle emissions to the global system with associated health effects. At the global level, polylactide was associated with the greatest adverse effects, partly due to P_2_O assuming a higher substitution rate of petrochemical plastics than for paper, and partly because of the high energy costs associated with polylactide production. Estimating the impacts of substituting petrochemical plastics with polylactide and other bio-based alternatives carries substantial uncertainty due to the multitude of possible materials on the market, for which there is an absence of accessible data on their value chain processes, relevant lifecycle inventories, and chemical composition. The most effective single lever for reducing overall global emissions and associated health impacts was to reduce primary production by eliminating unnecessary plastics with no material substitution.

Accounting for all plastics used in all sectors would increase our estimates of total lifecycle emissions and associated health burdens. This includes the sheer volume of plastics (approximately 53% increase to reflect total global plastics production) and from the greater use of more harmful polymers in specific sectors, such as polyvinyl chloride in construction. Specific sectors rely more heavily on specific waste management strategies—for example, the incineration of medical waste—and might be more or less amenable to reductions in plastics use, increased recycling, or material substitutions. A broader sectoral scope would influence the selection of alternatives and substitutes; glass might substitute plastics food packaging but not textiles in the fashion sector, for example. The function of plastics products should inform the prioritisation of unnecessary plastics that can be safely eliminated without compromising essential functions for society. Subsequently, any alternative materials or substitute systems used to provide essential functions should be comprehensively evaluated against the same safety and sustainability criteria as plastics to avoid substitutions that result in further harm to human health or the environment.^39^

We provide a model for estimating the impacts of emissions across plastics lifecycles under different global scenarios, but which currently underestimates crucial sources of adverse health effects. Our estimates of annual greenhouse gas emissions from primary plastics production were less than half of those estimated in two studies of global plastics production, comparing similar years (0·7–0·8 gigatons of greenhouse gas emissions denoted as carbon dioxide equivalents [GtCO_2_e] annually between 2016 and 2019 *vs* 1·9 GtCO_2_e for 2015^40^ and 2·24 GtCO_2_e for 2019^41^). The scope of plastics included in P_2_O was much narrower (approximately 64% of global plastics), product manufacturing was not included, and the inventory data probably underestimated emissions from raw material extraction and plastics chemical manufacturing.^42^ Industry-derived inventory data do not account for the significantly elevated cancer risk among people working in and living near to petrochemical industries, such as in so-called Cancer Alley in Louisiana, where measured levels of nitrogen oxides have exceeded Environmental Protection Agency estimates, and in specific areas reached up to 1000 times the safe limit.^43^

Air pollution is the leading risk factor in the Global Burden of Disease study, causing 225 million DALYs in 2021.^44^ Our results suggest that plastics lifecycles could cause more than 0·7% of global mortality from ambient particulate matter, which is likely to be a substantial underestimation globally, and which would be proportionally greater in regions with lower background ambient pollution and higher plastics production, such as in the USA. Our estimates of air pollution from open burning were also conservative; temperature ranges in uncontrolled fires are substantially lower than industrial incineration, resulting in greater particulate emissions.^45^ Black carbon is produced through incomplete combustion; it was not disaggregated in the available inventory datasets nor characterised in ReCiPe 2016, but is particularly damaging for respiratory health with possibly 900 times the global warming potential of carbon dioxide (100-year time horizon).^45^ Furthermore, open burning inventories do not reflect differences in plastics forms (eg, hard or soft), heterogeneous and mixed waste compositions, combustion characteristics, and the varying temperatures of uncontrolled fires, which influence quantity and composition of emissions.^46^

Hazardous chemicals contained in plastics might cause our results, and those of other LCA studies, to underestimate the human health impact of plastics by at least an order of magnitude. Bisphenols, flame retardants, perfluoroalkyl and polyfluoroalkyl substances, and phthalates, used to produce specific types of plastics or otherwise contained in some plastics materials and products, have been estimated to cause 33 million DALYs per year,^47^ or between US$250 billion^48^ and purchasing power parity $675 billion^4^ in health costs per year in the USA and between purchasing power parity $1·5 trillion^49^ and US$3·74 trillion^50^ globally. Although in specific regions these particular chemicals are now regulated for intentional use, with reduced human exposure, the absence of international harmonisation addressing their use across countries, products, and different materials, compounded by challenges of effective implementation and monitoring, means that these chemicals and others are still used or found as contaminants in plastics, which can be subsequently transferred to foods and humans,^51^ and transported around the world.^52^ More than 16 000 monomers, additives, processing aids and catalysts, and non-intentionally added substances have been identified in plastics, varying widely between polymers and products, and with poor data availability, although a quarter are known to be hazardous.^52^ These chemicals are largely missing, undecipherable, or included only as generic chemicals in lifecycle inventory datasets for plastics and other materials.^42^ Ensuring transparency on chemicals is essential for facilitating LCA studies that can more comprehensively assess health effects and therefore more effectively guide policy decisions on plastics and their alternatives.

Many other human health effects have yet to be characterised in LCA. The informal waste sector includes as many as 10–20 million people worldwide working in unsafe, unhygienic conditions on the front line of waste,^53^ where mixtures of plastics, other materials, organic matter, electronics, and hazardous waste pose compounding health risks that should be collectively addressed. Emerging evidence links the accumulation of plastics in the environment to reduced agricultural yields,^54^ aggravated flooding,^55^ and increased risk of infectious diseases.^56^ Microplastics and nanoplastics are pervasive across environments and have been identified in human tissues with early evidence of cell damage and immune responses.^57^ Microplastics and nanoplastic emissions across plastics lifecycles have yet to be fully assessed, which might influence the relative effects of different waste management strategies. Despite substantial gaps in data available for LCA analyses, even our conservative findings indicate that the global plastics economy is driving adverse health effects, which cannot be meaningfully mitigated without substantial reductions in primary plastics production alongside other strategies to promote safety and sustainability.

We provide a flexible, quantitative foundation for analysing global plastics systems that can be readily adapted with emerging data and lifecycle impact assessment methods to expand the scope and precision of modelled health effects. This framework can also be updated to reflect the changing sociopolitical context of plastics, including to account for the effects of national policies and trade restrictions introduced since 2019, international regulatory action such the Plastic Waste Amendments to the Basel Convention, and any future outcomes of the Global Plastics Treaty. Our results should not be interpreted as predictions but as quantitative illustrations of potential health effects across plastics lifecycles and global scenarios. LCA approaches include many limitations, including low availability of inventory data, significant sensitivity to assumptions and modelling choices, and uncertainty in impact assessment methods.

The absence of comprehensive inventory data was a major limitation, particularly for the composition and variability of chemicals contained in different petrochemical polymers, bio-based plastics, and substitutes including paper and glass. Mechanical and chemical recycling datasets were absent for most polymers. Our chemical recycling datasets were based on a single pyrolysis plant in Europe, and no data were available on emissions from informal recycling or plastics pollution remediation technologies. Inventories showed poor geographical representation. All were extrapolated from data collected or generated in Europe and North America,^28^ which could lead to underestimating emissions in regions with less regulation or fewer controls of waste management infrastructure and practices. We included short-term and long-term emissions in our assessments, informing the maximum burden of disease, although this might include emissions over thousands of years in the case of landfill, dumps, and plastics environmental degradation. All data sources and further discussion of their limitations are provided in the appendix (pp 6–58).

P_2_O global scenarios were defined on the basis of projected population growth and through stakeholder consultation conducted between 2018 and 2020, reflecting perspectives and data available at that time, which influenced the model’s defined trajectories of plastics consumption, trade, waste management, and alternatives to plastics. Product manufacturing and plastics’ use were not included, subsequently underestimating plastics lifecycle effects. We did not account for renewable energy transitions over time, which could reduce effects associated with several processes. To explore possible benefits of substituting plastics in the main system, we included the lower impact illustrative scenario among those we assessed, for each of our alternatives and substitute systems. Assumptions within these scenarios, including material specification and functional substitution ratios, reuse frequency, washing practices, transportation, lifespan, and waste management influence effects substantially and should be carefully evaluated in sectoral, geographical, and socioeconomically appropriate decision making. We did not account for any beneficial functions of plastics in DALY estimates, which will be needed in establishing priorities for eliminating and replacing products and materials. However, we ensured that scenarios of product substitution reflected equivalent functional service provision (ie, packaging the same quantity of food). Plastics were assessed in isolation from the rest of the municipal solid waste stream, therefore we did not account for the collective burdens of resource use, waste management, and mismanagement, nor the technical implications of mixed waste collection and sorting, which also need to be addressed and mitigated to protect human health. Further sensitivity analyses could guide priorities for primary data collection.

Although DALYs are useful for estimating and comparing the sum of healthy life lost from different diseases and premature mortality, this measure has been criticised on the basis of carrying inherent value judgements, particularly the weighting given to different diseases and disabilities, and for obscuring regional differences and the distinctions between morbidity and mortality.^58^^,^^59^ ReCiPe 2016 does not apply age weighting or discounting to DALYs but carries significant uncertainty in estimates of morbidity and mortality over a 100-year time horizon, posing challenges for interpretation and uptake in decision making. Nevertheless, advancing the capacity of LCA methods to account for and communicate health concerns, using recognised measures such as DALYs, is crucial to ensuring human health is systematically centred in evidence-informed actions to address plastics pollution.

Global production of plastics might not peak until after the year 2100, exacerbating environmental and health burdens in an already overwhelmed system. Globally implemented measures to mitigate plastics pollution could reduce plastics lifecycle emissions and associated health burdens but no P_2_O scenario provided a clear roadmap for a safe, sustainable system. Priorities to develop our model for assessing global scenarios for plastics include: (1) mandatory access to transparent, geographically representative data on the chemical composition of plastics and their existing or potential alternatives, validated measures of industrial process emissions, and international feedstocks, materials, products, and waste trade flows; (2) incorporating and developing LCA methods for health impact assessments across chemicals in products and the effects of microplastics and nanoplastics, including exposure during product use; and (3) prospective, spatiotemporally explicit LCA integrated with human rights, equity, and social justice considerations to inform localised and international decision making within a globally coordinated strategy to end plastics pollution and protect human health. This research would strengthen the imperative for safer, more sustainable and just systems and support effective policy development, monitoring, and evaluation. Meanwhile, there is clear evidence that plastics pollution, climate change, and global health are deeply interconnected challenges that require a full lifecycle approach in global policy to end plastics pollution and protect people and the planet.

## Data sharing

Full documentation of the inventory analysis processes, including all calculations and references, inventory data free of copyright, and impact assessment data is made available in the appendix with this publication. This excludes inventory data from ecoinvent, which cannot be distributed under the data licence.

## Declaration of interests

We declare no competing interests.
