# Supplementary material for: Global health burdens of plastics: a lifecycle assessment model from 2016 to 2040
Source: Lancet Planet Health. 2026 Jan 26;10(1):None. doi: 10.1016/j.lanplh.2025.101406 (PMC12867957; doi:10.1016/j.lanplh.2025.101406)
Supplement: Supplementary appendix [file mmc1.pdf]

THE LANCET
Planetary Health

Supplementary appendix

This appendix formed part of the original submission and has been peer reviewed.
We post it as supplied by the authors.

Supplement to: Deeney M, Hamelin L, Vialle C, et al. Global health burdens of plastics: a lifecycle assessment model from 2016 to 2040. *Lancet Planet Health* 2026. https://doi.org/10.1016/j.lanplh.2025.101406

Supplementary Material
Global health burdens of plastics: a life cycle assessment model from 2016 to 2040

Life Cycle Inventory Analysis and Impact Assessment

Megan Deeney[1*], Lorie Hamelin[2], Claire Vialle[3], Xiaoyu Yan[4], Rosemary Green[1], Joe Yates[1], Suneetha Kadiyala[1]

[1] London School of Hygiene & Tropical Medicine, London, United Kingdom
[2] Toulouse Biotechnology Institute, Université de Toulouse, CNRS, INRAE, INSA, Toulouse, France
[3] Laboratoire de Chimie Agro-industrielle (LCA), Université de Toulouse, INRAE, Toulouse, France
[4] University of Exeter, Exeter, United Kingdom
*Corresponding author: megan.deeney@lshtm.ac.uk

Table of Contents

OVERVIEW: Guide To Supplementary Materials 5

1. Converting Product to Polymer Categories 6
- **1.1. Data Sources for Polymer Composition of Plastic Products** 7
- **1.2. Conversion of Product-Based Categories of Plastic to Polymer-Based Categories** 8
- **1.3. Final Polymer-Based Estimates for Plastics-to-Ocean (P2O) Plastic Categories and Validation** 12

2. Inventory Data: Main Single-Use Plastic System 14
- 2.1. Plastic Production 14
  - **2.1.1. Virgin Plastic Production** 14
  - **2.1.2. Regionalisation Summary** 16
  - **2.1.3. Recycled Polymer Granulate Production** 16
- 2.2. Collection, Sorting and Transportation 16
  - **2.2.1. Municipal Mixed Waste Collection and Transport of Process Losses** 17
  - **2.2.2. Municipal Recycling Collection and Transport from Sorting Plant to Recycling Centre** 18
  - **2.2.3. Industrial Sorting** 18
  - **2.2.4. International Waste Trade Transport** 19
  - **2.2.5. Informal Sector Collection and Sorting** 21
  - **2.2.6. Littering and Pollution Removal** 21
  - **2.2.7. Regionalisation Summary** 21
- 2.3. Recycling 21
  - **2.3.1. Plastic Mechanical Recycling** 22
  - **2.3.2. Plastic Chemical Recycling** 24
  - **2.3.3. Regionalisation Summary** 25
- 2.4. End-of-Life Waste Management and Mismanagement 25
  - **2.4.1. Managed Waste: Industrial Incineration and Engineered Landfill** 26
  - **2.4.2. Open Dumpsites and Unsanitary Landfill** 27
  - **2.4.3. Open Burning** 28
  - **2.4.4. Terrestrial Pollution** 28
  - **2.4.5. Aquatic Pollution** 29
  - **3.4.6. Regionalisation Summary** 30

3. Inventory Data: Reduce and Substitute Options 30
- 3.1. Reduce Overview 30
- 3.2. Substitute Overview 30
- 3.3. Plastic Substitutes: Single-Use Paper and Coated Paper 34
  - **3.3.1. System Boundaries** 34
  - **3.3.2. Pulp and Laminate Production** 35
  - **3.3.3. Paper Recycling** 35
  - **3.3.4. Waste Management** 35
  - **3.3.5. Scenario Comparison** 36
  - **3.3.6. Regionalisation Summary** 37
- 3.4. Substitute: Single-Use Compostables 37

3.4.1. System Boundaries 38
3.4.2. Polylactide Granulate Production 38
3.4.3. Collection, Sorting and Transportation 38
3.4.4. Waste Management: Incineration 38
3.4.5. Waste Management: Composting 39
3.4.6. Scenario comparison 41
3.4.7. Regionalisation Summary 42
3.5. Substitute: Reusable Glass Systems 42
3.5.1. System Boundaries 44
3.5.2. Glass Production and End-of-Life Disposal 45
3.5.3. Substitution Rates 45
3.5.4. Consumer Reuse 47
3.5.5. New Delivery Model Reuse 48
3.5.6. Scenario Comparison 50
3.5.7. Regionalisation Summary 52

4. Regionalisation: Electricity Mix **52**
4.1. Modification of Existing Ecoinvent Electricity Datasets 53
4.2. Regionalised Electricity Mix Impact Comparison 54
4.3. Modification of Plastics-to-Ocean (P_2O) Processes with Regionalised Electricity Mixes 56

5. Results: Life Cycle Impact Assessment **59**

REFERENCES **89**

List of Tables

Table S1. Product composition of plastic categories in the Plastics-to-Ocean (P_2O) model in High-Income (HIC) and Low- and Middle-Income (LMIC) Geographical Archetypes[1] *6*
Table S2. Existing data sources for polymer composition of plastic product categories *7*
Table S3. Matching Plastics-to-Ocean (P_2O) model plastic product categories to polymer compositions *9*
Table S4. Summary of Virgin Plastic Production inventory *14*
Table S5. Summary of Plastic Collection, Sorting and Transportation inventories *16*
Table S6. International Plastic Waste Trade distances travelled by ocean freight (2010-2020) *20*
Table S7. Calculation of International Plastic Waste Trade distances travelled by ocean freight (2023) *20*
Table S8. Summary of Plastic Mechanical Recycling and Chemical Recycling inventories *22*
Table S9. Pyrolysis process inventory per kilogram of waste plastic *24*
Table S10. Avoided Fuel Production from Pyrolysis inventory *25*
Table S11. Summary of Waste Management and Mismanagement inventories *25*
Table S12. Geographic weighting for Open Dumpsite/Unsanitary Landfills *28*
Table S13. Comparison of polymer degradation rates under different environmental conditions[66] *29*
Table S14. Summary of life cycle inventories for single-use material substitutes (Paper, Coated Paper and Compostables) and reusable systems (Consumer Reuse and New Delivery Models) *31*
Table S15. Plastic product applications perceived as being substitutable by paper, coated paper and compostables in the Plastics-to-Ocean model[1] *32*
Table S16. Substitution ratios of petrochemical plastics by paper and bio-based polymers as identified in published literature *33*
Table S17. Paper and Coated Paper cradle-to-grave life cycle scenario comparison impact assessment on Human Health in Disability-Adjusted Life Years (DALYs) by midpoint impacts* *37*
Table S18. Polylactide (PLA) chemical composition[78] *39*
Table S19. European Commission Joint Research Centre (JRC) recommendations modelling industrial composting[72] *40*
Table S20. Estimated emissions to air per unit of polylactide (PLA) industrially composted *40*
Table S21. Polylactide (PLA) cradle-to-grave life cycle scenario comparison impact assessment on Human Health in Disability-Adjusted Life Years (DALYs) by midpoint impacts* *41*
Table S22. Categories of Plastics-to-Ocean (P_2O) plastic products assessed as substitutable within Consumer Reuse and New Delivery Models[1,2] and possible non-plastic materials for reusable systems *42*
Table S23. Substitution rates of single-use plastic packaging with reusable glass containers *44*
Table S24. Estimated product-based substitution rates of single-use plastics with reusable glass containers *46*
Table S25. Inventory data for dishwashing one reusable glass container during one year *48*

Table S26. Inventory data for handwashing one reusable glass container during one year 48
Table S27. Inventories for the reuse cycles of New Delivery Models on local and regional scales 49
*Table S28. Consumer Reuse cradle-to-grave life cycle scenario comparison impact assessment on Human Health in Disability-Adjusted Life Years (DALYs) by midpoint impacts** 50
*Table S29. New Delivery Model reuse cradle-to-grave life cycle scenario comparison impact assessment on Human Health in Disability-Adjusted Life Years (DALYs) by midpoint impacts** 52
Table S30. Ecoinvent datasets for national electricity production mix grouped by country income classification[52] 54
Table S31. Human Health impacts of estimated regional electricity production mixes in Disability-Adjusted Life Years (DALYs) 55
Table S32. Regionalisation modifications for each Plastics-to-Ocean (P_2O) life cycle process 56
Table S33. Total Disability-Adjusted Life Years (DALYs) associated with 1 Mt of each plastic category by life cycle process and health-related midpoint impacts 59
Table S34. Total Disability-Adjusted Life Years (DALYs) associated with global system scenarios by geographical archetype, plastic category and year 2016-2040 66
Table S35. Human Health midpoint impact contributions to total Disability-Adjusted Life Years (DALYs) associated with system scenarios in 2016 and 2040 80
Table S36. Sensitivity analysis of plastics mass substitution ratios by single-use paper-based substitutes and polylactide (PLA) alternatives on total Disability-Adjusted Life Years (DALYs) associated with relevant system scenarios in 2040 80
Table S37. Life cycle process contributions to total Disability-Adjusted Life Years (DALYs) associated with the Business-as-Usual system scenario in 2016 and 2040 81
Table S38. Life cycle process contribution to total Disability-Adjusted Life Years (DALYs) associated with all system scenarios in 2040 81
Table 39. Substance contributions to life cycle process, midpoint impacts and total Disability-Adjusted Life Years (DALYs) associated with the System Change scenario in 2040 83
Table 40. Sub-process activity contributions to total Disability-Adjusted Life Years (DALYs) associated with global system life cycle stages by midpoint impacts 88

List of Figures

Figure S1. Estimated polymer composition of Plastics-to-Ocean (P_2O) categories of plastic products 12
Figure S2. Validation of estimated polymer composition of Plastics-to-Ocean (P_2O) categories of plastic products. 13
Figure S3. System boundaries for cradle-to-grave life cycle scenarios for Paper and Coated Paper 34
*Figure S4. Paper and Coated Paper cradle-to-grave life cycle scenario comparison: impact assessment on Human Health in Disability-Adjusted Life Years (DALYs) by midpoint impacts** 36
Figure S5. System boundaries for cradle-to-grave life cycle scenarios of polylactide (PLA) 38
*Figure S6. Polylactide (PLA) cradle-to-grave life cycle scenario comparison: impact assessment on Human Health in Disability-Adjusted Life Years (DALYs) by midpoint impacts** 41
Figure S7. System boundaries for reusable glass systems based on Consumer Reuse and New Delivery Models 44
*Figure S8. Consumer Reuse cradle-to-grave life cycle scenario comparison: impact assessment on Human Health in Disability-Adjusted Life Years (DALYs) by midpoint impacts** 50
*Figure S9. New Delivery Model reuse cradle-to-grave life cycle scenario comparison: impact assessment on Human Health in Disability-Adjusted Life Years (DALYs) by midpoint impacts** 51
Figure S10. Electricity production mix by selected countries 53
Figure S11. Human Health impacts in Disability-Adjusted Life Years (DALYs) of estimated regional electricity production mixes based on Plastics-to-Ocean (P_2O) Geographical Archetypes 55

Table of Abbreviations

**Life Cycle Assessment**	
**LCI**	Life Cycle Inventory
**LCA**	Life Cycle Assessment
**Material Flow Analysis**	
**P_2O**	Plastics-to-Ocean Model
**Geographic Archetypes**	
**HIC**	High-Income Countries Archetype
**UMC**	Upper Middle-Income Countries Archetype
**LMC**	Lower Middle-Income Countries Archetype
**LIC**	Low-Income Countries Archetype
**LMIC**	Low- and Middle-Income Countries Archetype
**Plastic Categories**	
**RM**	Rigid Monomaterial Plastics
**FM**	Flexible Monomaterial Plastics
**MM**	Multilayer/Multimaterial Plastics
**Plastic Polymers**	
**PE**	Polyethylene
**LDPE**	Low-Density Polyethylene
**HDPE**	High-Density Polyethylene
**PP**	Polypropylene
**PS**	Polystyrene
**PVC**	Polyvinyl Chloride
**PET**	Polyethylene Terephthalate
**PETA**	Polyethylene Terephthalate - Amorphous
**PETB**	Polyethylene Terephthalate – Bottle-grade
**Organisations**	
**JRC**	European Commission Joint Research Centre
**OECD**	Organisation for Economic Co-operation and Development
**WRAP**	Waste & Resources Action Programme
**Units of measurement**	
**g**	Grams
**kg**	Kilograms
**kV**	Kilovolt
**kWh**	Kilowatt hour
**l**	Litres
**Mt**	Million metric tonnes
**tkm**	Tonne kilometres

OVERVIEW: Guide To Supplementary Materials

Our study combined Material Flow Analysis (MFA) with Life Cycle Assessment (LCA) to quantitatively estimate and compare the human health impacts in Disability-Adjusted Life Years (DALYs) associated with the life cycle of the most common plastics found in municipal solid waste, under different policy scenarios of global material management between 2016-2040.

We built our analyses on an existing MFA model, the Plastics-to-Ocean (P_2O) model[1,2]. This data-driven ordinary differential equation model described global flows of three categories of plastics products (Rigid Monomaterials, Flexible Monomaterials, Multilayer/Multimaterials), from material production to end-of-life fates including industrial waste management, mismanagement, and pollution.[1,2] The global model comprised sub-systems referred to as *geographical archetypes* (High-Income, Upper Middle-Income, Lower Middle-Income and Low-Income) to reflect differences in plastic material flows associated with country income classifications and population density.[1,2] P_2O tests the capacity of various policy scenarios to reduce marine pollution between 2016-2040, accounting for projected increases in plastic production. Scenarios included: (1) *Business-as-Usual*, (2) *Current Commitments* (full implementation of current (2016-2019) government and industry commitments), (3) *Collect and Dispose* (maximising waste collection and industrial disposal capacity), (4) *Recycling* (maximum foreseen recycling), (5) *Reduce and Substitute* (reducing plastic use, increasing reuse and material substitutions), and (6) a *System Change* scenario including all strategies.[1,2] We refer to P_2O categories of plastics, geographical archetypes and system scenarios throughout subsequent sections of the Supplementary Material.

The P_2O model produced data describing the mass of each category of plastic for each scenario, for each year between 2016-2040, and for each life cycle stage of the MFA system.[1,2] We paired this data with LCA; the inventory analysis for each process is presented in this Supplementary Material. In **Section 1,** we describe the conversion of P_2O product-based categories of plastics to polymer-based categories of plastics. The inventory analysis for the single-use plastic system is provided in **Section 2** and for the plastic substitute systems in **Section 3.** The functional unit for each process was 1 million metric tonnes (Mt) of plastics, or 1 Mt of plastics substituted during one year. The process of regionalising inventories by electricity production to reflect P_2O geographical archetypes is provided in **Section 4.**

We used secondary data from the Ecoinvent database versions 3.8 [3] and subsequent updates in 3.10 for the inventory analysis of each life cycle process. Ecoinvent is a life cycle inventory database that currently holds over 18,000 datasets that model human activities and processes, including the resources required such as electricity, water and raw materials, and the emissions generated including greenhouse gases and toxic chemicals, on different geographical resolutions.[3,4] Datasets are available to describe the geographically-relevant resources consumed and emissions generated by processes within a given country (e.g. *Switzerland*) or region (e.g. *Europe*). Ecoinvent also provides global datasets which are the weighted average of national or regional datasets designed to reflect the global market. Not all countries and regions are available as disaggregated datasets for each process. Ecoinvent provides a *Rest of the World* dataset for each process, which is extrapolated from existing data to estimate an average of countries and regions that are not covered separately within the database, and to support the construct of the *Global* average dataset.[3,4] The specific geographical relevance of datasets used in the inventory analysis are detailed throughout **Sections 1 – 3** of this Supplementary Material.

Life Cycle Impact Assessment (LCIA) for each life cycle process was conducted using the ReCiPe 2016 Hierarchic perspective impact assessment method[5] in Simapro 9.3[6]. The results are provided in **Section 5** with data for each of the graphs presented in the main manuscript.

1. Converting Product to Polymer Categories

The P_2O authors estimated that their modelling included about 64% of total global plastic production across all sectors.[1,2] The three compiled categories of plastics were based on municipal solid waste composition data from the World Bank *What a Waste* version 2.0[7] dataset and are designed to reflect the differences in possibilities for value recovery, supply and demand considerations, and the necessary infrastructure for material management.[1,2]

Products included in P_2O were primarily plastic packaging and single-use products, mostly related to food and drink, household goods and durable consumer products, personal care products, diapers and sanitary waste, cigarette butts and business-to-business packaging (**Table S1**).[1,2] Plastics used in building and construction, textiles and furnishings, transportation and machinery, medical waste, hazardous waste, electronics, agricultural and other industrial waste were out of scope of the P_2O analysis.[1,2]

Table S1. Product composition of plastic categories in the Plastics-to-Ocean (P_2O) model in High-Income (HIC) and Low- and Middle-Income (LMIC) Geographical Archetypes[1]

**Plastics-to-Ocean Plastic Categories and Product Proportions**					
**Plastic Category**	**Product**	**% plastic category in HIC**	**% total plastic in HIC (2016)**	**% plastic category in LMIC**	**% total plastic in LMIC (2016)**
RIGID MONOMATERIAL	WATER BOTTLES (Still water only, including bottle tops)	2%	1%	2%	1%
	OTHER FOOD GRADE BOTTLES (milk, soda, sparkling water, juice, concentrates, sports drinks, etc.)	15%	8%	15%	5%
	NON-FOOD GRADE BOTTLES (household, cosmetics. Includes spray tops, bottle tops and handles)	12%	6%	12%	4%
	FOOD SERVICE DISPOSABLES (Straws, stirrers, on-premises food service disposables, Off-premises plastic cups, lids, containers and clamshells, cutlery)	8%	4%	8%	3%
	POTS, TUBS AND TRAYS (Fresh fruit/vegetable tray/pot/punnet/tub, Pots/tubs for liquids and creams: yogurt, butter, spreads, chocolate/sweets, cream, chilled pot desserts and ice cream pots/tubs, Meat tray, Ready meals trays, instant pot snacks, Other	15%	8%	15%	5%
	B2B PACKAGING (Pallets, crates, Intermediate Bulk Containers (IBCs), drums and barrels and expanded polystyrene. Includes secondary and tertiary packaging)	8%	4%	8%	2%
	HOUSEHOLD GOODS (Cosmetics, toys, buckets, bowls, flip flops, small household objects, etc.)	13%	7%	13%	4%
	OTHER RIGID MONOMATERIAL PACKAGING (Consumer goods, EPS packaging, plastic egg boxes, blister packs, packaging clothes hangers, caps, and lids	27%	14%	27%	9%
	**TOTAL**	**100%**	**52%**	**100%**	**33%**
FLEXIBLE MONO-MATERIAL	CARRIER BAGS	17%	4%	17%	8%
	FILMS (Pouches, trash bags, wraps, 6-rings, netting and other flexibles)	67%	16%	67%	30%
	B2B FILMS: B2B shipping sacks, strapping, flexible intermediate bulk containers, bulk liners, and rolls)	16%	4.0%	16%	7%
	**TOTAL**	**100%**	**24%**	**100%**	**45%**
MULTIMATERIAL/ MULTILAYER	SACHETS AND MULTILAYER FLEXIBLES	17%	4%	80%	18%
	LAMINATED PAPER AND ALUMINIUM (Plastic component of laminated aluminium (e.g., toothpaste and aluminium cosmetics tubes), and of carton, paper, and aseptic cartons with >5% plastic coating, i.e., incompatible with paper recycling streams)	6%	2%	1%	0%
	HOUSEHOLD GOODS (MULTIMATERIAL) (Cosmetics, toys, pens, brooms, cigarette butts, small household objects)	67%	16%	9%	2%
	DIAPERS AND HYGIENE (PLASTIC PORTION) (Sanitary items, wet-wipes, cotton bud sticks, diapers)	10%	2.0%	10%	2%
	**TOTAL**	**100%**	**24%**	**100%**	**22%**

Notes: Replicated from the Supplementary Material of Lau et al (2020).[1]
Abbreviations: High-Income Countries Archetype (HIC), Low- and Middle-Income Countries Archetype (LMIC)

1.1. Data Sources for Polymer Composition of Plastic Products

There is relatively little publicly available data that describes specific polymers used in plastic products. We searched for published literature and industry reports and identified five suitable data sources to be used as core references on polymer compositions by product categories (**Table S2**). Data published by the Organisation for Economic Co-operation and Development (OECD)[8] and Plastics Europe[9] had the broadest geographical coverage and the most recent timeframe of data collection and analysis but included limited granularity of information for product categories. Both datasets described polymer proportions within sectoral categories, the most appropriate for P_2O being the Packaging (both), Consumer Products (OECD)[8] and Household Leisure and Sport categories (Plastics Europe).[9]

Data from Cimpan et al.[10], WRAP[11] and Deloitte[12] described polymer types by more granular product sub-categories of packaging including items such as films, bottles, pots, tubs, and trays. Both Cimpan et al. and Deloitte analyses were based on data from 2014 and referred to the European context, [10,12] WRAP data was obtained from supermarkets more recently (2017) but only within the United Kingdom.[11]

Table S2. Existing data sources for polymer composition of plastic product categories

Source	Data Time-frame	Geographic Coverage	Product Scope	Life Cycle Stage of Accounting
OECD (2022)[8]	2019	Global	All plastics, by categories: Packaging, Consumer Products, Textiles, Other	Plastic **waste generated**
Plastics Europe (2021)[9]	2020	EU 27 + Norway, Switzerland, and the United Kingdom	All plastics, by categories: Packaging, Building and Construction, Automotive, Electrical and Electronics, Agriculture, Household Leisure and Sport, Others	Converters **plastics demand**
Cimpan et al. (2021)[10]	2014	Europe	Packaging only: Foil, Sacks and Bags, Bottles PET, Bottles Other, Other Rigid Packaging	Plastic **production** and **placed on market**
WRAP (2018)[11]	2017	UK only	Plastic Packaging only (based on supermarket composition): Film, Carrier Bags, Bottles, Pots Tubs and Trays, Other<br><br>*These categories are detailed for Consumer/Retail Packaging and for Non-Consumer Packaging which includes applications in agriculture, construction, and commercial/industrial settings	Plastic packaging **placed on market**
Deloitte (2017)[12]	2014	France, Germany, Italy, Spain, and the UK, extrapolated to Europe	Plastic Packaging only: Bottle/flasks, Pots Tubs and Trays, Films<br><br>*These categories are described for Household Waste and for Commercial and Industrial Waste	Share of **waste generated**

Notes: Data sources were used to inform the conversion of Plastics-to-Ocean (P_2O) model product-based categories of plastics to polymer-based categories of plastics, and to validate our overall results for sectoral categories of plastics.

1.2. Conversion of Product-Based Categories of Plastic to Polymer-Based Categories

We estimated polymer profiles for each product sub-category in the P_2O model using available product-specific data, thereby converting each product-based P_2O category to a polymer-based category (**Table S3).** For each product subcategory, polymers were weighted according to the category's contribution to each of the broader P_2O plastic categories (**Table S1**). These contributions were then summed for each polymer, across product subcategories, to obtain overarching product-weighted polymer profiles for (1) Rigid Monomaterials, (2) Flexible Monomaterials, (3) Multilayers/Multimaterials in High-Income Countries Archetype, and (4) Multilayers/Multimaterials in Low- and Middle-Income Countries Archetype.

For example:

Water bottles account for 2% of the Rigid Monomaterial plastic category in P_2O. We estimated that the water bottle category comprised 95% PET, 3% HDPE and 2% PP. Therefore the contribution of each polymer used in water bottles to the P_2O category of Rigid Monomaterials is:

- PET: 95% x 2% = 1.9%
- HDPE: 3% x 2% = 0.06%
- PP: 2% x 2% = 0.04%
- Total = 2%

This was repeated for all categories and polymer estimates to obtain the overarching polymer profiles.

To provide a form of validation for the estimated polymer compositions, we also analysed the overarching estimates by creating a weighted total for packaging, and for household items that could be compared with the sectoral categories provided in OECD[8] and Plastics Europe[9] data. The results of this validation are presented in **Figure S2**.

Table S3. Matching Plastics-to-Ocean (P_2O) model plastic product categories to polymer compositions

P_2O Plastic Categories		Polymer Proportion Estimates (%)							Data Source	Notes And Justification
	Product Application	LD-PE	HD-PE	PP	PS	PVC	PET	Total		
RIGID MONOMATERIAL	WATER BOTTLES (Still water only, including bottle tops)		3.0 %	2.0 %			95.0 %	100.0 %	**Cimpan et al.:** Beverages: 1% Films, 1% Sacks and bags, 95% Bottles PET, 1% Bottles Other, 2% Other Rigid[10] **WRAP:** Plastics Drinks Packaging Total: HDPE 27%, PET 72%, Other 2%[11]	Cimpan et al. bottle specific estimates are the broadest in geographical scope but only include data for PET (95%).[10] This was supplemented by WRAP data on household waste bottles[11] and Deloitte data on consumer bottles to include the remaining 3% HDPE and 2% PP.[12] We assumed PET replaces higher levels of HDPE in WRAP[11] and Deloitte[12] data based on the properties required specifically for water bottles, which tend to be transparent as can be achieved with PET rather than semi-opaque as with HDPE[13]. The same typical polymers are referred to in data on bottle tops[14].
	OTHER FOOD GRADE BOTTLES (milk, soda, sparkling water, juice, concentrates, sports drinks, etc.)		3.0 %	2.0 %			95.0 %	100.0 %	*Same as above*	*Same as above* *Milk has previously been commonly packaged in HDPE due to higher temperatures needed for pasteurising[15] but this is reported to be in strong decline in favour of PET bottles and due to dietary changes away from dairy products[16] so Cimpan et al.[10] polymer proportions were maintained as these also reflect other soft drink categories included in P_2O.
	NON-FOOD GRADE BOTTLES (household, cosmetics. Includes spray tops, bottle tops and handles)		61.0 %	12.0 %			27.0 %	100.0 %	**Cimpan et al:** Personal and home care: 54.4% HDPE bottles, 24.1% PET bottles and jars, 10% Other bottles and jars.[10] **Deloitte:** 'Household Waste - Bottles/Flasks': 62% PET, 36% HDPE, 2% PP[12]	Cimpan et al. was the only dataset with disaggregated data for polymers in personal and home care bottles.[10] Proportions were scaled to 100% as films were also included in this category, which are not included in P_2O non-food grade bottles. Though Deloitte[12] and WRAP categories do not distinguish food grade and non-food grade bottles, the categories include bottles for cleaning and hygiene products, and concur roughly with each other,[11,12] therefore the third 'Other' category in Cimpan et al. was assumed to be PP.[10]
	FOOD SERVICE DISPOSABLES (Straws, stirrers, On-premise food service disposables, Off-premise plastic cups, lids, containers and clamshells, cutlery)		12.0 %	57.0 %			31.0 %	100.0 %	**Deloitte:** 'Household Waste - PTTs': 31% PET, 12% HDPE, 56% PP* [12] **Rounded to 57% to sum to 100%*	Large differences in datasets: Compared to Deloitte,[12] WRAP has much higher levels of HDPE, lower PET, half the PP, and PS is very high*.[11] Deloitte[12] data was used due to broader geographical coverage,[12] lower levels of HDPE to align more closely with overarching Plastics Europe data and lower PS to reflect recent bans in food serviceware[17–19] **Deloitte: 'Household Waste - PTTs': 31% PET, 12% HDPE, 56% PP[12]* **WRAP: 'Packaging in hospitality': HDPE 52%, LDPE 1%, PE 2%, PET 3%, PP 25%, PS 12%, PVC 5%[11]*
	POTS, TUBS AND TRAYS (Fresh fruit/vegetables, yogurt, butter, spreads, chocolate/sweets, cream, chilled desserts and ice cream, meat, ready meals, instant snacks, Other)		12.0 %	57.0 %			31.0 %	100.0 %	**Deloitte:** 'Household Waste - PTTs': 31% PET, 12% HDPE, 56% PP* [12] **Rounded to 57% to sum to 100%*	Large differences in datasets: WRAP includes much lower HDPE, lower PP and higher PET, PS and PVC*[11] Deloitte data[12] was used due to broader geographical coverage and regulatory shifts away from PS and PVC[17–20] **WRAP: 'Consumer (grocery and non-grocery)': HDPE: 3%, LDPE <1%, PE 1%, PET 52%, PP 28%, PS 11%, PVC 4.32%[11]*

P_2O Plastic Categories		Polymer Proportion Estimates (%)							Data Source	Notes And Justification
	**Product Application**	**LD-PE**	**HD-PE**	**PP**	**PS**	**PVC**	**PET**	**Total**		
	B2B PACKAGING (Pallets, crates, Intermediate Bulk Containers (IBCs), drums and barrels and expanded polystyrene. Secondary and tertiary packaging)		18.0 %	55.0 %	17.0 %		10.0 %	100.0 %	**WRAP:** 12.06% HDPE, 5.67% PE*, 9.93% PET, 54.61% PP, 17.73% PS[11]<br>**PE combined with HDPE*	Large differences in datasets: In comparison with WRAP[11] data, Deloitte has much higher PET and HDPE, lower PP though both show PP as the most common polymer in this category, WRAP also included high PS.[11]<br>WRAP data[11] was selected for this category despite high levels of uncertainty acknowledged in the original publication. WRAP[11] aligns more with Cimpan et al. which states that 100% of PS and 60% of PP is used in 'Other' rigid packaging, 40% of which is used in transportation of goods i.e. B2B packaging[10]<br>**Deloitte: 'Commercial and Industrial Waste - PTTs': 27% PET, 32% HDPE, 41% PP*[12]
	HOUSEHOLD GOODS (Cosmetics, toys, buckets, bowls, flip flops, small household objects, etc.)	21.0 %	15.0 %	44.0 %	14.0 %	6.0 %		100.0 %	**OECD:** Consumer products: PP 34.4%, Other Plastics 17.5%, HDPE 11.4%, PVC 5.1%, PS 10.7%.[8] Scaled to 100% to exclude 'Other Plastics' as polymers unclear, and to exclude PUR which is a thermoset not included in P_2O	OECD selected as the most geographically representative and recent data relating to consumer goods, though specific products are not detailed.[8] Deloitte, WRAP and Cimpan et al.[10–12] datasets only relate to packaging, therefore OECD consumer goods category[8] corresponds more closely to the household goods described in P_2O,[1,2] and reflects similar polymer proportions to those in Plastics Europe Household, Leisure, and Sports category.[9]<br>**OECD data does not distinguish between rigid/flexible/monomaterials/multimaterials; therefore the same distributions is applied across corresponding P_2O categories*[8]
	OTHER RIGID MONOMATERIAL PACKAGING (Consumer goods, EPS packaging, plastic egg boxes, blister packs, packaging clothes hangers, caps, and lids	12.0 %	28.0 %	38.0 %	1.5 %	0.5 %	20.0 %	100.0 %	**WRAP:** 'Consumer plastic packaging by format and polymer - Other': 28% HDPE, 12% LDPE, 20% PET, 38% PP, 2% PS, 1% PVC[11]<br>**PE 1% split 50/50 between HDPE and LDPE*	WRAP 'Other' category includes elements of packaging such as caps & lids, toothpaste tubes, chocolate/sweet wrappers, egg boxes, blister packs and clothing hangers,[11] aligning closely with products described by P_2O in this category. Deloitte data does not include PS or PVC,[12] but Cimpan et al. also suggests the 'other' category should include these polymers and uses WRAP data.[11]
FLEXIBLE MONOMATERIAL	CARRIER BAGS	33.0 %	67.0 %					100.0 %	**WRAP:** 'Consumer plastic packaging by format and polymer - Carrier bags': 67% HDPE, 33% LDPE[11]	WRAP is the only data source to provide separate data on carrier bags.[11] Predominance of LDPE and HDPE for plastic bags is also supported by a review of LCA on single-use plastic bags and their alternatives[21]
FLEXIBLE MONOMATERIAL	FILMS (Pouches, trash bags, wraps, 6-rings, netting and other flexibles)	66.5 %	12.0 %	16.0 %	0.5 %	2.0 %	3.0 %	100.0 %	**Deloitte:** 'Household Waste - Films': 3% PET, 12% HDPE, 69% LDPE, 16% PP[12]<br>**WRAP:** 'Consumer plastic packaging by format and polymer - Film': (2kt PS, 9kt PVC of total 395kt film)[11]	Deloitte[12] data selected for geographical coverage but adapted to include 2% PVC and 0.5% PS based on calculations taken from WRAP: 'Consumer plastic packaging by format and polymer - Film': (2kt PS, 9kt PVC of total 395kt film).[11] The 2.5% was subtracted from LDPE as the largest category so as to minimise changes to overall relative mix.<br>This was supported by Cimpan et al. data showing 23% of total PVC use is used for foil.[10]
FLEXIBLE MONOMATERIAL	B2B FILMS: B2B shipping sacks, strapping, flexible intermediate bulk containers, bulk liners, and rolls)	83.0 %		16.0 %			1.0 %	100.0 %	**Deloitte:** 'Commercial and Industrial Waste - Films': 1% PET, 83% LDPE, 16% PP[12]	Deloitte is the most geographically representative, disaggregated data on commercial and industrial flexibles only,[12] which corresponds to this P_2O category. Data from WRAP*[11] and other sources show similar distribution, for example data on netting[22]<br>**WRAP 2017: HDPE 3%, LDPE 68%, PE23%, PET 1%, PP 6%, PS 0%, PVC 0% Other 1%*[11]

P_2O Plastic Categories		Polymer Proportion Estimates (%)							Data Source	Notes And Justification
	Product Application	LD-PE	HD-PE	PP	PS	PVC	PET	Total		
MULTIMATERIAL/MULTILAYER	SACHETS AND MULTILAYER FLEXIBLES	66.5 %	12.0 %	16.0 %	0.5 %	2.0 %	3.0 %	100.0 %	**Deloitte:** 'Household Waste - Films': 3% PET, 12% HDPE, 69% LDPE, 16% PP[12] **WRAP:** 'Consumer plastic packaging by format and polymer - Film': (2kt PS, 9kt PVC of total 395kt film)[11]	Deloitte data selected for geographical coverage but adapted to include 2% PVC and 0.5% PS based on references [23] and calculations taken from WRAP[11]: 'Consumer plastic packaging by format and polymer - Film': (2kt PS, 9kt PVC of total 395kt film). The 2.5% was subtracted from LDPE as the largest category so as to minimise changes to overall relative mix. In the context of sachets, Unilever webpage supports the predominance of polyethylene in this application though does not specify between HDPE and LDPE.[24] The JRC supports the most common plastic being PE by a significant margin, also PET has some importance, and they suggest other plastics such as PA, but very limited information on proportions across multilayers so not included[23]
	LAMINATED PAPER AND ALUMINIUM (Plastic component of laminated aluminium (e.g., toothpaste and aluminium cosmetics tubes), and of carton, paper, and aseptic cartons with >5% plastic coating	63.0 %	37.0 %					100.0 %	**P_2O Supplementary Material:** assumes the plastic portion is PE[1] **Plastics Europe:** Packaging: LDPE 63%, HDPE 37%[9]	P_2O assumes PE based on WRAP data [25] but does not disaggregate by density, which we based on Plastics Europe overarching data for packaging[9]
	HOUSEHOLD GOODS (MULTIMATERIAL) (Cosmetics, toys, pens, brooms, cigarette butts, small household objects)	21.0 %	15.0 %	44.0 %	14.0 %	6.0 %		100.0 %	**OECD:** Consumer products: PP 34.4%, Other Plastics 17.5%, HDPE 11.4%, PVC 5.1%, PS 10.7%. Scaled to 100% to exclude 'Other Plastics' as polymers unclear,[8] and to exclude PUR which is a thermoset not included in P_2O[1,2]	OECD selected as the most geographically representative and recent data relating to consumer goods, though specific products are not detailed.[8] Deloitte, WRAP and Cimpan et al.[10–12] datasets only relate to packaging, therefore OECD consumer goods category[8] corresponds more closely to the household goods described in P_2O,[1,2] and reflects similar polymer proportions to those in Plastics Europe Household, Leisure, and Sports category.[9] **OECD data does not distinguish between rigid/flexible/monomaterials/multimaterials; therefore the same distributions is applied across corresponding P_2O categories*[8]
	DIAPERS AND HYGIENE (PLASTIC PORTION) (Sanitary items, wet-wipes, cotton bud sticks, diapers)	21.0 %	15.0 %	42.0 %	14.0 %	6.0 %	2.0 %	100.0 %	**OECD:** Consumer products: PP 34.4%, Other Plastics 17.5%, HDPE 11.4%, PVC 5.1%, PS 10.7%.[8] Scaled to 100% to exclude 'Other Plastics' as polymers unclear, and to exclude PUR which is a thermoset not included in P_2O[1,2]	OECD has broadest geographical coverage and most recent data on consumer products but is not specific to hygiene and personal care.[8] Several publications support the predominance of PP and PE in both diapers and menstrual products, though specific proportions are unavailable. Cotton buds are being widely banned[26–28] PET was mentioned in a UK study of diapers[29] and in a Swiss study of hygiene products[30] (though the proportion was not mentioned in the latter). For this reason we have supplemented with the UK data on 2% inclusion which will better reflect Plastics Europe data on Household products,[9] otherwise PS would not be included at all, subtracted from HDPE as the largest category so as not to alter overall distributions.

Notes: Detailed documentation of the matching process of product sub-groups included in the Plastics-to-Ocean model (P_2O) with likely polymer compositions based on available published literature. Abbreviations: Plastics-to-Ocean model (P_2O), Waste and Resources Action Programme (WRAP), Organisation for Economic Co-operation and Development (OECD), Life Cycle Assessment (LCA), Pots, Tubs and Trays (PTTs), Low-Density Polyethylene (LDPE), High-Density Polyethylene (HDPE), Polypropylene (PP), Polystyrene (PS), Polyvinyl Chloride (PVC), Polyethylene Terephthalate (PET).

1.3. Final Polymer-Based Estimates for Plastics-to-Ocean (P_2O) Plastic Categories and Validation

The product-weighted polymer compositions for each of the P_2O plastic categories are provided in **Figure S1**. Differences between P_2O categories in proportional composition of polymers aligned with typical polymer characteristics. For example, LDPE commonly produces flexible films that are translucent or opaque, whereas HDPE is harder, opaquer and can withstand higher temperatures[31]. PET has similar properties to HDPE but can be produced to be transparent and offers superior barrier properties[32]. In our estimates, PET is mostly found in the Rigid Monomaterials category, where transparent food contact containers predominate, whereas LDPE has a much greater presence in Flexible Monomaterials that include items such as grocery bags. The difference in Multi-layer/Multi-material polymer proportions between HICs and LMICs reflects the differences in proportions of products assumed in P_2O. Sachets accounted for 80% of this category in LMICs (vs. 17% in HICs), whereas in HICs the proportion of Multimaterial household goods such as cosmetics and toys was higher than in LMICs (67% vs 9% of this category). Sachets are flexible and predominantly made of LDPE, where household goods tend to be more durable, rigid plastic items. For this reason the polymer composition of Multi-layers/Multi-materials in HICs more closely corresponds to that of the Rigid Monomaterials and in LMICs it is closer to the Flexible Monomaterial composition.

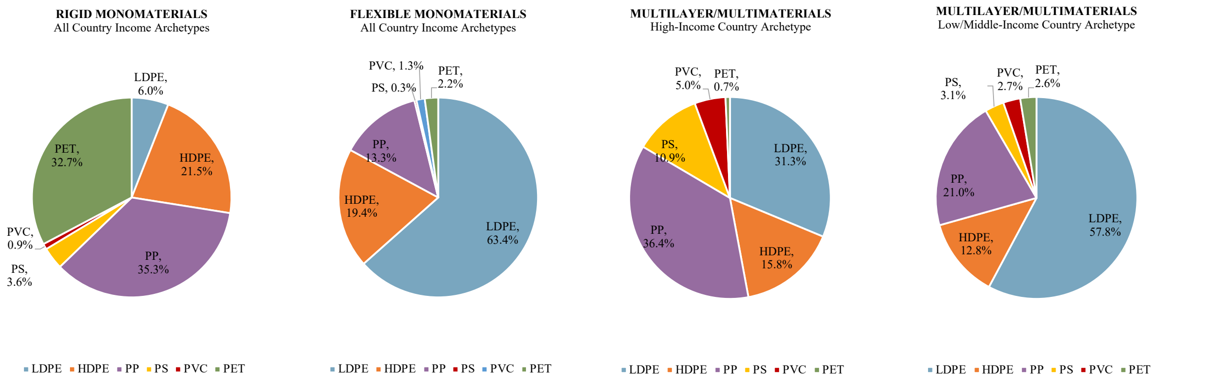

Figure S1. Estimated polymer composition of Plastics-to-Ocean (P_2O) categories of plastic products
Based on matching product sub-categories described in the P_2O model with likely polymer compositions to determine the overarching polymer proportions for plastic categories of Rigid Monomaterials, Flexible Monomaterials and Multi-layer/Multi-materials in High-Income Countries Archetype and in Low- and Middle-Income Countries Archetype. Abbreviations: Low-Density Polyethylene (LDPE), High-Density Polyethylene (HDPE), Polypropylene (PP), Polystyrene (PS), Polyvinyl Chloride (PVC), Polyethylene Terephthalate (PET).

Validating the results against polymer compositions estimated by Plastics Europe[9] and OECD[8] for (A) Packaging and (B) Household/Consumer Products showed good consistency with the results we obtained by matching polymers to P_2O product subcategories **(Figure S2).** Plastics Europe provided a European only estimate where OECD refers to the global scale. Our results for packaging align very closely with Plastics Europe estimates, particularly for HICs. The LMICs estimate contain higher levels of LDPE than OECD estimates for packaging but this is likely because OECD are global averages that do not account for the higher use of sachets present in the P_2O model in LMICs. For Household and Consumer goods, our estimates for P_2O categories were similar for HICs and LMICs, both showed lower presence of PVC and much higher PS and LDPE than Plastics Europe estimates but aligned closely with OECD. Given that OECD are the global estimates, the similarities lend some strength to our own estimates.

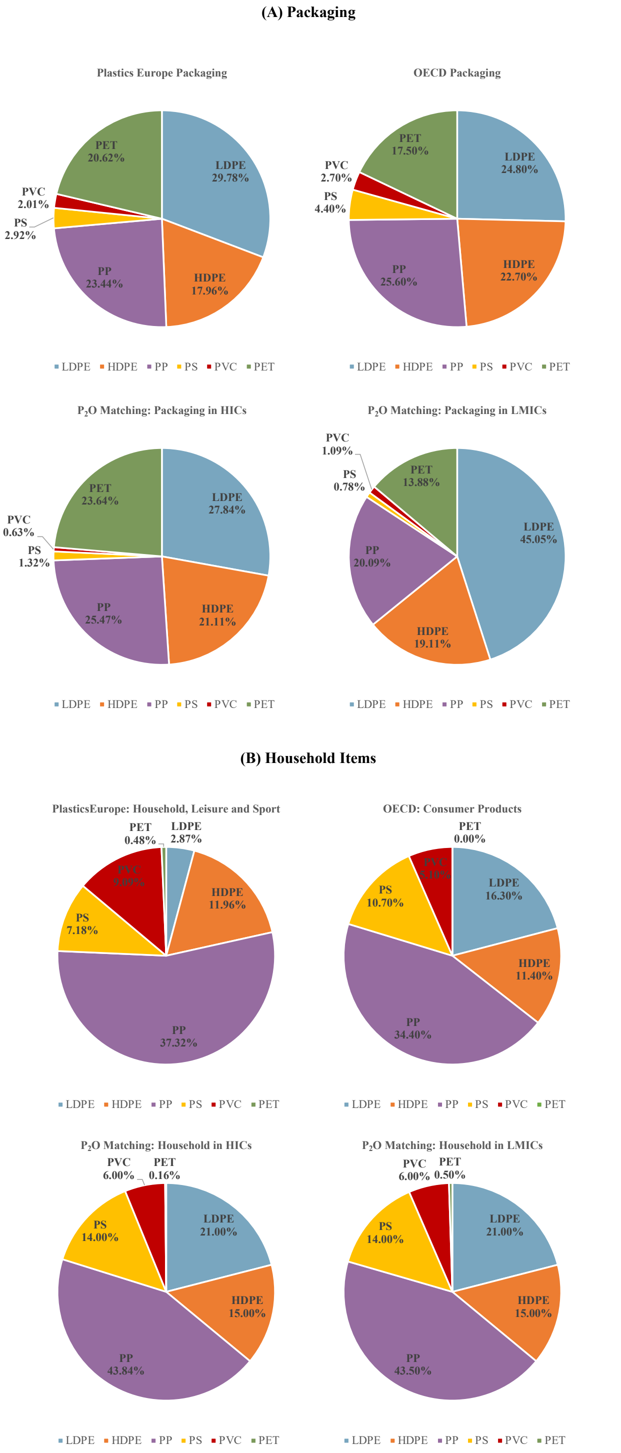

Figure S2. Validation of estimated polymer composition of Plastics-to-Ocean (P_2O) categories of plastic products.

Comparing: (A) polymer estimates for P_2O packaging products in High-Income Countries Archetype with Plastics Europe[9] packaging estimates, and polymer estimates for P_2O Low- and Middle-Income Countries Archetype with Economic Co-operation and Development (OECD)[8] packaging estimates; (B) polymer estimates for P_2O household products in High-Income Countries Archetype with Plastics Europe[9] estimates for Household, Leisure, and Sport items, and estimates for P_2O Low- and Middle-Income Countries Archetype with Economic Co-operation and Development (OECD)[8] Consumer Products estimates.

2. Inventory Data: Main Single-Use Plastic System

All LCI data were based on available Ecoinvent datasets[3] and published literature. Detailed descriptions of the inventory data and assumptions for each life cycle stage are available in the subsequent sections of the Supplementary Material.

2.1. Plastic Production

2.1.1. Virgin Plastic Production

P_2O estimates virgin polymer production on the basis of waste composition data, indicating demand. We used Ecoinvent datasets for production of specific polymers, combined based on our estimates of polymer composition, to reflect 1 Mt of production of each of the P_2O plastic categories (**Table S4**).

Table S4. Summary of Virgin Plastic Production inventory

Summary of inventory methods	Ecoinvent Inventory Data Source[3]	Primary Data Source*[3]
**SUMMARY APPROACH:** Combination of polymer-specific global datasets to reflect 1 Mt of P_2O plastic categories<br><br>**SCOPE OF DATASETS:** Process-specific resources, production of process inputs, emissions and waste treatment, industrial facility and land transformation, onwards transport from granulate production, including transportation infrastructure, vehicles, fuel, maintenance and emissions<br><br>**MODIFICATIONS:** Removal of recycled polymer production inputs to HDPE and PET datasets for 100% virgin polymer production, addition of chemical factory to PS dataset to ensure equivalence with other polymer datasets.	Ecoinvent: Market for LDPE granulate production (*Global*)	Plastics Europe (2011)[3]<br>Industry questionnaires in Europe, extrapolation for *Rest of the World*
	Ecoinvent: Market for HDPE granulate production (*Global*)	Plastics Europe (2011)[3]<br>Industry questionnaires in Europe, extrapolation for *Rest of the World*
	Ecoinvent: Market for PP granulate production (*Global*)	Plastics Europe (2011)[3]<br>Industry questionnaires in Europe, extrapolation for *Rest of the World*
	Ecoinvent: Market for general purpose PS granulate production (*Global*)	Plastics Europe (2001)[3,33]<br>Eco-profiles based on company survey
	Ecoinvent: Market for PVC, suspension polymerised granulate production (*Global*)	Plastics Europe (2013)[3]<br>Industry questionnaires in Europe, extrapolation for *Rest of the World*
	Ecoinvent: Market for amorphous PET granulate production (*Global*)	Plastics Europe (1999)[3]<br>Data from several European production sites and extrapolation for *Rest of the World*
	Ecoinvent: Market for bottle-grade PET granulate production (*Global*)	Plastics Europe (2015)[3]<br>Industry questionnaires in Europe, extrapolation for *Quebec* and *Rest of the World*

Notes: Details on the Ecoinvent dataset sources derived from Ecoinvent documentation for version 3.8 (cut off by classification).[3]
Abbreviations: Low-Density Polyethylene (LDPE), High-Density Polyethylene (HDPE), Polypropylene (PP), Polystyrene (PS), Polyvinyl Chloride (PVC), Polyethylene Terephthalate (PET).

Virgin plastic production inventories were based on polymer-specific global market Ecoinvent[3] datasets for virgin granulate production. We aimed to match Ecoinvent production datasets to each of the polymers present in our estimates for P_2O products including LDPE, HDPE, PP, PS, PVC, PET.

For LDPE, there were three available choices in Ecoinvent: global market for LDPE packaging film, LDPE granulate, or linear low-density polyethylene (LLDPE) granulate.[3] Packaging film is a product derived from LDPE granulate and therefore includes the granulate production plus an additional extrusion process to produce the film. As the product manufacture stage is outside of the system boundaries for this analysis, the granulate datasets were selected. LLDPE has a higher tensile strength than LDPE, can be made thinner and is more resistant to puncture and impact but is less easy to process.[31] Plastics Europe combine LDPE and LLDPE in a single category in their estimates of market shares[9]. In the absence of more granular data for products or markets, we selected the LDPE dataset for this analysis. This also offers greater consistency with subsequent life cycle modelling as LDPE is the only available option for all other processes.[3]

There was only one option for a global Ecoinvent dataset for the production of virgin HDPE.[3] The inventory contained a very small amount (<1%) of recycled HDPE content.[3] We replaced this with a virgin HDPE

production process from the same geography to ensure consistent modelling of virgin production across polymers. For PP, Ecoinvent provides global market datasets for granulate production or for PP non-woven textile production.[3] Similarly to LDPE, producing textiles includes the additional step from polymer production to product manufacturing, therefore the granulate production dataset was selected for consistency with system boundaries and the other polymers.

PVC is available in the form of bulk-polymerised, emulsion polymerised or suspension polymerised PVC.[3] Suspension polymerised PVC is reportedly by far the most common in usage, emulsion polymerized PVC tends to be used for coatings, and bulk polymerized for hard sheets and bottles that require high transparency and good plasticising properties.[34] Given that the P_2O products for which we estimated a proportion of PVC were more commonly flexibles and films, we selected the suspension polymerised PVC for 100% of PVC in this analysis.

Ecoinvent has four global market production datasets available for PS that include: extruded, general purpose, high-impact, and expandable polystyrene.[3] Extruded is described in Ecoinvent as being used in the construction sector,[3] therefore less relevant for P_2O products, and includes the extrusion process which would be inconsistent with other polymer datasets selected. General purpose polystyrene can be made into rigid or flexible film materials and can be transparent, offering greater scope than the high impact and expandable PS forms[3,35] to cover the range of products included in P_2O. Per unit of production, the general-purpose PS has the median impact in DALYs (using ReCiPe 2016 Hierarchic impact assessment) between the three forms and therefore offers a compromise in the uncertainty.

PET datasets include the global market for amorphous PET granulate production, and for bottle-grade PET granulate production.[3] The processes behind each dataset are similar though bottle-grade PET has an additional co-polymerization stage which includes the addition of benzene.[3] Benzene carries human health effects which are likely contributing to the differences in toxicity[3] between the two production processes. We used both datasets to reflect differences in P_2O products: for drinks bottles and food contact packaging we used the bottle grade dataset and for household products and non-food contact packaging the amorphous. This resulted in the following proportions of bottle-grade and amorphous PET for each P_2O plastic category:

- **RIGID MONOMATERIALS**: Total PET=32.72%, of which:
  - Bottle-grade PET=23.28% (Water Bottles, Other food grade bottles, food service disposables, pots tubs and trays) and,
  - Amorphous PET=9.44% (Non-food grade bottles, B2B packaging, household goods, other)
- **FLEXIBLE MONOMATERIALS:** Total PET=2.17%, of which,
  - Bottle-grade PET=1.35% based on P_2O stated assumption that of the films in this category 33% are non-food applications and 67% food contact.
  - Amorphous PET=0.82% (33% films (0.66%) and 100% of B2B films)
- **MULTILAYER/MULMATERIAL:** Total=0.71% in HICs, 2.43% in LMICs, of which:
  - All amorphous PET (sachets and multilayers, and diapers and hygiene products). Though some sachets are likely to be food packaging, analysis suggests PET is used as the outer non-food contact layer[23]

Both bottle-grade and amorphous PET market datasets contain very small amounts (<1%) of recycled content,[3] which was replaced with virgin production from the same geography for consistent modelling of virgin plastic production across polymers. All production datasets are derived from Plastics Europe, based on questionnaires sent out in 2011 to all production units in Europe operated by Plastics Europe member companies for LDPE, HDPE, PP and PVC, and in 2015 for bottle grade PET.[3] Data for amorphous PET were collected from European production sites in 1999 and for PS an industry survey in 2001.[3] Global geography data were extrapolated from these European data.[3] Datasets for each polymer include granulate production process-specific resources, emissions and waste treatment, production of process inputs, construction of the industrial facility and land transformation, and onwards transport from granulate production to the consumer, including transportation infrastructure, vehicles, fuel, maintenance and emissions.[3] PS was the only ‘black box’ dataset, which means that resources and emissions are provided as an aggregate list without indication of the sub-processes or inputs to which they belong. Reviewing the original data Eco-profile[33] revealed that the production facility was not included in the inventory. To ensure consistency with other polymer production datasets we included the same allocation of chemical factory production. Transport assumptions are the same for each polymer market production dataset.[3] Polymer-specific granulate production datasets were then combined proportionally according to **Figure S1** to create the inventory for the production of 1 Mt of each P_2O plastic category.

2.1.2. Regionalisation Summary

Total plastic input into the system was estimated by P_2O based on plastic waste generation data.[1,2] Therefore, estimates of virgin plastic inputs for each of the P_2O geographical archetypes correspond to regional demand for virgin plastic,[1,2] which does not necessarily equate to regional production. Plastic is a highly internationally traded commodity[36] and for this reason, we did not modify the Ecoinvent global market virgin polymer production datasets according to P_2O geographical archetypes. We considered this would better reflect the global market for plastic production that supplies the demand indicated in each of the P_2O geographical archetypes.

2.1.3. Recycled Polymer Granulate Production

In the P_2O system, recycled plastic granulate is obtained through (1) mechanical recycling, which directly produces recycled polymers that can substitute virgin polymer granulates, and through (2) chemical recycling pyrolysis that produces recycled monomers and hydrocarbons. For the purpose of this LCI, the latter required the addition of an extra polymerisation stage to deliver recycled polymer granulate equivalent to virgin production and mechanical recycling polymer production. The inventory for these processes is detailed with end-of-life recycling in **Sections 2.3.1. Plastic Mechanical Recycling, and 2.3.2. Plastic Chemical Recycling.**

2.2. Collection, Sorting and Transportation

The product manufacture and consumer use stages are not included in the P_2O[1,2] analysis and are therefore outside the system boundaries for this LCI. The next modelled life cycle stage following polymer production was post-consumer waste collection.[1,2] We compiled all waste transportation and industrial sorting inventories as global averages from existing geography-specific Ecoinvent datasets. We did not differentiate by polymers due to a lack of data, and because mostly the processes are similar or apply to mixed waste plastics (**Table S5**).

Table S5. Summary of Plastic Collection, Sorting and Transportation inventories

Summary of inventory methods	Ecoinvent Inventory Data Source[3]	Primary Data Source*[3]
**Municipal Mixed Waste Collection and Transport of Process Losses**		
**SUMMARY APPROACH:** Combination of Ecoinvent geographical datasets for mixed plastic waste collection to create global average for 1 Mt P_2O plastic categories weighted by country specific plastic waste generation data<br><br>**SCOPE OF DATASETS:** Geography specific transportation modes and distances, infrastructure and vehicle construction and maintenance, fuel production, emissions, waste treatment<br><br>**MODIFICATIONS:** Removal of end-of-life waste disposal processes from individual datasets to leave only transport assumptions	Ecoinvent: Market for waste plastic, mixture (multiple geographies)	Eurostat and Ecoinvent (2018)[3,37]
**Municipal Recycling Collection and Transport from Sorting Plant to Recycling Centre**		
**SUMMARY APPROACH:** Combination of Ecoinvent geographical datasets for source-separated recycling collection to create global average for 1 Mt P_2O plastic categories weighted by country specific plastic waste generation data<br><br>**SCOPE OF DATASETS:** Geography specific transportation modes and distances, infrastructure and vehicle construction and maintenance, fuel production, emissions, waste treatment<br><br>**MODIFICATIONS:** Removal of plastic waste input to estimate only impacts from transport processes. Removal of plastic collection container input as causing implausible impacts	Ecoinvent: Market for PET waste, unsorted for recycling (*Switzerland*)	Carbotech (2017)[38]
	Ecoinvent: Market for PET waste, unsorted for recycling (*Europe without Switzerland*)	Extrapolated from Franklin Associates (2011)[39]
	Ecoinvent: Market for PET waste, unsorted for recycling (*Rest of the World*)	Weighted average of *Switzerland*, *Europe without Switzerland*, *United States of America*[3]
	Ecoinvent: Market for PET waste, unsorted for recycling (*United States of America*)	Franklin Associates (2011)[39]
**Industrial Sorting**		
**SUMMARY APPROACH:** Use of Ecoinvent datasets for industrial sorting with updated inputs from JRC report.[40] Combination of	Ecoinvent: Waste PET, for recycling, sorting (*Switzerland*)	Carbotech (2017)[38]

Summary of inventory methods	Ecoinvent Inventory Data Source[3]	Primary Data Source*[3]
Ecoinvent geographical datasets to create global average for 1 Mt P_2O plastic categories weighted by country specific plastic waste generation data<br><br>**SCOPE OF DATASETS:** Process specific resource requirements and related emissions, facility infrastructure but not machinery<br><br>**MODIFICATIONS:** Update of quantities of electricity, heat, diesel, and propane, regionalisation of datasets by geography and regional electricity mix	Ecoinvent: Waste PET, for recycling, sorting (*Europe without Switzerland*)	Extrapolated from Franklin Associates (2011)[39]
	Ecoinvent: Waste PET, for recycling, sorting (*Rest of the World*)	Weighted average of *Switzerland*, *Europe without Switzerland* and *United States of America*[3]
	Ecoinvent: Waste PET, for recycling, sorting (*United States of America*)	Franklin Associates (2011)[39]
**International Waste Trade**		
**SUMMARY APPROACH:** Use of Ecoinvent dataset for ocean freight travel using estimated distance between major plastic waste exporting and importing countries to create global average for 1 Mt P_2O plastic categories<br><br>**SCOPE OF DATASETS:** Production of container ship and maintenance, construction of port facilities, fuel and emissions, waste treatment<br><br>**MODIFICATIONS:** None	Ecoinvent: Market for transport freight, sea, container ship (*Global*)	International Maritime Organization and published literature (2015)[3]
**Informal Sector Collecting and Sorting**		
**SUMMARY APPROACH:** No process-specific resources or emissions modelled	N/A	N/A
**Littering and Pollution Removal**		
**SUMMARY APPROACH:** No process-specific resources or emissions modelled	N/A	N/A

Notes: *Details on the Ecoinvent dataset sources derived from Ecoinvent documentation for version 3.8 (cut off by classification)[3]. Abbreviations: Low-Density Polyethylene (LDPE), High-Density Polyethylene (HDPE), Polypropylene (PP), Polystyrene (PS), Polyvinyl Chloride (PVC), Polyethylene Terephthalate (PET).

2.2.1. Municipal Mixed Waste Collection and Transport of Process Losses

Mixed waste collection by the 'formal sector' includes municipal waste collection of unsorted plastic waste from homes and businesses which is intended for incineration and landfill.[1,2] Ecoinvent contains national waste market datasets the end-of-life fate of mixed plastic waste and transport estimates.[3] Datasets are available for geographies including *Albania, Austria, Belgium, Bosnia and Herzegovina, Brazil, Bulgaria, Columbia, Croatia, Cyprus, Czech Republic, Denmark, Estonia, Finland, France, Germany, Greece, Hungary, Iceland, India, Ireland, Italy, Kosovo* (not included in the LCI as national waste generation data was not available), *Latvia, Lithuania, Luxembourg, Macedonia, Malta, Montenegro, Netherlands, Norway, Peru, Poland, Portugal, Romania, Serbia, Slovenia, Slovakia, South Africa, Spain, Sweden, Switzerland, United Kingdom,* and *Rest of the World.*[3] Ecoinvent transportation assumptions are based on Eurostat 2018[37] data for European countries, the Ecoinvent own transport model for non-European countries, and extrapolated using expert opinion for the *Rest of the World* dataset[3]. All data relate to different distances travelled by freight lorries based on the European market, except for India that uses the *Rest of the World* market and South Africa that uses a national-specific market, the Rest of the World dataset also includes some rail transportation of mixed plastic waste.[3]

We removed the end-of-life processes to obtain transport-only inventories for each country, combining datasets proportionally according to national contribution to global plastic waste generation[41,42] to create a weighted average global mixed waste plastic transportation inventory. The resulting inventory includes freight lorry manufacturing and maintenance, road construction and maintenance, fuel production (diesel), transport-specific emissions to air, and the waste treatment of break, road, and tyre-wear emissions.[3]

Compared to an LCA of plastics in Europe by the European Commission Joint Research Centre (JRC)[40], our transport assumptions are higher for mixed waste collection. JRC estimated 13.2tkm/t for mixed waste collection[40], whereas using Ecoinvent resulted in 68.4tkm/t for the European average.[3] This is likely because the JRC transport accounted for the transportation from point of collection to a central hub[40], whereas Ecoinvent includes onwards transportation to incineration and landfill.[3]

The transportation of losses from all collection, formal sorting and recycling processes are included as separate flows in P_2O.[1,2] We have applied the same mixed waste transportation assumptions to these flows, treating waste management industries as the same as any other businesses requiring municipal waste collection services. This is consistent with how Ecoinvent models the collection of losses in their recycling datasets.[3]

2.2.2. Municipal Recycling Collection and Transport from Sorting Plant to Recycling Centre

Recycling collection refers specifically to the collection of plastic waste from homes and businesses that is intended for recycling. It has therefore been separated at source and takes a different pathway than the municipal waste collection for landfill and incineration.[1,2] Ecoinvent datasets are available for the transportation of waste PET or PE in the geographies of *Switzerland*, *Europe without Switzerland*, the *Rest of the World,* and the *United States of America.*[3] The differences in datasets between polymers were relatively small and the reasons for differences were unclear,[3] so we conservatively selected the PET datasets as the basis for the recycling collection inventory as it included slightly higher tonne kilometre transport assumptions. These datasets include geography-specific transportation modes and distances for the collection of source-separated plastics through residential curbside, or drop-off programmes, deposit redemption systems, and commercial collection programmes, delivery to industrial sorting or recycling centre.[3] Swiss data were based on an industry source of PET bottle collection (2017)[38] including transportation by diesel passenger car and freight lorry, and other geographies were extrapolated from USA data, based on a multi-source analysis by Franklin Associates (2011),[39] also including transportation by diesel passenger car and freight lorry in *Europe without Switzerland* and the *United States of America*, with the addition of rail transportation in the *Rest of the World*.

Inventories for all transportation forms include data on resources and emissions relating to vehicle manufacturing and maintenance, road, or rail construction (including land transformation) and road maintenance, diesel or electricity production, transport-specific emissions to air, iron emissions to soil from rail transportation and the waste treatment of break, road, and tyre-wear emissions from road transportation.[3]

The JRC LCA based transportation assumptions on Rigamonti et al. (2013)[43] for separate (mono-material) collection of municipal plastic waste through kerbside and drop-off collection systems.[40] Referring to a well-developed waste management scheme in northern Italy, JRC considered that these data could be representative of several other regions in Europe.[40] We chose to use the Ecoinvent data as it had a broader geographical scope, more relevant to our global analyses, and for consistency with the data we used for mixed waste collection. However, we did use the JRC relative differences in transport distances for different stages of collection to apply these distances to the different P_2O flows: from consumer to sorting centre (65% of total transportation distance) and sorting centre to recycling facility (35%) as this was not available in Ecoinvent or Franklin Associates (2011).[39] These proportions were applied to all transport types included in the Ecoinvent datasets except for passenger cars, for which we assumed 100% of car usage related to collection and drop of stages immediately following consumer use. We also removed collection containers from the Ecoinvent datasets, as these were generating implausible results, and removed the plastic waste input to obtain the impacts of transportation alone.[3] The impacts of the plastic itself are accounted for in other P_2O compartments.

We created a weighted average global dataset using data on national contribution to plastic waste to estimate the proportion of each geographical dataset: *Switzerland (0.4%)*, *Europe without Switzerland (13%)*, the *Rest of the World (71%)* and the *United States of America (15%).* Basing the geographical proportions on national plastic waste contribution is consistent with the approach for municipal solid waste transportation and therefore the differences in impacts between mixed waste and recycling collection will more closely represent the difference systems. Eventually, the geographical proportions within the global estimate could be more accurately assessed based on national recycling collection statistics, but these are not currently readily available for plastics specifically and reflect different considerations from data that may be available on national recycling capacity, total recycling including international exports, and recycled plastic production, which all produce very different statistics. Given that there are only four inventory geographies available in Ecoinvent, this is likely to minimally affect results.

2.2.3. Industrial Sorting

Formal sector sorting is assumed to be conducted via industrial machinery or municipal solid waste facility staff in P_2O.[1,2] The only Ecoinvent datasets available on plastic sorting processes are for PET and PE in the geographies of *Switzerland*, *Europe without Switzerland*, the *Rest of the World,* and the *United States of America.*[3] Both PE and PET datasets include the same inventory resource inputs but slightly different quantities.[3] The Swiss inventory is based on confidential industry data and other geographies are based on

Franklin Associates (2011),[39] or a weighted average of the two (*Rest of the World*). We compared these data with those used in the JRC LCA for the plastic sorting process,[40] which were taken from an updated report by Franklin Associates (2018)[38] adapted for European inputs to inform a single inventory applied across all polymer types.[40] We used this data to modify the quantities of electricity, heat, diesel, and propane in Ecoinvent datasets to reflect the more recent estimates, whilst maintaining the geographical relevance of inputs as in the original Ecoinvent datasets.[3] To obtain the inventory for the sorting process alone, we removed plastic waste inputs and the treatment of losses as these are already taken into account in other compartments of P_2O and would otherwise result in double counting.

The final inventories include the construction of the waste preparation facility and steel inputs, diesel, electricity, heat, and propane. No process specific emissions are detailed. We created a weighted average global dataset based on national plastic waste generation data,[41,44] consistent with the modelling of other categories in the collection, sorting and transportation phase. Once more data is available, geographical proportions could be weighted according to the number or capacity of sorting facilities for more precise results.

2.2.4. International Waste Trade Transport

International trade data provides some insight into the legal exports of plastic waste around the world.[36] It is estimated that since tracking began in 1988, almost a quarter of a billion metric tonnes of plastic waste has been internationally traded, with the USA, Japan, and Germany accounting for more than a third of exports.[45] These estimates do not include illegal trade or misclassification of plastic waste, or plastic waste traded under other shipment codes. Annual trade can fluctuate substantially and is highly subject to politico-economic changes, for example the Chinese ban on plastic waste imports in 2018.[46] As China was historically the top importer, the ban drove imports up across many other South-East Asian countries.[45,46]

We originally estimated the nautical distance between the major ports of the top four exporting countries (USA, Japan, Germany, United Kingdom) and the top four importing countries (Hong Kong, Malaysia, India and Vietnam) for the period 2010-2020 using an online calculator[47] **(Table S6).**

The limitations of this approach include the lack of detail on all existing plastic waste trade routes between countries, and their fluctuation over time, the lack of weighting of the average distance by the quantity of plastics transported along different routes, and the lack of consideration of transport modes other than ocean freight as a substantial proportion of plastic waste is traded within regions and may not require shipping.

A subsequent update was conducted for the year 2023 using the top four exporting countries (Germany, Japan, Netherlands, United States of America) and the top four importing countries (Netherlands, Turkey, United States of America, Indonesia) by quantity of plastic waste traded according to the United Nations Comtrade Database (2023) using Commodity Code 3915 (**Table S7).** We converted nautical miles to kilometres and took the mean average distance across combinations of countries to inform the inventory of 1 Mt of internationally traded plastic waste. Kilometres were multiplied by 1,000,000 to get the tonne kilometres (tkm) for our functional unit of 1 Mt plastic and we assumed transport was conducted by ocean freight shipments.[45] Additional elements of transport, for example road or rail transportation to and from the port were not included due to high uncertainty and lack of data.

The limitations of this approach include the lack of detail on all existing plastic waste trade routes between countries, and their fluctuation over time, the lack of weighting of the average distance by the quantity of plastics transported along different routes, and the lack of consideration of transport modes other than ocean freight as a substantial proportion of plastic waste is traded within regions and may not require shipping.

Table S6. International Plastic Waste Trade distances travelled by ocean freight (2010-2020)

Major Port (Exporting Country)	Major Port (Importing Country)	Nautical miles	Kilometres
United States of America (New York)	Hong Kong (Kwai Chung)	11,207	20,755
	Malaysia (Port Klang/Swettenham)	9,936	18,401
	India (Mumbai)	8,165	15,122
	Vietnam (Hai Phong)	11,455	21,215
United States of America (Los Angeles)	Hong Kong (Kwai Chung)	6,363	11,784
	Malaysia (Port Klang/Swettenham)	7,879	14,592
	India (Mumbai)	10,104	18,713
	Vietnam (Hai Phong)	6,961	12,892
Japan (Nagoya)	Hong Kong (Kwai Chung)	1,483	2,747
	Malaysia (Port Klang/Swettenham)	3,000	5,556
	India (Mumbai)	5,225	9,677
	Vietnam (Hai Phong)	2,090	3,871
Germany (Hamburg)	Hong Kong (Kwai Chung)	10,001	18,522
	Malaysia (Port Klang/Swettenham)	8,344	15,453
	India (Mumbai)	6,573	12,173
	Vietnam (Hai Phong)	9,863	18,266
United Kingdom (Felixstowe)	Hong Kong (Kwai Chung)	9,679	17,926
	Malaysia (Port Klang/Swettenham)	8,022	14,857
	India (Mumbai)	6,251	11,577
	Vietnam (Hai Phong)	9,541	17,670
		**Average**	**14,088**

Notes: Estimates of the nautical miles between major plastic waste exporting countries (United States of America, Japan, Germany, and the United Kingdom) and major importing countries (Hong Kong, Malaysia, India and Vietnam) derived using an online nautical distance calculator[47] and converted to kilometres (1 nautical mile: 1.852 kilometres).

Table S7. Calculation of International Plastic Waste Trade distances travelled by ocean freight (2023)

Major Port (Exporting Country)	Major Port (Importing Country)	Nautical miles	Kilometres
Germany (Hamburg)	Netherlands (Rotterdam)	305	565
	Turkey (Haydarpasa, Istanbul)	3414	6323
	United States of America (New York)	3620	6704
	Indonesia (Tanjung Priok, Jakarta)	8803	16303
Japan (Nagoya)	Netherlands (Rotterdam)	11078	20516
	Turkey (Haydarpasa, Istanbul)	8590	15909
	United States of America (Los Angeles)	4988	9238
	Indonesia (Tanjung Priok, Jakarta)	3125	5788
Netherlands (Rotterdam)	Turkey (Haydarpasa, Istanbul)	3161	5854
	United States of America (New York)	3383	6265
	Indonesia (Tanjung Priok, Jakarta)	8550	15835
United States of America (New York)	Netherlands (Rotterdam)	3383	6265
	Turkey (Haydarpasa, Istanbul)	5006	9271
United States of America (Los Angeles)	Indonesia (Tanjung Priok, Jakarta)	7899	14629
		**Average**	**9,962**

Notes: Estimates of the nautical miles between major plastic waste exporting countries (Germany, Japan, Netherlands, United States of America) and major importing countries (Netherlands, Turkey, United States of America, Indonesia) derived using an online nautical distance calculator[47] and converted to kilometres (1 nautical mile: 1.852 kilometres).

2.2.5. Informal Sector Collection and Sorting

Informal collection and sorting were assumed to be carried out by waste pickers in P_2O.[1,2] *Waste picker* is an collective term used to describe as many as 10 to 20 million people worldwide estimated to work in the informal waste sector, including individuals and entrepreneurs, organised groups and co-operatives who carry out a range of activities from the collection of mixed waste, recycling and sorting and waste processing.[48] Almost 60% of global plastic recycling has been estimated to be processed by the informal sector, though documentation of their contributions and operations is very limited.[2,48]

Waste pickers are known to operate in a range of locations including at dumpsites and waste transfer stations, formal waste collection sites, streetsides, and via door-to-door operations[48]. Data on the numbers of people working in different capacities across these locations is limited[48] and there is no representative data on the modes of transportation or equipment used by waste pickers. We therefore modelled this process without material or resource inputs, assuming non-mechanised modes of transport and human energy as the main process inputs, and without any process-specific emissions. This is an important limitation of current LCA methods, as substantial health burdens are likely to be borne by informal waste workers.

2.2.6. Littering and Pollution Removal

We modelled littering and post-collection environmental leakage with no process-specific resources or emissions. The subsequent impacts of this plastic are accounted for in onwards waste treatment and pollution compartments of P_2O[1,2]. Pollution removal was defined by P_2O as organised beach clean ups and other services for removing existing plastic pollution from waterways, the latter with very little contribution to overall system change for managing plastics.[1]

The processes behind these pollution removal approaches are not well documented and may include a variety of different activities. If organised community-based beach clean-ups are assumed, these are predominantly conducted by groups of volunteers, waste-picking by hand, possibly with specific inputs such as waste collections bins, gloves, and metal waste picker apparatus.[49] These activities are similar to the informal sector of waste collection, with minimal material inputs and predominantly powered by human time and energy, for the purpose of this analysis these processes were considered to consume no resources and to generate no emissions. At the other end of the scale, specialised ocean and river clean-up technologies are being developed such as those used by *The Ocean Clean-Up.*[50] These are floating U-shaped devices, powered by boat, or constructed across rivers that catch and collect floating plastics from aquatic environments.[50] These processes have not been accounted for in the current analysis due to the absence of inventory data and minimal contribution to P_2O systemic change, but environmental impact assessments are emerging.[51] It will be important to factor this in to future assessments.

2.2.7. Regionalisation Summary

Only industrial sorting within the Collection, Sorting and Transportation stages was regionalised by electricity mix. We created separate inventories for industrial sorting for each of the P_2O geographical archetypes as this process required electricity inputs. We assigned the existing Ecoinvent geography-specific datasets to P_2O archetypes according to Country Income Classifications[52] whilst maintaining the same overall proportional contributions to global sorting as detailed in section **2.2.3. Industrial Sorting.** We modified the Rest of the World datasets with regionalised electricity mixes for HIC, UMC, LMC and LIC. Further details on regionalisation of inventories are available in **Section 4. Regionalisation: Electricity Mix.**

2.3. Recycling

Recycling comprises separate processes for mechanical recycling and chemical recycling through pyrolysis in the P_2O model.[1,2] Details of the inventories for both types of recycling are provided in this section.

Table S8. Summary of Plastic Mechanical Recycling and Chemical Recycling inventories

Summary of inventory methods	Ecoinvent Inventory Data Source[3]	Primary Data Source*[3]
**Mechanical Recycling: Industrial Recycling Process**		
**SUMMARY APPROACH:** Ecoinvent datasets for recycled polymer production in different geographies combined based on country-level recycling capacity data, combination of polymer specific global average datasets to reflect 1 Mt of P_2O plastic categories<br><br>**SCOPE OF DATASETS:** Process-specific resources and emissions, industrial facility, and wastewater treatment<br><br>**MODIFICATIONS:** Scaled inputs and emissions for recycling treatment rather than production of 1 Mt recycled plastics (losses were accounted for separately by P_2O), removal of waste plastic inputs and waste treatment of losses, regionalisation of datasets by electricity mix	Ecoinvent: HDPE granulate, recycled (*Switzerland*, *Europe without Switzerland*, *Rest of the World, United States of America*)<br><br>Ecoinvent: PET amorphous granulate, recycled (*Switzerland*, *Europe without Switzerland*, *Rest of the World, United States of America*)<br><br>Ecoinvent: PET bottle-grade, recycled (*Switzerland*, *Rest of the World*)	Carbotech (2017)[38]
**Mechanical Recycling: Avoided Secondary Plastic Production**		
**SUMMARY APPROACH:** Virgin Production datasets applied as substituting secondary plastics on a 1:1 ratio. Combination of polymer-specific global datasets to reflect 1 Mt of P_2O plastic categories.<br><br>**SCOPE OF DATASETS:** Same as virgin polymer production<br><br>**MODIFICATIONS:** None	Same as virgin polymer production	Plastics Europe (1999 - 2015)*<br><br>Substitution rate: Klotz and Haupt (2022)[53]
**Chemical Recycling: Pyrolysis**		
**SUMMARY APPROACH:** Creation of dataset based on Somoza-Tornoz et al (2020)[54] data, addition of sorting process and sorting to recycling transportation for equivalence with mechanical recycling process. Applied as a proxy for all polymers in 1 Mt of P_2O plastic categories.<br><br>**SCOPE OF DATASETS:** Process-specific resources and emissions, industrial furnace, and industrial facility<br><br>**MODIFICATIONS:** Regionalisation of datasets by electricity mix	Ecoinvent: High voltage electricity mix; Market for heat, from steam in chemical industry (*Europe*); Market for steel, chromium steel, 18/8 (*Global*); Industrial furnace, natural gas (*Global*); Market for waste preparation facility (*Global*); Carbon dioxide	Somoza-Tornoz et al (2020)[54]
**Chemical Recycling: Polymerisation for granulate production**		
**SUMMARY APPROACH:** Adaptation of Ecoinvent LDPE granulate production without the input of ethylene. Applied as a proxy for all polymers in 1 Mt of P_2O plastic categories.<br><br>**SCOPE OF DATASETS:** Process-specific resources and emissions, industrial facility, and waste treatment<br><br>**MODIFICATIONS:** Removal of ethylene input (product of pyrolysis stage), regionalisation of datasets by electricity mix	Ecoinvent: Market for LDPE granulate production (*Global*)	Plastics Europe (2011)*<br>Same as virgin production
**Chemical Recycling: Avoided Fuel Production**		
**SUMMARY APPROACH:** Ecoinvent datasets based on Somoza-Tornoz et al (2020) pyrolysis outputs. Applied as a proxy for all polymers in 1 Mt of P_2O plastic categories.<br><br>**SCOPE OF DATASETS:** Market geographies of fuels, pipeline transport and construction<br><br>**MODIFICATIONS:** None	Ecoinvent: Market for natural gas, high pressure (*Global*); Market for propylene (*Rest of the World*); Market for benzene (*Global*)	Somoza-Tornoz et al (2020)[54]

Notes: *Details on the Ecoinvent dataset sources derived from Ecoinvent documentation for version 3.8 (cut off by classification)[3].
Abbreviations: Plastics-to-Ocean (P_2O), Million metric tonne (Mt) Low-density polyethylene (LDPE), High-density polyethylene (HDPE), Polypropylene (PP), Polystyrene (PS), Polyvinyl chloride (PVC), Polyethylene terephthalate (PET).

2.3.1. Plastic Mechanical Recycling

Mechanical recycling is the most common form of plastic recycling worldwide and refers to the processing of plastic waste to form secondary raw materials and/or products without altering the chemical structure of the polymer.[55] The process involves the collection and separation of plastics from other waste materials, sorting,

shredding, washing and extrusion to form homogenous pellets that can be supplied for secondary plastic product manufacture.[55]

Ecoinvent contains inventory datasets for the process of mechanical recycling of HDPE and amorphous PET (in the geographies of *Switzerland, Europe without Switzerland, Rest of the World,* and the *United States of America*), and bottle-grade PET (in the geographies of *Switzerland* and *Rest of the World*). These data were based on a commissioned report by Carbotech in 2017; [38] all geographies were extrapolated from the confidential Swiss industry data.[3]

These datasets describe the recycling plant processes needed to produce a unit of recycled polymer, including sorting and cleaning, homogenization, shredding, melting and finally cooling into pellets.[3] To harmonise this process with P_2O flows, we scaled all inputs and emissions to reflect units of waste polymer treated, rather than produced, and removed the inputs of plastic waste and the treatment of material losses. This avoided double counting of elements considered elsewhere in P_2O and provided the inventory for the recycling process alone. We used HDPE datasets as a proxy for LDPE, PP, PVC and PS, and combined geographies to produce a weighted global inventory for each polymer on the basis of plastic recycling capacity data:

In 2022, total annual plastic waste was estimated at 379.31 Mt,[56] with 15% (56.90 Mt) collected for recycling (this represents recycling input, rather than the 9% estimated to be effectively recycled into secondary plastics after accounting for losses)[57]. This would equate to 59.60 Mt plastic collected for recycling globally in 2022. According to a report by Plastics Recyclers Europe, European installed annual plastic recycling capacity was estimated at 9.5 Mt, of which Switzerland accounted for between 0.25 – 0.5 Mt.[58] Different estimates were found for the USA; Greenpeace estimated 2.05 Mt of USA-generated plastic waste were domestically recycled in 2018,[59] though this does not account for imported waste and an interview in the report suggested recycling plants were operating below capacity.[59] To adjust for these factors and for greater consistency with European data, we increased the USA estimate by 40% to 2.87 Mt. The *Rest of the World* estimate was based on the global estimate for plastic recycling minus these data for *Switzerland*, *Europe without Switzerland,* and the *United States of America*.

Therefore, for every 1 Mt of average global plastic recycling: *Switzerland* was estimated to account for 0.88% (0.5 Mt / 56.90 Mt), *Europe without Switzerland* 15.82% ((9.5-0.5) Mt / 56.90 Mt), *United States of America* 5.04% (2.87 Mt / 56.90 Mt) and the *Rest of the World* for the remainder at 78.26% ((9.5-0.5-9.0-2.87) Mt / 56.90 Mt). Polymer-specific datasets were then combined to reflect P_2O plastic categories (**Figure S1**). The losses from the recycling process are accounted for in a specific P_2O flow that describes the quantity of residual plastic collected from recycling plants and subsequently treated in landfill, incineration or mismanaged with the potential for release into the environment.[1,2] As such the rates of process losses are pre-determined by P_2O, and the impacts of transportation and end-of-life treatment or mismanagement are accounted for in the separate LCIs for these processes.

Similarly, the rate of virgin plastic substitution via closed-loop recycling was pre-determined by P_2O flows and was reflected in the reduced rates of virgin plastic input into the system under certain scenarios through substitution with recycled polymer.[1,2] Ecoinvent datasets include the production of recycled polymer pellets as a final product output,[3] which is the equivalent stage of production to virgin polymer granulate. Therefore, no additional steps were required in LCI modelling to reflect closed-loop substitution.

For open-loop recycling, P_2O considers that this plastic leaves the existing system and is converted to lower grade plastic products, substituting virgin plastics or other materials.[1,2] There is extremely limited data available on secondary plastic applications from open-loop recycling; it is not including in Plastics Europe, or Plastic Recyclers Europe, reports.[9,58]. Nevertheless it was necessary to consider 'avoided burdens' of secondary products through open-loop recycling so as not to bias our impact assessment, as P_2O accounts for the avoided burdens of virgin plastic production in closed-loop recycling. A recent Swiss study conducted a material flow analysis of the main plastic types arising in waste in Switzerland, detailing their original application and the uptake of these polymers into secondary applications following recycling.[53] This study assumed a 1:1 substitution ratio of virgin polymers in secondary applications, acknowledging this may not be the case for all polymers,[53] but as demonstrated starting point for our analysis, we adopted the same approach. The inventory for the avoided burdens therefore is an exact duplicate of virgin plastic production inventories detailed in **Section: 2.1.1. Virgin Plastic Production.**

2.3.2. Plastic Chemical Recycling

In contrast to mechanical recycling where the chemical structure of the polymer is maintained, chemical recycling refers to the process of breaking plastic waste polymer chains into shorter hydrocarbon fractions or monomers using chemical, thermal, or catalytic processes.[55] The benefits of chemical recycling can include a reduced need for sorting as mixed plastics can be input into the process, though it requires significant energy and does not yet operate at any substantial scale.[1,2]

P_2O modelled 'chemical conversion' in the form of pyrolysis, further defined as either Plastic to Plastic (P2P) – chemical recycling or Plastic to Fuel (P2F) waste disposal.[1,2] Both processes were applied to very little of the overall global system for plastics: 3-4% of total plastics in the *Recycling* and *System Change* scenarios are processed through chemical conversion by 2040.[1,2] This was only applied in to categories of Flexible Monomaterials and Mult-layers/Multi-materials in urban settings within the HIC, UMC and LMC archetypes, supplied through the mixed waste and informal recycling flows in P_2O.[1,2] To inform the modelling of this process, P_2O estimated installed chemical conversion capacity in 2019 from 28 facilities in HIC (489 kt/year), and 300 P2F facilities in India of 5t/d each, extrapolated by expert opinion for UMC and LMC.[1,2] Projections to 2040 included 2% compound annual growth rate for P2F and no development of P2P for the business-as-usual system scenario.

To match the LCI for the chemical conversion process to P_2O flows, we modelled three distinct phases: (1) pyrolysis, which is the same process for P2P and P2F, (2) polymerisation of monomers to produce granulate in line with virgin production, and (3) the avoided burdens of fuel production:

(1) There is no Ecoinvent dataset available for pyrolysis, so we created a new dataset based on an inventory described in an existing LCA of PE pyrolysis for ethylene production in Europe.[54] These data were derived from a simulation of the process of waste PE pyrolysis in Aspen Plus at an industrial scale, accounting for mass and energy flows and the sizes of the equipment units.[54] The inventory included the process-specific resources and emissions, with the allocation of an industrial furnace and the facility.[54] We scaled the quantities of each to represent the treatment of a unit of waste PE, rather than a unit of ethylene produced in the original publication (**Table S9**). We also added a sorting process and transportation from sorting to recycling for equivalence with modelling of the mechanical recycling process. The same LCI process was applied as a proxy to all polymers and aggregate P_2O plastic categories, but we regionalised the electricity mix according to P_2O geographical archetypes.

Table S9. Pyrolysis process inventory per kilogram of waste plastic

Inputs: Per kg of waste polymer processed		
ECOINVENT: High voltage electricity mix[3]	1.339	kWh
ECOINVENT: Market for heat, from steam in chemical industry (*Europe*)[3]	0.102	kWh
ECOINVENT: Market for steel, chromium steel, 18/8 (*Global*)[3]	4.44E-5	kg
ECOINVENT: Industrial furnace, natural gas (*Global*)[3]	1.11E-10	single unit
ECOINVENT: Market for waste preparation facility (*Global*)[3]	1.73E-9	kg
Emissions to air: Per kg of waste polymer processed		
ECOINVENT: Carbon dioxide[3]	0.454	kg

Notes: The inputs for the inventory of pyrolysis were adapted from a published Life Cycle Assessment (LCA) of pyrolysis-based ethylene production in Europe by Somoza-Tornos et al. (2020).[54] Data presented were adapted from the original study (based on a unit of ethylene produced) to represent a unit of waste polymer processed.

(2) This particular pyrolysis process produced ethylene directly, which can be polymerised to produce PE polymers. P_2O contains a specific flow that describes post-pyrolysis monomer input into total plastic production.[1,2] To estimate the impacts of this, equivalent to the granulate production of virgin material and mechanically recycled polymers, we need to add a polymerisation step in the LCI for this flow. We selected the Ecoinvent dataset for the production of virgin LDPE granulate in the global geography to modify as the most common polymer. We used the datasets for production with no transportation included as this is already accounted for in the collection of waste plastics. We removed the input of ethylene, as this is the product supplied by the chemical conversion process and scaled all polymerisation-specific inputs and emissions to represent the polymerisation of one unit of ethylene, as opposed to the production of one unit of LDPE. In the

absence of polymer specific data, we applied the same LCI for this process to all polymers and P_2O plastic categories but regionalised electricity mix according to geographical archetype.

To account for the avoided burdens of fuel production we used estimates provided in the Somoza-Tornos et al. (2020) publication for the units of methane (0.204 kg/h) propylene (0.378kg/h) and benzene (0.287kg/h) produced per kg of ethylene.[54] We scaled these inputs to represent equivalent proportions of 1 Mt of fuel production substituted and matched Ecoinvent market datasets to include transportation in the global geography where possible, or *Rest of the World* (**Table S10**).

Table S10. Avoided Fuel Production from Pyrolysis inventory

Avoided burdens of 1 Mt fuel production via pyrolysis		
Natural gas, high pressure {*Global*} market group[3]<br>*(converted from kg using Ecoinvent 0.735kg/m3 estimate)*	-31.94E+7	m^3
Propylene {*Rest of the World*}| market[3]	-0.43	Mt
Benzene {*Rest of the World*}| market[3]	-0.33	Mt

Notes: Datasets taken from the Ecoinvent database version 3.8 (cut off by classification)[3] based on data inputs in Somoza-Tornos et al. (2020)[54]

2.3.3. Regionalisation Summary

We created separate inventories for mechanical recycling for each of the P_2O geographical archetypes as this process required electricity inputs. We assigned Ecoinvent geography-specific datasets according to Country Income Classifications[52] whilst maintaining the same overall proportions as detailed in **Section 2.3.1. Plastic Mechanical Recycling.** For chemical recycling, the dataset created for the pyrolysis process was duplicated and modified with regional electricity mixes. The sorting process was regionalised as detailed in **Section 2.2.7. Regionalisation Summary** by redistributing Ecoinvent geographical datasets and modifying electricity mixes for *Rest of the World*. Geography specific datasets for transportation from sorting to chemical recycling were also assigned by Country Income Classification[52] to P_2O archetypes as no international trade was considered in P_2O.[1,2] The polymerisation process was modified with regionalised electricity mixes.

No regionalisation of avoided burdens of secondary plastics from mechanical recycling or from avoided fuel production from pyrolysis was conducted, assuming products are placed on the global market. Further details on regionalisation of mechanical and chemical recycling inventories are available in **Section 4. Regionalisation: Electricity Mix.**

2.4. End-of-Life Waste Management and Mismanagement

Waste management includes industrial incineration and sanitary landfill in P_2O whilst mismanagement refers to various forms of disposal that can lead to environmental leakage of macroplastics including open burning and open dumpsites, and the environmental pollution of macroplastics on land and in waterways. A summary of inventories for these processes is provided in **Table S11** and further details are available in the subsequent sections of the Supplementary Material.[1,2]

Table S11. Summary of Waste Management and Mismanagement inventories

Summary of inventory methods	Ecoinvent Inventory Data Source[3]	Primary Data Source*[3]
**Industrial Incineration**		
**SUMMARY APPROACH:** Combination of polymer-specific global datasets to reflect 1 Mt of P_2O plastic categories<br><br>**SCOPE OF DATASETS:** Process-specific resources and emissions, industrial facility, and waste treatment<br><br>**MODIFICATIONS:** None	Ecoinvent: Waste PE, PP, PS, PVC, PET municipal incineration (GLO)	Doka Life Cycle Assessments (2003)[60]: Waste-specific analysis of Swiss literature data (1994) for *Switzerland*, extrapolated for *Rest of the World*[3]
**Sanitary Landfill**		
**SUMMARY APPROACH:** Combination of polymer-specific global datasets to reflect 1 Mt of P_2O plastic categories	Ecoinvent: Waste PE, PP, PS, PVC, PET, sanitary landfill (GLO)	Doka Life Cycle Assessments (2003)[60]: Waste-specific analysis of

Summary of inventory methods	Ecoinvent Inventory Data Source[3]	Primary Data Source*[3]
**SCOPE OF DATASETS:** Process-specific resources and emissions, landfill site, and waste treatment<br><br>**MODIFICATIONS:** Regionalisation of datasets using geographical archetype electricity mixes		Swiss literature data (1994) for *Switzerland*, extrapolated for *Rest of the World*[3]
**Open Burning**		
**SUMMARY APPROACH:** Combination of polymer-specific global datasets to reflect 1 Mt of P_2O plastic categories<br><br>**SCOPE OF DATASETS:** Process specific emissions to air and soil and oxygen consumption<br><br>**MODIFICATIONS:** None	Ecoinvent: Waste PE, PP, PS, PVC, PET open burning (GLO)	Ecoinvent (2023)[3]: Secondary data based on generic municipal waste incineration without any pollution control
**Open Dumpsites/Unsanitary Landfill**		
**SUMMARY APPROACH:** Regionalised geographical datasets to reflect P_2O geographical archetypes, estimating proportion of open dumps and unsanitary landfill, and proportions of infiltration class weighted by country-level data taken from Doka et al. (2018)[61] Combination of polymer specific regionalised datasets to reflect 1 Mt of P_2O plastic categories for each geographical archetype<br><br>**SCOPE OF DATASETS:** Waste specific short-term and long term emissions to air, water and soil<br><br>**MODIFICATIONS:** Geographic regionalisation by national infiltration classes and proportion of waste in open dumpsites versus unsanitary landfill	Ecoinvent: Waste PE/PP/PS/PVC/PET, open dump, hyperarid/dry/moist/wet/very wet infiltration (*Global*)<br><br>Ecoinvent: Waste PE/PP/PS/PVC/PET, unsanitary landfill, hyperarid/dry/moist/wet/very wet infiltration (*Global*)	Doka Life Cycle Assessments (2018)[61]
**Terrestrial Pollution**		
**SUMMARY APPROACH:** Modification of available open dump datasets in Ecoinvent. Regionalised geographical datasets to reflect P_2O geographical archetypes, estimating proportions of infiltration class weighted by country-level data taken from Doka et al. (2018)[61]. Modified degradation rate and combination of polymer-specific regionalised datasets to reflect 1 Mt of P_2O plastic categories for each geographical archetype<br><br>**SCOPE OF DATASETS:** Waste specific short-term and long-term emissions to air, water, and soil<br><br>**MODIFICATIONS:** Removal of land use and heat emissions to soil, acceleration of emissions to all compartments, regionalisation by national infiltration classes	Ecoinvent: Waste PE/PP/PS/PVC/PET, open dump, hyperarid/dry/moist/wet/very wet infiltration (*Global*)	Doka Life Cycle Assessments (2018)[61]
**Aquatic Pollution**		
**SUMMARY APPROACH:** Modification of available open dump datasets in Ecoinvent. Modified degradation rate of existing Ecoinvent open dump datasets and combined polymer-specific datasets to reflect 1 Mt of P_2O plastic categories.<br><br>**SCOPE OF DATASETS:** Waste specific short-term and long-term emissions to air, water, and soil<br><br>**MODIFICATIONS:** Removal of land use and heat emissions to soil, acceleration of emissions to water, and change of emission compartment from groundwater to river water	Ecoinvent: Waste PE/PP/PS/PVC/PET, open dump, very wet infiltration (*Global*)	Doka Life Cycle Assessments (2018)[61]

Notes: *Details on the Ecoinvent dataset sources derived from Ecoinvent documentation[3]. Abbreviations: Plastics-to-Ocean (P_2O), Million metric tonne (Mt) Low-density polyethylene (LDPE), High-density polyethylene (HDPE), Polypropylene (PP), Polystyrene (PS), Polyvinyl chloride (PVC), Polyethylene terephthalate (PET).

2.4.1. Managed Waste: Industrial Incineration and Engineered Landfill

Ecoinvent holds inventory datasets for industrial incineration and sanitary landfill of polymers.[3] The datasets include the quantities of process-specific resources consumed and emissions generated (including both short- and long-term emissions from landfill), the allocated burdens of land use and waste treatment facility construction and decommissioning, and subsequent treatment of residual materials, for example the landfilling of incineration residue and the leachate treatment from engineered landfills.[3]

Datasets are available for both incineration and landfill in the geographies of *Switzerland* and *Rest of the World*, for each polymer including PE (which we used as a proxy for both LDPE and HDPE), PP, PS, PVC, and PET (which we used as a proxy for both bottle grade and amorphous PET).[3] These datasets are the product of modelling commissioned by Ecoinvent and are based on literature informed waste-specific calculations for *Switzerland* and modification of inputs to reflect the *Rest of the World* geography.[60] Data were produced in 1994 and published in 2003.[60]

For each polymer we created a geographically weighted global average inventory dataset, based on national contribution to plastic waste generation,[41,44] to reflect 1 Mt of polymer incinerated or landfilled at the global scale. This included 0.004085127 Mt of the *Switzerland* dataset and 0.995914873 Mt of the *Rest of the World* dataset for each polymer. These weighted global average datasets for each polymer were then proportionally combined to represent each P_2O plastic category.

The resulting datasets for landfill for each P_2O plastic category were subsequently regionalised according to electricity mixes For HIC, UMC, LMC and LIC. Incineration did not have electricity as a process input therefore the same global average inventory was applied to all geographical archetypes. Avoided burdens are not included for the production of electricity from incineration, nor the production of heat from landfill.

2.4.2. Open Dumpsites and Unsanitary Landfill

Open dumpsites and unsanitary landfill are modelled as a single compartment in P_2O.[1,2] This end-of-life fate category is based on the International Solid Waste Association definition of dumpsites: "a land disposal site where the indiscriminate deposit of solid waste takes place with either no, or at best very limited, measures to control the operation and protect the surrounding environment."[62] P_2O extended this definition to include unsanitary landfills, considered to be landfill sites without daily and intermediate covers to prevent waste leaking to the environment.[1,2]

In Ecoinvent, separate inventory datasets are available for open dumpsites and for unsanitary landfill, for each polymer including PE (which we used as a proxy for both LDPE and HDPE), PP, PS, PVC, and PET (which we used as a proxy for both bottle grade and amorphous PET).[3] Datasets include the occupation of arable land in m^2, in the case of landfill the machinery and fuel required for excavating the land, and for both the emissions to air, soil and water.[3]

Rather than being geographically differentiated by country, Ecoinvent datasets for both dumpsites and unsanitary landfill are sub-divided by five categories of infiltration class which reflects regional differences in climatic conditions. These datasets were the result of modelling by Doka (2018)[61] commissioned by Ecoinvent; the climate-sensitivity was included because differences in rainfall affect water infiltrating the dump or landfill which influences the speed of weathering and emissions from waste content.[61] The Doka model calculated a mean infiltration class for 154 countries based on grid point precipitation minus evapotranspiration, weighted by population density to account for greater landfill and dumpsites close to population dense zones and fewer in more rural, sparsely populated areas.[61] The Doka model also included the proportion of waste sent to open dumpsites and unsanitary landfill for 218 countries, based on data from 2019.[61]

We cross-referenced the country-level data on infiltration classes and the proportion of open dumpsite to unsanitary landfill fate in the Doka model[61] with data on national-level contribution to global plastic waste generation[41,44] and organised countries by income classification based on World Bank data[52] to mirror the P_2O geographical archetypes.[1,2] Combining these data sources, we created regionalised datasets for each of the P_2O plastic categories **(Table S12),** in HIC, UMC, LMC and LIC, weighted by national differences in proportion of open dumpsites and unsanitary landfill, and the weighted mean infiltration class.

Table S12. Geographic weighting for Open Dumpsite/Unsanitary Landfills

Country Income Classification	Infiltration Class (same proportions of infiltration class apply to open dumpsites and to unsanitary landfill)	(%)	End of Life Fate	(%)
HIC	Infiltration Class 1: Hyperarid	2.23%	Open Dumpsite	7.51%
	Infiltration Class 2: Dry	2.47%		
	Infiltration Class 3: Moist	28.31%		
	Infiltration Class 4: Wet	58.65%	Unsanitary Landfill	92.49%
	Infiltration Class 5: Very Wet	8.34%		
UMC	Infiltration Class 1: Hyperarid	2.43%	Open Dumpsite	32.17%
	Infiltration Class 2: Dry	23.01%		
	Infiltration Class 3: Moist	11.51%		
	Infiltration Class 4: Wet	62.73%	Unsanitary Landfill	67.83%
	Infiltration Class 5: Very Wet	0.32%		
LMC	Infiltration Class 1: Hyperarid	10.19%	Open Dumpsite	58.65%
	Infiltration Class 2: Dry	10.96%		
	Infiltration Class 3: Moist	5.16%		
	Infiltration Class 4: Wet	52.44%	Unsanitary Landfill	41.35%
	Infiltration Class 5: Very Wet	21.25%		
LIC	Infiltration Class 1: Hyperarid	23.21%	Open Dumpsite	81.41%
	Infiltration Class 2: Dry	23.50%		
	Infiltration Class 3: Moist	41.14%		
	Infiltration Class 4: Wet	9.40%	Unsanitary Landfill	18.59%
	Infiltration Class 5: Very Wet	2.75%		

Notes: National proportions of unsanitary landfill versus open dumpsites, and on average infiltration class, derived from Doka et al. (2018)[61], grouped and weighted according to World Bank Country Income Classifications[52]

2.4.3. Open Burning

Open burning of waste is common in many countries with insufficient waste management facilities and can occur at dumpsites, businesses, and individual residences. It is a major source of underestimated pollution, residential waste burning alone may account for more than a fifth of China's PM10 air pollution.[63] P$_2$O estimates of the quantity of plastic open burned are based on both dumpsites and residential burning.[1,2]

We used available Ecoinvent polymer-specific global (generic) inventories for open burning (no geographically disaggregated data available).[3] These data were generated in 2006 and updated in 2023 and are based on industrial incineration datasets with the pollution control removed.[3] The datasets are available for PE (which we used as a proxy for both LDPE and HDPE), PP, PS, PVC, and PET (which we used as a proxy for both bottle grade and amorphous PET) and reflect oxygen consumption for burning and emissions to air and soil. There is no allocation of land use or any other inputs.[3] Polymer specific datasets were combined proportionally according to the P$_2$O plastic categories to create 1 Mt of each polymer mix open burned.

Open burning estimates were likely conservative; temperature ranges in uncontrolled fires are substantially lower than industrial incineration, resulting in greater particulate emissions.[64] Furthermore, generalised inventories do not reflect differences in plastics form (e.g. hard or soft), heterogenous waste compositions, combustion characteristics and the varying temperatures of uncontrolled fires, which strongly influences the quantity of different emissions produced.[65] It was unclear whether black carbon was included in emissions data on particulates,[3] and its contribution to climate change is not characterised in ReCiPe 2016.[5] Black carbon is produced through incomplete combustion of fossil-plastics; it is particularly damaging for respiratory health and climate, with possibly 900 times the global warming potential of carbon dioxide (100-year time horizon).[64]

2.4.4. Terrestrial Pollution

Terrestrial pollution accounts for macroplastic leaked into the natural land-based environment in P$_2$O.[1,2] The compiled inventory represents the environmental degradation of plastic and the subsequent release of its chemical components over the long term. There are no pollution specific datasets available in Ecoinvent, therefore open dumpsite datasets were modified as proxies.[3] Similarly to the Open Dumpsite and Unsanitary Landfill inventory compiling, we used the country-level data on infiltration classes available in the Doka model[61] with data on national-level contribution to global plastic waste generation[41,44] and organised countries

by income classification[52] to create regionalised, weighted average datasets for each of the P2O plastic categories in HIC, UMC, LMC and LIC. This was based on the assumption that the differences in climatic conditions affect chemical releases from terrestrial macroplastic pollution in a similar way to macroplastics in open dumpsites. However, we considered that as pollution, macroplastics are more exposed to sunlight and heat which would likely increase the speed of degradation compared to those potentially partially buried in dumpsites.[66]

We searched published literature and identified an overview of thermoplastic polymers that compared plastic degradation rates under different environmental conditions including (1) buried in land, (2) in land accelerated by ultraviolet sunlight exposure and heat, (3) marine environment, (4) marine environment accelerated by ultraviolet sunlight exposure and heat.[66] We calculated the average degradation rate ratio across polymers between plastics buried in land and on land accelerated by UV/heat **(Table S13).**[66] We found that on average plastics degraded 1.6 times faster when exposed to UV and heat. This figure was used to modify Ecoinvent open dumpsite datasets for faster rate of near-term emissions.

Open dumpsite datasets include emissions to water in the near term and in the long term for each chemical.[3] We multiplied near term emission quantities by 1.6 to reflect the faster degradation rate and subtracted this quantity from the long term to maintain the same overall emission quantity. This reflects an assumption that the polymer degradation rate is equal to the rate of release of its chemical components to the environment. In fact, this is likely to underestimate chemical release as the greater the surface area as a plastic fragments, the faster the release of its chemical contents may be.

Table S13. Comparison of polymer degradation rates under different environmental conditions[66]

Plastic	Estimated Specific Surface Degradation Rate and Comparative Environmental Degradation Rates						
	**Land (buried)**	**Land (accelerated by UV/heat)**	**Rate Ratio (Land (accelerated): Land (buried)**	**Marine**	**Rate Ratio (Marine: Land (buried)**	**Marine (accelerated by UV/heat)**	**Rate Ratio (Marine (accelerated): Land (buried)**
	(mm/yr)	*(mm/yr)*	*Ratio*	*(mm/yr)*	*Ratio*	*(mm/yr)*	*Ratio*
HDPE	1.0	1.3	1.3	4.3	4.3	9.5	9.5
LDPE	11	22	2	15	1.4	10	0.9
PET	0*	-	-	-	-	110	111
PP	- (assumed 0)	0.51	1.51	7.5	8.5	4.6	5.6
Average Rate Ratio	n/a	n/a	1.6	n/a	5	n/a	32

Notes: *Refers to studies finding no degradation occurred though it was likely that study duration was too short to observe the degradation that would occur over a longer period of time.[66]

2.4.5. Aquatic Pollution

Aquatic pollution accounts for macroplastic leaked into the natural aquatic environments including rivers and ocean compartments in P2O.[1,2] There are no pollution datasets available in Ecoinvent so the Open Dumpsite 'very wet' infiltration class datasets[3] were used as the closest proxies for aquatic pollution for each polymer. Based on the literature review[66] identified for Terrestrial Pollution, we calculated the average rate ratio that compared plastic in the marine environment (5 times faster degradation), and marine environment exposed to UV and heat (32 times faster degradation), with plastic buried in land **(Table S13).** Based on existing literature that suggests 1% of marine plastic floats on the surface whilst the rest sinks,[67] we created a weighted rate ratio to reflect this (99% exposed to marine environment and 1% exposed to marine environment accelerated by UV and heat is equal to a weighted average rate ratio of 5.27 relative to plastic buried in land). The Ecoinvent near term emissions were multiplied by 5.27 to reflect the faster rate of degradation and long-term emission reduced to maintain the total quantity of each chemical emission. Similarly to the Terrestrial Pollution proxies, this method is likely to underestimate chemical emissions from macroplastic pollution but offered a starting point for analyses in the absence of more data.

3.4.6. Regionalisation Summary

We modified the inventories for sanitary landfill for each of the P2O geographical archetypes as this process required electricity inputs. Whilst maintaining Ecoinvent assumptions for overall global proportions of *Switzerland* and *Rest of the World* datasets, we assigned the *Switzerland* and a modified *Rest of the World* (substituting HIC electricity mix) to the HIC inventory and *Rest of the World* for all other archetypes, modified with the corresponding electricity mix for UMC, LMC and LIC.

Open Dumpsites and Unsanitary Landfill was regionalised based on national-level proportions of each end-of-life fate and on climatic conditions based on infiltration class, terrestrial pollution was regionalised based on infiltration class as detailed in **Sections 2.4.2. Open Dumpsites and Unsanitary Landfill** and **2.4.4. Terrestrial Pollution**. These processes did not require electricity and were therefore not modified by any electricity mix. Further details on regionalisation of inventories are available in **Section 4. Regionalisation: Electricity Mix.**

3. Inventory Data: Reduce and Substitute Options

Reduce and substitute is a category of policy levers modelled by P2O to reduce pollution.[1,2] The 'reduce' lever models the elimination of 'avoidable' plastics (as perceived by the P2O expert panel) from the system, with no material replacements. The 'substitute' component models the replacement of single-use plastics with another material that provides the same functional service, including three categories of single-use material substitutes and two reusable systems. These levers form the basis of the Reuse and Substitute system scenario in P2O, and part of the *System Change* scenario.[1,2] P2O provides data on the quantity of plastic eliminated, and the quantity substituted by each of the material replacements under each of the system scenarios, by geographical archetype and by year.[1,2] P2O does not model the substitute systems themselves, therefore we designed substitute systems, introducing our own life cycle assumptions, in order to conduct the LCA for these components.

3.1. Reduce Overview

Under the current commitments scenario, P2O modelled the potential reduction in plastic from industry commitments taken from the New Plastics Economy Global Commitments[68] and in line with 89 identified national and regional level government policies including bans and levies passed into legislation but not implemented at the time, identified through the United Nations Single Use plastic report[69] and the European Union Single-Use Plastics Directive.[70] Bans were modelled as being 100% effective where levies were considered to be 69% effective.[1,2]

The maximally foreseen elimination of plastics included in the *Reduce and Substitute* and *System Change* scenarios was based on expert stakeholder engagement.[1,2] On a product-by-product basis, P2O assessed plastic reduction potential and key limiting factors according to geographic archetypes, using a scoring framework to capture perceived technology readiness level, performance, convenience, and cost. Authors reported this assessment as being conservative with potential to change over time and by region.[1,2] The overall reduction in plastic demand is provided for each system scenario, by geographical archetype and by year in the P2O model.[1,2] We did not conduct an LCA for the quantity of plastic reduced in the system, as this refers only to the elimination of mass, with no material replacement.

3.2. Substitute Overview

P2O determined the quantity of plastic that could be substituted through alternative materials and reuse systems using the same expert stakeholder engagement process as for plastics elimination. Product categories were scored according to perceived technology readiness level, performance, convenience, and cost, with time-based limits set to reflect market penetration lags.[1,2]

We created LCI inventories based on the three categories of single-use material substitutes described in P2O (paper, coated paper and compostables) and for the reuse system substitutions (Consumer Reuse and New Delivery Models) (**Table S14**).[1,2] The life cycle stages included in the system boundaries for each material were designed to mirror the main plastic system, so that the impacts of substitution could be compared. For all substitutes, we designed two life cycle scenarios to examine the influence of certain assumptions on life cycle impacts. We selected the option with the lowest impacts to include in combined analyses with the P2O model in order to explore greater potential differences with the P2O *Business-as-Usual* scenario.

Table S14. Summary of life cycle inventories for single-use material substitutes (Paper, Coated Paper and Compostables) and reusable systems (Consumer Reuse and New Delivery Models)

Summary of inventory methods	Ecoinvent Inventory Data Source[3]	Primary Data Source*[3]
**Paper And Coated Paper Substitution**		
**SUMMARY APPROACH:** Equivalent life cycle inventory for substituting 1 Mt of plastics with (1) single-use paper and (2) single-use coated paper (0.03% LDPE/HDPE coating) on a 1:1 mass basis<br><br>**Scenario 1: 100% virgin pulp production and 100% market disposal**<br>**Scenario 2: 75% recycled pulp production, 71% recycling collection rate and 29% market waste disposal**<br><br>The paper component of coated paper substitutes was modelled identically to the paper system, scaled to include 0.03% virgin LDPE/HDPE with market disposal for the plastic lining<br><br>Geographic datasets for pulp production combined based on production data,[71] maximum substitution of virgin pulp with recycled pulp (75%) estimated from JRC guidance[72], geographical datasets for paper waste treatment combined based on national paper waste production[7]<br><br>**SCOPE OF DATASETS:** Production process-specific resources, emissions and waste treatment, industrial facility and land transformation, onwards transport from pulp production, transport of source separated paper and delivery to sorting facility, waste paper sorting process-specific resources and emissions, sorting facility, machinery and waste treatment, transport from sorting to recycling process, recycling process-specific resources and emissions, recycling facility and waste treatment, other waste disposal process-specific resources and emissions, industrial facilities, and final waste and residual treatment[3]<br><br>**MODIFICATIONS**: Regionalisation of recycled pulp production and waste paper sorting datasets using geographical archetype electricity mixes, market waste disposal regionalised by geography	Virgin Pulp:<br>Ecoinvent: Market for sulphate pulp, unbleached (*Europe*)<br>Ecoinvent: Market for sulphate pulp, unbleached (*Rest of the World*)	European Pulp Industry Sector Association AISBL (EPIS) (2020)[73] extrapolated for *Rest of the World*[3]
	Recycled pulp:<br>Ecoinvent: Market for deinked pulp, wet lap (*Global*)<br>Ecoinvent: Market for sorted waste paper (*Global*)	Industry Data (Quebec, confidential) extrapolated for *Rest of the World*[3]
	Waste treatment:<br>Ecoinvent: Market for waste graphical paper (Multiple geographies)	Eurostat and ECOINVENT (2018)[3,37]
**Compostables Substitution**		
**SUMMARY APPROACH**: Equivalent life cycle inventory for substituting 1 Mt of plastics with compostable material polylactide (PLA) on a 1:1 mass basis<br><br>Scenario 1: 100% virgin granulate production and 100% incineration<br>Scenario 2: 100% virgin granulate production and 100% composting<br><br>Global dataset for polylactide (PLA) granulate production, Mixed Waste Collection from main plastic system applied, and modification of datasets on industrial incineration of PE and industrial composting of biowaste to reflect PLA composition using JRC methods guidance and case studies<br><br>**SCOPE OF DATASETS:** PLA production-process specific resources, emissions and waste treatment, industrial facility and land transformation, onwards transport from granulate production*, transportation modes and distances for the collection of mixed waste*, incineration and composting process-specific resources and emissions, industrial facility and waste treatments<br>* All transport inputs include transportation modes and distances, infrastructure and vehicle construction and maintenance, fuel production, emissions, waste treatment<br><br>**MODIFICATIONS:** Industrial incineration: removal of emissions to air and water of all metals as not part of PLA composition: Aluminium, antimony, arsenic, barium, beryllium, cadmium, calcium, chromium, cobalt, copper,, iron, lead, magnesium, manganese, mercury, nickel, sodium, strontium, thallium, tin, titanium, vanadium, zinc. Replaced fossil carbon dioxide with biogenic carbon dioxide. Industrial composting: PLA-specific emissions estimated and replaced in biowaste dataset.<br><br>Regionalisation of incineration datasets by geography only	Ecoinvent: Market for Polylactide granulate (*Global*)	Industry data: NatureWorks (2006) generalised for *Global*[3]
	Ecoinvent: Market for waste plastic, mixture (multiple geographies) (only transport assumptions)	Eurostat and ECOINVENT (2018)[3,37]
	Ecoinvent: Waste polyethylene treatment, municipal incineration (*Switzerland* and *Rest of the World*)	Doka Life Cycle Assessments (2003)[60] modified for *Rest of the World*[3]

Summary of inventory methods	Ecoinvent Inventory Data Source[3]	Primary Data Source*[3]
**CONSUMER REUSE and NEW DELIVERY MODELS**		
**SUMMARY APPROACH:** Equivalent life cycle inventory for substituting 1 Mt of plastics with reusable glass container system, estimated on the basis of the functional unit of providing equivalent packaging service for 1l/1kg of food or drink over 1 year<br><br>Material mass substitution based on existing LCA reviews and weighted according to P_2O product type. Ecoinvent data used for glass production, recycling and collection rate estimates, and market disposal, weighted according to national contribution to global glass waste. Containers assumed to be washed at home (Consumer Reuse), every day for bottles and once per week for food containers (Consumer Reuse) or transported and washed on a weekly rotation (New Delivery Models. Resources used for washing based on published literature.<br><br>**SCOPE OF DATASETS:** Glass production-process specific resources, emissions and waste treatment, industrial production facility, onwards transport to consuming industry, collection transportation including lorry production, road infrastructure and maintenance*, washing process-specific resources, emissions, and dishwasher production, end-of-life collection, sorting process-specific resources and emissions, sorting facility construction, onwards transportation and waste treatments, non-recycled glass market glass disposal transport and processes for various geographies<br><br>*New delivery models only: no collection transportation modelled for Consumer Reuse. All transport inputs include transportation modes and distances, infrastructure and vehicle construction and maintenance, fuel production, emissions, waste treatment<br><br>**MODIFICATIONS:** Removal of the Swiss glass production from globally aggregated datasets as it was a 'black box' dataset with no information on recycled inputs<br><br>Regionalisation of washing datasets using geographical archetype electricity mixes and by proportion of dishwashing versus handwashing in the Consumer Reuse case, regionalisation of market waste disposal by geography	Glass Production:<br>Ecoinvent: Market for white packaging glass (*Global*)	(Multiple published literature sources) (2012)[3]
	Washing:<br>Ecoinvent: Market for dishwasher (*Global*) (not for handwashing)<br>Ecoinvent: Market for tap water (*Global*)<br>Ecoinvent: Market for electricity (*Global*) (regionalised)<br>Ecoinvent: Market for soap (*Global*)	Ecoinvent:<br>(Published literature) (1992 – 2015)[3]
	Transport*:<br>Ecoinvent: Market group for freight lorry transport (*Global*)<br><br>*New delivery models only	(Multiple published literature sources) (2012)[3]
	Waste treatment:<br>Ecoinvent: Market for waste glass (Multiple geographies)	Eurostat and Ecoinvent:<br>(2018)[3,37]

Notes: *Details on the Ecoinvent dataset sources derived from Ecoinvent documentation[3]. Abbreviations: Plastics-to-Ocean (P_2O), Million metric tonne (Mt) Polylactide (PLA), Joint Research Centre (JRC).

In the primary assessment of P_2O system scenarios, we assumed a 1:1 mass substitution ratio for all single-use substitute materials. There is a paucity of data for product-specific substitution ratios and significant variation in available estimates. Based on the products assumed to be substitutable by each material in the P_2O model **(Table S15)**, we searched published literature for product-specific substitution ratios **(Table S16)**. We subsequently conducted a sensitivity analysis using the upper and lower ratios identified to define a range of possible mass substitution ratios for paper-based substitutes and for PLA alternatives. The results of this sensitivity analysis for the year 2040 are provided in **Table S36.**

Table S15. Plastic product applications perceived as being substitutable by paper, coated paper and compostables in the Plastics-to-Ocean model[1]

Product Application	Paper	Coated Paper	Compos tables
Straws and stirrers	x		x
On premise food service disposables			x
Off premise plastic cups		x	
Off premise lids		x	
Off premise containers and clamshells	x		x
Off premise cutlery	x		x
Fresh fruit/vegetable tray/pot/punnet/tub	x		
Pots for liquids and creams, yoghurt, butter, spreads, chocolate, sweets, cream, chilled pot desserts and ice creampots/tubs		x	

Meat tray		x	
Ready meal trays/instant pot snacks	x	x	
Other		x	
Consumer goods, EPS pacakgign, plastic egg boxes, blister packs, clothes hangers, caps and lids	x		x
Carrier bags	x		x
Pouches, trash bags, wraps, 6-rings, netting and other flexibles	x	x	x
B2B shipping sacks, strapping, flexible intermediate bulk containers, bulk liners and rolls			x
Sachets	x	x	x
Multilayer flexibles	x	x	x
Plastic component of laminated aluminium (e.g. toothpaste, and cosmetic tubes), and of carton, paper and aseptic cartons with >5% plastic coating, i.e. incompatible with paper recycling streams			x
Sanitary items			x
Wet wipes	x		x
Cotton bud sticks	x		
Diapers			x

Notes: Reproduced from Lau et al. (2020)

Table S16. Substitution ratios of petrochemical plastics by paper and bio-based polymers as identified in published literature

Product application	Petro-chemical plastic (g)	Bio-based plastic (g)	Mass ratio	Notes
**BIO-BASED POLYMERS**				
Beverage Bottles **0.5 L**	**10g** (PET)	**10g** (bio-based PET)	**1**	Reference: European Commission 2019[74] Notes: Biobased PET has the same mass by product application since it is chemically identical to PET (JRC note ongoing efforts to lightweight bottles which have seen the mass of PET bottles half since 2000 from around 18.9g)
Single use beverage cups	**3.88g** (PP) **5.51g** (PET)	**4.59g** (PLA)	**0.8-1.2**	Reference: European Commission 2019[74] Notes: Based on the information obtained from a small market survey and interviewing some producers
**Cutlery:** Spoon Fork Knife	**-** **5.1g** **4.1g** **4.4g** (PS)	**-** **3.8g** **3g** **3.3g** (PLA)	**1.4**	Reference: European Commission 2019[74] Notes: Assumed to be roughly 35% heavier based on confidential communications with industry
Carrier bag	**17.9g** **(**LDPE)	**22.4g** Bio-based starch polymer	**1.3**	Reference: European Commission 2019[74] Notes: Assumed to be 25% heavier for the same carrying capacity, based on personal communication with industry
Food packaging films	**100g** (PP)	**100g** (PLA)	**1**	Reference: European Commission 2019[74] Notes: Assumed the same weight for the same product applications. "Since the application is still a niche market, it is hard to identify a dominant design. We found contradictory arguments and designs for the same application. Based on a small market survey conducted in this study, we did not find sufficient evidence for the PLA film packaging products which fit in line with the theoretical calculations based on the material substitution factors. Some film packaging companies (who produce film grade specifically for fresh vegetables) do offer PLA films with the same thickness as PP films for the same applications (Bi-AX, 2018). Moreover, via personal communication with the industrial experts, we understood that it is also possible to have the same area density (in m2/kg) for both PLA and PP films for this application. Since PLA has a higher density than PP this results in a thinner PLA film than a PP film."
**PAPER**				
Carrier bag	**6g** HDPE	**12.3g** Paper	**2.1**	Reference: CE Delft 2020[75]
Takeaway bag	**6** HDPE	**31** Paper	**5**	Reference: CE Delft 2020[75]
Shopping bags	**24.2g** LDPE **30g**	**44.7g** Paper	**1.5-1.8**	Reference: Danish EPA 2018[76]

	LDPE recycled rigid handle		
Shopping bags		**5-9**	National Council for Air and Stream Improvement (NCASI) 2020[77]
Beverage containers		**0.5-1.5**	National Council for Air and Stream Improvement (NCASI) 2020[77]
Cups and Plates		**1-4.1**	National Council for Air and Stream Improvement (NCASI) 2020[77]
Food packaging		**0.4-2.1**	National Council for Air and Stream Improvement (NCASI) 2020[77]

3.3. Plastic Substitutes: Single-Use Paper and Coated Paper

For both uncoated and coated paper, we created two separate scenarios for the paper life cycle. The first scenario includes the market for 100% virgin paper production, waste collection and zero recycling, with 100% market disposal, which is based on geography-specific data for the proportions of paper waste sent to landfill (sanitary and unsanitary), incineration, open dumps, and open burning.[3] The second scenario is based on the same production processes but includes 75% recycled pulp production, with 71% waste paper collection rate for recycling in a closed-loop system, with the remaining 29% disposed of through market end-of-life. The system boundaries are depicted in (**Figure S3**).

The coated paper system is the same as the pure paper system, but the paper processes were scaled down to 99.7% of every 1 Mt, to include 0.3% PE polymer laminate assumed by P2O[1]. We estimated that this laminate was made from 63% LDPE and 37% HDPE based on the polymer matching used for laminates in the main plastic system (**Table S3**). The life cycle included 100% virgin polymer production and market disposal for mixed plastic waste.

3.3.1. System Boundaries

We created two illustrative life cycle scenarios for Paper and Coated Paper that included: (1) 100% virgin pulp production and 100% market disposal and (2) 75% recycled pulp production. The system boundaries for these comparative scenarios are shown in **Figure S3**.

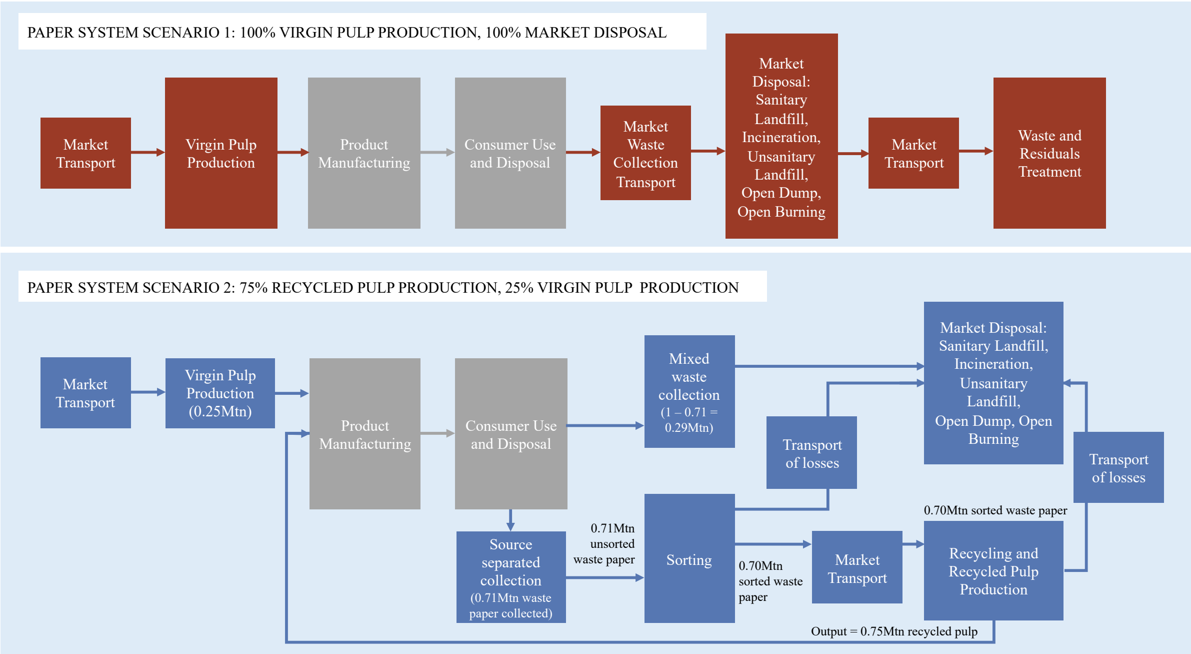

Figure S3. System boundaries for cradle-to-grave life cycle scenarios for Paper and Coated Paper
Cradle-to-grave assessment based on: Scenario 1: 100% virgin pulp production, post-consumer collection and transport and 100% market disposal with no recycling, Scenario 2: 25% virgin pulp production, 75% recycled pulp production, post-consumer collection and transport and 29% market paper disposal. Product

manufacturing and consumer use (in grey) are considered outside of the system boundaries for equivalence with the main plastic system in the Plastics-to-Ocean (P_2O) model.[1,2] *The same system boundaries were applied for Coated Paper, with paper inputs scaled to reflect 99.7% of every unit, and 0.3% virgin polyethylene laminate coating with market disposal.*

3.3.2. Pulp and Laminate Production

Pulp produced as an intermediate step to producing paper is the equivalent to polymer granulate used to produce plastic. For the virgin pulp production inventories of paper and coated paper substitutes we selected Ecoinvent data for unbleached sulphate pulp production available for the geographies of *Europe* and *Rest of the World*.[3] These data are derived from industry data provided in 2020 by the European Pulp Industry Sector Association AISBL (EPIS)[73] as a weighted average of the participating mills located in Europe.[3] The technology described is considered state of the art for Europe.[3] The *Rest of the World* dataset is a weighted average of these European data combined with additional information from Latin America.[3]

Virgin pulp production datasets include production process-specific resources, emissions and waste treatment, the pulp production industrial facility and land transformation, and onwards transport from pulp production.[3] Transport inputs include transportation modes and distances, infrastructure and vehicle construction and maintenance, fuel production, emissions, waste treatment. We weighted geographical datasets according to a report by the Confederation of European Paper Industries (Cepi) which states that 25.6% of total global pulp is produced within *Europe*,[71] and we therefore estimated the remainder as produced in the *Rest of the World* (74.4%).

3.3.3. Paper Recycling

The maximum feasible substitution of virgin pulp with recycled pulp (75%) was estimated from a JRC Technical Report[78] and the European Commission Environmental Footprint reference packages dataset[79], which reflects the recycling output rate for Europe of paper packaging[79].

The only recycled pulp dataset available in Ecoinvent is the global market for *Deinked pulp, wet lap* production in the geographies of *Quebec* and *Rest of the World*[3]. A JRC report on prospective scenarios for the pulp and paper industry, confirmed the widespread use of recycled pulp in all paper and board products, with the only exception of high-grade paper.[80] The inventory for the production process of deinked pulp contains the input of paper waste required to produce a unit of recycled pulp (0.93 kg waste per kg pulp produced).[3] This quantity was used to link the inventory for the waste paper sorting process available in Ecoinvent (global market including *Switzerland*, *Europe without Switzerland,* and *Rest of the World*).[3] These datasets include the required unsorted wastepaper input to generate a unit of sorted waste (weighted average 1.02kg per kg across geographies)[3], which can be used as an estimate of the required collection rate. Based on these data, to maintain mass balance for closed-loop recycling we estimated that for every 1 Mt of pulp produced in Scenario 2, 0.75 Mt was recycled pulp, produced from 0.70 Mt of waste paper, which required 0.71 Mt of waste paper to be collected accounting for sorting losses, leaving 0.29 Mt to be collected as mixed waste for market disposal.

These datasets include Ecoinvent market transport assumptions for source separated paper and delivery to the sorting facility, transport from sorting to recycling site, sorting and recycling process-specific resources and emissions, industrial facilities and land use and transformation, machinery production and maintenance, and waste treatment of process losses. All transport inputs include transportation modes and distances, infrastructure and vehicle construction and maintenance, fuel production, emissions, waste treatment.[3]

3.3.4. Waste Management

The remainder of the waste was assumed to be disposed of via mixed waste collection (Scenario 1: 100% and Scenario 2: 29%), for which we used geography specific market disposal datasets for waste paper in Ecoinvent that include: *Albania, Austria, Belgium, Bosnia and Herzegovina, Brazil, Bulgaria, Columbia, Croatia, Cyprus, Czech Republic, Denmark, Estonia, Finland, France, Germany, Greece, Hungary, Iceland, India, Ireland, Italy, Kosovo* (not included in the LCI as no waste generation data), *Latvia, Lithuania, Luxembourg, Macedonia, Malta, Montenegro, Netherlands, Norway, Peru, Poland, Portugal, Romania, Serbia, Slovenia, Slovakia, South Africa, Spain, Sweden, Switzerland, United Kingdom,* and *Rest of the World.*[3] These datasets contain national-level proportions of paper waste sent to landfill (sanitary and unsanitary), incineration, open dumps, and open

burning.[3] We combined datasets to create a geographically weighted average according to proportional contribution to global paper waste generation, taken from the World Bank *What a Waste* dataset.[81] The datasets contain waste collection transportation assumptions including transportation modes and distances, infrastructure and vehicle construction and maintenance, fuel production, emissions, waste treatment, alongside the waste disposal process-specific resources consumed and emissions generated (including both short- and long-term emissions from landfill), with any allocated burdens of land use and waste treatment facility construction, and subsequent treatment of residual materials, for example the landfilling of incineration residue and the leachate treatment from engineered landfills.[3]

3.3.5. Scenario Comparison

We compared the Human Health impacts of the two life cycle scenarios for Paper and Coated Paper **(Figure S4, Table 17).** Scenario 2 including 75% recycled pulp content had lower Human Health impacts than Scenario 1 based on 100% virgin paper production, therefore we selected Scenario 2 for both Paper and Coated Paper for inclusion in the combined P2O and LCA analysis.

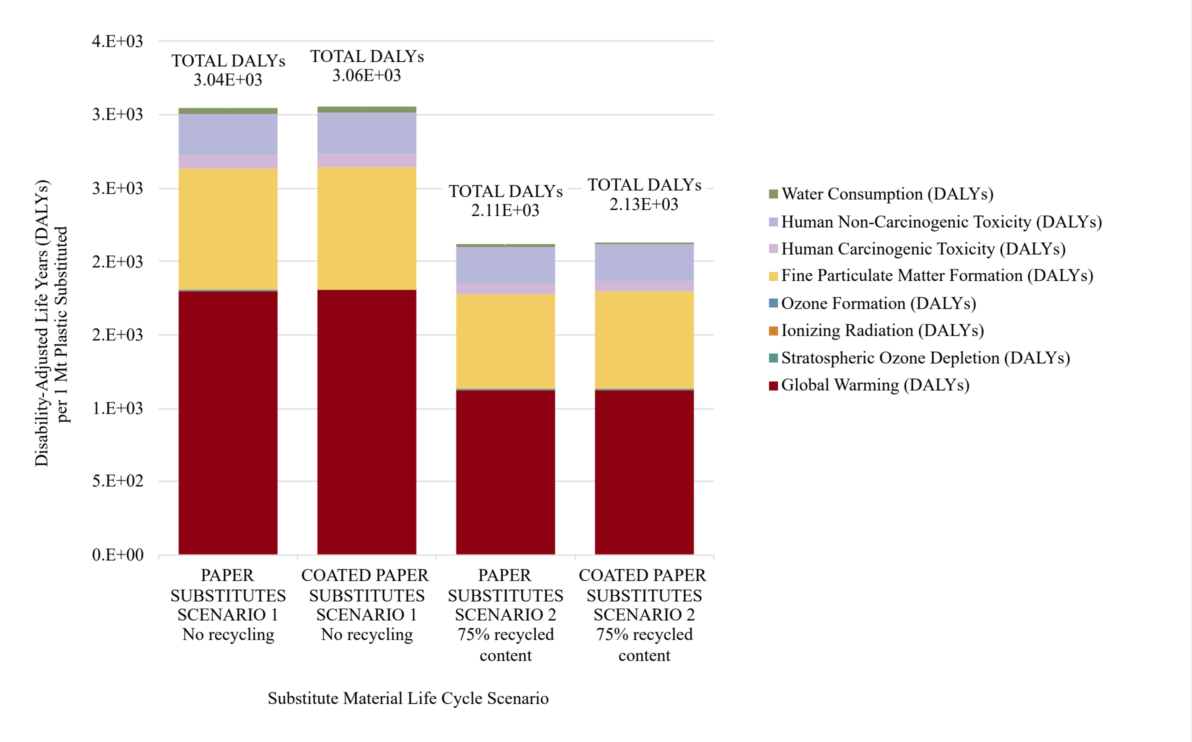

Figure S4. Paper and Coated Paper cradle-to-grave life cycle scenario comparison: impact assessment on Human Health in Disability-Adjusted Life Years (DALYs) by midpoint impacts*

Impact assessment conducted per million metric tonnes of plastic substituted by equivalent mass of paper and coated paper) The paper and coated paper cradle-to-grave assessment includes the impacts of: Scenario 1: 100% virgin pulp production, post-consumer collection and transport and 100% market disposal with no recycling, Scenario 2: 25% virgin pulp production, 75% recycled pulp production, post-consumer collection and transport and 29% market paper disposal. Impact assessment conducted using ReCiPe 2016 Hierarchic perspective impact assessment method.[5] Abbreviations: Disability-Adjusted Life Years (DALYs).

Table S17. Paper and Coated Paper cradle-to-grave life cycle scenario comparison impact assessment on Human Health in Disability-Adjusted Life Years (DALYs) by midpoint impacts*

ReCiPe 2016 IMPACT CATEGORY	PAPER SUBSTITUTES SCENARIO 1 No recycling	COATED PAPER SUBSTITUTES SCENARIO 1 No recycling	PAPER SUBSTITUTES SCENARIO 2 75% recycled content	COATED PAPER SUBSTITUTES SCENARIO 2 75% recycled content
	Disability-Adjusted Life Years per million metric tonnes of plastic substituted (DALYs)			
**Global Warming (DALYs)**	1.80E+03	1.80E+03	1.12E+03	1.12E+03
**Stratospheric Ozone Depletion (DALYs)**	1.84E-01	1.84E-01	2.39E-01	2.40E-01
**Ionizing Radiation (DALYs)**	8.20E-02	8.39E-02	2.78E-01	2.79E-01
**Ozone Formation (DALYs)**	2.31E+00	2.32E+00	1.13E+01	1.13E+01
**Fine Particulate Matter Formation (DALYs)**	8.34E+02	8.41E+02	6.52E+02	6.59E+02
**Human Carcinogenic Toxicity (DALYs)**	8.69E+01	8.78E+01	6.93E+01	7.03E+01
**Human Non-Carcinogenic Toxicity (DALYs)**	2.80E+02	2.82E+02	2.49E+02	2.52E+02
**Water Consumption (DALYs)**	4.09E+01	4.10E+01	1.48E+01	1.50E+01
**TOTAL DALYs**	3.04E+03	3.06E+03	2.11E+03	2.13E+03

Notes: Impact assessment conducted per million metric tonnes of plastic substituted by equivalent mass of paper and coated paper. The paper and coated paper cradle-to-grave assessment includes the impacts of: Scenario 1: 100% virgin pulp production, post-consumer collection and transport and 100% market disposal with no recycling, Scenario 2: 25% virgin pulp production, 25% recycled pulp production, post-consumer collection and transport and 29% market paper disposal. Impact assessment conducted using ReCiPe 2016 Hierarchic perspective impact assessment method.[5] Abbreviations: Disability-Adjusted Life Years (DALYs).

*Impact data were updated in 2024 to incorporate the ecoinvent v3.10 release. The final impact assessment results based on updated inventory data are available in **Section 5. Results: Life Cycle Impact Assessment.**

3.3.6. Regionalisation Summary

Virgin pulp production datasets were not regionalised by electricity mix in accordance with the main plastic system, as P2O is based on waste data indicating regional demand for materials rather than regional production.[1,2,52] The global market datasets were therefore considered to better reflect the international trade of paper production inputs. Subsequent stages of the paper life cycle were regionalised including the electricity inputs of the recycled pulp production, reorganised by country income classifications; the electricity inputs of the waste paper sorting process; and a geographical regionalisation by country income classification[52] of the market disposal datasets in Ecoinvent.[3] Further details on regionalisation of inventories are available in **Section 4. Regionalisation: Electricity Mix.**

3.4. Substitute: Single-Use Compostables

Bio-based, biodegradable, and compostable plastics are terms that describe a growing range of materials purported to be sustainable alternatives to fossil-based plastics.[82,83] Often these terms are confused and certain amongst them overlap. Bio-based plastics are made from biological resources but can behave very similarly to their fossil fuel-based counterparts; they are not necessarily biodegradable or compostable.[83] Biodegradable plastics is an umbrella term to describe plastics that biodegrade under a range of conditions.[83] Compostable plastics are a sub-set of biodegradable plastics and typically require industrial composting treatment to decompose. Biodegradable and compostable plastics may be made of fossil or biological resources.[82,83]

Polylactide, or polylactic acid (PLA), is a bio-based and biodegradable compostable polymer made from lactic acid derived from corn (or other plants).[84] Bio-based plastics occupy a small share of the global plastics market, projected to account for only 0.5% of total plastic production even by 2060.[8] PLA is currently the most widely produced and consumed bio-based plastic.[85] Limited inventory data are available for any bio-based plastics and Ecoinvent contains information on PLA only.[3]

3.4.1. System Boundaries

We created two illustrative life cycle scenarios for PLA that included: (1) 100% industrial incineration at the end-of-life and (2) 100% industrial composting. The system boundaries for these comparative scenarios are shown in **Figure S5**.

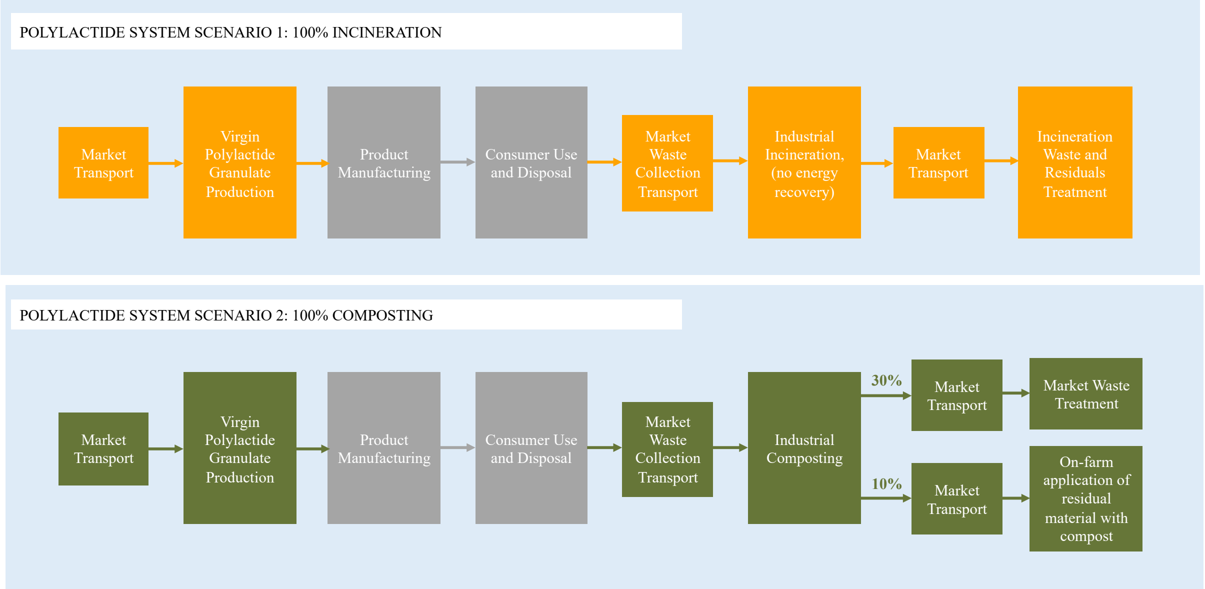

Figure S5. System boundaries for cradle-to-grave life cycle scenarios of polylactide (PLA)
Cradle-to-grave assessment based on: Scenario 1: 100% virgin PLA production, post-consumer collection and transport and 100% incineration, Scenario 2: 100% virgin PLA production, post-consumer collection and transport and 100% industrial composting. Product manufacturing and consumer use (in grey) are considered outside of the system boundaries for equivalence with the main plastic system in the Plastics-to-Ocean (P_2O) model.[1,2]

3.4.2. Polylactide Granulate Production

A global market dataset for PLA granulate production is available in Ecoinvent, which is equivalent to the production process applied for polymers in the main plastic system.[3] This is a geographically generic dataset (no geographical sub-group production datasets), based on data from the largest PLA production plant, which belongs to NatureWorks in Nebraska.[3] The dataset represents 100% virgin granulate production that includes process-specific resources, emissions and waste treatment, industrial production facility and land transformation, onwards transport from granulate production to the consumer of granulate including transportation modes and distances, infrastructure and vehicle construction and maintenance, fuel production, emissions and waste treatments.[3]

3.4.3. Collection, Sorting and Transportation

Waste collection and transportation was assumed to be the same as for municipal collection of mixed waste in the main plastic system as there is currently no separate municipal waste stream for compostable polymers. The same transportation was applied for both 100% incineration and 100% composting scenarios. Further details are available in **2.2.1. Municipal Mixed Waste Collection and Transport of Process Losses**. The inventory includes freight lorry manufacturing and maintenance, road construction and maintenance, fuel production (diesel), transport-specific emissions to air, and the waste treatment of break, road, and tyre-wear emissions.[3]

3.4.4. Waste Management: Incineration

There are no available Ecoinvent datasets for waste management of PLA.[3] For this reason we created a proxy dataset based on incineration of polyethylene,[3] with modified emissions to reflect the chemical composition of PLA, based on guidance from the JRC[78]. We removed emissions to air and water of all metals: Aluminium, antimony, arsenic, barium, beryllium, cadmium, calcium, chromium, cobalt, copper, iron, lead, magnesium,

manganese, mercury, nickel, sodium, strontium, thallium, tin, titanium, vanadium, zinc and replaced fossil carbon dioxide with biogenic carbon dioxide, set to zero. The remaining incineration datasets include the quantities of process-specific resources consumed and modified emissions generated, allocated burdens of land use and waste treatment facility construction, and subsequent treatment of residual materials including the landfilling of incineration residue.[3] Geographic datasets for *Switzerland* and *Rest of the World* were combined on the basis of proportional contribution to global plastic waste generation.[41,44]

3.4.5. Waste Management: Composting

For the purposes of this analysis, we modelled industrial composting defined as "a biological treatment process where biodegradable waste is typically converted, under aerobic conditions, into carbon dioxide, water, smaller amounts of methane and Non-Methane Volatile Organic Compounds (NMVOC), mineral salts of any other elements present in the waste, and into a residual solid fraction of simpler organic compounds (the compost), which is the main output of the process."[72] This is distinguished from home composting, and from industrial anaerobic digestion, which is carried out under anaerobic conditions to produce biogas which can be used as a fuel or energy substitute.[72]

Since there are no existing Ecoinvent datasets for PLA composting, we adapted the only available dataset in Ecoinvent for industrial composting of biowaste,[3] modified using PLA composition data taken from JRC case studies[78] and assumptions detailed in JRC methods guidance for LCA of alternative feedstocks for plastic.[72] Biowaste composting datasets are available in Ecoinvent for the geographies of *Switzerland* and *Rest of the World*.[3]

On arrival at the composting facility, JRC assumes 30% of material is screened out and sent to waste disposal.[72] We therefore adapted Ecoinvent biowaste datasets to account for this, using existing links to market municipal solid waste treatment datasets scaled to account for 30% of material input. Biowaste composting dataset inputs for the composting facility, machine operation, diesel and electricity were all kept the same.[3] Of the remaining 70% of material composted after screening, 90% of carbon and volatile solids are assumed to degrade during the process.[72] Based on PLA composition (**Table 18**)[78], we used JRC guidance **(Table 19)**[72] to estimate the carbon dioxide, methane, ammonia, dinitrogen monoxide and nitrogen gas emissions to air from industrial composting **(Table 20)**. We calculated conversion factors to translate contained chemical mass into emission mass by calculating the total mass of an emission compound (molar mass[86] multiplied by the number of moles for each element within the compound), divided by the mass of the individual chemical of interest **(Table 20).** Emissions per unit of PLA industrially composted were added to the Ecoinvent dataset.

Table S18. Polylactide (PLA) chemical composition[78]

Element	Polylactide Polymer Composition
Total Solids (TS)	100%
Water	0%
Volatile Solids (% TS)	100
Ash (% TS)	0
Fossil Carbon (% TS)	-
Biogenic Carbon (% TS)	49.5
Hydrogen (% TS)	5.60
Oxygen (% TS)	44.5
Nitrogen (% TS)	0.1
Sulphur (% TS)	0.3

Notes: Recreated from the Joint Research Centre (JRC) Life Cycle Assessment Case Studies on alternative feedstocks for plastics production[78]

Table S19. European Commission Joint Research Centre (JRC) recommendations modelling industrial composting[72]

Parameter	Unit	Requirement/recommendation
Carbon Dioxide (CO_2)	kg/kg waste	99.99% of mineralised carbon in the product/material
Methane (CH_4)	kg/kg waste	0.01% of mineralised carbon in the product/material
Ammonia (NH_3)	kg/kg waste	98.50% of N content in the product/material
Dinitrogen Monoxide (N_2O)	kg/kg waste	1.40% of N content in the product/material
Nitrogen gas (N_2)	kg/kg waste	0.10% of N content in the product/material

Notes: Recreated from the Joint Research Centre (JRC) guidance for Life Cycle Assessments of alternative feedstocks for plastics production.[72] JRC recommended proportions of chemical compounds emitted to air during industrial composting on the basis of material chemical composition.

Table S20. Estimated emissions to air per unit of polylactide (PLA) industrially composted

Total waste polymer (kg)	Total waste after screening (70%) (kg)	Total degraded (90%) (kg)	Waste Polymer Material composition			
1kg	0.7kg	0.63kg	Biogenic Carbon (49.5%)	**Carbon emissions to air**		
			0.31kg	Biogenic carbon to carbon dioxide		
				Contained carbon converted (99%)	Chemical mass conversion factor	Carbon Dioxide (kg)
				0.31kg	3.66	1.14kg
				Biogenic carbon to methane		
				Contained carbon converted (0.01%)	Chemical mass conversion factor	Methane (kg)
				0.000031kg	1.34	0.000042kg
			Nitrogen (0.1%)	**Nitrogen emissions to air**		
			0.00063kg	Nitrogen to Ammonia		
				Contained nitrogen converted (98.5%)	Chemical mass conversion factor	Ammonia (kg)
				0.00062kg	1.22	0.00076kg
				Nitrogen to Dinitrogen Monoxide		
				Contained nitrogen converted (1.4%)	Chemical mass conversion factor	Dinitrogen Monoxide (kg)
				0.0000088kg	1.57	0.000014kg
				Nitrogen to Nitrogen gas		
				Contained nitrogen converted (0.1%)	Chemical mass conversion factor	Nitrogen gas (kg)
				0.00000063kg	4.43	0.0000028kg

Notes: Detailed process of estimating emissions to air from industrial composting of polylactide (PLA) on the basis of chemical composition. Specific recommendations recreated from the Joint Research Centre (JRC) guidance for Life Cycle Assessments of alternative feedstocks for plastics production[72] and applied to chemical composition taken from JRC case studies.[78] Resulting estimates on the basis of JRC guidance are the authors' own.

We assume that the 10% residual material from the composting processes is spread on fields with compost produced by the composting plant, though no credits are applied for avoided fertilizer since no nutrients are contained in this polymer.[72] To include this final step in the LCI we assumed the same transport as for the original collection of PLA waste and transportation to the composting facility, applied at 10% to reflect the residual mass. We created a new dataset in Ecoinvent for the process of on-farm spreading of compost with data sourced from the JRC[72]. The created dataset includes estimated diesel requirements for spreading operations, air emissions (carbon dioxide, methane, ammonia, dinitrogen monoxide), emissions to groundwater via leaching (nitrate) and emissions to surface water via runoff (nitrate),[72] which were calculated on the basis of PLA composition.[78]

3.4.6. Scenario comparison

We compared the Human Health impacts of the two life cycle scenarios for PLA (**Figure S6, Table 21**). Scenario 1 based on 100% incineration at the end-of-life had lower Human Health impacts than Scenario 2 with 100% composting, therefore Scenario 1 was selected for inclusion in the combined P_2O and LCA analysis.

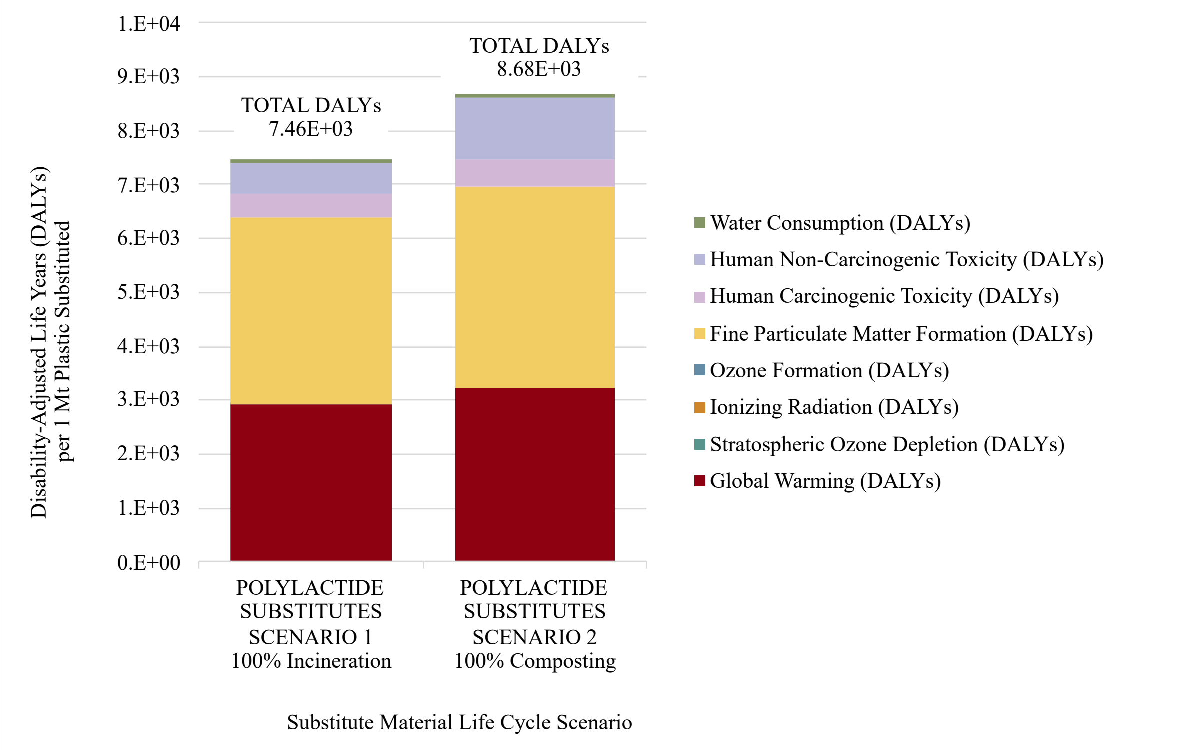

Figure S6. Polylactide (PLA) cradle-to-grave life cycle scenario comparison: impact assessment on Human Health in Disability-Adjusted Life Years (DALYs) by midpoint impacts*
Impact assessment conducted per million metric tonnes of plastic substituted by equivalent mass of polylactide (PLA). The PLA cradle-to-grave assessment includes the impacts of: Scenario 1: 100% virgin production, transportation and 100% incineration and, Scenario 2: 100% virgin production, transportation and 100% industrial composting. Impact assessment conducted using ReCiPe 2016 Hierarchic perspective impact assessment method.[5] Abbreviations: Disability-Adjusted Life Years (DALYs).

Table S21. Polylactide (PLA) cradle-to-grave life cycle scenario comparison impact assessment on Human Health in Disability-Adjusted Life Years (DALYs) by midpoint impacts*

ReCiPe 2016 IMPACT CATEGORY	POLYLACTIDE SUBSTITUTES SCENARIO 1 100% Incineration	POLYLACTIDE SUBSTITUTES SCENARIO 2 100% Composting
	Disability-Adjusted Life Years per million metric tonnes of plastic substituted (DALYs)	
**Global Warming (DALYs)**	2.92E+03	3.21E+03
**Stratospheric Ozone Depletion (DALYs)**	5.91E+00	5.92E+00
**Ionizing Radiation (DALYs)**	1.62E+00	1.63E+00
**Ozone Formation (DALYs)**	7.82E+00	7.68E+00
**Fine Particulate Matter Formation (DALYs)**	3.44E+03	3.75E+03
**Human Carcinogenic Toxicity (DALYs)**	4.64E+02	4.92E+02
**Human Non-Carcinogenic Toxicity (DALYs)**	5.46E+02	1.14E+03
**Water Consumption (DALYs)**	7.09E+01	7.13E+01
**TOTAL DALYs**	7.46E+03	8.68E+03

Notes: Impact assessment conducted per million metric tonnes of plastic substituted by equivalent mass of polylactide (PLA). The PLA cradle-to-grave assessment includes the impacts of: Scenario 1: 100% virgin production, transportation and 100% incineration and, Scenario 2: 100% virgin production, transportation and 100% industrial composting. Impact assessment conducted using ReCiPe 2016 Hierarchic perspective impact assessment method.[5] Abbreviations: Disability-Adjusted Life Years (DALYs).

*Impact data were updated in 2024 to incorporate the ecoinvent v3.10 release. The final impact assessment results based on updated inventory data are available in **Section 5. Results: Life Cycle Impact Assessment.**

3.4.7. Regionalisation Summary

Virgin PLA production datasets were not regionalised by electricity mix in accordance with the main plastic system, as P_2O is based on waste data indicating regional demand for materials rather than regional production.[1,2] The global market datasets were therefore considered to better reflect the international trade of potential production inputs. Incineration of PLA was regionalised according to country income classification[52] using available datasets in Ecoinvent (*Switzerland* and *Rest of the World*).[3] Further details on regionalisation of inventories are available in **Section 4. Regionalisation: Electricity Mix.**

3.5. Substitute: Reusable Glass Systems

Reuse is a cornerstone of circular economy approaches to reduce waste and covers a broad range of possible products, actions and supporting systems. Reuse can include refillable packaging systems, including at-home, on-the-go and in-store refills, returnable systems including deposit return schemes, and the reuse of service ware in the hospitality industry or business-to-business packaging for transportation between industries for example.[87]

P_2O estimates the amount of single-use plastic in the main MFA system that could be substituted by (1) Consumer Reuse and (2) New Delivery Models for reusable products.[1,2] Using expert stakeholder engagement, plastics product categories were scored on their potential to be substituted by reuse systems according to perceived technology readiness level, performance, convenience, and cost. These scores determined the categories of plastics products presumed to be potentially substitutable (**Table 22**) and the proportion of each product category subsequently modelled as substituted according to the feasibility of implementing reuse systems in different geographic archetypes and within time-based limits reflecting market penetration lags.[1,2] Some demand for single-use plastics was assumed to be met through reusable plastic products that were accounted for in P_2O plastic flows within the *Current Commitments*, *Reduce and Substitute* and *System Change* scenarios. P_2O also provided data on the quantity of plastic replaced by non-plastic reuse systems, which are suggested to be based on glass, metal, and fibre-based packages, but does not model the substitute systems themselves.[1,2]

Whilst reuse systems may be effective in terms of reducing single-use plastic pollution, these systems can introduce their own life cycle environmental and health burdens, which are important to assess to account for any trade-offs in single-use substitutions. We created illustrative scenarios to explore possible impacts of different forms of reuse to include in P_2O global scenario assessments. The broad range of possible reuse systems and the almost infinite range of modelling assumptions from materials, weight, and dimensions, washing and transportation, product life span, and final disposal, means that these scenarios should only be considered as exploratory and were clearly not designed to be representative of all systems on the global scale.

To compare the impacts of reusable systems within the parameters of the P_2O model, we defined the functional unit as the reusable packaging system required to provide equivalent packaging service to 1 Mt of single-use plastics over one year.

Table S22. Categories of Plastics-to-Ocean (P_2O) plastic products assessed as substitutable within Consumer Reuse and New Delivery Models[1,2] and possible non-plastic materials for reusable systems

P_2O plastic	P_2O products assessed as substitutable	% plastic in HIC	% plastic in LMIC	P_2O specified reusable material	Possible reusable system materials based on published literature
CONSUMER REUSE					
RM	Water bottles	1%	1%	Glass, metal and fibre-based	**Glass/Aluminium**<br>Life Cycle Initiative Review: Water Bottles (glass/aluminium)[88]
	On premise food service	4%	3%		**Glass/Stainless steel**

P_2O plastic	P_2O products assessed as substitutable	% plastic in HIC	% plastic in LMIC	P_2O specified reusable material	Possible reusable system materials based on published literature
					Life Cycle Initiative Review: Takeaway containers (glass)[89]<br>Life Cycle Initiative Review: Tableware (stainless steel)[90]
	Fruit/vegetable/meat packaging	8%	5%		**Glass**<br>Life Cycle Initiative Review: Supermarket Packaging: (honey: glass)[91]
FM	Carrier bags	4%	8%	Plastic	N/A: 100% plastic accounted for in P_2O[1,2]
MM	Sanitary items, Wet Wipes, Diapers	2%	2%	Glass, metal and fibre-based	**Cotton**<br>Life Cycle Initiative Review: Nappies (cotton)[92]
	**TOTAL**	**19%**	**19%**		
			NEW DELIVERY MODELS		
RM	Water bottles	1%	1%	Glass, metal, and fibre-based packages	**Glass/Aluminium**<br>Life Cycle Initiative Review: Water Bottles (Glass/Aluminium)[88]
RM	Other food grade bottles	8%	5%	Glass, metal, and fibre-based packages	**Glass/Aluminium**<br>Life Cycle Initiative Review: Water Bottles (Glass/Aluminium)[88]<br>Life Cycle Initiative Review: Supermarket packaging (milk: glass)[91]
RM	Non-food bottles	6%	4%	Plastic	N/A: 100% plastic accounted for in P_2O[1,2]
RM	Off premise plastic cups, lids, containers, and clamshells	4%	3%	Glass, metal, and fibre-based packages	**Glass/Stainless steel**<br>Life Cycle Initiative Review: Takeaway containers (glass)[89]<br>Life Cycle Initiative Review: Tableware (stainless steel)[90]
RM	Fresh fruit/vegetable/creams and liquids, deserts, and ready meal packaging	8%	5%	Glass, metal, and fibre-based packages	**Glass**<br>Life Cycle Initiative Review: Supermarket Packaging: (honey: glass)[91]
RM	Pallets, crates, B2B packaging	4%	2%	Plastic	N/A: 100% plastic accounted for in P_2O[1,2]
FM	Pouches, trash bags, wraps, 6-rings, nettings, and other flexibles	16%*	30%*	*33% plastic, 64% glass, metal and fibre-based	**Glass**<br>Life Cycle Initiative Review: Supermarket Packaging: (honey: glass)[91]
FM	B2B films	4%	7%	Plastic	N/A: 100% plastic accounted for in P_2O[1,2]
MM	Sachets and multilayer flexibles	4%*	18%*	*33% plastic, 64% glass, metal and fibre-based	**Glass**<br>Life Cycle Initiative Review: Supermarket Packaging: (honey: glass)[91]
MM	Plastic component of laminated aluminium	2%	0%	Glass, metal and fibre-based	**Glass**<br>Life Cycle Initiative Review: Supermarket Packaging: (honey: glass)[91]
MM	Diapers	2%	2%	Glass, metal and fibre-based	**Cotton**<br>Life Cycle Initiative Review: Nappies (cotton)[92]
	**TOTAL**	**48.4%**	**54.16%**		

Notes: Categories of plastic and substitution assessment recreated from Lau et al (2020).[1] Possible materials for reusable systems assessed through United Nations Environment Programme (UNEP) Life Cycle Initiative reviews of Life Cycle Assessment studies of single-use plastics and their alternatives.[88–92]

We used the Life Cycle Initiative publication series of product-specific reviews[88–92] of Life Cycle Assessments of single-use plastics and their alternatives to consider possible materials for the reuse systems (**Table 22**) Glass was the most commonly assessed alternative to single-use plastics across products as identified in existing LCA. As an initial exploratory evaluation, we designed one reusable glass packaging format for both Consumer Reuse and New Delivery Model systems, that could feasibly substitute most packaging categories indicated in P_2O. This illustrative reusable product therefore does not account for the substitution of diapers, as this category constitutes only 2% of all plastics in P_2O we considered this acceptable for the purpose of our analysis, but it could be a useful focus of future assessments. We reviewed data from individual studies included in the Life Cycle Initiative reviews[88–92] to determine the average weight of reusable glass packaging alternatives (511g) used to provide 1l or 1kg of food or drink (**Table 23**).

Table S23. Substitution rates of single-use plastic packaging with reusable glass containers

Study	Product	Volume	Glass reusable	Plastic single-use	Weight Ratio
Amienyo et al 2013[93]	Bottle	1 litre	797 grams	47.9/21.4 grams	17/37:1
Postacchini et al. 2018[94]	Honey jar	1 kilogram	323.7 grams	N/A	
Stefanini et al. 2021[95]	Bottle	1 litre	400 grams	22 grams	18:1
Greenwood et al. 2021[87]	Takeaway	300 grams	N/A	34.4 grams (x3.3)	
Gallego-Schmid et al. 2018[96]	Food containers	1.1 litre	575 grams (522.73/litre)	172 grams (reusable)	3:1
**Average container mass per litre/kilogram content**			**511 grams**	**51.49 grams**	**10:1**

Notes: Publications identified through searching Life Cycle Initiative reviews of Life Cycle Assessment studies of single-use plastics and their alternatives.[88,89,91,92] Extracted data on the weight of the single-use plastic product and the reusable glass alternative to estimate the average substitution ratio based on the mass of glass to plastic for the provision of packaging service for 1l or 1kg of food or drink.

3.5.1. System Boundaries

To be comparable with the single-use plastic system in P2O, the system boundaries for reusable glass life cycles included glass production, and final waste collection and disposal, with the added stages of reuse as suggested by JRC guidance to include washing (in households for Consumer Reuse systems and in industrial facilities for the New Delivery Models) and the transportation required for New Delivery Models (**Figure S7**).[72]

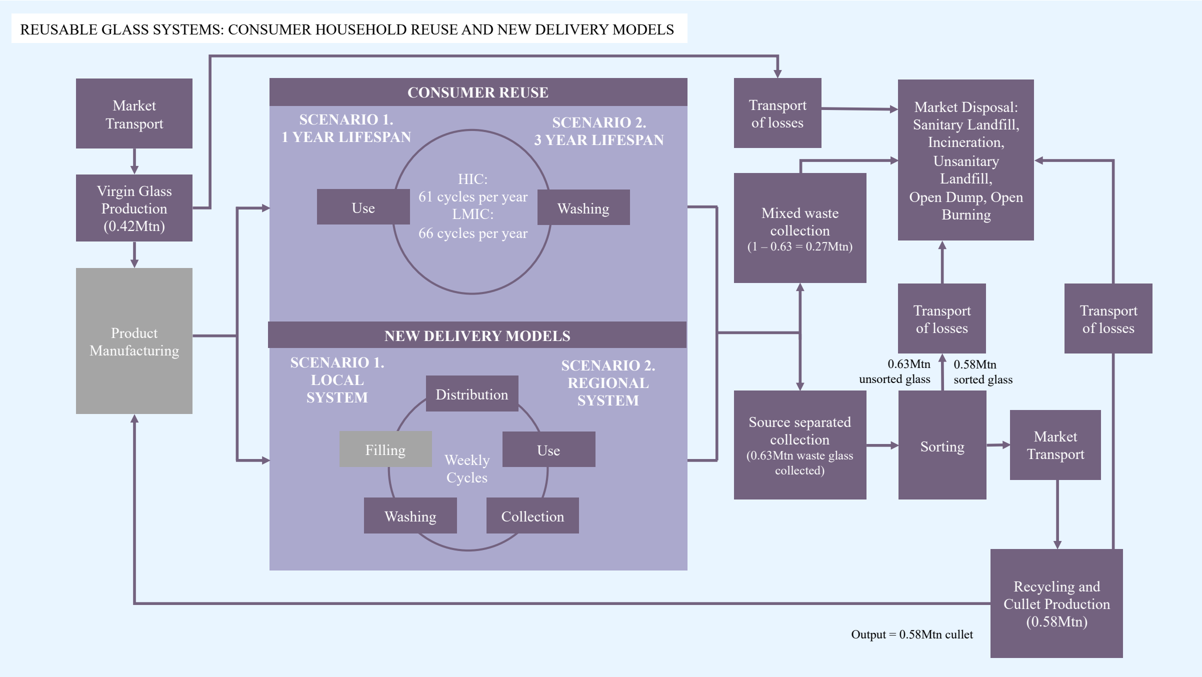

Figure S7. System boundaries for reusable glass systems based on Consumer Reuse and New Delivery Models

Cradle-to-grave system boundaries for reusable glass systems. Consumer reuse and New Delivery Models contained the same processes for glass production and end-of-life recycling and waste disposal. Consumer reuse systems contained the additional life cycle stage of washing the glass container, assumed to be carried out in households by hand or with dishwashers. Two scenarios were created to examine the influence of glass container lifespan within Consumer Reuse of (1) one year and (2) three years. New delivery models contained additional life cycle stages of washing, assumed to be carried out in an industrial facility, and transportation for the delivery and collection. Two scenarios were created within the New Delivery Model to reflect (1) a local delivery system with 50km of transportation by small van and (2) a regional delivery system with 1000km

transportation by lorry. Product manufacturing and filling stages (grey boxes) were not included in the system boundaries as in the main plastic system in the Plastics-to-Ocean model.

3.5.2. Glass Production and End-of-Life Disposal

Ecoinvent contains datasets for the market production of white packaging glass,[3] which we selected as being most appropriate (as opposed to coloured glass) for the predominantly food sector-related applications indicated by P_2O for reuse systems.[1,2] The datasets include production processes within the geographies of *Switzerland, Germany* (based on an LCA study),[3] *Europe without Switzerland and Germany* (based on information from EU-IPPC BAT document about the glass industry (2012),[3] *Global* production without cullet, and *Rest of the World* (extrapolated from European data).[3] Each dataset contained information on the proportion of cullet (recycled glass input) and virgin glass, with production process-specific resources, emissions and waste treatment, industrial facility, and onwards transport assumptions. Transport inputs include transportation modes and distances, infrastructure and vehicle construction and maintenance, fuel production, emissions, waste treatment.[3] We removed the Swiss dataset from the global market dataset as it was provided in aggregate and so it was impossible to decipher the quantity of recycled cullet included in the production. We adjusted the proportions of other geographies to offset this.

The geographically weighted average recycled cullet content in white glass production was 58%,[3] indicated through the inventory inputs of geography-specific quantities of waste glass sorting processes. The sorting processes (available for *Europe* and *Rest of the World*)[3] contained data on the required input of unsorted waste glass, from which we estimated that for every 1 Mt of white glass produced under market assumptions, on average 0.63 Mt of unsorted glass waste was required to be collected to produce the 0.58 Mt of recycled content,[3] for a closed-loop recycling scenario. The glass sorting datasets include transportation to and from the sorting process, including transport modes and distances, infrastructure and vehicle construction and maintenance, fuel production, emissions, and waste treatment, with the sorting process-specific resources and emissions, sorting facility construction and waste treatments.[3]

The remaining glass waste was presumed to be collected and disposed of through mixed waste streams, for which we used market waste glass datasets available in Ecoinvent.[3] These datasets are available for geographies including *Albania, Austria, Belgium, Bosnia and Herzegovina, Brazil, Bulgaria, Columbia, Croatia, Cyprus, Czech Republic, Denmark, Estonia, Finland, France, Germany, Greece, Hungary, Iceland, India, Ireland, Italy, Kosovo* (not included in the LCI as no waste generation data), *Latvia, Lithuania, Luxembourg, Macedonia, Malta, Montenegro, Netherlands, Norway, Peru, Poland, Portugal, Romania, Serbia, Slovenia, Slovakia, South Africa, Spain, Sweden, Switzerland, United Kingdom,* and *Rest of the World.*[3] These datasets contain national-level proportions of glass waste sent to landfill (sanitary and unsanitary), incineration, open dumps, and open burning.[3] Datasets contain waste collection transportation assumptions, including transport modes and distances, infrastructure and vehicle construction and maintenance, fuel production, emissions and waste treatment; waste disposal process-specific resources consumed and emissions generated (including both short- and long-term emissions from landfill), any allocated burdens of land use and waste treatment facility construction and decommissioning, and subsequent treatment of residual materials, for example the landfilling of incineration residues and the leachate treatment from engineered landfills.[3] We combined datasets for a geographically weighted average according to proportional contribution to global glass waste generation, based on the World Bank *What a Waste* dataset.[81]

3.5.3. Substitution Rates

We weighted substitution rates of single-use plastics with reusable glass containers by product type to increase accuracy in relation to demand for providing equivalent food and drink packaging service. We did not consider products that could not reasonably be substituted with a glass container and scaled up the other categories to account for 100% of the plastic substituted. Whilst the categories that were excluded are a relatively small proportion of the total plastics substituted, this is a current limitation that requires further exploration of different materials in reuse systems in future analyses.

For Consumer Reuse, there were three categories of single-use plastic products considered substitutable in the P_2O model and which we considered could reasonably be substituted with a glass container: water bottles, on-premise food service plastics and packaging for fresh fruit, vegetables and meats.[1,2] Based on literature sources detailed in **Table 23**, we estimated that packaging 1l or 1kg of food or drink, on average would require 31g of single-use plastic for drinks bottles, and 115g of plastic for food service and food packaging applications.[87,93–96]

We estimated an average 511g glass container would substitute these products, with a mass ratio of 16.48 and 4.44 respectively. We further assumed that reusable glass bottles would be used every day whereas reusable glass food service and food packaging products would be used once per week. Combining these data, we estimated the glass material mass and number of containers required to substitute 1 Mt of single-use plastic packaging during one year in HIC and in LMIC (**Table S24**).

For New Delivery Models, there were six categories of single-use plastic products considered substitutable in the P$_2$O model and which we considered could reasonably be substituted with a glass container: water bottles, other food grade bottles, on-premise food service plastics, primary food packaging, pouches and other flexibles, sachets and multilayer flexibles.[1,2] As for Consumer Reuse, we estimated an average mass of 31g of single-use plastic for bottles, 115g of plastic for food service and food packaging applications,[87,93–96] and 120g for flexibles.[97] The substitution rates with a 511g reusable glass container were estimated for HIC and LMIC on the basis of providing 1l or 1kg of packaging service, assuming weekly rotation of each container (**Table S24**).

Table S24. Estimated product-based substitution rates of single-use plastics with reusable glass containers

**P$_2$O Product**	**Average plastic mass per L/kg packaging service** (g)	**Average glass mass per L/kg packaging service** (g)	**Mass ratio** (glass: plastic)	**Frequency of reusable product use per year** (frequency)	**Product proportion of total plastic replaced in HIC** (%)	**Glass mass weighted by product substitution rate and frequency of product use in HIC** (Mt per year)	**Product proportion of total plastic replaced in LMIC** (%)	**Glass mass weighted by product substitution rate and frequency of product use in LMIC** (Mt glass per year/ Mt of plastic)
**CONSUMER REUSE**								
Water bottles	31g	511g	16.48	365 (daily)	7%	0.0032	11%	0.005
On premise food service	115g	511g	4.44	52 (weekly)	31%	0.026	33%	0.028
Food packaging	115g	511g	4.44	52 (weekly)	62%	0.053	56%	0.048
**Total weighted glass substitution** (per 1 Mt single-use plastics)						**0.082 Mt =161 million packaging items**		**0.081 Mt =159 million packaging items**
**NEW DELIVERY MODELS**								
Water bottles	31g	511g	16.48	52 (weekly)	3%	0.0095	2%	0.0063
Food grade bottles	31g	511g	16.48	52 (weekly)	23%	0.073	11%	0.035
Off premise takeaway packaging	115g	511g	4.44	52 (weekly)	12%	0.010	6%	0.0051
Food packaging	115g	511g	4.44	52 (weekly)	23%	0.020	11%	0.0094
Pouches, trash bags, and other flexibles	0.12g per g product (120g per kg)	511g	4.26	52 (weekly)	31%	0.025	44%	0.036
Sachets and multilayer flexibles	0.12g per g product (120g per kg)	511g	4.26	52 (weekly)	8%	0.0066	26%	0.021

P_2O Product	Average plastic mass per L/kg packaging service (g)	Average glass mass per L/kg packaging service (g)	Mass ratio (glass: plastic)	Frequency of reusable product use per year (frequency)	Product proportion of total plastic replaced in HIC (%)	Glass mass weighted by product substitution rate and frequency of product use in HIC (Mt per year)	Product proportion of total plastic replaced in LMIC (%)	Glass mass weighted by product substitution rate and frequency of product use in LMIC (Mt glass per year/ Mt of plastic)
**Total weighted glass substitution** (per 1 Mt single-use plastics)						**0.14 Mt =282 million packaging items**		**0.11 Mt =221 million packaging items**

Notes: Estimated on the basis of Life Cycle Initiative reviews of Life Cycle Assessment studies of single-use plastics and their alternatives.[88,89,91,92] Extracted data on the weight of the single-use plastic product and the reusable glass alternative to estimate the average substitution ratio based on the mass of glass to plastic for the provision of packaging service for 1l or 1kg of food or drink. Frequency of reuse estimated by authors and substitution rate estimated according to product categories in P_2O. Abbreviations: Plastics-to-Ocean (P_2O), million metric tonnes (Mt), High-Income Countries Archetype, Low- and Middle-Income Countries Archetype (LMIC).

The calculations in **Table S24** provide an estimate of the total amount of glass and the equivalent number of reusable glass containers required to provide the same packaging service as 1 Mt of single-use plastics, within one year. These data were used to inform our modelling of the use phase, as the number of glass containers that would require washing and transportation during the year per 1 Mt plastics substituted. It was also important to also consider the lifespan of reusable containers, which informed the total glass material input required and final waste disposal for our functional unit based on one year of product usage. According to JRC guidance, the impacts of material production and waste management for reuse systems should be allocated equally across the number of uses.[72]

For Consumer Reuse, we created two scenarios to reflect (1) a one-year lifespan of the reusable glass container, and (2) a three-year lifespan. Under the first scenario the quantity of glass required to substitute 1 Mt of single-use plastics during one year remains the same as indicated in **Table S24** under the second scenario the quantity is divided by three (0.0274 Mt of glass per year), which therefore accounts for the glass container substituting three times as much plastic over the course of its three-year lifespan. The number of washes per year remain the same in both scenarios as this related to the frequency of use within one year.

For New Delivery Models, we assumed that each container had a lifespan of 30 trips, based on JRC guidance.[72] Given that we assumed each container is used once per week, 1.67 containers were required to provide 1l or 1kg of packaging service provided weekly over the course of a year. The material input indicated in **Table S24** was therefore multiplied by 1.67 (0.24 Mt in HIC, 0.19 Mt in LMIC per year) to account for the shorter lifespan and material replacements for one year but the number of washes required remained the same. No scenarios were constructed for New Delivery Models on the basis of lifespan, instead we modified the transport assumptions, detailed in **Section 3.5.5. New Delivery Model**.

3.5.4. Consumer Reuse

We did not include consumer transportation in the inventory for Consumer Reuse as this was not included in the main plastic system and is not a functional part of the product's reuse (the product could be reused at home with no transport in the case of food storage for example). The number of washes during one year was assumed to be once every two days for water bottles (used every day but washed every other), and once per week for food service and storage containers. Based on product proportions in P_2O, this resulted in a weighted average of 61 washes per product per year for HIC and 66 washes LMIC.

We created inventories for washing by electric dishwasher and by handwashing. For dishwasher use, we allocated the impacts of the dishwasher and resource inputs per product per year on the basis of a dishwasher having a lifespan of 12.5 years (according to Ecoinvent),[3] used 365 days per year with or without the reusable glass container. We assumed that each full dishwasher cycle required 15l of water, 1kWh of electricity, and 30g of detergent[98,99], and that each reusable glass container (1l or 1kg capacity, weighing 511g) accounts for 4% of dishwasher capacity in one wash cycle, based on another LCA (**Table S25**)[96] For handwashing, based on published literature we estimated that 140 items (reported to be equivalent to 25 glass food savers)[100]

handwashed, on average required 103l of water, 2.5kWh of electricity, and 35g detergent, with 4% allocated to each container (**Table S26**).[100]

Based on published literature sources we estimated the proportion of dishwashing versus handwashing in P_2O geographical archetypes:

- HIC: 45% dishwasher use versus 55% handwashing[96]
- UMC and LMC: 10% dishwasher use versus 90% handwashing
- LIC: 0% dishwasher use, 100% handwashing[98,99]

For both dishwashing and handwashing we included wastewater treatment for 96% of water input using the *Rest of the World* wastewater from residence treatment dataset, assuming 4% of water input is lost as steam.[3]

Table S25. Inventory data for dishwashing one reusable glass container during one year

Inventory Input	Per container per year in HIC (Based on weighted average of 61 washes per container per year)	Per container per year in LMIC (Based on weighted average of 66 washes per container per year)
Dishwasher (n)	5.35E-04	5.79E-04
Water (l)	3.66E+01	4.00E+01
Electricity (kWh)	2.44E+00	2.60E+00
Detergent (g)	7.32E+01	7.90E+01
Wastewater treatment (l)	3.51E+01	3.84E+01

Table S26. Inventory data for handwashing one reusable glass container during one year

Inventory Input	Per container per year in HIC (Based on weighted average of 61 washes per container per year)	Per container per year in LMIC (Based on weighted average of 66 washes per container per year)
Water (l)	2.51E+02	2.68E+02
Electricity (kWh)	6.10E+00	6.50E+00
Detergent (g)	8.54E+01	9.10E+01
Wastewater treatment (l)	2.41E+02	2.57E+02

We used Ecoinvent *Global* market datasets for the inventories which included all process-specific resources and emissions associated with producing dishwashers, producing tap water, electricity and detergent, and with treating wastewater, the industrial facilities required and land use and transformation, machinery production and maintenance, and waste treatment of process losses. All transport inputs between inventory item production stages included transportation modes and distances, infrastructure and vehicle construction and maintenance, fuel production, emissions, waste treatment.[3]

3.5.5. New Delivery Model Reuse

For the New Delivery System of reuse we modelled two sub-scenarios, the first based on a small-scale, local delivery system and the second on a larger regional system. We considered greater transportation distances in the regional delivery and collection system, by lorry as opposed to small vans, and greater commercial dishwashing capacity in the washing facility (**Table S27**).

For the small-scale, local delivery scenario we modelled a 50km round-trip delivery service in a small van. We assumed that for every container delivered, another was collected therefore assuming constant weight within the van, allocating the burdens of the changing weight of container contents (full versus empty) to the food or drink item (not included in our functional unit). We assumed 100% commercial dishwasher use with a capacity of 150 containers per cycle, 15 minutes per cycles continuously over an 8-hour day (24 cycles per day), 7 days per week, for a total processing capacity of 25,200 containers per year per dishwasher. For the inputs of water (0.083l per container per wash) and electricity (0.083l per container per wash), we used data from an LCA of

commercial reuse delivery systems for glass honey jars in Italy.[94] This study did not consider detergent input and so we included the same requirements per container as in the case of the domestic dishwasher.[98,99] We included wastewater treatment for 96% of water input using the *Rest of the World* wastewater from residence treatment dataset, assuming 4% of water input is lost as steam.[3] We did not include the industrial washing and filling facility (assumed to be the same facility), the energy required to refill containers, nor the avoided burdens of consumer transport to supermarkets or food outlets as these stages were not equivalent with the boundaries of the main plastic system in P_2O.[1,2] Some reuse systems may rely on separate washing facilities to the filling site, therefore our impacts may underestimate these impacts.

For the regional delivery scenario, we modelled the same system with a 1000km round-trip on the basis of the LCA conducted for regional reuse of glass jars for honey in Italy.[94] We considered that the capacity of the commercial dishwasher could be greater and modelled 500 containers per cycle, 15 minutes per cycle operated continuously over an 8-hour day (24 cycles per day), 7 days per week, for a total processing capacity of 84,000 containers per year per dishwasher. All other assumptions and inventory data were consistent with the local delivery system. We did not include any consumer to supermarket or outlet transportation as this was not included in the main single-use plastic system.[1,2]

We used Ecoinvent *Global* market datasets for the inventories which included all process-specific resources and emissions associated with producing dishwashers, producing tap water, electricity and detergent, and with treating wastewater, the industrial facilities required and land use and transformation, machinery production and maintenance, and waste treatment of process losses. All transport inputs between inventory item production stages, and for the main delivery mechanism, included transportation modes and distances, infrastructure and vehicle construction and maintenance, fuel production, emissions, waste treatment.[3]

All inputs were scaled to reflect the number of containers estimated to be required to provide equivalent packaging service to 1 Mt of plastics in HIC and LMIC (reflecting different quantities of packaging types in these geographical archetypes) (**Table S27**).

Table S27. Inventories for the reuse cycles of New Delivery Models on local and regional scales

Inventory Input	Quantity for total containers processed per year per Mt plastic substituted in HIC *(282,259,521 glass containers)*	Quantity for total containers processed per year per Mt plastic substituted in LMIC *(221,255,457 glass containers)*
SCENARIO 1: LOCAL DELIVERY		
Transportation		
Delivery Transport (km) *(Small van)*	50km	50km
Washing		
Dishwasher (n)*	8.96E+02	7.02E+02
Water (l) *(0.083l per container per wash)*	2.35E+07	1.84E+07
Electricity (kWh) *(0.018kWh per container per wash)*	5.08E+06	3.98E+06
Detergent (g) *(1.2g per container per wash)*	3.39E+08	2.66E+08
SCENARIO 2: REGIONAL DELIVERY		
Transportation		
Delivery Transport (km) *(Lorry)*	1000km	1000km
Washing		
Dishwasher (n)*	2.69E+02	2.11E+02
Water (l) *(0.083l per container per wash)*	2.35E+07	1.84E+07
Electricity (kWh) *(0.018kWh per container per wash)*	5.08E+06	3.98E+06
Detergent (g) *(1.2g per container per wash)*	3.39E+08	2.66E+08

3.5.6. Scenario Comparison

We compared the Human Health impacts of the two life cycle scenarios for Consumer Reuse (**Figure S8** and **Table S28**) and for New Delivery Models (**Figure S9** and **Table S29**) Lower impacts were found across geographical archetypes for a three-year lifespan of the glass container under Consumer Reuse (Scenario 2) and the regional delivery system under New Delivery Models (Scenario 2), therefore these scenarios were included in the main analyses with P_2O.

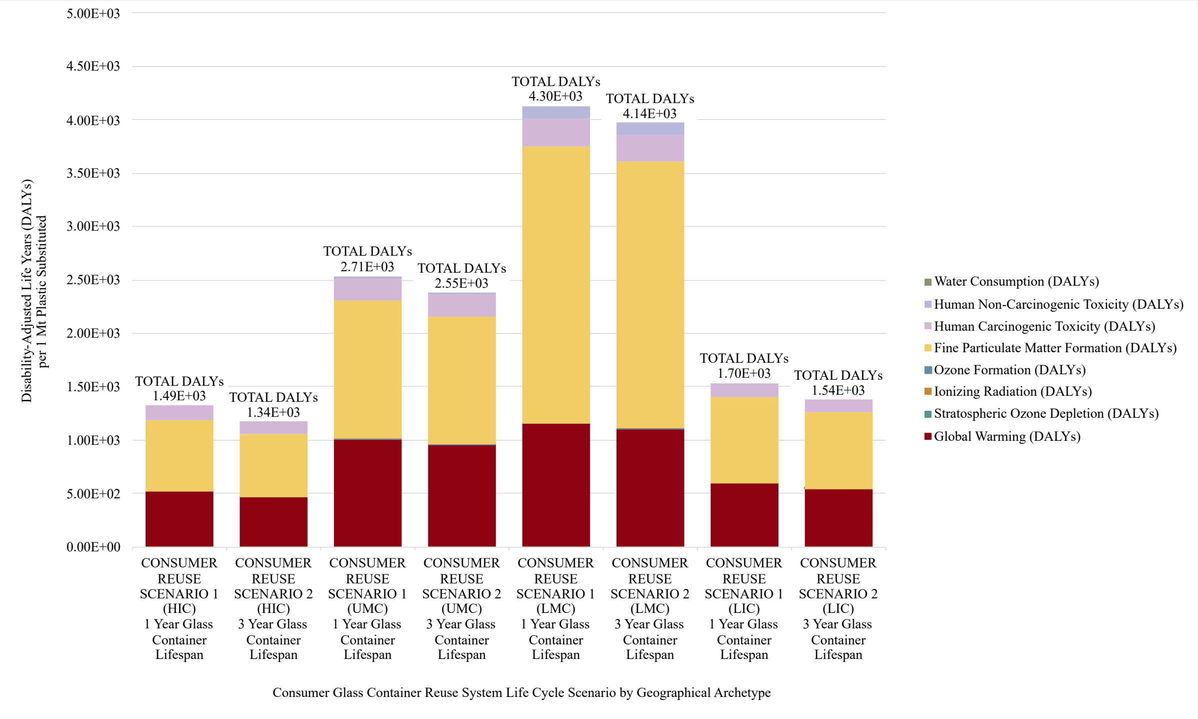

Figure S8. Consumer Reuse cradle-to-grave life cycle scenario comparison: impact assessment on Human Health in Disability-Adjusted Life Years (DALYs) by midpoint impacts*
Impact assessment conducted per million metric tonnes of plastic substituted by the reusable glass system on the basis of the functional unit of providing equivalent packaging service during one year. The glass system scenarios include the impacts of glass material production, washing and final waste disposal on the basis of: Scenario 1: one-year lifespan of the glass container and, Scenario 2: three-year lifespan of the glass container. Impact assessment conducted using ReCiPe 2016 Hierarchic perspective impact assessment method.[5]
Abbreviations: Disability-Adjusted Life Years (DALYs), High-Income Countries Archetype (HIC), Upper Middle-Income Countries Archetype (UMC), Lower Middle-Income Countries Archetype (LMC), Low-Income Countries Archetype (LIC).

Table S28. Consumer Reuse cradle-to-grave life cycle scenario comparison impact assessment on Human Health in Disability-Adjusted Life Years (DALYs) by midpoint impacts*

ReCiPe 2016 IMPACT CATEGORY	HIC Scenario. 1 1 year lifespan	HIC Scenario 2. 3 year lifespan	UMC Scenario. 1 1 year lifespan	UMC Scenario 2. 3 year lifespan	LMC Scenario. 1 1 year lifespan	LMC Scenario 2. 3 year lifespan	LIC Scenario. 1 1 year lifespan	LIC Scenario 2. 3 year lifespan
	Disability-Adjusted Life Years per million metric tonnes of plastic substituted (DALYs)							
**Global Warming (DALYs)**	5.19E+02	4.65E+02	1.01E+03	9.56E+02	1.16E+03	1.10E+03	5.95E+02	5.43E+02
**Stratospheric Ozone Depletion (DALYs)**	2.30E-01	2.17E-01	3.70E-01	3.57E-01	3.34E-01	3.20E-01	3.39E-01	3.26E-01
**Ionizing Radiation (DALYs)**	9.52E-01	9.33E-01	3.91E-01	3.72E-01	4.25E-01	4.06E-01	1.35E-01	1.16E-01
**Ozone Formation (DALYs)**	1.01E+00	8.28E-01	2.56E+00	2.38E+00	2.63E+00	2.45E+00	1.68E+00	1.50E+00
**Fine Particulate Matter Formation (DALYs)**	6.70E+02	5.87E+02	1.29E+03	1.20E+03	2.60E+03	2.51E+03	8.09E+02	7.19E+02

ReCiPe 2016 IMPACT CATEGORY	HIC Scenario. 1 1 year lifespan	HIC Scenario 2. 3 year lifespan	UMC Scenario. 1 1 year lifespan	UMC Scenario 2. 3 year lifespan	LMC Scenario. 1 1 year lifespan	LMC Scenario 2. 3 year lifespan	LIC Scenario. 1 1 year lifespan	LIC Scenario 2. 3 year lifespan
	Disability-Adjusted Life Years per million metric tonnes of plastic substituted (DALYs)							
**Human Carcinogenic Toxicity (DALYs)**	1.50E+02	1.42E+02	2.08E+02	2.00E+02	2.51E+02	2.43E+02	1.58E+02	1.50E+02
**Human Non-Carcinogenic Toxicity (DALYs)**	1.41E+02	1.31E+02	1.81E+02	1.71E+02	2.78E+02	2.69E+02	1.26E+02	1.17E+02
**Water Consumption (DALYs)**	1.23E+01	1.19E+01	1.52E+01	1.48E+01	1.42E+01	1.37E+01	1.33E+01	1.28E+01
**TOTAL DALYs**	1.49E+03	1.34E+03	2.71E+03	2.55E+03	4.30E+03	4.14E+03	1.70E+03	1.54E+03

Notes: Impact assessment conducted per million metric tonnes of plastic substituted by the reusable glass system on the basis of the functional unit of providing equivalent packaging service during one year. The glass system scenarios include the impacts of glass material production, washing and final waste disposal on the basis of: Scenario 1: one-year lifespan of the glass container and, Scenario 2: three-year lifespan of the glass container. Impact assessment conducted using ReCiPe 2016 Hierarchic perspective impact assessment method.[5] Abbreviations: Disability-Adjusted Life Years (DALYs), High-Income Countries Archetype (HIC), Upper Middle-Income Countries Archetype (UMC), Lower Middle-Income Countries Archetype (LMC), Low-Income Countries Archetype (LIC).

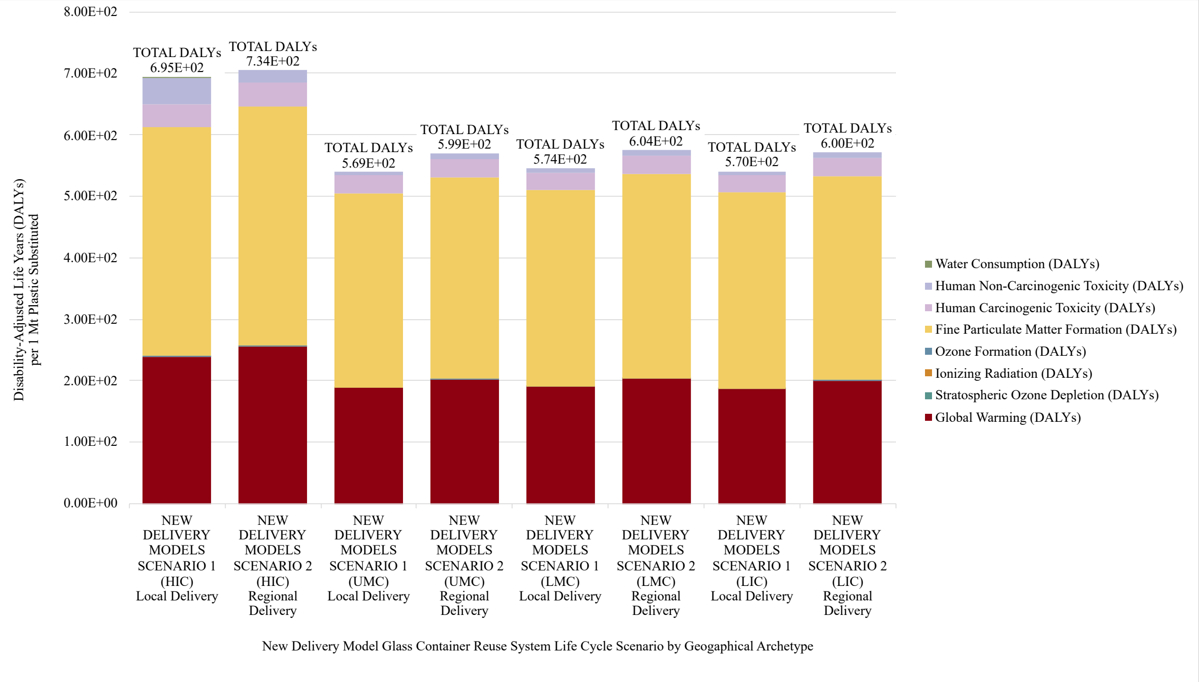

Figure S9. New Delivery Model reuse cradle-to-grave life cycle scenario comparison: impact assessment on Human Health in Disability-Adjusted Life Years (DALYs) by midpoint impacts*
Impact assessment conducted per million metric tonnes of plastic substituted by the New Delivery Model reusable glass system on the basis of the functional unit of providing equivalent packaging service during one year. The glass system scenarios include the impacts of glass material production, transportation and washing and final waste disposal Scenario 1: Local delivery system with 50km transport, Scenario 2: Regional delivery with 1000km transport and greater annual dishwashing capacity. Impact assessment conducted using ReCiPe 2016 Hierarchic perspective impact assessment method.[5] Abbreviations: Disability-Adjusted Life Years (DALYs), High-Income Countries Archetype (HIC), Upper Middle-Income Countries Archetype (UMC), Lower Middle-Income Countries Archetype (LMC), Low-Income Countries Archetype (LIC).

Table S29. New Delivery Model reuse cradle-to-grave life cycle scenario comparison impact assessment on Human Health in Disability-Adjusted Life Years (DALYs) by midpoint impacts*

	Life Cycle Scenario by Geographical Archetype							
	HIC		UMC		LMC		LIC	
**ReCiPe 2016 IMPACT CATEGORY**	**Scenario. 1 Local Delivery**	**Scenario 2. Regional Delivery**	**Scenario. 1 Local Delivery**	**Scenario 2. Regional Delivery**	**Scenario. 1 Local Delivery**	**Scenario 2. Regional Delivery**	**Scenario. 1 Local Delivery**	**Scenario 2. Regional Delivery**
	Disability-Adjusted Life Years per million metric tonnes of plastic substituted (DALYs)							
**Global Warming (DALYs)**	2.39E+02	2.56E+02	1.89E+02	2.02E+02	1.90E+02	2.04E+02	1.87E+02	2.00E+02
**Stratospheric Ozone Depletion (DALYs)**	6.00E-02	6.49E-02	4.99E-02	5.37E-02	5.26E-02	5.65E-02	5.00E-02	5.38E-02
**Ionizing Radiation (DALYs)**	9.09E-02	9.36E-02	6.74E-02	6.95E-02	6.75E-02	6.97E-02	6.63E-02	6.84E-02
**Ozone Formation (DALYs)**	8.14E-01	8.99E-01	6.44E-01	7.10E-01	6.48E-01	7.15E-01	6.40E-01	7.06E-01
**Fine Particulate Matter Formation (DALYs)**	3.73E+02	3.89E+02	3.16E+02	3.28E+02	3.18E+02	3.31E+02	3.19E+02	3.31E+02
**Human Carcinogenic Toxicity (DALYs)**	3.72E+01	3.95E+01	2.87E+01	3.05E+01	2.88E+01	3.06E+01	2.82E+01	3.00E+01
**Human Non-Carcinogenic Toxicity (DALYs)**	4.29E+01	4.58E+01	3.35E+01	3.58E+01	3.39E+01	3.61E+01	3.32E+01	3.55E+01
**Water Consumption (DALYs)**	2.16E+00	2.20E+00	1.69E+00	1.72E+00	1.69E+00	1.72E+00	1.68E+00	1.71E+00
**TOTAL DALYs**	6.95E+02	7.34E+02	5.69E+02	5.99E+02	5.74E+02	6.04E+02	5.70E+02	6.00E+02

Notes: Impact assessment conducted per million metric tonnes of plastic substituted by the New Delivery Model reusable glass system on the basis of the functional unit of providing equivalent packaging service during one year. The glass system scenarios include the impacts of glass material production, transportation and washing and final waste disposal Scenario 1: Local delivery system with 50km transport, Scenario 2: Regional delivery with 1000km transport and greater annual dishwashing capacity. Impact assessment conducted using ReCiPe 2016 Hierarchic perspective impact assessment method.[5] Abbreviations: Disability-Adjusted Life Years (DALYs), High-Income Countries Archetype (HIC), Upper Middle-Income Countries Archetype (UMC), Lower Middle-Income Countries Archetype (LMC), Low-Income Countries Archetype (LIC).

*Data were updated in 2024 to incorporate the ecoinvent v3.10 release. The final impact assessment results based on updated inventory data are available in **Section 5. Results: Life Cycle Impact Assessment.**

3.5.7. Regionalisation Summary

Glass production datasets were not regionalised by electricity mix in accordance with the main plastic system, as P_2O is based on waste data indicating regional demand for materials rather than regional production.[1,2] The global market datasets were therefore considered to better reflect the international trade of potential production inputs. The electricity inputs for domestic dishwashers, heating waster used for handwashing and for industrial dishwashers was regionalised according to geographical archetype electricity mixes. The proportion of domestic dishwashing versus handwashing was also regionalised according to published literature on practices in HIC, UMC/LMC and LIC countries.[96,98,99] Market waste disposal methods were regionalised according to country income classifications[52] of available national datasets in Ecoinvent. Further details on regionalisation of inventories are available in **Section 4. Regionalisation: Electricity Mix.**

4. Regionalisation: Electricity Mix

Electricity is a central requirement for most industrial processes, the production of which is responsible for around a quarter of global greenhouse gas emissions.[101] Electricity is generated in different ways; some is sourced from fossil fuels with high environmental impacts and other production methods are based on renewable energy including solar, wind or hydro power. Electricity production will often determine, to a large extent, the overall impacts of an industrial process, with highly variable impacts based on the production methods.[102]

Different countries and regions rely on different methods of electricity production in different proportions and at different scales (**Figure S10**).[103] For this reason, the same industrial process, requiring the same quantity of electricity, may present very different environmental impacts in different countries. The proportions of different electricity production methods used within a single process, country or region are referred to as the 'electricity mix'.

The P_2O model differentiated between four primary geographical archetypes: HIC, UMC, LMC and LIC. The quantity of plastic processed in different ways throughout the life cycle differs in these archetypes and changes at different rates over time (2016 – 2040). For this reason and given the likely importance of electricity mix in determining many of the life cycle impacts, we aimed to modify process inventories, where possible, with a regionalised electricity mix for HIC, UMC, LMC and LIC created on the basis of Ecoinvent country-level electricity production data[3] and their contributions to global electricity production, reapportioned and compiled according to World Bank Country Income Classifications to reflect P_2O archetypes.[52]

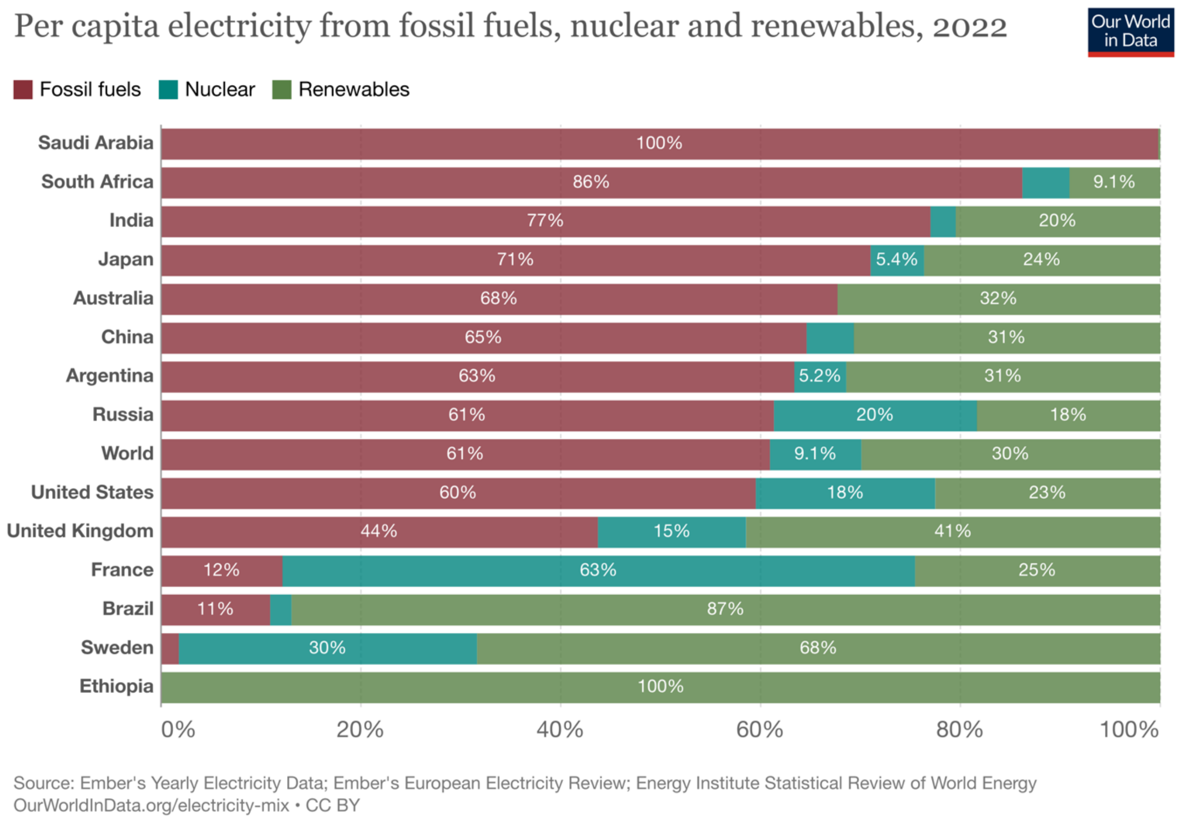

Figure S10. Electricity production mix by selected countries
Source: Image taken from Our World in Data: Electricity Mix webpage: Country level proportional consumption of electricity from fossil fuel production (red), nuclear production (teal), and renewables (green).[103]

4.1. Modification of Existing Ecoinvent Electricity Datasets

Ecoinvent contains datasets for the production of high voltage (above 24 kilovolts (kV)), medium voltage (between 1 kV and 24 kV) and low voltage electricity (below 1 kV) in over 250 geographies.[3,101] These datasets are designed to reflect electricity supply and consumption including processes of electricity generation, transformation, transmission, distribution and use.[101] The types of electricity generation covered by Ecoinvent include fossil-based production through coal, natural gas, oil and peat; renewable production via solar, hydro, wind and geothermal generation; and production using biogas and waste incineration.[101]

Geography-specific market electricity datasets describe the proportions of different methods of electricity production used within countries, aggregated into regions and eventually into a global scale dataset.[3] The global dataset therefore describes, for each kWh of high-, medium- or low-voltage electricity produced on a global scale, the proportions of each country dataset responsible.[3] Using the global-level datasets as a starting point, we separated the countries contained into groups of HIC, UMC, LMC and LIC according to World Bank Country Income Classifications[52], in order to reflect the P_2O geographical archetypes. Using each country's proportional contribution to global electricity production, we then scaled contributions within each income classification to produce 1kWh of electricity. This approach therefore maintained the original Ecoinvent assumptions on

countries' relative contributions to global electricity production whilst creating sub-groups to describe regional electricity mixes by country income classification[52] (**Table S30**). Country-level datasets were updated within the ecoinvent v.310 release.

Table S30. Ecoinvent datasets for national electricity production mix grouped by country income classification[52]

**World Bank Country Income Classification[52]**						
High-Income Countries		Upper Middle-Income Countries		Lower Middle-Income Countries		Low-Income Countries
Australia	Latvia	Albania	Moldova	Algeria	Ukraine	Congo, The Democratic Republic of
Austria	Lithuania	Argentina	Montenegro	Angola	Uzbekistan	Eritrea
Bahrain	Luxembourg	Armenia	Namibia	Bangladesh	Vietnam	Ethiopia
Belgium	Malta	Azerbaijan	Paraguay	Benin	Zimbabwe	Korea, Democratic People's Republic Of
Brunei Darussalam	Netherlands	Belarus	Peru	Bolivia		Mozambique
Canada	New Zealand	Bosnia and Herzegovina	Russia	Cambodia		Niger
Canada	Norway	Botswana	Serbia	Cameroon		South Sudan
Chile	Oman	Brazil	South Africa	Congo		Sudan
Croatia	Panama	Bulgaria	Thailand	Cote d'Ivoire		Syrian Arab Republic
Curaçao	Poland	China	Turkey	Egypt		Togo
Cyprus	Portugal	Colombia	Turkmenistan	El Salvador		Yemen
Czech Republic	Puerto Rico	Costa Rica	Venezuela	Ghana		Zambia
Denmark	Qatar	Cuba	Mongolia	Haiti		
Estonia	Romania	Dominican Republic	Morocco	Honduras		
Finland	Saudi Arabia	Ecuador	Myanmar	India		
France	Singapore	Gabon	Nepal	Indonesia		
Germany	Slovakia	Georgia	Nicaragua	Iran		
Gibraltar	Slovenia	Guatemala		Kenya		
Greece	Spain	Iraq		Kyrgyzstan		
Hong Kong	Sweden	Jamaica		Lebanon		
Hungary	Switzerland	Jordan		Nigeria		
Iceland	Taiwan, Province Of China	Kazakhstan		Pakistan		
Ireland	Trinidad and Tobago	Kosovo		Philippines		
Israel	UK	Libyan Arab		Senegal		
Italy	United Arab Emirates	Macedonia		Sri Lanka		
Japan	United States	Malaysia		Tajikistan		
Korea, Republic Of	Uruguay	Mauritius		Tanzania		
Kuwait		Mexico		Tunisia		

Notes: Groupings based on World Bank Country Income Classifications[52]

4.2. Regionalised Electricity Mix Impact Comparison

The Human Health impacts differed by the estimated regional electricity mixes for HIC, UC, LMC and LIC per 1kWh of high-, medium-, and low-voltage electricity. Electricity production in LIC mostly had the lowest impacts, whereas the greatest impacts on health were found for the LMC production mixes (**Figure S11** and **Table S31**)

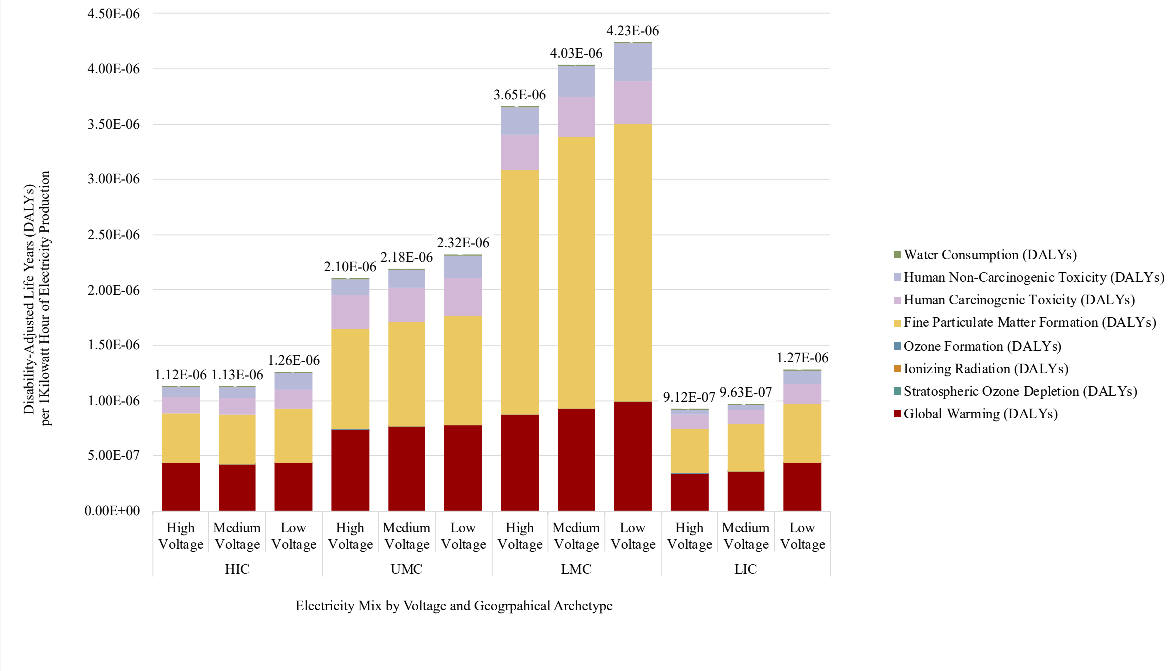

Figure S11. Human Health impacts in Disability-Adjusted Life Years (DALYs) of estimated regional electricity production mixes based on Plastics-to-Ocean (P_2O) Geographical Archetypes
Estimated Human health impacts in Disability-Adjusted Life Years per 1 kilowatt hour (kWh) of high (red), medium (orange) and low (yellow) voltage electricity produced according to the regional production mix estimated for geographical archetypes of the Plastics-to-Ocean (P_2O) model: High-Income Countries Archetype (HIC), Upper Middle-Income Countries Archetype (UMC), Lower Middle-Income Countries Archetype (LMC) and Low-Income Countries Archetype (LIC) archetypes.

Table S31. Human Health impacts of estimated regional electricity production mixes in Disability-Adjusted Life Years (DALYs)

	**Geographic Archetype Electricity Mix**											
	HIC			UMC			LMC			LIC		
Recipe 2016 Impact Category[5]	High Voltage	Medium Voltage	Low Voltage	High Voltage	Medium Voltage	Low Voltage	High Voltage	Medium Voltage	Low Voltage	High Voltage	Medium Voltage	Low Voltage
Global Warming	4.30E-07	4.19E-07	4.34E-07	7.37E-07	7.60E-07	7.73E-07	8.73E-07	9.23E-07	9.86E-07	3.41E-07	3.57E-07	4.28E-07
Stratospheric Ozone Depletion	9.61E-11	9.31E-11	9.75E-11	1.75E-10	1.76E-10	1.90E-10	1.33E-10	1.39E-10	1.52E-10	1.14E-10	1.18E-10	1.42E-10
Ionizing Radiation	1.20E-09	1.25E-09	1.20E-09	3.88E-10	4.02E-10	4.13E-10	3.26E-10	3.55E-10	3.57E-10	2.08E-11	2.18E-11	2.94E-11
Ozone Depletion	6.89E-10	6.59E-10	7.22E-10	1.89E-09	1.95E-09	1.99E-09	1.88E-09	2.04E-09	2.14E-09	9.57E-10	9.92E-10	1.21E-09
Fine Particulate Matter Formation	4.54E-07	4.56E-07	4.95E-07	9.11E-07	9.45E-07	9.88E-07	2.21E-06	2.46E-06	2.51E-06	4.06E-07	4.24E-07	5.41E-07
Human Carcinogenic Toxicity	1.45E-07	1.50E-07	1.70E-07	3.04E-07	3.18E-07	3.39E-07	3.23E-07	3.59E-07	3.87E-07	1.25E-07	1.33E-07	1.77E-07
Human Non-Carcinogenic Toxicity	8.51E-08	9.52E-08	1.50E-07	1.42E-07	1.54E-07	2.12E-07	2.44E-07	2.81E-07	3.43E-07	3.94E-08	4.80E-08	1.19E-07
Water Consumption	3.90E-09	3.97E-09	4.22E-09	2.94E-09	3.06E-09	3.33E-09	2.10E-09	1.94E-09	2.67E-09	6.48E-10	6.94E-10	1.03E-09
Total DALYs	1.12E-06	1.13E-06	1.26E-06	2.10E-06	2.18E-06	2.32E-06	3.65E-06	4.03E-06	4.23E-06	9.12E-07	9.63E-07	1.27E-06

Notes: Impact assessment conducted per kilowatt hour (kWh) of electricity produced based on Ecoinvent global electricity mix[3], with country-level datasets regrouped according to World Bank Country Income Classifications.[52] Impact assessment conducted using ReCiPe 2016 Hierarchic perspective impact assessment method.[5] Abbreviations: Kilowatt hour (kWh), Disability-Adjusted Life Years (DALYs), High-Income Countries Archetype (HIC), Upper Middle-Income Countries Archetype (UMC), Lower Middle-Income Countries Archetype (LMC), Low-Income Countries Archetype (LIC).

4.3. Modification of Plastics-to-Ocean (P_2O) Processes with Regionalised Electricity Mixes

The aim of adapting the datasets with regionalised electricity mixes was to increase the precision of process impact estimates, for different P_2O geographical archetypes and subsequently the combined global impacts. Our approach was to modify only the first order electricity inputs into each process (i.e. the dominating electricity inputs), leaving the electricity inputs of sub-processes as existing in Ecoinvent. Though this could be part of future analyses, the assumptions required were beyond the scope of current modelling and may in fact be further from the reality of electricity production and consumption as an internationally traded resource.[104]

Where possible, if modifications were made for electricity, we aimed to also apportion the geographical datasets by P_2O archetypes for consistency. However, we also wanted to maintain the overarching Ecoinvent assumptions of geographical contributions to global market processes, therefore some compromises were made. Where possible, the approach was to redistribute available Ecoinvent geography-specific datasets according to P_2O categories, replacing the *Rest of the World* dataset with a copy with modified electricity inputs for HIC, UMC, LMC and LIC. However if this approach were to subsequently alter total geographical contributions as assumed in Ecoinvent, we kept the existing distribution for each P_2O archetype and modified geographies for relevant P_2O archetype electricity inputs. The specific approach taken for each process is detailed in **Table S32**.

Regionalised datasets for specific processes and polymers were combined according to the same proportions described in **Figure S1** to create 1 Mt of each of the P_2O plastic categories. In doing so, separate inventories were created for each of the plastic categories (Rigid Monomaterials, Flexible Monomaterials and Multilayer/Multimaterials) in each of the geographical archetypes (HIC, UMC, LMC, LIC) and for each of the life cycle processes (where electricity was a first order input).

Table S32. Regionalisation modifications for each Plastics-to-Ocean (P_2O) life cycle process

Life Cycle Process	Regionalisation by input	Method of Regionalisation
Production		
Virgin Plastic Production	Not regionalised by electricity	• Production of polymers requires electricity but we maintained Ecoinvent global market dataset assumptions to reflect the international production market and trade that supplies the plastic demand indicated for each of the P_2O geographical archetypes
	No other regionalisation	• N/A
Collecting and Sorting		
Transport	No electricity	• N/A
	No other regionalisation	• N/A
Sorting	Regionalised by electricity	• Sorting process: geographical redistribution and modified electricity inputs for each polymer<br>⇒ HIC: *Switzerland*, *Europe*, *United States of America*, *Rest of the World* (modified for HIC electricity mix)<br>⇒ UMC: *Rest of the World* (modified with UMC electricity mix)<br>⇒ LMC: *Rest of the World* (modified with LMC electricity mix)<br>⇒ LIC: *Rest of the World* (modified with LIC electricity mix)
	No other regionalisation	• N/A
Recycling		
Mechanical Recycling and Recycled Granulate Production	Regionalised by electricity	• Recycling process: geographical redistribution and modified electricity inputs for each polymer<br>⇒ HIC: *Switzerland*, *Europe*, *United States of America*, *Rest of the World* (modified for HIC electricity mix)<br>⇒ UMC: *Rest of the World* (modified with UMC electricity mix)<br>⇒ LMC: *Rest of the World* (modified with LMC electricity mix)<br>⇒ LIC: *Rest of the World* (modified with LIC electricity mix)
	No other regionalisation	• N/A
Chemical Recycling and Monomer Production	Regionalised by electricity	• Pyrolysis process: modified electricity input of geographically generic dataset to create new datasets with HIC, UMC, LMC and LIC electricity mixes

Life Cycle Process	Regionalisation by input	Method of Regionalisation
		• Sorting process: geographical redistribution and modified electricity inputs for each polymer<br>⇒ HIC: *Switzerland*, *Europe*, *United States of America*, *Rest of the World* (modified for HIC electricity mix)<br>⇒ UMC: *Rest of the World* (modified with UMC electricity mix)<br>⇒ LMC: *Rest of the World* (modified with LMC electricity mix)<br>⇒ LIC: *Rest of the World* (modified with LIC electricity mix)<br>• Polymerisation process for recycled granulate production: modified all geographies with regionalised electricity inputs<br>⇒ HIC: *Europe* and *Rest of the World* (modified for HIC electricity mix)<br>⇒ UMC: *Europe* and *Rest of the World* (modified with UMC electricity mix)<br>⇒ LMC: *Europe* and *Rest of the World* (modified with LMC electricity mix)<br>⇒ LIC: *Europe* and *Rest of the World* (modified with LIC electricity mix)
	Geographical regionalisation of transport from sorting to recycling*	• Transport from sorting to recycling:<br>⇒ HIC: *Switzerland*, *Europe without Switzerland*, *Rest of the World* and *United States of America* (same as mechanical recycling)<br>⇒ UMC, LMC, LIC: *Rest of the World* transport only*<br><br>**This differs from mechanical recycling transportation: in P_2O transportation of source separated plastic for mechanical recycling is linked through international trade and therefore considered global datasets for these transportation processes, for chemical recycling the transportation is not linked with international trade and so we can regionalise the transport to the extent possible with Ecoinvent datasets*
Waste Management		
Incineration	No electricity	• N/A
	No other regionalisation	• N/A
Sanitary Landfill	Regionalised by electricity	• Landfill process: geographical redistribution and modified electricity inputs for each polymer<br>⇒ HIC: *Switzerland* and *Rest of the World* (modified for HIC electricity mix)<br>⇒ UMC: *Rest of the World* (modified with UMC electricity mix)<br>⇒ LMC: *Rest of the World* (modified with LMC electricity mix)<br>⇒ LIC: *Rest of the World* (modified with LIC electricity mix)
	No other regionalisation	• N/A
Open Dumpsites and Unsanitary Landfill	No electricity	• N/A
	Regionalised by climatic conditions	• Detailed in **Section**: **2.4.2. Open Dumpsites and Unsanitary Landfill**
Open Burning	No electricity	• N/A
	No other regionalisation	• N/A
Terrestrial Pollution	No electricity	• N/A
	Regionalised by climatic conditions	• Detailed in **Section**: **2.4.4. Terrestrial Pollution**
Aquatic Pollution	No electricity	• N/A
	No other regionalisation	• N/A
Substitutions		
Single-Use Paper and Coated Paper	Regionalised by electricity in multiple stages	• Virgin pulp production and plastic coating granulate production: *Global* (no regionalisation as based on demand rather than production)<br>• Deinked wet lap pulp production (Recycling)<br>⇒ Redistribution of geographical databases (*Quebec* and *Rest of the World*) and replacement of *Rest of the World* electricity inputs with modified inputs for HIC, UMC, LMC, LIC whilst maintaining Ecoinvent overall geographical proportions<br>⇒ Sorting available for *Switzerland, Europe without Switzerland* and *Rest of the World*, unable to apportion geographically for P_2O without changing the overall Ecoinvent proportions, replaced *Rest*

Life Cycle Process	Regionalisation by input	Method of Regionalisation
		of the World electricity with relevant mix for each geographical archetype<br>• Non-recycling market for paper and coating waste: Regionalised by categorising geography-specific datasets by Country Income Classification whilst maintaining Ecoinvent overall proportions for total of archetypes
Single-Use Compostables (Polylactide)	No electricity regionalisation	• Virgin PLA production: *Global*<br>• Transport: *Global*<br>• End of life Incineration:<br>⇒ HIC: *Switzerland* and *Rest of the World* (according to national contribution to global plastic waste generation data)[41,42]<br>⇒ UMC: *Rest of the World*<br>⇒ LMC: *Rest of the World*<br>⇒ LIC: *Rest of the World*
	No other regionalisation	• N/A
Reusable Glass System – Household	Regionalised by electricity for washing and waste treatment	• Glass production and recycling: *Global*<br>• Glass washing: *Global* electricity input for dishwashing and heating water for handwashing modified for HIC, UMC, LMC and LIC<br>• Glass market waste disposal: by geography: country-level Ecoinvent datasets grouped by income classification, proportions based on national contribution to global glass waste
	Regionalised substitution rate	• Detailed in **Section 3.5.3. Substitution Rates**<br>• Based on different proportions of P_2O products in HIC vs LMIC archetypes, glass substitution determined on the basis of providing equivalent packaging service during one year
	Regionalised by dishwashing vs handwashing practices	• Detailed in **Section 3.5.4. Consumer Reuse**<br>⇒ HIC: 45% dishwasher use versus 55% handwashing[96]<br>⇒ UMC and LMC: 10% dishwasher use versus 90% handwashing[98,99]<br>⇒ LIC: 0% dishwasher use, 100% handwashing[98,99]
Reusable Glass System – New Delivery Model	Regionalised by electricity for washing and waste treatment	• Glass production and recycling: *Global*<br>• Glass Collection and Delivery: *Global*<br>• Glass washing: *Global* electricity input for dishwashing modified for HIC, UMC, LMC and LIC<br>• Glass market waste disposal: by geography: country-level Ecoinvent datasets grouped by income classification, proportions based on national contribution to global glass waste
	Regionalised substitution rate	• Detailed in **Section 3.5.3. Substitution Rates**<br>⇒ Based on different proportions of P_2O products in HIC vs LMIC archetypes, glass substitution determined on the basis of providing equivalent packaging service during one year

Notes: Abbreviations: High-Income Countries Archetype (HIC) and in Upper Middle-Income Countries Archetype (UMC), Lower Middle-Income Countries Archetype (UMC), Low-Income Countries Archetype (LIC).

5. Results: Life Cycle Impact Assessment

Table S33. Total Disability-Adjusted Life Years (DALYs) associated with 1 Mt of each plastic category by life cycle process and health-related midpoint impacts

	ReCiPe 2016 Hierarchic perspective midpoint and endpoint Human Health impacts								
**Plastic Life Cycle Stages and Plastic Substitute Systems by Polymer Mix and Geographical Archetype**	**Global Warming**	**Stratospheric Ozone Depletion**	**Ionizing Radiation**	**Ozone Formation**	**Fine Particulate Matter Formation**	**Human Carcinogenic Toxicity**	**Human Non-Carcinogenic Toxicity**	**Water Consumption**	**Total Human Health Impacts**
	(DALYs per 1Mtn plastic)	(DALYs per 1Mtn plastic)	(DALYs per 1Mtn plastic)	(DALYs per 1Mtn plastic)	(DALYs per 1Mtn plastic)	(DALYs per 1Mtn plastic)	(DALYs per 1Mtn plastic)	(DALYs per 1Mtn plastic)	(DALYs per 1Mtn plastic)
Virgin Plastic Production: RIGIDMONO (GLO)	3.24E+03	3.70E+00	1.09E+00	6.85E+00	2.55E+03	2.11E+03	7.30E+02	4.28E+01	8.69E+03
Virgin Plastic Production: FLEXMONO (GLO)	3.03E+03	5.72E-01	1.15E+00	6.81E+00	2.37E+03	1.94E+03	6.42E+02	5.24E+01	8.05E+03
Virgin Plastic Production: HICMULTI (GLO)	3.13E+03	4.06E-01	9.70E-01	6.58E+00	2.33E+03	1.84E+03	6.11E+02	5.56E+01	7.98E+03
Virgin Plastic Production: LMICMULTI (GLO)	3.08E+03	6.17E-01	1.12E+00	6.79E+00	2.39E+03	1.91E+03	6.39E+02	5.38E+01	8.08E+03
Mechanical Plastic Recycling: RIGIDMONO (HIC)	2.40E+02	5.71E-02	6.75E-01	4.37E-01	2.89E+02	1.28E+02	9.06E+01	2.76E+00	7.50E+02
Mechanical Plastic Recycling: RIGIDMONO (UMC)	4.36E+02	1.03E-01	1.98E-01	1.07E+00	5.45E+02	2.06E+02	1.18E+02	2.14E+00	1.31E+03
Mechanical Plastic Recycling: RIGIDMONO (LMC)	5.31E+02	8.56E-02	1.73E-01	1.13E+00	1.22E+03	2.28E+02	1.76E+02	1.85E+00	2.16E+03
Mechanical Plastic Recycling: RIGIDMONO (LIC)	3.19E+02	8.18E-02	4.85E-02	7.80E-01	4.74E+02	1.48E+02	9.09E+01	1.23E+00	1.03E+03
Mechanical Plastic Recycling: FLEXMONO (HIC)	1.84E+02	5.28E-02	7.14E-01	3.08E-01	2.20E+02	1.13E+02	8.11E+01	2.50E+00	6.01E+02
Mechanical Plastic Recycling: FLEXMONO (UMC)	3.87E+02	1.00E-01	1.95E-01	9.61E-01	4.86E+02	1.93E+02	1.08E+02	1.83E+00	1.18E+03
Mechanical Plastic Recycling: FLEXMONO (LMC)	4.86E+02	8.26E-02	1.69E-01	1.03E+00	1.19E+03	2.15E+02	1.69E+02	1.52E+00	2.06E+03
Mechanical Plastic Recycling: FLEXMONO (LIC)	2.31E+02	7.81E-02	1.94E-02	6.05E-01	2.87E+02	1.19E+02	6.60E+01	7.77E-01	7.04E+02
Mechanical Plastic Recycling: MULTI (HIC)	1.81E+02	5.27E-02	7.18E-01	3.01E-01	2.16E+02	1.12E+02	8.07E+01	2.49E+00	5.94E+02
Mechanical Plastic Recycling: MULTI (UMC)	3.93E+02	1.01E-01	1.98E-01	9.72E-01	4.90E+02	1.95E+02	1.09E+02	1.85E+00	1.19E+03
Mechanical Plastic Recycling: MULTI (LMC)	4.93E+02	8.35E-02	1.72E-01	1.04E+00	1.20E+03	2.18E+02	1.71E+02	1.54E+00	2.09E+03
Mechanical Plastic Recycling: MULTI (LIC)	2.32E+02	7.89E-02	1.84E-02	6.07E-01	2.80E+02	1.19E+02	6.55E+01	7.73E-01	6.98E+02

	ReCiPe 2016 Hierarchic perspective midpoint and endpoint Human Health impacts								
**Plastic Life Cycle Stages and Plastic Substitute Systems by Polymer Mix and Geographical Archetype**	**Global Warming**	**Stratospheric Ozone Depletion**	**Ionizing Radiation**	**Ozone Formation**	**Fine Particulate Matter Formation**	**Human Carcinogenic Toxicity**	**Human Non-Carcinogenic Toxicity**	**Water Consumption**	**Total Human Health Impacts**
	(DALYs per 1Mtn plastic)	(DALYs per 1Mtn plastic)	(DALYs per 1Mtn plastic)	(DALYs per 1Mtn plastic)	(DALYs per 1Mtn plastic)	(DALYs per 1Mtn plastic)	(DALYs per 1Mtn plastic)	(DALYs per 1Mtn plastic)	(DALYs per 1Mtn plastic)
Avoided Plastic Production (Secondary Plastics): RIGIDMONO (ALL)	-3.24E+03	-3.70E+00	-1.09E+00	-6.85E+00	-2.55E+03	-2.11E+03	-7.30E+02	-4.28E+01	-8.69E+03
Avoided Plastic Production (Secondary Plastics): FLEXMONO (ALL)	-3.03E+03	-5.72E-01	-1.15E+00	-6.81E+00	-2.37E+03	-1.94E+03	-6.42E+02	-5.24E+01	-8.05E+03
Avoided Plastic Production (Secondary Plastics): HICMULTI (ALL)	-3.13E+03	-4.06E-01	-9.70E-01	-6.58E+00	-2.33E+03	-1.84E+03	-6.11E+02	-5.56E+01	-7.98E+03
Avoided Plastic Production (Secondary Plastics): LMICMULTI (ALL)	-3.08E+03	-6.17E-01	-1.12E+00	-6.79E+00	-2.39E+03	-1.91E+03	-6.39E+02	-5.38E+01	-8.08E+03
Chemical Plastic Recycling: Pyrolysis: ALL (HIC)	1.12E+03	1.42E-01	1.64E+00	1.28E+00	7.15E+02	6.37E+02	1.46E+02	5.86E+00	2.63E+03
Chemical Plastic Recycling: Pyrolysis: ALL (UMC)	1.54E+03	2.48E-01	5.38E-01	2.91E+00	1.33E+03	8.53E+02	2.23E+02	4.57E+00	3.95E+03
Chemical Plastic Recycling: Pyrolysis: ALL (LMC)	1.72E+03	1.92E-01	4.55E-01	2.90E+00	3.09E+03	8.79E+02	3.61E+02	3.44E+00	6.06E+03
Chemical Plastic Recycling: Pyrolysis: ALL (LIC)	1.00E+03	1.67E-01	4.23E-02	1.65E+00	6.52E+02	6.11E+02	8.47E+01	1.48E+00	2.35E+03
Chemical Plastic Recycling: Polymerisation: ALL (HIC)	5.59E+02	1.16E-01	1.28E+00	1.48E+00	6.31E+02	4.91E+02	2.30E+02	4.20E+01	1.96E+03
Chemical Plastic Recycling: Polymerisation: ALL (UMC)	8.83E+02	1.94E-01	4.67E-01	2.71E+00	1.10E+03	6.51E+02	2.87E+02	4.12E+01	2.96E+03
Chemical Plastic Recycling: Polymerisation: ALL (LMC)	1.04E+03	1.59E-01	4.22E-01	2.79E+00	2.54E+03	6.90E+02	4.07E+02	4.01E+01	4.72E+03
Chemical Plastic Recycling: Polymerisation: ALL (LIC)	4.99E+02	1.40E-01	1.05E-01	1.80E+00	6.00E+02	4.75E+02	1.86E+02	3.89E+01	1.80E+03
Avoided Fuel Production Burdens: ALL (ALL)	-2.06E+03	-1.94E-01	-3.54E-01	-4.01E+00	-1.42E+03	-1.14E+03	-3.30E+02	-1.62E+01	-4.97E+03
Transport for Municipal Recycling Collection: ALL (ALL)	6.44E+01	8.93E-03	6.80E-03	2.64E-01	4.83E+01	4.14E+01	1.28E+01	2.09E-01	1.67E+02
Transport for Municipal Mixed Waste Collection: ALL (ALL)	5.61E+00	8.88E-04	6.95E-04	2.75E-02	4.68E+00	4.16E+00	1.12E+00	1.70E-02	1.56E+01
Industrial Plastic Waste Facility Sorting: ALL (HIC)	4.29E+01	5.43E-03	2.52E-02	1.48E-01	4.93E+01	3.84E+02	1.62E+01	4.51E-01	4.93E+02

	ReCiPe 2016 Hierarchic perspective midpoint and endpoint Human Health impacts								
**Plastic Life Cycle Stages and Plastic Substitute Systems by Polymer Mix and Geographical Archetype**	**Global Warming**	**Stratospheric Ozone Depletion**	**Ionizing Radiation**	**Ozone Formation**	**Fine Particulate Matter Formation**	**Human Carcinogenic Toxicity**	**Human Non-Carcinogenic Toxicity**	**Water Consumption**	**Total Human Health Impacts**
	(DALYs per 1Mtn plastic)	(DALYs per 1Mtn plastic)	(DALYs per 1Mtn plastic)	(DALYs per 1Mtn plastic)	(DALYs per 1Mtn plastic)	(DALYs per 1Mtn plastic)	(DALYs per 1Mtn plastic)	(DALYs per 1Mtn plastic)	(DALYs per 1Mtn plastic)
Industrial Plastic Waste Facility Sorting: ALL (UMC)	4.77E+01	6.71E-03	1.22E-02	1.66E-01	5.57E+01	3.87E+02	1.70E+01	4.36E-01	5.08E+02
Industrial Plastic Waste Facility Sorting: ALL (LMC)	5.04E+01	6.23E-03	1.15E-02	1.68E-01	7.50E+01	3.87E+02	1.87E+01	4.27E-01	5.32E+02
Industrial Plastic Waste Facility Sorting: ALL (LIC)	4.33E+01	6.10E-03	7.33E-03	1.56E-01	5.00E+01	3.85E+02	1.58E+01	4.07E-01	4.94E+02
Transport from Plastic Sorting to Recycling: ALL (ALL)	3.45E+01	4.79E-03	3.64E-03	1.42E-01	2.59E+01	2.21E+01	6.88E+00	1.12E-01	8.97E+01
Transport of International Plastic Waste Trade: ALL (ALL)	1.34E+02	3.70E-02	5.75E-03	2.51E+00	5.42E+02	7.56E+01	4.72E+00	1.54E-01	7.59E+02
Transport of Industrial Plastic Waste Management Process Losses: ALL (ALL)	5.61E+00	8.88E-04	6.95E-04	2.75E-02	4.68E+00	4.16E+00	1.12E+00	1.70E-02	1.56E+01
Plastic Incineration: RIGIDMONO (GLO)	2.40E+03	2.29E-01	3.03E-03	4.18E-01	4.32E+01	8.75E+01	3.19E+02	6.98E-01	2.85E+03
Plastic Incineration: FLEXMONO (GLO)	2.78E+03	7.45E-02	3.66E-03	4.30E-01	4.49E+01	9.92E+01	3.90E+02	8.76E-01	3.32E+03
Plastic Incineration: HICMULTI (GLO)	2.67E+03	8.06E-02	7.07E-03	4.31E-01	5.20E+01	1.29E+02	3.88E+02	2.01E+00	3.24E+03
Plastic Incineration: LMICMULTI (GLO)	2.74E+03	8.05E-02	4.98E-03	4.31E-01	4.77E+01	1.10E+02	3.86E+02	1.31E+00	3.28E+03
Plastic Sanitary Landfill: RIGIDMONO (HIC)	9.42E+01	3.82E-03	1.88E-03	7.41E-02	1.42E+01	1.07E+01	5.51E+02	5.28E-01	6.71E+02
Plastic Sanitary Landfill: RIGIDMONO (UMC)	9.42E+01	3.82E-03	1.86E-03	7.42E-02	1.42E+01	1.07E+01	5.51E+02	5.34E-01	6.71E+02
Plastic Sanitary Landfill: RIGIDMONO (LMC)	9.42E+01	3.82E-03	1.85E-03	7.42E-02	1.42E+01	1.07E+01	5.51E+02	5.33E-01	6.71E+02
Plastic Sanitary Landfill: RIGIDMONO (LIC)	9.42E+01	3.82E-03	1.85E-03	7.42E-02	1.42E+01	1.07E+01	5.51E+02	5.33E-01	6.71E+02
Plastic Sanitary Landfill: FLEXMONO (HIC)	1.08E+02	2.55E-03	1.83E-03	7.38E-02	1.42E+01	1.08E+01	7.23E+02	5.28E-01	8.56E+02
Plastic Sanitary Landfill: FLEXMONO (UMC)	1.08E+02	2.55E-03	1.81E-03	7.39E-02	1.42E+01	1.08E+01	7.23E+02	5.33E-01	8.57E+02
Plastic Sanitary Landfill: FLEXMONO (LMC)	1.08E+02	2.55E-03	1.81E-03	7.39E-02	1.42E+01	1.08E+01	7.23E+02	5.33E-01	8.57E+02

	ReCiPe 2016 Hierarchic perspective midpoint and endpoint Human Health impacts								
**Plastic Life Cycle Stages and Plastic Substitute Systems by Polymer Mix and Geographical Archetype**	**Global Warming**	**Stratospheric Ozone Depletion**	**Ionizing Radiation**	**Ozone Formation**	**Fine Particulate Matter Formation**	**Human Carcinogenic Toxicity**	**Human Non-Carcinogenic Toxicity**	**Water Consumption**	**Total Human Health Impacts**
	(DALYs per 1Mtn plastic)	(DALYs per 1Mtn plastic)	(DALYs per 1Mtn plastic)	(DALYs per 1Mtn plastic)	(DALYs per 1Mtn plastic)	(DALYs per 1Mtn plastic)	(DALYs per 1Mtn plastic)	(DALYs per 1Mtn plastic)	(DALYs per 1Mtn plastic)
Plastic Sanitary Landfill: FLEXMONO (LIC)	1.08E+02	2.55E-03	1.81E-03	7.39E-02	1.42E+01	1.08E+01	7.23E+02	5.33E-01	8.57E+02
Plastic Sanitary Landfill: MULTI (HIC)	1.03E+02	2.61E-03	1.83E-03	7.38E-02	1.41E+01	1.13E+01	7.73E+02	5.28E-01	9.02E+02
Plastic Sanitary Landfill: MULTI (UMC)	1.06E+02	2.61E-03	1.81E-03	7.39E-02	1.42E+01	1.09E+01	7.27E+02	5.33E-01	8.58E+02
Plastic Sanitary Landfill: MULTI (LMC)	1.06E+02	2.61E-03	1.81E-03	7.39E-02	1.42E+01	1.09E+01	7.27E+02	5.33E-01	8.58E+02
Plastic Sanitary Landfill: MULTI (LIC)	1.06E+02	2.61E-03	1.81E-03	7.39E-02	1.42E+01	1.09E+01	7.27E+02	5.33E-01	8.58E+02
Plastic Open Burning: RIGIDMONO (GLO)	2.49E+03	6.79E-01	0.00E+00	3.89E+00	2.18E+03	1.68E+02	1.05E+02	0.00E+00	4.94E+03
Plastic Open Burning: FLEXMONO (GLO)	2.88E+03	6.79E-01	0.00E+00	1.93E+00	2.79E+03	2.27E+02	1.28E+02	0.00E+00	6.02E+03
Plastic Open Burning: HICMULTI (GLO)	2.75E+03	6.79E-01	0.00E+00	1.97E+00	8.19E+03	7.12E+02	1.18E+02	0.00E+00	1.18E+04
Plastic Open Burning: LMICMULTI (GLO)	2.82E+03	6.79E-01	0.00E+00	1.99E+00	4.88E+03	4.14E+02	1.24E+02	0.00E+00	8.24E+03
Plastic Unsanitary Landfill: RIGIDMONO (HIC)	1.51E+02	8.80E-04	1.91E-04	6.31E-02	2.72E+02	3.96E+00	5.54E+02	4.33E-03	9.82E+02
Plastic Unsanitary Landfill: RIGIDMONO (UMC)	1.41E+02	6.45E-04	1.40E-04	5.24E-02	2.94E+02	3.84E+00	5.66E+02	3.18E-03	1.01E+03
Plastic Unsanitary Landfill: RIGIDMONO (LMC)	1.30E+02	3.93E-04	8.53E-05	9.90E-02	1.22E+03	2.61E+00	5.08E+02	1.94E-03	1.86E+03
Plastic Unsanitary Landfill: RIGIDMONO (LIC)	1.20E+02	1.77E-04	3.83E-05	1.88E-01	2.76E+03	2.58E+00	4.69E+02	8.71E-04	3.36E+03
Plastic Unsanitary Landfill: FLEXMONO (HIC)	1.76E+02	8.80E-04	1.91E-04	5.13E-02	2.79E+02	4.12E+00	7.26E+02	4.33E-03	1.18E+03
Plastic Unsanitary Landfill: FLEXMONO (UMC)	1.64E+02	6.45E-04	1.40E-04	3.95E-02	3.01E+02	4.07E+00	7.41E+02	3.18E-03	1.21E+03
Plastic Unsanitary Landfill: FLEXMONO (LMC)	1.51E+02	3.93E-04	8.53E-05	4.52E-02	1.25E+03	2.78E+00	6.67E+02	1.94E-03	2.07E+03
Plastic Unsanitary Landfill: FLEXMONO (LIC)	1.40E+02	1.77E-04	3.83E-05	6.54E-02	2.83E+03	2.89E+00	6.15E+02	8.71E-04	3.59E+03
Plastic Unsanitary Landfill: MULTI (HIC)	1.68E+02	8.80E-04	1.91E-04	5.15E-02	2.69E+02	4.70E+00	7.72E+02	4.33E-03	1.21E+03

	ReCiPe 2016 Hierarchic perspective midpoint and endpoint Human Health impacts								
**Plastic Life Cycle Stages and Plastic Substitute Systems by Polymer Mix and Geographical Archetype**	**Global Warming**	**Stratospheric Ozone Depletion**	**Ionizing Radiation**	**Ozone Formation**	**Fine Particulate Matter Formation**	**Human Carcinogenic Toxicity**	**Human Non-Carcinogenic Toxicity**	**Water Consumption**	**Total Human Health Impacts**
	(DALYs per 1Mtn plastic)	(DALYs per 1Mtn plastic)	(DALYs per 1Mtn plastic)	(DALYs per 1Mtn plastic)	(DALYs per 1Mtn plastic)	(DALYs per 1Mtn plastic)	(DALYs per 1Mtn plastic)	(DALYs per 1Mtn plastic)	(DALYs per 1Mtn plastic)
Plastic Unsanitary Landfill: MULTI (UMC)	1.61E+02	6.45E-04	1.40E-04	3.99E-02	2.98E+02	4.26E+00	7.42E+02	3.18E-03	1.20E+03
Plastic Unsanitary Landfill: MULTI (LMC)	1.48E+02	4.00E-04	8.68E-05	4.65E-02	1.21E+03	3.00E+00	6.70E+02	1.97E-03	2.03E+03
Plastic Unsanitary Landfill: MULTI (LIC)	1.37E+02	1.77E-04	3.83E-05	6.92E-02	2.80E+03	3.03E+00	6.16E+02	8.71E-04	3.56E+03
Plastic Terrestrial Pollution: RIGIDMONO (HIC)	1.11E+02	0.00E+00	0.00E+00	1.95E-02	2.66E+02	2.97E+00	5.46E+02	0.00E+00	9.25E+02
Plastic Terrestrial Pollution: RIGIDMONO (UMC)	1.11E+02	0.00E+00	0.00E+00	2.12E-02	2.89E+02	3.26E+00	5.59E+02	0.00E+00	9.63E+02
Plastic Terrestrial Pollution: RIGIDMONO (LMC)	1.11E+02	0.00E+00	0.00E+00	8.90E-02	1.21E+03	3.02E+00	5.14E+02	0.00E+00	1.84E+03
Plastic Terrestrial Pollution: RIGIDMONO (LIC)	1.11E+02	0.00E+00	0.00E+00	2.03E-01	2.77E+03	3.34E+00	4.76E+02	0.00E+00	3.36E+03
Plastic Terrestrial Pollution: FLEXMONO (HIC)	1.28E+02	0.00E+00	0.00E+00	9.17E-03	2.73E+02	3.07E+00	7.05E+02	0.00E+00	1.11E+03
Plastic Terrestrial Pollution: FLEXMONO (UMC)	1.28E+02	0.00E+00	0.00E+00	9.99E-03	2.97E+02	3.40E+00	7.20E+02	0.00E+00	1.15E+03
Plastic Terrestrial Pollution: FLEXMONO (LMC)	1.28E+02	0.00E+00	0.00E+00	4.19E-02	1.25E+03	3.17E+00	6.64E+02	0.00E+00	2.04E+03
Plastic Terrestrial Pollution: FLEXMONO (LIC)	1.28E+02	0.00E+00	0.00E+00	9.54E-02	2.84E+03	3.62E+00	6.13E+02	0.00E+00	3.59E+03
Plastic Terrestrial Pollution: MULTI (HIC)	1.22E+02	0.00E+00	0.00E+00	8.76E-03	2.63E+02	3.44E+00	7.58E+02	0.00E+00	1.15E+03
Plastic Terrestrial Pollution: MULTI (UMC)	1.26E+02	0.00E+00	0.00E+00	1.01E-02	2.93E+02	3.52E+00	7.24E+02	0.00E+00	1.15E+03
Plastic Terrestrial Pollution: MULTI (LMC)	1.26E+02	0.00E+00	0.00E+00	4.25E-02	1.23E+03	3.27E+00	6.68E+02	0.00E+00	2.03E+03
Plastic Terrestrial Pollution: MULTI (LIC)	1.26E+02	0.00E+00	0.00E+00	9.69E-02	2.81E+03	3.74E+00	6.15E+02	0.00E+00	3.55E+03
Plastic Aquatic Pollution: RIGIDMONO (GLO)	1.11E+02	0.00E+00	0.00E+00	3.66E-05	8.03E-02	2.69E+00	5.39E+02	0.00E+00	6.52E+02
Plastic Aquatic Pollution: FLEXMONO (GLO)	1.28E+02	0.00E+00	0.00E+00	1.72E-05	1.01E-01	2.73E+00	6.97E+02	0.00E+00	8.28E+02
Plastic Aquatic Pollution: HICMULTI (GLO)	1.22E+02	0.00E+00	0.00E+00	1.64E-05	1.10E-01	3.02E+00	7.54E+02	0.00E+00	8.80E+02

	ReCiPe 2016 Hierarchic perspective midpoint and endpoint Human Health impacts								
**Plastic Life Cycle Stages and Plastic Substitute Systems by Polymer Mix and Geographical Archetype**	**Global Warming**	**Stratospheric Ozone Depletion**	**Ionizing Radiation**	**Ozone Formation**	**Fine Particulate Matter Formation**	**Human Carcinogenic Toxicity**	**Human Non-Carcinogenic Toxicity**	**Water Consumption**	**Total Human Health Impacts**
	(DALYs per 1Mtn plastic)	(DALYs per 1Mtn plastic)	(DALYs per 1Mtn plastic)	(DALYs per 1Mtn plastic)	(DALYs per 1Mtn plastic)	(DALYs per 1Mtn plastic)	(DALYs per 1Mtn plastic)	(DALYs per 1Mtn plastic)	(DALYs per 1Mtn plastic)
Plastic Aquatic Pollution: LMICMULTI (GLO)	1.26E+02	0.00E+00	0.00E+00	1.75E-05	1.04E-01	2.81E+00	7.03E+02	0.00E+00	8.31E+02
Paper Substitute (with Recycling) ALL (HIC)	6.78E+02	4.58E-01	4.24E-01	1.53E+00	5.83E+02	2.94E+02	2.69E+02	3.42E+00	1.83E+03
Paper Substitute (with Recycling) ALL (UMC)	9.76E+02	4.51E-01	1.85E-01	1.93E+00	7.81E+02	3.32E+02	2.95E+02	3.51E+00	2.39E+03
Paper Substitute (with Recycling) ALL (LMC)	9.81E+02	4.52E-01	1.72E-01	2.04E+00	1.22E+03	3.44E+02	3.27E+02	3.19E+00	2.87E+03
Paper Substitute (with Recycling) ALL (LIC)	8.42E+02	4.39E-01	7.76E-02	1.67E+00	6.56E+02	2.80E+02	2.63E+02	2.84E+00	2.05E+03
Coated Paper Substitute (with Recycling) ALL (HIC)	6.88E+02	4.58E-01	4.26E-01	1.55E+00	5.90E+02	3.00E+02	2.72E+02	3.56E+00	1.86E+03
Coated Paper Substitute (with Recycling) ALL (UMC)	9.83E+02	4.51E-01	1.88E-01	1.94E+00	7.89E+02	3.37E+02	2.99E+02	3.65E+00	2.41E+03
Coated Paper Substitute (with Recycling) ALL (LMC)	9.89E+02	4.52E-01	1.74E-01	2.06E+00	1.23E+03	3.49E+02	3.31E+02	3.34E+00	2.90E+03
Coated Paper Substitute (with Recycling) ALL (LIC)	8.50E+02	4.39E-01	8.08E-02	1.69E+00	6.65E+02	2.85E+02	2.67E+02	2.99E+00	2.07E+03
Compostable Substitute (Plastic Incineration:) ALL (HIC)	3.01E+03	5.72E+00	1.53E+00	7.81E+00	3.32E+03	1.54E+03	1.09E+03	1.91E+02	9.16E+03
Compostable Substitute (Plastic Incineration:) ALL (UMC)	3.01E+03	5.72E+00	1.53E+00	7.81E+00	3.32E+03	1.54E+03	1.09E+03	1.91E+02	9.16E+03
Compostable Substitute (Plastic Incineration:) ALL (LMC)	3.01E+03	5.72E+00	1.53E+00	7.81E+00	3.32E+03	1.54E+03	1.09E+03	1.91E+02	9.16E+03
Compostable Substitute (Plastic Incineration:) ALL (LIC)	3.01E+03	5.72E+00	1.53E+00	7.81E+00	3.32E+03	1.54E+03	1.09E+03	1.91E+02	9.16E+03
Consumer Reuse Glass Substitute: ALL (HIC)	4.27E+02	1.82E-01	8.99E-01	7.78E-01	4.86E+02	3.49E+02	1.50E+02	4.11E+00	1.42E+03
Consumer Reuse Glass Substitute: ALL (UMC)	8.80E+02	3.15E-01	4.53E-01	2.22E+00	1.10E+03	5.97E+02	2.52E+02	3.65E+00	2.84E+03
Consumer Reuse Glass Substitute: ALL (LMC)	1.09E+03	2.79E-01	3.99E-01	2.36E+00	2.58E+03	6.44E+02	3.78E+02	3.01E+00	4.69E+03

	ReCiPe 2016 Hierarchic perspective midpoint and endpoint Human Health impacts								
**Plastic Life Cycle Stages and Plastic Substitute Systems by Polymer Mix and Geographical Archetype**	**Global Warming**	**Stratospheric Ozone Depletion**	**Ionizing Radiation**	**Ozone Formation**	**Fine Particulate Matter Formation**	**Human Carcinogenic Toxicity**	**Human Non-Carcinogenic Toxicity**	**Water Consumption**	**Total Human Health Impacts**
	(DALYs per 1Mtn plastic)	(DALYs per 1Mtn plastic)	(DALYs per 1Mtn plastic)	(DALYs per 1Mtn plastic)	(DALYs per 1Mtn plastic)	(DALYs per 1Mtn plastic)	(DALYs per 1Mtn plastic)	(DALYs per 1Mtn plastic)	(DALYs per 1Mtn plastic)
Consumer Reuse Glass Substitute: ALL (LIC)	5.77E+02	2.81E-01	8.62E-02	1.54E+00	7.07E+02	4.59E+02	1.69E+02	1.28E+00	1.91E+03
New Delivery Model Reuse Glass Substitute: ALL (HIC)	2.72E+02	4.88E-02	8.84E-02	9.33E-01	3.76E+02	1.28E+02	5.24E+01	2.06E+00	8.32E+02
New Delivery Model Reuse Glass Substitute: ALL (UMC)	2.14E+02	4.12E-02	6.60E-02	7.37E-01	3.18E+02	9.97E+01	4.11E+01	1.72E+00	6.76E+02
New Delivery Model Reuse Glass Substitute: ALL (LMC)	2.16E+02	4.40E-02	6.57E-02	7.42E-01	3.22E+02	9.94E+01	4.16E+01	1.72E+00	6.81E+02
New Delivery Model Reuse Glass Substitute: ALL (LIC)	2.13E+02	4.14E-02	6.44E-02	7.35E-01	3.22E+02	9.89E+01	4.08E+01	1.71E+00	6.77E+02

Notes: All impact assessment conducted using ReCiPe 2016 Hierarchic perspective impact assessment methodology.[5] Abbreviations: Rigid Monomaterials (RIGIDMONO), Flexible Monomaterials (FLEXMONO), Multilayers/Multimaterials in High-Income Countries Archetype (HICMULTI), Multilayers/Multimaterials in Low- and Middle-Income Countries Archetypes (LMICMULTI), all plastic categories (ALL), all geographical archetypes (GLO), High-Income Countries Archetype (HIC), Upper Middle-Income Countries Archetype (UMC), Lower Middle-Income Countries Archetype (UMC), Low-Income Countries Archetype (LIC).

Table S34. Total Disability-Adjusted Life Years (DALYs) associated with global system scenarios by geographical archetype, plastic category and year 2016-2040

SCENARIO NO.	SCENARIO DESCRIPTOR	2016	2017	2018	2019	2020	2021	2022	2023	2024	2025	2026	2027	2028	2029	2030	2031	2032	2033	2034	2035	2036	2037	2038	2039	2040
1	BAUHI_Ruralfl	3.71E+04	3.76E+04	3.77E+04	3.82E+04	3.85E+04	3.88E+04	3.91E+04	3.93E+04	3.95E+04	3.97E+04	3.99E+04	4.00E+04	4.02E+04	4.02E+04	4.03E+04	4.04E+04	4.03E+04	4.03E+04	4.03E+04	4.03E+04	4.02E+04	4.01E+04	4.00E+04	3.98E+04	3.96E+04
1	BAUHI_Ruralmu	3.88E+04	3.94E+04	3.97E+04	4.04E+04	4.09E+04	4.14E+04	4.18E+04	4.23E+04	4.27E+04	4.30E+04	4.34E+04	4.37E+04	4.40E+04	4.41E+04	4.46E+04	4.47E+04	4.49E+04	4.50E+04	4.52E+04	4.51E+04	4.52E+04	4.54E+04	4.54E+04	4.54E+04	4.54E+04
1	BAUHI_Ruralri	8.59E+04	8.64E+04	8.67E+04	8.71E+04	8.74E+04	8.75E+04	8.76E+04	8.77E+04	8.77E+04	8.76E+04	8.76E+04	8.74E+04	8.74E+04	8.71E+04	8.67E+04	8.64E+04	8.60E+04	8.56E+04	8.51E+04	8.45E+04	8.40E+04	8.33E+04	8.27E+04	8.20E+04	8.13E+04
**1**	**TOTAL HI RURAL BAU**	**1.62E+05**	**1.63E+05**	**1.64E+05**	**1.66E+05**	**1.67E+05**	**1.68E+05**	**1.69E+05**	**1.69E+05**	**1.70E+05**	**1.70E+05**	**1.71E+05**	**1.71E+05**	**1.72E+05**	**1.71E+05**	**1.72E+05**	**1.72E+05**	**1.71E+05**	**1.71E+05**	**1.71E+05**	**1.70E+05**	**1.69E+05**	**1.69E+05**	**1.68E+05**	**1.67E+05**	**1.66E+05**
1	BAUHI_Urbanfl	1.58E+05	1.61E+05	1.65E+05	1.68E+05	1.72E+05	1.75E+05	1.79E+05	1.82E+05	1.86E+05	1.89E+05	1.93E+05	1.97E+05	2.00E+05	2.04E+05	2.08E+05	2.11E+05	2.15E+05	2.18E+05	2.23E+05	2.26E+05	2.30E+05	2.34E+05	2.38E+05	2.41E+05	2.45E+05
1	BAUHI_Urbanmu	1.64E+05	1.68E+05	1.71E+05	1.77E+05	1.81E+05	1.85E+05	1.90E+05	1.94E+05	1.99E+05	2.03E+05	2.08E+05	2.13E+05	2.18E+05	2.22E+05	2.27E+05	2.32E+05	2.37E+05	2.42E+05	2.47E+05	2.53E+05	2.58E+05	2.63E+05	2.68E+05	2.73E+05	2.79E+05
1	BAUHI_Urbanri	3.75E+05	3.81E+05	3.89E+05	3.95E+05	4.01E+05	4.08E+05	4.14E+05	4.20E+05	4.26E+05	4.32E+05	4.38E+05	4.44E+05	4.49E+05	4.56E+05	4.61E+05	4.67E+05	4.73E+05	4.78E+05	4.84E+05	4.90E+05	4.95E+05	5.01E+05	5.06E+05	5.11E+05	5.16E+05
**1**	**TOTAL HI URBAN BAU**	**6.96E+05**	**7.11E+05**	**7.24E+05**	**7.40E+05**	**7.54E+05**	**7.69E+05**	**7.82E+05**	**7.96E+05**	**8.11E+05**	**8.25E+05**	**8.39E+05**	**8.53E+05**	**8.67E+05**	**8.82E+05**	**8.96E+05**	**9.10E+05**	**9.24E+05**	**9.39E+05**	**9.54E+05**	**9.69E+05**	**9.83E+05**	**9.97E+05**	**1.01E+06**	**1.03E+06**	**1.04E+06**
1	BAULI_Ruralfl	2.34E+04	2.49E+04	2.65E+04	2.81E+04	2.97E+04	3.13E+04	3.30E+04	3.47E+04	3.63E+04	3.80E+04	3.97E+04	4.14E+04	4.31E+04	4.49E+04	4.66E+04	4.83E+04	5.00E+04	5.18E+04	5.35E+04	5.52E+04	5.69E+04	5.86E+04	6.03E+04	6.20E+04	6.36E+04
1	BAULI_Ruralmu	1.30E+04	1.38E+04	1.47E+04	1.56E+04	1.65E+04	1.74E+04	1.83E+04	1.92E+04	2.02E+04	2.11E+04	2.20E+04	2.30E+04	2.39E+04	2.49E+04	2.58E+04	2.68E+04	2.77E+04	2.87E+04	2.96E+04	3.06E+04	3.15E+04	3.25E+04	3.34E+04	3.43E+04	3.52E+04
1	BAULI_Ruralri	1.73E+04	1.84E+04	1.95E+04	2.06E+04	2.17E+04	2.28E+04	2.40E+04	2.51E+04	2.62E+04	2.73E+04	2.85E+04	2.96E+04	3.07E+04	3.18E+04	3.29E+04	3.41E+04	3.52E+04	3.62E+04	3.73E+04	3.84E+04	3.95E+04	4.05E+04	4.15E+04	4.26E+04	4.36E+04
**1**	**TOTAL LI RURAL BAU**	**5.36E+04**	**5.71E+04**	**6.07E+04**	**6.42E+04**	**6.79E+04**	**7.15E+04**	**7.52E+04**	**7.90E+04**	**8.27E+04**	**8.65E+04**	**9.02E+04**	**9.40E+04**	**9.78E+04**	**1.02E+05**	**1.05E+05**	**1.09E+05**	**1.13E+05**	**1.17E+05**	**1.20E+05**	**1.24E+05**	**1.28E+05**	**1.32E+05**	**1.35E+05**	**1.39E+05**	**1.42E+05**
1	BAULI_Urbanfl	1.52E+04	1.69E+04	1.87E+04	2.06E+04	2.25E+04	2.46E+04	2.68E+04	2.91E+04	3.15E+04	3.41E+04	3.67E+04	3.95E+04	4.25E+04	4.55E+04	4.87E+04	5.20E+04	5.55E+04	5.91E+04	6.28E+04	6.67E+04	7.08E+04	7.50E+04	7.93E+04	8.38E+04	8.85E+04
1	BAULI_Urbanmu	1.15E+04	1.26E+04	1.36E+04	1.48E+04	1.60E+04	1.72E+04	1.86E+04	1.99E+04	2.14E+04	2.29E+04	2.45E+04	2.61E+04	2.78E+04	2.96E+04	3.15E+04	3.35E+04	3.55E+04	3.76E+04	3.98E+04	4.21E+04	4.45E+04	4.69E+04	4.95E+04	5.21E+04	5.48E+04
1	BAULI_Urbanri	9.58E+03	1.07E+04	1.18E+04	1.30E+04	1.42E+04	1.55E+04	1.68E+04	1.82E+04	1.96E+04	2.12E+04	2.27E+04	2.44E+04	2.61E+04	2.79E+04	2.98E+04	3.17E+04	3.37E+04	3.58E+04	3.80E+04	4.02E+04	4.25E+04	4.49E+04	4.74E+04	5.00E+04	5.27E+04
**1**	**TOTAL LI URBAN BAU**	**3.63E+04**	**4.01E+04**	**4.41E+04**	**4.83E+04**	**5.27E+04**	**5.73E+04**	**6.21E+04**	**6.72E+04**	**7.25E+04**	**7.81E+04**	**8.39E+04**	**9.00E+04**	**9.64E+04**	**1.03E+05**	**1.10E+05**	**1.17E+05**	**1.25E+05**	**1.33E+05**	**1.41E+05**	**1.49E+05**	**1.58E+05**	**1.67E+05**	**1.76E+05**	**1.86E+05**	**1.96E+05**

SCENARIO NO.	SCENARIO DESCRIPTOR	2016	2017	2018	2019	2020	2021	2022	2023	2024	2025	2026	2027	2028	2029	2030	2031	2032	2033	2034	2035	2036	2037	2038	2039	2040
1	BAULMI_Ruralf	1.05E+05	1.09E+05	1.13E+05	1.16E+05	1.20E+05	1.23E+05	1.27E+05	1.30E+05	1.34E+05	1.37E+05	1.40E+05	1.44E+05	1.47E+05	1.50E+05	1.53E+05	1.56E+05	1.58E+05	1.61E+05	1.63E+05	1.66E+05	1.68E+05	1.70E+05	1.73E+05	1.75E+05	1.76E+05
1	BAULMI_Ruralm	6.75E+04	6.99E+04	7.23E+04	7.46E+04	7.69E+04	7.93E+04	8.15E+04	8.37E+04	8.59E+04	8.81E+04	9.02E+04	9.22E+04	9.42E+04	9.61E+04	9.80E+04	9.98E+04	1.02E+05	1.03E+05	1.05E+05	1.06E+05	1.08E+05	1.09E+05	1.11E+05	1.12E+05	1.13E+05
1	BAULMI_Ruralr	7.80E+04	8.05E+04	8.30E+04	8.54E+04	8.78E+04	9.02E+04	9.24E+04	9.47E+04	9.68E+04	9.89E+04	1.01E+05	1.03E+05	1.05E+05	1.07E+05	1.08E+05	1.10E+05	1.12E+05	1.13E+05	1.14E+05	1.16E+05	1.17E+05	1.18E+05	1.19E+05	1.20E+05	1.21E+05
**1**	**TOTAL LMI RURAL BAU**	**2.51E+05**	**2.59E+05**	**2.68E+05**	**2.76E+05**	**2.85E+05**	**2.93E+05**	**3.01E+05**	**3.09E+05**	**3.17E+05**	**3.24E+05**	**3.32E+05**	**3.39E+05**	**3.46E+05**	**3.52E+05**	**3.59E+05**	**3.65E+05**	**3.71E+05**	**3.77E+05**	**3.83E+05**	**3.88E+05**	**3.93E+05**	**3.98E+05**	**4.02E+05**	**4.06E+05**	**4.10E+05**
1	BAULMI_Urbanf	8.81E+04	9.43E+04	1.01E+05	1.07E+05	1.14E+05	1.21E+05	1.28E+05	1.35E+05	1.43E+05	1.51E+05	1.59E+05	1.68E+05	1.76E+05	1.85E+05	1.95E+05	2.05E+05	2.14E+05	2.24E+05	2.35E+05	2.45E+05	2.56E+05	2.67E+05	2.79E+05	2.90E+05	3.02E+05
1	BAULMI_Urbanm	7.44E+04	7.86E+04	8.29E+04	8.74E+04	9.21E+04	9.68E+04	1.02E+05	1.07E+05	1.12E+05	1.17E+05	1.23E+05	1.28E+05	1.34E+05	1.40E+05	1.46E+05	1.53E+05	1.59E+05	1.65E+05	1.72E+05	1.79E+05	1.86E+05	1.93E+05	2.00E+05	2.07E+05	2.15E+05
1	BAULMI_Urbanr	6.44E+04	6.85E+04	7.27E+04	7.70E+04	8.15E+04	8.61E+04	9.09E+04	9.60E+04	1.01E+05	1.07E+05	1.12E+05	1.18E+05	1.24E+05	1.30E+05	1.36E+05	1.42E+05	1.49E+05	1.55E+05	1.61E+05	1.68E+05	1.75E+05	1.82E+05	1.89E+05	1.96E+05	2.03E+05
**1**	**TOTAL LMI URBAN BAU**	**2.27E+05**	**2.41E+05**	**2.56E+05**	**2.72E+05**	**2.87E+05**	**3.04E+05**	**3.20E+05**	**3.38E+05**	**3.56E+05**	**3.75E+05**	**3.94E+05**	**4.14E+05**	**4.34E+05**	**4.55E+05**	**4.77E+05**	**4.99E+05**	**5.22E+05**	**5.45E+05**	**5.68E+05**	**5.92E+05**	**6.17E+05**	**6.42E+05**	**6.67E+05**	**6.93E+05**	**7.20E+05**
1	BAUUMI_Ruralf	9.90E+04	1.02E+05	1.04E+05	1.07E+05	1.09E+05	1.11E+05	1.13E+05	1.15E+05	1.16E+05	1.18E+05	1.20E+05	1.21E+05	1.22E+05	1.24E+05	1.25E+05	1.26E+05	1.27E+05	1.28E+05	1.29E+05	1.30E+05	1.31E+05	1.32E+05	1.32E+05	1.33E+05	1.34E+05
1	BAUUMI_Ruralm	5.46E+04	5.62E+04	5.75E+04	5.88E+04	6.00E+04	6.12E+04	6.23E+04	6.33E+04	6.42E+04	6.51E+04	6.60E+04	6.67E+04	6.75E+04	6.82E+04	6.88E+04	6.94E+04	7.00E+04	7.06E+04	7.11E+04	7.16E+04	7.21E+04	7.25E+04	7.30E+04	7.34E+04	7.38E+04
1	BAUUMI_Ruralr	7.39E+04	7.56E+04	7.72E+04	7.87E+04	8.01E+04	8.14E+04	8.26E+04	8.36E+04	8.46E+04	8.55E+04	8.63E+04	8.71E+04	8.77E+04	8.84E+04	8.89E+04	8.94E+04	8.99E+04	9.03E+04	9.07E+04	9.11E+04	9.14E+04	9.17E+04	9.19E+04	9.22E+04	9.24E+04
**1**	**TOTAL UMI RURAL BAU**	**2.27E+05**	**2.33E+05**	**2.39E+05**	**2.44E+05**	**2.49E+05**	**2.53E+05**	**2.58E+05**	**2.62E+05**	**2.65E+05**	**2.69E+05**	**2.72E+05**	**2.75E+05**	**2.78E+05**	**2.80E+05**	**2.83E+05**	**2.85E+05**	**2.87E+05**	**2.89E+05**	**2.91E+05**	**2.93E+05**	**2.94E+05**	**2.96E+05**	**2.97E+05**	**2.99E+05**	**3.00E+05**
1	BAUUMI_Urbanf	2.20E+05	2.39E+05	2.56E+05	2.74E+05	2.92E+05	3.10E+05	3.29E+05	3.48E+05	3.67E+05	3.87E+05	4.07E+05	4.27E+05	4.47E+05	4.68E+05	4.88E+05	5.09E+05	5.30E+05	5.51E+05	5.72E+05	5.93E+05	6.15E+05	6.36E+05	6.57E+05	6.78E+05	7.00E+05
1	BAUUMI_Urbanm	1.37E+05	1.48E+05	1.58E+05	1.68E+05	1.79E+05	1.89E+05	2.00E+05	2.11E+05	2.22E+05	2.33E+05	2.44E+05	2.55E+05	2.67E+05	2.78E+05	2.90E+05	3.01E+05	3.13E+05	3.24E+05	3.36E+05	3.48E+05	3.59E+05	3.71E+05	3.83E+05	3.94E+05	4.06E+05
1	BAUUMI_Urbanr	1.36E+05	1.47E+05	1.58E+05	1.70E+05	1.81E+05	1.93E+05	2.05E+05	2.18E+05	2.30E+05	2.43E+05	2.56E+05	2.69E+05	2.82E+05	2.95E+05	3.09E+05	3.22E+05	3.35E+05	3.48E+05	3.61E+05	3.74E+05	3.87E+05	4.00E+05	4.13E+05	4.26E+05	4.39E+05
**1**	**TOTAL UMI**	**4.94E+05**	**5.34E+05**	**5.73E+05**	**6.12E+05**	**6.52E+05**	**6.92E+05**	**7.34E+05**	**7.76E+05**	**8.19E+05**	**8.63E+05**	**9.07E+05**	**9.51E+05**	**9.96E+05**	**1.04E+06**	**1.09E+06**	**1.13E+06**	**1.18E+06**	**1.22E+06**	**1.27E+06**	**1.32E+06**	**1.36E+06**	**1.41E+06**	**1.45E+06**	**1.50E+06**	**1.55E+06**

SCENARIO NO.	SCENARIO DESCRIPTOR	2016	2017	2018	2019	2020	2021	2022	2023	2024	2025	2026	2027	2028	2029	2030	2031	2032	2033	2034	2035	2036	2037	2038	2039	2040
	**URBAN BAU**																									
**1**	**TOTAL GLOBAL BAU ANNUAL**	**2.15E+06**	**2.24E+06**	**2.33E+06**	**2.42E+06**	**2.51E+06**	**2.61E+06**	**2.70E+06**	**2.80E+06**	**2.89E+06**	**2.99E+06**	**3.09E+06**	**3.19E+06**	**3.29E+06**	**3.39E+06**	**3.49E+06**	**3.59E+06**	**3.69E+06**	**3.79E+06**	**3.90E+06**	**4.00E+06**	**4.10E+06**	**4.21E+06**	**4.31E+06**	**4.42E+06**	**4.52E+06**
**1**	**TOTAL GLOBAL BAU CUMULATIVE**	**2.15E+06**	**4.39E+06**	**6.72E+06**	**9.14E+06**	**1.17E+07**	**1.43E+07**	**1.70E+07**	**1.98E+07**	**2.26E+07**	**2.56E+07**	**2.87E+07**	**3.19E+07**	**3.52E+07**	**3.86E+07**	**4.21E+07**	**4.57E+07**	**4.94E+07**	**5.31E+07**	**5.70E+07**	**6.10E+07**	**6.51E+07**	**6.94E+07**	**7.37E+07**	**7.81E+07**	**8.26E+07**
2	CCSHI_Ruralfl	3.72E+04	3.76E+04	3.79E+04	3.83E+04	3.86E+04	3.74E+04	3.66E+04	3.68E+04	3.70E+04	3.78E+04	3.85E+04	3.86E+04	3.88E+04	3.88E+04	3.89E+04	3.90E+04	3.89E+04	3.90E+04	3.89E+04	3.89E+04	3.88E+04	3.88E+04	3.86E+04	3.85E+04	3.84E+04
2	CCSHI_Ruralmu	3.90E+04	3.96E+04	3.99E+04	4.07E+04	4.11E+04	4.16E+04	4.21E+04	4.25E+04	4.29E+04	4.21E+04	4.17E+04	4.20E+04	4.21E+04	4.24E+04	4.27E+04	4.28E+04	4.29E+04	4.30E+04	4.33E+04	4.34E+04	4.33E+04	4.36E+04	4.36E+04	4.35E+04	4.35E+04
2	CCSHI_Ruralri	8.57E+04	8.63E+04	8.66E+04	8.69E+04	8.72E+04	8.66E+04	8.60E+04	8.60E+04	8.62E+04	8.56E+04	8.50E+04	8.49E+04	8.47E+04	8.44E+04	8.41E+04	8.39E+04	8.34E+04	8.30E+04	8.25E+04	8.20E+04	8.15E+04	8.09E+04	8.03E+04	7.97E+04	7.90E+04
**2**	**TOTAL HI RURAL CCS**	**1.62E+05**	**1.64E+05**	**1.64E+05**	**1.66E+05**	**1.67E+05**	**1.66E+05**	**1.65E+05**	**1.65E+05**	**1.66E+05**	**1.65E+05**	**1.65E+05**	**1.66E+05**	**1.66E+05**	**1.66E+05**	**1.66E+05**	**1.66E+05**	**1.65E+05**	**1.65E+05**	**1.65E+05**	**1.64E+05**	**1.64E+05**	**1.63E+05**	**1.62E+05**	**1.62E+05**	**1.61E+05**
2	CCSHI_Urbanfl	1.59E+05	1.62E+05	1.66E+05	1.69E+05	1.73E+05	1.70E+05	1.68E+05	1.71E+05	1.74E+05	1.81E+05	1.87E+05	1.90E+05	1.94E+05	1.97E+05	2.01E+05	2.04E+05	2.08E+05	2.12E+05	2.15E+05	2.19E+05	2.22E+05	2.26E+05	2.30E+05	2.33E+05	2.37E+05
2	CCSHI_Urbanmu	1.65E+05	1.69E+05	1.72E+05	1.77E+05	1.82E+05	1.86E+05	1.91E+05	1.95E+05	2.00E+05	1.99E+05	2.00E+05	2.04E+05	2.09E+05	2.13E+05	2.18E+05	2.23E+05	2.27E+05	2.32E+05	2.37E+05	2.42E+05	2.47E+05	2.52E+05	2.57E+05	2.62E+05	2.67E+05
2	CCSHI_Urbanri	3.74E+05	3.83E+05	3.90E+05	3.97E+05	4.02E+05	4.05E+05	4.08E+05	4.14E+05	4.21E+05	4.19E+05	4.23E+05	4.30E+05	4.35E+05	4.41E+05	4.46E+05	4.51E+05	4.56E+05	4.61E+05	4.74E+05	4.82E+05	4.85E+05	5.00E+05	4.94E+05	5.06E+05	5.17E+05
**2**	**TOTAL HI URBAN CCS**	**6.98E+05**	**7.14E+05**	**7.28E+05**	**7.43E+05**	**7.57E+05**	**7.61E+05**	**7.67E+05**	**7.80E+05**	**7.94E+05**	**7.99E+05**	**8.09E+05**	**8.25E+05**	**8.38E+05**	**8.52E+05**	**8.65E+05**	**8.78E+05**	**8.91E+05**	**9.05E+05**	**9.26E+05**	**9.42E+05**	**9.54E+05**	**9.78E+05**	**9.81E+05**	**1.00E+06**	**1.02E+06**
2	CCSLI_Ruralfl	2.33E+04	2.49E+04	2.65E+04	2.81E+04	2.97E+04	2.95E+04	3.02E+04	3.17E+04	3.33E+04	3.74E+04	4.07E+04	4.24E+04	4.41E+04	4.59E+04	4.76E+04	4.94E+04	5.11E+04	5.29E+04	5.46E+04	5.64E+04	5.81E+04	5.98E+04	6.16E+04	6.33E+04	6.50E+04
2	CCSLI_Ruralmu	1.30E+04	1.38E+04	1.47E+04	1.56E+04	1.65E+04	1.74E+04	1.83E+04	1.93E+04	2.02E+04	1.77E+04	1.66E+04	1.74E+04	1.81E+04	1.88E+04	1.95E+04	2.02E+04	2.10E+04	2.17E+04	2.24E+04	2.31E+04	2.38E+04	2.45E+04	2.52E+04	2.59E+04	2.66E+04
2	CCSLI_Ruralri	1.73E+04	1.83E+04	1.94E+04	2.06E+04	2.17E+04	2.27E+04	2.37E+04	2.49E+04	2.60E+04	2.69E+04	2.79E+04	2.90E+04	3.01E+04	3.12E+04	3.22E+04	3.33E+04	3.44E+04	3.55E+04	3.65E+04	3.76E+04	3.86E+04	3.96E+04	4.06E+04	4.16E+04	4.26E+04
**2**	**TOTAL LI RURAL CCS**	**5.36E+04**	**5.71E+04**	**6.06E+04**	**6.42E+04**	**6.79E+04**	**6.96E+04**	**7.23E+04**	**7.59E+04**	**7.94E+04**	**8.20E+04**	**8.52E+04**	**8.87E+04**	**9.23E+04**	**9.58E+04**	**9.94E+04**	**1.03E+05**	**1.06E+05**	**1.10E+05**	**1.14E+05**	**1.17E+05**	**1.21E+05**	**1.24E+05**	**1.27E+05**	**1.31E+05**	**1.34E+05**

SCENARIO NO.	SCENARIO DESCRIPTOR	2016	2017	2018	2019	2020	2021	2022	2023	2024	2025	2026	2027	2028	2029	2030	2031	2032	2033	2034	2035	2036	2037	2038	2039	2040
2	CCSLI_Urbanfl	1.52E+04	1.69E+04	1.86E+04	2.05E+04	2.25E+04	2.29E+04	2.41E+04	2.62E+04	2.84E+04	3.32E+04	3.75E+04	4.04E+04	4.33E+04	4.65E+04	4.97E+04	5.31E+04	5.66E+04	6.03E+04	6.41E+04	6.81E+04	7.22E+04	7.64E+04	8.09E+04	8.54E+04	9.02E+04
2	CCSLI_Urbanmu	1.15E+04	1.26E+04	1.37E+04	1.48E+04	1.60E+04	1.73E+04	1.86E+04	2.00E+04	2.14E+04	1.92E+04	1.84E+04	1.97E+04	2.10E+04	2.23E+04	2.37E+04	2.52E+04	2.67E+04	2.83E+04	3.00E+04	3.17E+04	3.35E+04	3.53E+04	3.73E+04	3.92E+04	4.13E+04
2	CCSLI_Urbanri	9.59E+03	1.07E+04	1.18E+04	1.30E+04	1.42E+04	1.54E+04	1.66E+04	1.80E+04	1.95E+04	2.07E+04	2.21E+04	2.37E+04	2.54E+04	2.72E+04	2.90E+04	3.09E+04	3.28E+04	3.49E+04	3.70E+04	3.92E+04	4.15E+04	4.38E+04	4.62E+04	4.88E+04	5.13E+04
**2**	**TOTAL LI URBAN CCS**	**3.63E+04**	**4.01E+04**	**4.41E+04**	**4.83E+04**	**5.27E+04**	**5.55E+04**	**5.93E+04**	**6.42E+04**	**6.93E+04**	**7.30E+04**	**7.81E+04**	**8.38E+04**	**8.97E+04**	**9.59E+04**	**1.02E+05**	**1.09E+05**	**1.16E+05**	**1.23E+05**	**1.31E+05**	**1.39E+05**	**1.47E+05**	**1.56E+05**	**1.64E+05**	**1.73E+05**	**1.83E+05**
2	CCSLMI_Ruralf	1.05E+05	1.09E+05	1.13E+05	1.16E+05	1.20E+05	1.16E+05	1.15E+05	1.18E+05	1.22E+05	1.34E+05	1.43E+05	1.46E+05	1.49E+05	1.52E+05	1.55E+05	1.58E+05	1.61E+05	1.64E+05	1.66E+05	1.69E+05	1.71E+05	1.73E+05	1.75E+05	1.77E+05	1.79E+05
2	CCSLMI_Ruralm	6.78E+04	7.01E+04	7.25E+04	7.49E+04	7.73E+04	7.96E+04	8.18E+04	8.41E+04	8.63E+04	7.43E+04	6.90E+04	7.06E+04	7.21E+04	7.36E+04	7.50E+04	7.64E+04	7.77E+04	7.90E+04	8.02E+04	8.14E+04	8.25E+04	8.36E+04	8.46E+04	8.56E+04	8.65E+04
2	CCSLMI_Ruralr	7.78E+04	8.03E+04	8.28E+04	8.52E+04	8.76E+04	8.99E+04	9.07E+04	9.20E+04	9.42E+04	9.54E+04	9.69E+04	9.88E+04	1.01E+05	1.02E+05	1.04E+05	1.06E+05	1.07E+05	1.09E+05	1.10E+05	1.11E+05	1.13E+05	1.14E+05	1.15E+05	1.16E+05	1.17E+05
**2**	**TOTAL LMI RURAL CCS**	**2.51E+05**	**2.59E+05**	**2.68E+05**	**2.76E+05**	**2.85E+05**	**2.85E+05**	**2.88E+05**	**2.95E+05**	**3.02E+05**	**3.03E+05**	**3.09E+05**	**3.16E+05**	**3.22E+05**	**3.28E+05**	**3.34E+05**	**3.40E+05**	**3.46E+05**	**3.51E+05**	**3.56E+05**	**3.61E+05**	**3.66E+05**	**3.70E+05**	**3.75E+05**	**3.78E+05**	**3.82E+05**
2	CCSLMI_Urbanf	8.77E+04	9.39E+04	1.00E+05	1.07E+05	1.13E+05	1.09E+05	1.10E+05	1.17E+05	1.24E+05	1.44E+05	1.62E+05	1.71E+05	1.80E+05	1.89E+05	1.98E+05	2.08E+05	2.18E+05	2.28E+05	2.39E+05	2.50E+05	2.61E+05	2.72E+05	2.83E+05	2.95E+05	3.07E+05
2	CCSLMI_Urbanm	7.47E+04	7.89E+04	8.33E+04	8.78E+04	9.24E+04	9.71E+04	1.02E+05	1.07E+05	1.12E+05	9.86E+04	9.22E+04	9.65E+04	1.01E+05	1.05E+05	1.10E+05	1.15E+05	1.19E+05	1.24E+05	1.29E+05	1.34E+05	1.40E+05	1.45E+05	1.50E+05	1.56E+05	1.62E+05
2	CCSLMI_Urbanr	6.44E+04	6.84E+04	7.27E+04	7.70E+04	8.15E+04	8.61E+04	8.87E+04	9.24E+04	9.76E+04	1.02E+05	1.06E+05	1.12E+05	1.17E+05	1.23E+05	1.29E+05	1.35E+05	1.41E+05	1.47E+05	1.54E+05	1.60E+05	1.67E+05	1.73E+05	1.80E+05	1.87E+05	1.94E+05
**2**	**TOTAL LMI URBAN CCS**	**2.27E+05**	**2.41E+05**	**2.56E+05**	**2.72E+05**	**2.87E+05**	**2.93E+05**	**3.01E+05**	**3.16E+05**	**3.33E+05**	**3.45E+05**	**3.60E+05**	**3.79E+05**	**3.98E+05**	**4.17E+05**	**4.37E+05**	**4.58E+05**	**4.79E+05**	**5.00E+05**	**5.22E+05**	**5.44E+05**	**5.67E+05**	**5.90E+05**	**6.14E+05**	**6.38E+05**	**6.62E+05**
2	CCSUMI_Ruralf	9.91E+04	1.02E+05	1.04E+05	1.07E+05	1.09E+05	1.06E+05	1.06E+05	1.08E+05	1.09E+05	1.19E+05	1.26E+05	1.27E+05	1.29E+05	1.30E+05	1.31E+05	1.32E+05	1.34E+05	1.35E+05	1.36E+05	1.37E+05	1.38E+05	1.39E+05	1.39E+05	1.40E+05	1.41E+05
2	CCSUMI_Ruralm	5.49E+04	5.64E+04	5.78E+04	5.91E+04	6.03E+04	6.15E+04	6.26E+04	6.36E+04	6.45E+04	5.49E+04	4.92E+04	4.97E+04	5.03E+04	5.08E+04	5.13E+04	5.18E+04	5.22E+04	5.26E+04	5.30E+04	5.34E+04	5.38E+04	5.41E+04	5.44E+04	5.48E+04	5.51E+04
2	CCSUMI_Ruralr	7.37E+04	7.55E+04	7.71E+04	7.86E+04	7.99E+04	8.12E+04	8.23E+04	8.34E+04	8.44E+04	8.46E+04	8.50E+04	8.57E+04	8.64E+04	8.70E+04	8.75E+04	8.80E+04	8.85E+04	8.89E+04	8.93E+04	8.96E+04	9.00E+04	9.02E+04	9.05E+04	9.08E+04	9.10E+04
**2**	**TOTAL UMI RURAL CCS**	**2.28E+05**	**2.34E+05**	**2.39E+05**	**2.44E+05**	**2.49E+05**	**2.49E+05**	**2.51E+05**	**2.55E+05**	**2.58E+05**	**2.58E+05**	**2.60E+05**	**2.63E+05**	**2.65E+05**	**2.68E+05**	**2.70E+05**	**2.72E+05**	**2.74E+05**	**2.76E+05**	**2.78E+05**	**2.80E+05**	**2.81E+05**	**2.83E+05**	**2.84E+05**	**2.86E+05**	**2.87E+05**

SCENARIO NO.	SCENARIO DESCRIPTOR	2016	2017	2018	2019	2020	2021	2022	2023	2024	2025	2026	2027	2028	2029	2030	2031	2032	2033	2034	2035	2036	2037	2038	2039	2040
2	CCSUMI_Urbanf	2.21E+05	2.39E+05	2.56E+05	2.74E+05	2.92E+05	2.95E+05	3.05E+05	3.22E+05	3.40E+05	3.89E+05	4.30E+05	4.52E+05	4.73E+05	4.94E+05	5.16E+05	5.38E+05	5.60E+05	5.82E+05	6.04E+05	6.26E+05	6.49E+05	6.71E+05	6.94E+05	7.16E+05	7.39E+05
2	CCSUMI_Urbanm	1.38E+05	1.49E+05	1.59E+05	1.69E+05	1.80E+05	1.90E+05	2.01E+05	2.12E+05	2.23E+05	1.94E+05	1.79E+05	1.87E+05	1.96E+05	2.04E+05	2.13E+05	2.21E+05	2.30E+05	2.38E+05	2.47E+05	2.55E+05	2.64E+05	2.73E+05	2.81E+05	2.90E+05	2.98E+05
2	CCSUMI_Urbanr	1.36E+05	1.47E+05	1.58E+05	1.70E+05	1.81E+05	1.93E+05	2.05E+05	2.17E+05	2.30E+05	2.51E+05	2.60E+05	2.75E+05	2.89E+05	3.02E+05	3.16E+05	3.30E+05	3.44E+05	3.58E+05	3.72E+05	3.85E+05	3.99E+05	4.13E+05	4.26E+05	4.39E+05	4.52E+05
**2**	**TOTAL UMI URBAN CCS**	**4.95E+05**	**5.35E+05**	**5.74E+05**	**6.13E+05**	**6.53E+05**	**6.77E+05**	**7.10E+05**	**7.51E+05**	**7.93E+05**	**8.34E+05**	**8.70E+05**	**9.14E+05**	**9.57E+05**	**1.00E+06**	**1.05E+06**	**1.09E+06**	**1.13E+06**	**1.18E+06**	**1.22E+06**	**1.27E+06**	**1.31E+06**	**1.36E+06**	**1.40E+06**	**1.45E+06**	**1.49E+06**
**2**	**TOTAL GLOBAL CCS ANNUAL**	**2.15E+06**	**2.24E+06**	**2.33E+06**	**2.43E+06**	**2.52E+06**	**2.56E+06**	**2.61E+06**	**2.70E+06**	**2.80E+06**	**2.86E+06**	**2.94E+06**	**3.03E+06**	**3.13E+06**	**3.22E+06**	**3.32E+06**	**3.42E+06**	**3.51E+06**	**3.61E+06**	**3.71E+06**	**3.81E+06**	**3.91E+06**	**4.02E+06**	**4.11E+06**	**4.21E+06**	**4.32E+06**
**2**	**TOTAL GLOBAL CCS CUMULATIVE**	**2.15E+06**	**4.39E+06**	**6.73E+06**	**9.16E+06**	**1.17E+07**	**1.42E+07**	**1.68E+07**	**1.95E+07**	**2.23E+07**	**2.52E+07**	**2.81E+07**	**3.12E+07**	**3.43E+07**	**3.75E+07**	**4.08E+07**	**4.43E+07**	**4.78E+07**	**5.14E+07**	**5.51E+07**	**5.89E+07**	**6.28E+07**	**6.68E+07**	**7.09E+07**	**7.52E+07**	**7.95E+07**
3	CDSHI_Ruralfl	3.72E+04	3.78E+04	3.82E+04	3.85E+04	3.88E+04	3.77E+04	3.66E+04	3.68E+04	3.70E+04	3.79E+04	3.85E+04	3.87E+04	3.88E+04	3.89E+04	3.90E+04	3.90E+04	3.91E+04	3.91E+04	3.91E+04	3.90E+04	3.90E+04	3.89E+04	3.88E+04	3.87E+04	3.86E+04
3	CDSHI_Ruralmu	3.86E+04	3.94E+04	4.00E+04	4.05E+04	4.09E+04	4.16E+04	4.16E+04	4.19E+04	4.23E+04	4.14E+04	4.09E+04	4.11E+04	4.14E+04	4.16E+04	4.18E+04	4.20E+04	4.22E+04	4.24E+04	4.25E+04	4.26E+04	4.27E+04	4.27E+04	4.28E+04	4.28E+04	4.28E+04
3	CDSHI_Ruralri	8.56E+04	8.63E+04	8.68E+04	8.71E+04	8.73E+04	8.66E+04	8.57E+04	8.58E+04	8.58E+04	8.52E+04	8.46E+04	8.45E+04	8.44E+04	8.42E+04	8.39E+04	8.36E+04	8.33E+04	8.29E+04	8.25E+04	8.20E+04	8.14E+04	8.09E+04	8.03E+04	7.96E+04	7.90E+04
**3**	**TOTAL HI RURAL CDS**	**1.61E+05**	**1.64E+05**	**1.65E+05**	**1.66E+05**	**1.67E+05**	**1.66E+05**	**1.64E+05**	**1.64E+05**	**1.65E+05**	**1.64E+05**	**1.64E+05**	**1.64E+05**	**1.65E+05**	**1.65E+05**	**1.65E+05**	**1.65E+05**	**1.65E+05**	**1.64E+05**	**1.64E+05**	**1.64E+05**	**1.63E+05**	**1.63E+05**	**1.62E+05**	**1.61E+05**	**1.60E+05**
3	CDSHI_Urbanfl	1.59E+05	1.63E+05	1.67E+05	1.70E+05	1.74E+05	1.72E+05	1.67E+05	1.71E+05	1.74E+05	1.81E+05	1.87E+05	1.90E+05	1.94E+05	1.98E+05	2.01E+05	2.05E+05	2.08E+05	2.12E+05	2.16E+05	2.19E+05	2.23E+05	2.27E+05	2.31E+05	2.34E+05	2.38E+05
3	CDSHI_Urbanmu	1.63E+05	1.68E+05	1.73E+05	1.77E+05	1.81E+05	1.88E+05	1.89E+05	1.94E+05	1.98E+05	1.97E+05	1.98E+05	2.02E+05	2.07E+05	2.11E+05	2.16E+05	2.20E+05	2.25E+05	2.30E+05	2.35E+05	2.40E+05	2.44E+05	2.49E+05	2.54E+05	2.59E+05	2.64E+05
3	CDSHI_Urbanri	3.74E+05	3.83E+05	3.92E+05	3.98E+05	4.04E+05	4.08E+05	4.03E+05	4.08E+05	4.14E+05	4.06E+05	4.08E+05	4.13E+05	4.18E+05	4.24E+05	4.29E+05	4.34E+05	4.39E+05	4.45E+05	4.50E+05	4.56E+05	4.61E+05	4.66E+05	4.72E+05	4.77E+05	4.83E+05
**3**	**TOTAL HI URBAN CDS**	**6.95E+05**	**7.14E+05**	**7.31E+05**	**7.46E+05**	**7.60E+05**	**7.68E+05**	**7.60E+05**	**7.73E+05**	**7.86E+05**	**7.84E+05**	**7.92E+05**	**8.06E+05**	**8.19E+05**	**8.32E+05**	**8.46E+05**	**8.59E+05**	**8.73E+05**	**8.87E+05**	**9.01E+05**	**9.15E+05**	**9.29E+05**	**9.43E+05**	**9.57E+05**	**9.71E+05**	**9.85E+05**
3	CDSLI_Ruralfl	2.34E+04	2.49E+04	2.65E+04	2.81E+04	2.97E+04	2.95E+04	3.01E+04	3.15E+04	3.29E+04	3.69E+04	4.00E+04	4.16E+04	4.31E+04	4.47E+04	4.62E+04	4.77E+04	4.92E+04	5.07E+04	5.22E+04	5.36E+04	5.50E+04	5.64E+04	5.78E+04	5.91E+04	6.04E+04

SCENARIO NO.	SCENARIO DESCRIPTOR	2016	2017	2018	2019	2020	2021	2022	2023	2024	2025	2026	2027	2028	2029	2030	2031	2032	2033	2034	2035	2036	2037	2038	2039	2040
3	CDSLI_Ruralmu	1.29E+04	1.38E+04	1.47E+04	1.55E+04	1.64E+04	1.73E+04	1.81E+04	1.90E+04	1.98E+04	1.73E+04	1.61E+04	1.67E+04	1.73E+04	1.79E+04	1.85E+04	1.91E+04	1.97E+04	2.02E+04	2.08E+04	2.13E+04	2.19E+04	2.24E+04	2.29E+04	2.33E+04	2.38E+04
3	CDSLI_Ruralri	1.73E+04	1.84E+04	1.95E+04	2.06E+04	2.17E+04	2.27E+04	2.37E+04	2.48E+04	2.58E+04	2.66E+04	2.75E+04	2.85E+04	2.95E+04	3.05E+04	3.14E+04	3.24E+04	3.33E+04	3.42E+04	3.51E+04	3.60E+04	3.68E+04	3.77E+04	3.85E+04	3.93E+04	4.00E+04
**3**	**TOTAL LI RURAL CDS**	**5.36E+04**	**5.71E+04**	**6.07E+04**	**6.43E+04**	**6.79E+04**	**6.95E+04**	**7.20E+04**	**7.53E+04**	**7.86E+04**	**8.08E+04**	**8.37E+04**	**8.68E+04**	**9.00E+04**	**9.30E+04**	**9.61E+04**	**9.92E+04**	**1.02E+05**	**1.05E+05**	**1.08E+05**	**1.11E+05**	**1.14E+05**	**1.16E+05**	**1.19E+05**	**1.22E+05**	**1.24E+05**
3	CDSLI_Urbanfl	1.52E+04	1.69E+04	1.87E+04	2.06E+04	2.25E+04	2.07E+04	2.13E+04	2.26E+04	2.41E+04	2.82E+04	3.19E+04	3.39E+04	3.61E+04	3.83E+04	4.05E+04	4.29E+04	4.54E+04	4.79E+04	5.05E+04	5.31E+04	5.58E+04	5.85E+04	6.13E+04	6.40E+04	6.68E+04
3	CDSLI_Urbanmu	1.15E+04	1.25E+04	1.36E+04	1.48E+04	1.59E+04	1.70E+04	1.83E+04	1.95E+04	2.08E+04	1.85E+04	1.76E+04	1.86E+04	1.97E+04	2.07E+04	2.18E+04	2.30E+04	2.41E+04	2.53E+04	2.65E+04	2.77E+04	2.89E+04	3.02E+04	3.14E+04	3.26E+04	3.39E+04
3	CDSLI_Urbanri	9.58E+03	1.07E+04	1.18E+04	1.30E+04	1.42E+04	1.49E+04	1.64E+04	1.76E+04	1.89E+04	2.00E+04	2.12E+04	2.26E+04	2.40E+04	2.55E+04	2.69E+04	2.84E+04	3.00E+04	3.17E+04	3.34E+04	3.52E+04	3.70E+04	3.88E+04	4.07E+04	4.27E+04	4.47E+04
**3**	**TOTAL LI URBAN CDS**	**3.63E+04**	**4.01E+04**	**4.41E+04**	**4.83E+04**	**5.27E+04**	**5.27E+04**	**5.59E+04**	**5.98E+04**	**6.39E+04**	**6.68E+04**	**7.07E+04**	**7.52E+04**	**7.98E+04**	**8.45E+04**	**8.93E+04**	**9.43E+04**	**9.95E+04**	**1.05E+05**	**1.10E+05**	**1.16E+05**	**1.22E+05**	**1.28E+05**	**1.33E+05**	**1.39E+05**	**1.45E+05**
3	CDSLMI_Ruralf	1.05E+05	1.09E+05	1.13E+05	1.16E+05	1.20E+05	1.16E+05	1.15E+05	1.18E+05	1.20E+05	1.32E+05	1.41E+05	1.43E+05	1.46E+05	1.48E+05	1.51E+05	1.53E+05	1.55E+05	1.57E+05	1.59E+05	1.61E+05	1.63E+05	1.64E+05	1.66E+05	1.67E+05	1.68E+05
3	CDSLMI_Ruralm	6.74E+04	6.98E+04	7.22E+04	7.45E+04	7.69E+04	7.88E+04	8.07E+04	8.25E+04	8.43E+04	7.21E+04	6.64E+04	6.75E+04	6.85E+04	6.95E+04	7.03E+04	7.12E+04	7.21E+04	7.28E+04	7.35E+04	7.41E+04	7.47E+04	7.51E+04	7.55E+04	7.58E+04	7.61E+04
3	CDSLMI_Ruralr	7.81E+04	8.06E+04	8.31E+04	8.55E+04	8.79E+04	9.00E+04	9.06E+04	9.17E+04	9.36E+04	9.45E+04	9.58E+04	9.74E+04	9.89E+04	1.00E+05	1.02E+05	1.03E+05	1.04E+05	1.05E+05	1.06E+05	1.07E+05	1.08E+05	1.09E+05	1.09E+05	1.10E+05	1.10E+05
**3**	**TOTAL LMI RURAL CDS**	**2.51E+05**	**2.59E+05**	**2.68E+05**	**2.76E+05**	**2.85E+05**	**2.85E+05**	**2.86E+05**	**2.92E+05**	**2.98E+05**	**2.99E+05**	**3.03E+05**	**3.08E+05**	**3.13E+05**	**3.18E+05**	**3.23E+05**	**3.27E+05**	**3.31E+05**	**3.35E+05**	**3.39E+05**	**3.42E+05**	**3.45E+05**	**3.48E+05**	**3.51E+05**	**3.53E+05**	**3.55E+05**
3	CDSLMI_Urbanf	8.81E+04	9.43E+04	1.01E+05	1.07E+05	1.14E+05	1.06E+05	1.08E+05	1.13E+05	1.19E+05	1.39E+05	1.55E+05	1.62E+05	1.69E+05	1.76E+05	1.83E+05	1.91E+05	1.98E+05	2.06E+05	2.14E+05	2.21E+05	2.29E+05	2.37E+05	2.45E+05	2.52E+05	2.60E+05
3	CDSLMI_Urbanm	7.43E+04	7.85E+04	8.29E+04	8.73E+04	9.20E+04	9.58E+04	1.00E+05	1.05E+05	1.09E+05	9.49E+04	8.76E+04	9.09E+04	9.41E+04	9.74E+04	1.01E+05	1.04E+05	1.08E+05	1.11E+05	1.14E+05	1.18E+05	1.21E+05	1.25E+05	1.28E+05	1.31E+05	1.34E+05
3	CDSLMI_Urbanr	6.44E+04	6.84E+04	7.28E+04	7.71E+04	8.16E+04	8.49E+04	8.85E+04	9.17E+04	9.62E+04	9.96E+04	1.03E+05	1.08E+05	1.13E+05	1.18E+05	1.22E+05	1.27E+05	1.32E+05	1.37E+05	1.42E+05	1.48E+05	1.53E+05	1.58E+05	1.63E+05	1.68E+05	1.74E+05
**3**	**TOTAL LMI URBAN CDS**	**2.27E+05**	**2.41E+05**	**2.56E+05**	**2.72E+05**	**2.87E+05**	**2.87E+05**	**2.97E+05**	**3.10E+05**	**3.24E+05**	**3.33E+05**	**3.46E+05**	**3.61E+05**	**3.76E+05**	**3.91E+05**	**4.06E+05**	**4.22E+05**	**4.38E+05**	**4.54E+05**	**4.71E+05**	**4.87E+05**	**5.03E+05**	**5.19E+05**	**5.36E+05**	**5.52E+05**	**5.69E+05**
3	CDSUMI_Ruralf	9.90E+04	1.02E+05	1.04E+05	1.07E+05	1.09E+05	1.06E+05	1.05E+05	1.07E+05	1.08E+05	1.17E+05	1.24E+05	1.25E+05	1.27E+05	1.28E+05	1.29E+05	1.29E+05	1.30E+05	1.31E+05	1.32E+05	1.32E+05	1.33E+05	1.34E+05	1.34E+05	1.35E+05	1.35E+05

SCENARIO NO.	SCENARIO DESCRIPTOR	2016	2017	2018	2019	2020	2021	2022	2023	2024	2025	2026	2027	2028	2029	2030	2031	2032	2033	2034	2035	2036	2037	2038	2039	2040
3	CDSUMI_Ruralm	5.45E+04	5.60E+04	5.74E+04	5.87E+04	5.99E+04	6.09E+04	6.17E+04	6.25E+04	6.33E+04	5.36E+04	4.77E+04	4.81E+04	4.85E+04	4.88E+04	4.91E+04	4.94E+04	4.96E+04	4.98E+04	5.00E+04	5.01E+04	5.03E+04	5.04E+04	5.05E+04	5.06E+04	5.07E+04
3	CDSUMI_Ruralr	7.39E+04	7.57E+04	7.72E+04	7.88E+04	8.01E+04	8.12E+04	8.22E+04	8.31E+04	8.39E+04	8.40E+04	8.42E+04	8.48E+04	8.53E+04	8.58E+04	8.62E+04	8.65E+04	8.68E+04	8.70E+04	8.72E+04	8.74E+04	8.76E+04	8.77E+04	8.78E+04	8.79E+04	8.80E+04
**3**	**TOTAL UMI RURAL CDS**	**2.27E+05**	**2.34E+05**	**2.39E+05**	**2.44E+05**	**2.49E+05**	**2.48E+05**	**2.49E+05**	**2.52E+05**	**2.55E+05**	**2.55E+05**	**2.56E+05**	**2.58E+05**	**2.60E+05**	**2.62E+05**	**2.64E+05**	**2.65E+05**	**2.67E+05**	**2.68E+05**	**2.69E+05**	**2.70E+05**	**2.71E+05**	**2.72E+05**	**2.73E+05**	**2.73E+05**	**2.74E+05**
3	CDSUMI_Urbanf	2.20E+05	2.39E+05	2.56E+05	2.74E+05	2.92E+05	2.88E+05	2.96E+05	3.10E+05	3.26E+05	3.69E+05	4.07E+05	4.24E+05	4.42E+05	4.59E+05	4.77E+05	4.95E+05	5.13E+05	5.32E+05	5.50E+05	5.69E+05	5.87E+05	6.05E+05	6.24E+05	6.42E+05	6.61E+05
3	CDSUMI_Urbanm	1.37E+05	1.48E+05	1.58E+05	1.68E+05	1.78E+05	1.83E+05	1.91E+05	2.00E+05	2.08E+05	1.81E+05	1.64E+05	1.70E+05	1.76E+05	1.82E+05	1.87E+05	1.93E+05	1.99E+05	2.05E+05	2.11E+05	2.17E+05	2.23E+05	2.29E+05	2.35E+05	2.40E+05	2.46E+05
3	CDSUMI_Urbanr	1.36E+05	1.47E+05	1.59E+05	1.70E+05	1.81E+05	1.89E+05	2.01E+05	2.12E+05	2.23E+05	2.53E+05	2.63E+05	2.74E+05	2.86E+05	2.98E+05	3.09E+05	3.21E+05	3.32E+05	3.44E+05	3.56E+05	3.67E+05	3.79E+05	3.90E+05	4.02E+05	4.13E+05	4.25E+05
**3**	**TOTAL UMI URBAN CDS**	**4.93E+05**	**5.34E+05**	**5.72E+05**	**6.11E+05**	**6.51E+05**	**6.59E+05**	**6.88E+05**	**7.22E+05**	**7.57E+05**	**8.03E+05**	**8.34E+05**	**8.69E+05**	**9.04E+05**	**9.39E+05**	**9.74E+05**	**1.01E+06**	**1.05E+06**	**1.08E+06**	**1.12E+06**	**1.15E+06**	**1.19E+06**	**1.22E+06**	**1.26E+06**	**1.30E+06**	**1.33E+06**
**3**	**TOTAL GLOBAL CDS ANNUAL**	**2.14E+06**	**2.24E+06**	**2.34E+06**	**2.43E+06**	**2.52E+06**	**2.53E+06**	**2.57E+06**	**2.65E+06**	**2.73E+06**	**2.79E+06**	**2.85E+06**	**2.93E+06**	**3.01E+06**	**3.08E+06**	**3.16E+06**	**3.24E+06**	**3.32E+06**	**3.40E+06**	**3.48E+06**	**3.56E+06**	**3.64E+06**	**3.71E+06**	**3.79E+06**	**3.87E+06**	**3.94E+06**
**3**	**TOTAL GLOBAL CDS CUMULATIVE**	**2.14E+06**	**4.39E+06**	**6.72E+06**	**9.15E+06**	**1.17E+07**	**1.42E+07**	**1.68E+07**	**1.94E+07**	**2.22E+07**	**2.49E+07**	**2.78E+07**	**3.07E+07**	**3.37E+07**	**3.68E+07**	**4.00E+07**	**4.32E+07**	**4.65E+07**	**4.99E+07**	**5.34E+07**	**5.70E+07**	**6.06E+07**	**6.43E+07**	**6.81E+07**	**7.20E+07**	**7.59E+07**
4	RESHI_Ruralfl	3.71E+04	3.75E+04	3.77E+04	3.82E+04	3.85E+04	3.85E+04	3.66E+04	3.70E+04	3.74E+04	3.75E+04	3.76E+04	3.79E+04	3.81E+04	3.84E+04	3.85E+04	3.87E+04	3.88E+04	3.88E+04	3.88E+04	3.87E+04	3.86E+04	3.84E+04	3.81E+04	3.77E+04	3.71E+04
4	RESHI_Ruralmu	3.89E+04	3.95E+04	3.98E+04	4.06E+04	4.10E+04	4.27E+04	4.12E+04	4.11E+04	4.10E+04	4.08E+04	4.06E+04	4.04E+04	4.02E+04	3.99E+04	3.96E+04	3.93E+04	3.90E+04	3.87E+04	3.83E+04	3.79E+04	3.75E+04	3.71E+04	3.66E+04	3.62E+04	3.57E+04
4	RESHI_Ruralri	8.51E+04	8.56E+04	8.59E+04	8.63E+04	8.65E+04	8.68E+04	8.47E+04	8.46E+04	8.44E+04	8.36E+04	8.28E+04	8.24E+04	8.19E+04	8.14E+04	8.08E+04	8.04E+04	7.99E+04	7.93E+04	7.88E+04	7.81E+04	7.74E+04	7.67E+04	7.59E+04	7.51E+04	7.42E+04
**4**	**TOTAL HI RURAL RES**	**1.61E+05**	**1.63E+05**	**1.63E+05**	**1.65E+05**	**1.66E+05**	**1.68E+05**	**1.63E+05**	**1.63E+05**	**1.63E+05**	**1.62E+05**	**1.61E+05**	**1.61E+05**	**1.60E+05**	**1.60E+05**	**1.59E+05**	**1.58E+05**	**1.58E+05**	**1.57E+05**	**1.56E+05**	**1.55E+05**	**1.54E+05**	**1.52E+05**	**1.51E+05**	**1.49E+05**	**1.47E+05**
4	RESHI_Urbanfl	1.58E+05	1.61E+05	1.65E+05	1.68E+05	1.72E+05	1.74E+05	1.67E+05	1.71E+05	1.75E+05	1.78E+05	1.81E+05	1.85E+05	1.89E+05	1.92E+05	1.96E+05	2.00E+05	2.03E+05	2.07E+05	2.10E+05	2.13E+05	2.16E+05	2.19E+05	2.22E+05	2.24E+05	2.25E+05

SCENARIO NO.	SCENARIO DESCRIPTOR	2016	2017	2018	2019	2020	2021	2022	2023	2024	2025	2026	2027	2028	2029	2030	2031	2032	2033	2034	2035	2036	2037	2038	2039	2040
4	RESHI_Urbanmu	1.64E+05	1.68E+05	1.71E+05	1.77E+05	1.82E+05	1.93E+05	1.87E+05	1.89E+05	1.91E+05	1.93E+05	1.94E+05	1.96E+05	1.97E+05	1.99E+05	2.00E+05	2.01E+05	2.03E+05	2.04E+05	2.06E+05	2.07E+05	2.09E+05	2.10E+05	2.11E+05	2.13E+05	2.15E+05
4	RESHI_Urbanri	3.72E+05	3.79E+05	3.85E+05	3.92E+05	3.98E+05	4.11E+05	3.98E+05	4.04E+05	4.08E+05	4.10E+05	4.13E+05	4.17E+05	4.21E+05	4.25E+05	4.29E+05	4.34E+05	4.39E+05	4.44E+05	4.48E+05	4.52E+05	4.57E+05	4.61E+05	4.65E+05	4.69E+05	4.72E+05
**4**	**TOTAL HI URBAN RES**	**6.94E+05**	**7.09E+05**	**7.21E+05**	**7.37E+05**	**7.51E+05**	**7.77E+05**	**7.53E+05**	**7.64E+05**	**7.75E+05**	**7.81E+05**	**7.88E+05**	**7.98E+05**	**8.07E+05**	**8.16E+05**	**8.25E+05**	**8.35E+05**	**8.45E+05**	**8.55E+05**	**8.64E+05**	**8.73E+05**	**8.82E+05**	**8.90E+05**	**8.98E+05**	**9.05E+05**	**9.12E+05**
4	RESLI_Ruralfl	2.33E+04	2.49E+04	2.64E+04	2.80E+04	2.96E+04	3.00E+04	3.18E+04	3.44E+04	3.72E+04	3.97E+04	4.24E+04	4.55E+04	4.86E+04	5.19E+04	5.52E+04	5.87E+04	6.23E+04	6.59E+04	6.97E+04	7.36E+04	7.75E+04	8.16E+04	8.57E+04	8.99E+04	9.42E+04
4	RESLI_Ruralmu	1.30E+04	1.39E+04	1.47E+04	1.56E+04	1.65E+04	1.67E+04	1.65E+04	1.62E+04	1.58E+04	1.52E+04	1.45E+04	1.38E+04	1.29E+04	1.20E+04	1.09E+04	9.77E+03	8.50E+03	7.11E+03	5.61E+03	4.00E+03	2.29E+03	4.58E+02	-1.48E+03	-3.53E+03	-5.68E+03
4	RESLI_Ruralri	1.72E+04	1.83E+04	1.94E+04	2.05E+04	2.16E+04	2.26E+04	2.37E+04	2.48E+04	2.60E+04	2.69E+04	2.79E+04	2.90E+04	3.01E+04	3.12E+04	3.23E+04	3.34E+04	3.45E+04	3.56E+04	3.67E+04	3.77E+04	3.88E+04	3.99E+04	4.09E+04	4.19E+04	4.29E+04
**4**	**TOTAL LI RURAL RES**	**5.35E+04**	**5.70E+04**	**6.06E+04**	**6.42E+04**	**6.78E+04**	**6.94E+04**	**7.20E+04**	**7.55E+04**	**7.89E+04**	**8.17E+04**	**8.48E+04**	**8.82E+04**	**9.16E+04**	**9.51E+04**	**9.85E+04**	**1.02E+05**	**1.05E+05**	**1.09E+05**	**1.12E+05**	**1.15E+05**	**1.19E+05**	**1.22E+05**	**1.25E+05**	**1.28E+05**	**1.31E+05**
4	RESLI_Urbanfl	1.51E+04	1.68E+04	1.86E+04	2.05E+04	2.24E+04	2.11E+04	2.26E+04	2.51E+04	2.81E+04	3.11E+04	3.46E+04	3.85E+04	4.28E+04	4.74E+04	5.24E+04	5.77E+04	6.34E+04	6.95E+04	7.60E+04	8.29E+04	9.02E+04	9.80E+04	1.06E+05	1.15E+05	1.24E+05
4	RESLI_Urbanmu	1.16E+04	1.26E+04	1.37E+04	1.48E+04	1.60E+04	1.65E+04	1.67E+04	1.67E+04	1.66E+04	1.62E+04	1.59E+04	1.54E+04	1.47E+04	1.39E+04	1.30E+04	1.18E+04	1.04E+04	8.82E+03	7.00E+03	4.93E+03	2.61E+03	4.96E+00	-2.88E+03	-6.08E+03	-9.59E+03
4	RESLI_Urbanri	9.51E+03	1.06E+04	1.17E+04	1.29E+04	1.41E+04	1.49E+04	1.64E+04	1.77E+04	1.92E+04	2.04E+04	2.18E+04	2.33E+04	2.50E+04	2.67E+04	2.85E+04	3.04E+04	3.23E+04	3.44E+04	3.65E+04	3.87E+04	4.09E+04	4.33E+04	4.57E+04	4.82E+04	5.08E+04
**4**	**TOTAL LI URBAN RES**	**3.62E+04**	**4.00E+04**	**4.40E+04**	**4.82E+04**	**5.26E+04**	**5.25E+04**	**5.56E+04**	**5.96E+04**	**6.39E+04**	**6.77E+04**	**7.22E+04**	**7.73E+04**	**8.26E+04**	**8.81E+04**	**9.38E+04**	**9.99E+04**	**1.06E+05**	**1.13E+05**	**1.19E+05**	**1.26E+05**	**1.34E+05**	**1.41E+05**	**1.49E+05**	**1.57E+05**	**1.65E+05**
4	RESLMI_Ruralf	1.05E+05	1.09E+05	1.12E+05	1.16E+05	1.20E+05	1.18E+05	1.21E+05	1.29E+05	1.36E+05	1.42E+05	1.49E+05	1.57E+05	1.65E+05	1.72E+05	1.80E+05	1.88E+05	1.96E+05	2.05E+05	2.13E+05	2.21E+05	2.29E+05	2.37E+05	2.45E+05	2.53E+05	2.61E+05
4	RESLMI_Ruralm	6.79E+04	7.03E+04	7.27E+04	7.51E+04	7.74E+04	7.65E+04	7.42E+04	7.14E+04	6.83E+04	6.44E+04	6.06E+04	5.67E+04	5.26E+04	4.82E+04	4.36E+04	3.88E+04	3.37E+04	2.84E+04	2.29E+04	1.73E+04	1.14E+04	5.37E+03	-8.14E+02	-7.14E+03	-1.36E+04
4	RESLMI_Ruralr	7.76E+04	8.01E+04	8.26E+04	8.50E+04	8.73E+04	8.96E+04	9.03E+04	9.16E+04	9.37E+04	9.48E+04	9.63E+04	9.81E+04	9.98E+04	1.01E+05	1.03E+05	1.04E+05	1.05E+05	1.06E+05	1.07E+05	1.08E+05	1.09E+05	1.10E+05	1.10E+05	1.11E+05	1.11E+05
**4**	**TOTAL LMI RURAL RES**	**2.50E+05**	**2.59E+05**	**2.68E+05**	**2.76E+05**	**2.84E+05**	**2.84E+05**	**2.86E+05**	**2.92E+05**	**2.98E+05**	**3.01E+05**	**3.06E+05**	**3.12E+05**	**3.17E+05**	**3.22E+05**	**3.27E+05**	**3.31E+05**	**3.36E+05**	**3.39E+05**	**3.43E+05**	**3.46E+05**	**3.49E+05**	**3.52E+05**	**3.54E+05**	**3.57E+05**	**3.58E+05**

SCENARIO NO.	SCENARIO DESCRIPTOR	2016	2017	2018	2019	2020	2021	2022	2023	2024	2025	2026	2027	2028	2029	2030	2031	2032	2033	2034	2035	2036	2037	2038	2039	2040
4	RESLMI_Urbanf	8.82E+04	9.44E+04	1.01E+05	1.07E+05	1.14E+05	1.10E+05	1.18E+05	1.30E+05	1.43E+05	1.55E+05	1.68E+05	1.83E+05	1.99E+05	2.16E+05	2.33E+05	2.51E+05	2.71E+05	2.91E+05	3.13E+05	3.35E+05	3.59E+05	3.84E+05	4.10E+05	4.37E+05	4.67E+05
4	RESLMI_Urbanm	7.48E+04	7.91E+04	8.34E+04	8.80E+04	9.26E+04	9.29E+04	9.16E+04	8.95E+04	8.69E+04	8.30E+04	7.91E+04	7.48E+04	6.99E+04	6.44E+04	5.82E+04	5.16E+04	4.45E+04	3.67E+04	2.83E+04	1.92E+04	9.49E+03	-1.01E+03	-1.24E+04	-2.47E+04	-3.81E+04
4	RESLMI_Urbanr	6.39E+04	6.81E+04	7.25E+04	7.67E+04	8.12E+04	8.32E+04	8.69E+04	8.96E+04	9.36E+04	9.64E+04	9.96E+04	1.03E+05	1.07E+05	1.11E+05	1.15E+05	1.19E+05	1.22E+05	1.25E+05	1.29E+05	1.32E+05	1.35E+05	1.38E+05	1.41E+05	1.44E+05	1.47E+05
**4**	**TOTAL LMI URBAN RES**	**2.27E+05**	**2.42E+05**	**2.57E+05**	**2.72E+05**	**2.88E+05**	**2.86E+05**	**2.96E+05**	**3.09E+05**	**3.24E+05**	**3.34E+05**	**3.47E+05**	**3.62E+05**	**3.76E+05**	**3.91E+05**	**4.06E+05**	**4.21E+05**	**4.37E+05**	**4.53E+05**	**4.70E+05**	**4.86E+05**	**5.03E+05**	**5.21E+05**	**5.39E+05**	**5.57E+05**	**5.76E+05**
4	RESUMI_Ruralf	9.88E+04	1.02E+05	1.04E+05	1.06E+05	1.09E+05	1.08E+05	1.11E+05	1.17E+05	1.22E+05	1.26E+05	1.31E+05	1.37E+05	1.42E+05	1.47E+05	1.53E+05	1.58E+05	1.63E+05	1.69E+05	1.74E+05	1.79E+05	1.84E+05	1.90E+05	1.95E+05	2.01E+05	2.06E+05
4	RESUMI_Ruralm	5.49E+04	5.65E+04	5.78E+04	5.92E+04	6.04E+04	5.90E+04	5.62E+04	5.31E+04	4.99E+04	4.62E+04	4.25E+04	3.89E+04	3.52E+04	3.14E+04	2.74E+04	2.34E+04	1.93E+04	1.51E+04	1.08E+04	6.46E+03	2.06E+03	-2.40E+03	-6.91E+03	-1.15E+04	-1.61E+04
4	RESUMI_Ruralr	7.34E+04	7.52E+04	7.67E+04	7.82E+04	7.96E+04	8.08E+04	8.18E+04	8.28E+04	8.37E+04	8.39E+04	8.42E+04	8.49E+04	8.55E+04	8.60E+04	8.64E+04	8.67E+04	8.68E+04	8.69E+04	8.69E+04	8.70E+04	8.69E+04	8.69E+04	8.68E+04	8.66E+04	8.65E+04
**4**	**TOTAL UMI RURAL RES**	**2.27E+05**	**2.33E+05**	**2.39E+05**	**2.44E+05**	**2.49E+05**	**2.48E+05**	**2.49E+05**	**2.53E+05**	**2.56E+05**	**2.56E+05**	**2.58E+05**	**2.60E+05**	**2.63E+05**	**2.65E+05**	**2.66E+05**	**2.68E+05**	**2.69E+05**	**2.70E+05**	**2.72E+05**	**2.73E+05**	**2.73E+05**	**2.74E+05**	**2.75E+05**	**2.76E+05**	**2.76E+05**
4	RESUMI_Urbanf	2.21E+05	2.39E+05	2.56E+05	2.74E+05	2.92E+05	2.94E+05	3.15E+05	3.44E+05	3.74E+05	4.02E+05	4.32E+05	4.65E+05	4.99E+05	5.35E+05	5.72E+05	6.09E+05	6.46E+05	6.85E+05	7.24E+05	7.64E+05	8.05E+05	8.47E+05	8.90E+05	9.34E+05	9.79E+05
4	RESUMI_Urbanm	1.38E+05	1.49E+05	1.59E+05	1.69E+05	1.80E+05	1.77E+05	1.74E+05	1.70E+05	1.64E+05	1.56E+05	1.47E+05	1.38E+05	1.27E+05	1.15E+05	1.02E+05	8.71E+04	7.14E+04	5.44E+04	3.61E+04	1.65E+04	-4.38E+03	-2.65E+04	-4.98E+04	-7.43E+04	-1.00E+05
4	RESUMI_Urbanr	1.35E+05	1.46E+05	1.57E+05	1.68E+05	1.80E+05	1.84E+05	1.97E+05	2.06E+05	2.16E+05	2.23E+05	2.31E+05	2.39E+05	2.48E+05	2.56E+05	2.64E+05	2.71E+05	2.78E+05	2.84E+05	2.90E+05	2.95E+05	3.00E+05	3.04E+05	3.08E+05	3.12E+05	3.15E+05
**4**	**TOTAL UMI URBAN RES**	**4.94E+05**	**5.34E+05**	**5.73E+05**	**6.12E+05**	**6.52E+05**	**6.55E+05**	**6.86E+05**	**7.20E+05**	**7.54E+05**	**7.80E+05**	**8.09E+05**	**8.42E+05**	**8.74E+05**	**9.06E+05**	**9.38E+05**	**9.67E+05**	**9.95E+05**	**1.02E+06**	**1.05E+06**	**1.08E+06**	**1.10E+06**	**1.13E+06**	**1.15E+06**	**1.17E+06**	**1.19E+06**
**4**	**TOTAL GLOBAL RES ANNUAL**	**2.14E+06**	**2.24E+06**	**2.32E+06**	**2.42E+06**	**2.51E+06**	**2.54E+06**	**2.56E+06**	**2.63E+06**	**2.71E+06**	**2.77E+06**	**2.83E+06**	**2.90E+06**	**2.97E+06**	**3.04E+06**	**3.11E+06**	**3.18E+06**	**3.25E+06**	**3.32E+06**	**3.39E+06**	**3.45E+06**	**3.51E+06**	**3.58E+06**	**3.64E+06**	**3.70E+06**	**3.76E+06**
**4**	**TOTAL GLOBAL**	**2.14E+06**	**4.38E+06**	**6.70E+06**	**9.12E+06**	**1.16E+07**	**1.42E+07**	**1.67E+07**	**1.94E+07**	**2.21E+07**	**2.48E+07**	**2.77E+07**	**3.06E+07**	**3.35E+07**	**3.66E+07**	**3.97E+07**	**4.29E+07**	**4.61E+07**	**4.95E+07**	**5.28E+07**	**5.63E+07**	**5.98E+07**	**6.34E+07**	**6.70E+07**	**7.07E+07**	**7.45E+07**

SCENARIO NO.	SCENARIO DESCRIPTOR	2016	2017	2018	2019	2020	2021	2022	2023	2024	2025	2026	2027	2028	2029	2030	2031	2032	2033	2034	2035	2036	2037	2038	2039	2040
	**RES CUMULATIVE**																									
5	RSSHI_Ruralfl	3.71E+04	3.75E+04	3.78E+04	3.81E+04	3.85E+04	3.96E+04	3.80E+04	3.74E+04	3.69E+04	3.72E+04	3.72E+04	3.65E+04	3.58E+04	3.50E+04	3.43E+04	3.32E+04	3.20E+04	3.08E+04	2.96E+04	2.84E+04	2.72E+04	2.59E+04	2.47E+04	2.34E+04	2.22E+04
5	RSSHI_Ruralmu	3.90E+04	3.96E+04	3.99E+04	4.07E+04	4.12E+04	4.29E+04	4.17E+04	4.18E+04	4.19E+04	4.09E+04	4.03E+04	4.03E+04	4.04E+04	4.04E+04	4.03E+04	4.01E+04	3.97E+04	3.92E+04	3.88E+04	3.83E+04	3.78E+04	3.72E+04	3.67E+04	3.61E+04	3.55E+04
5	RSSHI_Ruralri	8.53E+04	8.59E+04	8.60E+04	8.66E+04	8.67E+04	8.78E+04	8.46E+04	8.31E+04	8.15E+04	7.99E+04	7.82E+04	7.65E+04	7.48E+04	7.30E+04	7.12E+04	6.92E+04	6.70E+04	6.49E+04	6.27E+04	6.05E+04	5.83E+04	5.61E+04	5.40E+04	5.18E+04	4.96E+04
**5**	**TOTAL HI RURAL RSS**	**1.61E+05**	**1.63E+05**	**1.64E+05**	**1.65E+05**	**1.66E+05**	**1.70E+05**	**1.64E+05**	**1.62E+05**	**1.60E+05**	**1.58E+05**	**1.56E+05**	**1.53E+05**	**1.51E+05**	**1.48E+05**	**1.46E+05**	**1.42E+05**	**1.39E+05**	**1.35E+05**	**1.31E+05**	**1.27E+05**	**1.23E+05**	**1.19E+05**	**1.15E+05**	**1.11E+05**	**1.07E+05**
5	RSSHI_Urbanfl	1.58E+05	1.62E+05	1.65E+05	1.68E+05	1.72E+05	1.79E+05	1.74E+05	1.74E+05	1.73E+05	1.78E+05	1.80E+05	1.80E+05	1.79E+05	1.78E+05	1.77E+05	1.74E+05	1.71E+05	1.67E+05	1.64E+05	1.60E+05	1.56E+05	1.51E+05	1.47E+05	1.42E+05	1.37E+05
5	RSSHI_Urbanmu	1.65E+05	1.69E+05	1.72E+05	1.77E+05	1.82E+05	1.93E+05	1.89E+05	1.93E+05	1.96E+05	1.94E+05	1.94E+05	1.97E+05	2.00E+05	2.03E+05	2.07E+05	2.09E+05	2.10E+05	2.12E+05	2.13E+05	2.14E+05	2.15E+05	2.16E+05	2.17E+05	2.17E+05	2.18E+05
5	RSSHI_Urbanri	3.72E+05	3.80E+05	3.88E+05	3.94E+05	4.01E+05	4.17E+05	3.98E+05	3.95E+05	3.93E+05	3.81E+05	3.78E+05	3.75E+05	3.72E+05	3.68E+05	3.65E+05	3.61E+05	3.55E+05	3.50E+05	3.45E+05	3.39E+05	3.33E+05	3.27E+05	3.20E+05	3.13E+05	3.06E+05
**5**	**TOTAL HI URBAN RSS**	**6.95E+05**	**7.11E+05**	**7.25E+05**	**7.40E+05**	**7.54E+05**	**7.89E+05**	**7.61E+05**	**7.62E+05**	**7.62E+05**	**7.52E+05**	**7.52E+05**	**7.51E+05**	**7.51E+05**	**7.50E+05**	**7.48E+05**	**7.43E+05**	**7.37E+05**	**7.29E+05**	**7.21E+05**	**7.12E+05**	**7.03E+05**	**6.94E+05**	**6.84E+05**	**6.73E+05**	**6.62E+05**
5	RSSLI_Ruralfl	2.33E+04	2.48E+04	2.64E+04	2.80E+04	2.96E+04	3.10E+04	3.22E+04	3.33E+04	3.44E+04	3.82E+04	4.09E+04	4.20E+04	4.29E+04	4.39E+04	4.47E+04	4.51E+04	4.53E+04	4.53E+04	4.53E+04	4.52E+04	4.49E+04	4.46E+04	4.41E+04	4.36E+04	4.29E+04
5	RSSLI_Ruralmu	1.30E+04	1.38E+04	1.47E+04	1.56E+04	1.65E+04	1.72E+04	1.79E+04	1.85E+04	1.92E+04	1.67E+04	1.55E+04	1.59E+04	1.64E+04	1.69E+04	1.73E+04	1.75E+04	1.77E+04	1.78E+04	1.78E+04	1.78E+04	1.78E+04	1.78E+04	1.77E+04	1.76E+04	1.74E+04
5	RSSLI_Ruralri	1.72E+04	1.83E+04	1.94E+04	2.05E+04	2.16E+04	2.25E+04	2.34E+04	2.41E+04	2.49E+04	2.56E+04	2.63E+04	2.70E+04	2.76E+04	2.82E+04	2.88E+04	2.92E+04	2.95E+04	2.97E+04	2.99E+04	3.01E+04	3.02E+04	3.02E+04	3.02E+04	3.01E+04	3.00E+04
**5**	**TOTAL LI RURAL RSS**	**5.35E+04**	**5.70E+04**	**6.05E+04**	**6.41E+04**	**6.77E+04**	**7.08E+04**	**7.34E+04**	**7.59E+04**	**7.84E+04**	**8.05E+04**	**8.27E+04**	**8.49E+04**	**8.70E+04**	**8.89E+04**	**9.08E+04**	**9.18E+04**	**9.24E+04**	**9.28E+04**	**9.30E+04**	**9.31E+04**	**9.29E+04**	**9.26E+04**	**9.20E+04**	**9.13E+04**	**9.04E+04**
5	RSSLI_Urbanfl	1.52E+04	1.68E+04	1.86E+04	2.05E+04	2.25E+04	2.22E+04	2.37E+04	2.52E+04	2.67E+04	3.09E+04	3.45E+04	3.63E+04	3.81E+04	4.00E+04	4.19E+04	4.34E+04	4.47E+04	4.60E+04	4.72E+04	4.84E+04	4.96E+04	5.08E+04	5.19E+04	5.29E+04	5.39E+04
5	RSSLI_Urbanmu	1.15E+04	1.26E+04	1.37E+04	1.48E+04	1.60E+04	1.70E+04	1.81E+04	1.91E+04	2.02E+04	1.80E+04	1.70E+04	1.79E+04	1.89E+04	1.99E+04	2.09E+04	2.17E+04	2.24E+04	2.30E+04	2.37E+04	2.43E+04	2.49E+04	2.54E+04	2.59E+04	2.63E+04	2.67E+04
5	RSSLI_Urbanri	9.55E+03	1.06E+04	1.18E+04	1.29E+04	1.41E+04	1.49E+04	1.61E+04	1.72E+04	1.82E+04	1.93E+04	2.04E+04	2.14E+04	2.25E+04	2.36E+04	2.48E+04	2.57E+04	2.65E+04	2.73E+04	2.81E+04	2.88E+04	2.95E+04	3.01E+04	3.07E+04	3.12E+04	3.17E+04
**5**	**TOTAL LI URBAN RSS**	**3.62E+04**	**4.00E+04**	**4.40E+04**	**4.82E+04**	**5.26E+04**	**5.41E+04**	**5.79E+04**	**6.15E+04**	**6.52E+04**	**6.82E+04**	**7.19E+04**	**7.57E+04**	**7.96E+04**	**8.35E+04**	**8.75E+04**	**9.08E+04**	**9.36E+04**	**9.63E+04**	**9.90E+04**	**1.02E+05**	**1.04E+05**	**1.06E+05**	**1.08E+05**	**1.10E+05**	**1.12E+05**

SCENARIO NO.	SCENARIO DESCRIPTOR	2016	2017	2018	2019	2020	2021	2022	2023	2024	2025	2026	2027	2028	2029	2030	2031	2032	2033	2034	2035	2036	2037	2038	2039	2040
5	RSSLMI_Ruralf	1.05E+05	1.08E+05	1.12E+05	1.16E+05	1.19E+05	1.22E+05	1.24E+05	1.26E+05	1.27E+05	1.39E+05	1.46E+05	1.47E+05	1.48E+05	1.49E+05	1.49E+05	1.48E+05	1.46E+05	1.44E+05	1.42E+05	1.39E+05	1.37E+05	1.34E+05	1.30E+05	1.27E+05	1.24E+05
5	RSSLMI_Ruralm	6.78E+04	7.02E+04	7.26E+04	7.50E+04	7.73E+04	7.88E+04	8.00E+04	8.10E+04	8.20E+04	7.00E+04	6.41E+04	6.48E+04	6.54E+04	6.60E+04	6.64E+04	6.61E+04	6.54E+04	6.46E+04	6.37E+04	6.27E+04	6.16E+04	6.05E+04	5.92E+04	5.78E+04	5.64E+04
5	RSSLMI_Ruralr	7.76E+04	8.01E+04	8.25E+04	8.49E+04	8.73E+04	8.90E+04	9.01E+04	9.11E+04	9.20E+04	9.28E+04	9.35E+04	9.41E+04	9.45E+04	9.49E+04	9.51E+04	9.48E+04	9.41E+04	9.33E+04	9.24E+04	9.14E+04	9.03E+04	8.90E+04	8.77E+04	8.62E+04	8.47E+04
**5**	**TOTAL LMI RURAL RSS**	**2.50E+05**	**2.59E+05**	**2.67E+05**	**2.76E+05**	**2.84E+05**	**2.90E+05**	**2.94E+05**	**2.98E+05**	**3.01E+05**	**3.02E+05**	**3.04E+05**	**3.06E+05**	**3.08E+05**	**3.10E+05**	**3.11E+05**	**3.09E+05**	**3.06E+05**	**3.02E+05**	**2.98E+05**	**2.93E+05**	**2.89E+05**	**2.83E+05**	**2.77E+05**	**2.71E+05**	**2.65E+05**
5	RSSLMI_Urbanf	8.80E+04	9.42E+04	1.01E+05	1.07E+05	1.14E+05	1.17E+05	1.23E+05	1.28E+05	1.33E+05	1.53E+05	1.69E+05	1.74E+05	1.79E+05	1.84E+05	1.90E+05	1.92E+05	1.93E+05	1.95E+05	1.96E+05	1.96E+05	1.97E+05	1.97E+05	1.97E+05	1.98E+05	1.98E+05
5	RSSLMI_Urbanm	7.47E+04	7.90E+04	8.34E+04	8.79E+04	9.25E+04	9.60E+04	9.96E+04	1.03E+05	1.06E+05	9.27E+04	8.54E+04	8.83E+04	9.13E+04	9.42E+04	9.72E+04	9.92E+04	1.01E+05	1.02E+05	1.03E+05	1.04E+05	1.05E+05	1.06E+05	1.06E+05	1.06E+05	1.06E+05
5	RSSLMI_Urbanr	6.42E+04	6.83E+04	7.26E+04	7.69E+04	8.15E+04	8.43E+04	8.89E+04	9.22E+04	9.56E+04	9.90E+04	1.02E+05	1.06E+05	1.09E+05	1.12E+05	1.15E+05	1.17E+05	1.19E+05	1.20E+05	1.22E+05	1.23E+05	1.24E+05	1.24E+05	1.25E+05	1.25E+05	1.25E+05
**5**	**TOTAL LMI URBAN RSS**	**2.27E+05**	**2.41E+05**	**2.57E+05**	**2.72E+05**	**2.88E+05**	**2.97E+05**	**3.12E+05**	**3.23E+05**	**3.35E+05**	**3.45E+05**	**3.56E+05**	**3.68E+05**	**3.79E+05**	**3.91E+05**	**4.02E+05**	**4.08E+05**	**4.13E+05**	**4.17E+05**	**4.20E+05**	**4.23E+05**	**4.25E+05**	**4.27E+05**	**4.28E+05**	**4.29E+05**	**4.29E+05**
5	RSSUMI_Ruralf	9.85E+04	1.01E+05	1.04E+05	1.06E+05	1.08E+05	1.10E+05	1.10E+05	1.10E+05	1.11E+05	1.19E+05	1.25E+05	1.24E+05	1.24E+05	1.23E+05	1.22E+05	1.20E+05	1.17E+05	1.15E+05	1.12E+05	1.09E+05	1.06E+05	1.03E+05	1.00E+05	9.69E+04	9.37E+04
5	RSSUMI_Ruralm	5.49E+04	5.64E+04	5.78E+04	5.91E+04	6.04E+04	6.09E+04	6.11E+04	6.13E+04	6.13E+04	5.19E+04	4.60E+04	4.61E+04	4.61E+04	4.61E+04	4.60E+04	4.55E+04	4.46E+04	4.38E+04	4.29E+04	4.20E+04	4.10E+04	4.00E+04	3.90E+04	3.80E+04	3.69E+04
5	RSSUMI_Ruralr	7.34E+04	7.51E+04	7.67E+04	7.82E+04	7.96E+04	8.02E+04	8.03E+04	8.04E+04	8.03E+04	8.01E+04	7.98E+04	7.94E+04	7.89E+04	7.84E+04	7.78E+04	7.69E+04	7.56E+04	7.43E+04	7.30E+04	7.16E+04	7.02E+04	6.87E+04	6.73E+04	6.58E+04	6.42E+04
**5**	**TOTAL UMI RURAL RSS**	**2.27E+05**	**2.33E+05**	**2.38E+05**	**2.43E+05**	**2.48E+05**	**2.51E+05**	**2.52E+05**	**2.52E+05**	**2.52E+05**	**2.51E+05**	**2.50E+05**	**2.50E+05**	**2.49E+05**	**2.47E+05**	**2.46E+05**	**2.42E+05**	**2.38E+05**	**2.33E+05**	**2.28E+05**	**2.23E+05**	**2.17E+05**	**2.12E+05**	**2.06E+05**	**2.01E+05**	**1.95E+05**
5	RSSUMI_Urbanf	2.20E+05	2.38E+05	2.55E+05	2.73E+05	2.91E+05	3.01E+05	3.15E+05	3.28E+05	3.41E+05	3.84E+05	4.19E+05	4.32E+05	4.45E+05	4.57E+05	4.69E+05	4.75E+05	4.79E+05	4.82E+05	4.85E+05	4.86E+05	4.85E+05	4.84E+05	4.82E+05	4.78E+05	4.74E+05
5	RSSUMI_Urbanm	1.38E+05	1.49E+05	1.59E+05	1.69E+05	1.80E+05	1.84E+05	1.91E+05	1.98E+05	2.05E+05	1.79E+05	1.63E+05	1.68E+05	1.74E+05	1.79E+05	1.85E+05	1.89E+05	1.91E+05	1.93E+05	1.94E+05	1.96E+05	1.97E+05	1.97E+05	1.97E+05	1.97E+05	1.97E+05
5	RSSUMI_Urbanr	1.36E+05	1.47E+05	1.58E+05	1.69E+05	1.81E+05	1.87E+05	1.97E+05	2.05E+05	2.14E+05	2.42E+05	2.51E+05	2.59E+05	2.66E+05	2.74E+05	2.81E+05	2.87E+05	2.91E+05	2.94E+05	2.97E+05	3.00E+05	3.02E+05	3.04E+05	3.05E+05	3.05E+05	3.06E+05
**5**	**TOTAL UMI**	**4.94E+05**	**5.34E+05**	**5.73E+05**	**6.12E+05**	**6.52E+05**	**6.71E+05**	**7.02E+05**	**7.31E+05**	**7.60E+05**	**8.05E+05**	**8.33E+05**	**8.59E+05**	**8.85E+05**	**9.11E+05**	**9.35E+05**	**9.51E+05**	**9.61E+05**	**9.69E+05**	**9.76E+05**	**9.81E+05**	**9.84E+05**	**9.85E+05**	**9.84E+05**	**9.81E+05**	**9.76E+05**

SCENARIO NO.	SCENARIO DESCRIPTOR	2016	2017	2018	2019	2020	2021	2022	2023	2024	2025	2026	2027	2028	2029	2030	2031	2032	2033	2034	2035	2036	2037	2038	2039	2040
	**URBAN RSS**																									
**5**	**TOTAL GLOBAL RSS ANNUAL**	**2.14E+06**	**2.24E+06**	**2.33E+06**	**2.42E+06**	**2.51E+06**	**2.59E+06**	**2.62E+06**	**2.67E+06**	**2.71E+06**	**2.76E+06**	**2.81E+06**	**2.85E+06**	**2.89E+06**	**2.93E+06**	**2.97E+06**	**2.98E+06**	**2.98E+06**	**2.97E+06**	**2.97E+06**	**2.95E+06**	**2.94E+06**	**2.92E+06**	**2.89E+06**	**2.87E+06**	**2.84E+06**
**5**	**TOTAL GLOBAL RSS CUMULATIVE**	**2.14E+06**	**4.38E+06**	**6.71E+06**	**9.13E+06**	**1.16E+07**	**1.42E+07**	**1.69E+07**	**1.95E+07**	**2.22E+07**	**2.50E+07**	**2.78E+07**	**3.06E+07**	**3.35E+07**	**3.65E+07**	**3.94E+07**	**4.24E+07**	**4.54E+07**	**4.84E+07**	**5.13E+07**	**5.43E+07**	**5.72E+07**	**6.01E+07**	**6.30E+07**	**6.59E+07**	**6.87E+07**
6	SCSHI_Ruralfl	3.72E+04	3.77E+04	3.81E+04	3.84E+04	3.87E+04	3.87E+04	3.81E+04	3.77E+04	3.73E+04	3.67E+04	3.62E+04	3.56E+04	3.50E+04	3.43E+04	3.36E+04	3.25E+04	3.12E+04	2.98E+04	2.85E+04	2.71E+04	2.58E+04	2.44E+04	2.30E+04	2.17E+04	2.03E+04
6	SCSHI_Ruralmu	3.86E+04	3.94E+04	4.00E+04	4.05E+04	4.09E+04	4.11E+04	4.06E+04	4.03E+04	4.00E+04	3.97E+04	3.94E+04	3.91E+04	3.88E+04	3.85E+04	3.81E+04	3.78E+04	3.73E+04	3.68E+04	3.63E+04	3.58E+04	3.53E+04	3.48E+04	3.42E+04	3.37E+04	3.31E+04
6	SCSHI_Ruralri	8.53E+04	8.59E+04	8.64E+04	8.67E+04	8.69E+04	8.60E+04	8.41E+04	8.24E+04	8.07E+04	7.89E+04	7.71E+04	7.52E+04	7.33E+04	7.13E+04	6.92E+04	6.72E+04	6.51E+04	6.29E+04	6.07E+04	5.85E+04	5.63E+04	5.41E+04	5.18E+04	4.96E+04	4.74E+04
**6**	**TOTAL HI RURAL SCS**	**1.61E+05**	**1.63E+05**	**1.65E+05**	**1.66E+05**	**1.66E+05**	**1.66E+05**	**1.63E+05**	**1.60E+05**	**1.58E+05**	**1.55E+05**	**1.53E+05**	**1.50E+05**	**1.47E+05**	**1.44E+05**	**1.41E+05**	**1.37E+05**	**1.34E+05**	**1.30E+05**	**1.26E+05**	**1.21E+05**	**1.17E+05**	**1.13E+05**	**1.09E+05**	**1.05E+05**	**1.01E+05**
6	SCSHI_Urbanfl	1.58E+05	1.62E+05	1.66E+05	1.69E+05	1.73E+05	1.76E+05	1.74E+05	1.75E+05	1.75E+05	1.75E+05	1.74E+05	1.74E+05	1.73E+05	1.72E+05	1.70E+05	1.67E+05	1.63E+05	1.58E+05	1.53E+05	1.48E+05	1.43E+05	1.38E+05	1.33E+05	1.28E+05	1.23E+05
6	SCSHI_Urbanmu	1.63E+05	1.68E+05	1.73E+05	1.77E+05	1.81E+05	1.86E+05	1.85E+05	1.86E+05	1.87E+05	1.88E+05	1.89E+05	1.90E+05	1.91E+05	1.92E+05	1.93E+05	1.94E+05	1.95E+05	1.95E+05	1.96E+05	1.96E+05	1.97E+05	1.98E+05	1.98E+05	1.99E+05	2.00E+05
6	SCSHI_Urbanri	3.72E+05	3.81E+05	3.89E+05	3.95E+05	4.01E+05	4.04E+05	3.95E+05	3.93E+05	3.90E+05	3.87E+05	3.83E+05	3.79E+05	3.75E+05	3.70E+05	3.65E+05	3.61E+05	3.55E+05	3.49E+05	3.43E+05	3.37E+05	3.30E+05	3.23E+05	3.16E+05	3.08E+05	3.00E+05
**6**	**TOTAL HI URBAN SCS**	**6.93E+05**	**7.10E+05**	**7.27E+05**	**7.41E+05**	**7.55E+05**	**7.65E+05**	**7.54E+05**	**7.53E+05**	**7.51E+05**	**7.49E+05**	**7.47E+05**	**7.43E+05**	**7.39E+05**	**7.34E+05**	**7.29E+05**	**7.22E+05**	**7.13E+05**	**7.03E+05**	**6.92E+05**	**6.82E+05**	**6.70E+05**	**6.58E+05**	**6.47E+05**	**6.35E+05**	**6.23E+05**
6	SCSLI_Ruralfl	2.33E+04	2.49E+04	2.64E+04	2.80E+04	2.96E+04	3.15E+04	3.36E+04	3.57E+04	3.78E+04	3.99E+04	4.19E+04	4.39E+04	4.58E+04	4.77E+04	4.96E+04	5.08E+04	5.16E+04	5.24E+04	5.29E+04	5.34E+04	5.36E+04	5.37E+04	5.36E+04	5.33E+04	5.28E+04
6	SCSLI_Ruralmu	1.29E+04	1.38E+04	1.46E+04	1.55E+04	1.64E+04	1.64E+04	1.59E+04	1.53E+04	1.47E+04	1.40E+04	1.32E+04	1.24E+04	1.15E+04	1.07E+04	9.75E+03	8.85E+03	7.95E+03	7.07E+03	6.21E+03	5.39E+03	4.62E+03	3.90E+03	3.24E+03	2.66E+03	2.17E+03
6	SCSLI_Ruralri	1.72E+04	1.83E+04	1.94E+04	2.05E+04	2.16E+04	2.25E+04	2.33E+04	2.41E+04	2.48E+04	2.55E+04	2.62E+04	2.68E+04	2.74E+04	2.79E+04	2.84E+04	2.88E+04	2.91E+04	2.93E+04	2.95E+04	2.96E+04	2.96E+04	2.96E+04	2.96E+04	2.95E+04	2.93E+04
**6**	**TOTAL LI RURAL SCS**	**5.35E+04**	**5.70E+04**	**6.05E+04**	**6.41E+04**	**6.77E+04**	**7.04E+04**	**7.29E+04**	**7.52E+04**	**7.73E+04**	**7.93E+04**	**8.13E+04**	**8.31E+04**	**8.47E+04**	**8.63E+04**	**8.77E+04**	**8.84E+04**	**8.87E+04**	**8.87E+04**	**8.86E+04**	**8.83E+04**	**8.79E+04**	**8.72E+04**	**8.64E+04**	**8.54E+04**	**8.43E+04**

SCENARIO NO.	SCENARIO DESCRIPTOR	2016	2017	2018	2019	2020	2021	2022	2023	2024	2025	2026	2027	2028	2029	2030	2031	2032	2033	2034	2035	2036	2037	2038	2039	2040
6	SCSLI_Urbanfl	1.51E+04	1.68E+04	1.86E+04	2.05E+04	2.25E+04	2.25E+04	2.46E+04	2.68E+04	2.91E+04	3.15E+04	3.41E+04	3.67E+04	3.94E+04	4.22E+04	4.50E+04	4.72E+04	4.91E+04	5.09E+04	5.27E+04	5.43E+04	5.58E+04	5.72E+04	5.85E+04	5.97E+04	6.09E+04
6	SCSLI_Urbanmu	1.15E+04	1.25E+04	1.36E+04	1.47E+04	1.59E+04	1.61E+04	1.60E+04	1.57E+04	1.54E+04	1.49E+04	1.44E+04	1.37E+04	1.30E+04	1.22E+04	1.13E+04	1.04E+04	9.48E+03	8.52E+03	7.55E+03	6.59E+03	5.64E+03	4.73E+03	3.88E+03	3.11E+03	2.43E+03
6	SCSLI_Urbanri	9.46E+03	1.06E+04	1.17E+04	1.29E+04	1.41E+04	1.47E+04	1.58E+04	1.68E+04	1.77E+04	1.86E+04	1.95E+04	2.04E+04	2.13E+04	2.21E+04	2.30E+04	2.37E+04	2.45E+04	2.52E+04	2.59E+04	2.65E+04	2.71E+04	2.76E+04	2.81E+04	2.85E+04	2.88E+04
**6**	**TOTAL LI URBAN SCS**	**3.61E+04**	**3.99E+04**	**4.39E+04**	**4.81E+04**	**5.25E+04**	**5.33E+04**	**5.64E+04**	**5.93E+04**	**6.21E+04**	**6.50E+04**	**6.79E+04**	**7.08E+04**	**7.37E+04**	**7.65E+04**	**7.92E+04**	**8.14E+04**	**8.31E+04**	**8.47E+04**	**8.61E+04**	**8.74E+04**	**8.85E+04**	**8.96E+04**	**9.05E+04**	**9.13E+04**	**9.21E+04**
6	SCSLMI_Ruralf	1.05E+05	1.09E+05	1.13E+05	1.16E+05	1.20E+05	1.25E+05	1.30E+05	1.36E+05	1.41E+05	1.46E+05	1.50E+05	1.55E+05	1.59E+05	1.63E+05	1.66E+05	1.67E+05	1.68E+05	1.67E+05	1.67E+05	1.65E+05	1.64E+05	1.62E+05	1.59E+05	1.56E+05	1.53E+05
6	SCSLMI_Ruralm	6.74E+04	6.98E+04	7.22E+04	7.46E+04	7.69E+04	7.49E+04	7.12E+04	6.72E+04	6.31E+04	5.89E+04	5.47E+04	5.04E+04	4.62E+04	4.20E+04	3.79E+04	3.39E+04	3.01E+04	2.65E+04	2.31E+04	1.99E+04	1.70E+04	1.43E+04	1.19E+04	9.84E+03	8.08E+03
6	SCSLMI_Ruralr	7.77E+04	8.02E+04	8.26E+04	8.51E+04	8.74E+04	8.89E+04	8.99E+04	9.08E+04	9.15E+04	9.21E+04	9.25E+04	9.28E+04	9.30E+04	9.30E+04	9.30E+04	9.24E+04	9.15E+04	9.04E+04	8.92E+04	8.79E+04	8.65E+04	8.49E+04	8.33E+04	8.15E+04	7.96E+04
**6**	**TOTAL LMI RURAL SCS**	**2.50E+05**	**2.59E+05**	**2.67E+05**	**2.76E+05**	**2.84E+05**	**2.89E+05**	**2.91E+05**	**2.94E+05**	**2.95E+05**	**2.97E+05**	**2.97E+05**	**2.98E+05**	**2.98E+05**	**2.98E+05**	**2.97E+05**	**2.94E+05**	**2.89E+05**	**2.84E+05**	**2.79E+05**	**2.73E+05**	**2.67E+05**	**2.61E+05**	**2.54E+05**	**2.47E+05**	**2.40E+05**
6	SCSLMI_Urbanf	8.81E+04	9.43E+04	1.01E+05	1.07E+05	1.14E+05	1.19E+05	1.30E+05	1.39E+05	1.49E+05	1.58E+05	1.68E+05	1.78E+05	1.87E+05	1.97E+05	2.07E+05	2.15E+05	2.22E+05	2.30E+05	2.38E+05	2.47E+05	2.57E+05	2.67E+05	2.77E+05	2.87E+05	2.95E+05
6	SCSLMI_Urbanm	7.44E+04	7.86E+04	8.30E+04	8.75E+04	9.21E+04	9.10E+04	8.79E+04	8.41E+04	8.01E+04	7.57E+04	7.11E+04	6.63E+04	6.13E+04	5.60E+04	5.07E+04	4.59E+04	4.12E+04	3.68E+04	3.26E+04	2.87E+04	2.49E+04	2.14E+04	1.81E+04	1.52E+04	1.27E+04
6	SCSLMI_Urbanr	6.37E+04	6.79E+04	7.22E+04	7.65E+04	8.10E+04	8.28E+04	8.59E+04	8.79E+04	8.99E+04	9.18E+04	9.35E+04	9.52E+04	9.68E+04	9.82E+04	9.96E+04	1.00E+05	1.01E+05	1.01E+05	1.01E+05	1.01E+05	1.01E+05	1.00E+05	1.00E+05	9.95E+04	9.89E+04
**6**	**TOTAL LMI URBAN SCS**	**2.26E+05**	**2.41E+05**	**2.56E+05**	**2.71E+05**	**2.87E+05**	**2.92E+05**	**3.03E+05**	**3.11E+05**	**3.19E+05**	**3.26E+05**	**3.33E+05**	**3.39E+05**	**3.45E+05**	**3.51E+05**	**3.57E+05**	**3.61E+05**	**3.64E+05**	**3.67E+05**	**3.71E+05**	**3.77E+05**	**3.82E+05**	**3.89E+05**	**3.95E+05**	**4.01E+05**	**4.06E+05**
6	SCSUMI_Ruralf	9.90E+04	1.02E+05	1.04E+05	1.07E+05	1.09E+05	1.12E+05	1.16E+05	1.19E+05	1.23E+05	1.26E+05	1.29E+05	1.31E+05	1.33E+05	1.35E+05	1.37E+05	1.37E+05	1.36E+05	1.35E+05	1.33E+05	1.31E+05	1.29E+05	1.27E+05	1.24E+05	1.21E+05	1.18E+05
6	SCSUMI_Ruralm	5.45E+04	5.61E+04	5.74E+04	5.88E+04	5.99E+04	5.79E+04	5.42E+04	5.05E+04	4.67E+04	4.30E+04	3.93E+04	3.57E+04	3.22E+04	2.89E+04	2.56E+04	2.26E+04	1.97E+04	1.70E+04	1.45E+04	1.22E+04	1.02E+04	8.40E+03	6.83E+03	5.50E+03	4.41E+03
6	SCSUMI_Ruralr	7.35E+04	7.53E+04	7.68E+04	7.83E+04	7.97E+04	8.02E+04	8.02E+04	8.01E+04	7.99E+04	7.96E+04	7.92E+04	7.87E+04	7.81E+04	7.75E+04	7.67E+04	7.56E+04	7.42E+04	7.27E+04	7.12E+04	6.96E+04	6.81E+04	6.64E+04	6.48E+04	6.31E+04	6.14E+04
**6**	**TOTAL UMI RURAL SCS**	**2.27E+05**	**2.33E+05**	**2.38E+05**	**2.44E+05**	**2.48E+05**	**2.50E+05**	**2.50E+05**	**2.50E+05**	**2.49E+05**	**2.48E+05**	**2.47E+05**	**2.45E+05**	**2.44E+05**	**2.42E+05**	**2.39E+05**	**2.35E+05**	**2.30E+05**	**2.24E+05**	**2.19E+05**	**2.13E+05**	**2.07E+05**	**2.02E+05**	**1.96E+05**	**1.90E+05**	**1.84E+05**

SCENARIO NO.	SCENARIO DESCRIPTOR	2016	2017	2018	2019	2020	2021	2022	2023	2024	2025	2026	2027	2028	2029	2030	2031	2032	2033	2034	2035	2036	2037	2038	2039	2040
6	SCSUMI_Urbanf	2.20E+05	2.39E+05	2.56E+05	2.74E+05	2.92E+05	3.06E+05	3.29E+05	3.52E+05	3.75E+05	3.98E+05	4.21E+05	4.43E+05	4.65E+05	4.87E+05	5.08E+05	5.22E+05	5.32E+05	5.41E+05	5.49E+05	5.56E+05	5.61E+05	5.67E+05	5.72E+05	5.78E+05	5.82E+05
6	SCSUMI_Urbanm	1.37E+05	1.48E+05	1.58E+05	1.68E+05	1.78E+05	1.74E+05	1.68E+05	1.60E+05	1.52E+05	1.44E+05	1.34E+05	1.25E+05	1.15E+05	1.04E+05	9.37E+04	8.40E+04	7.45E+04	6.53E+04	5.65E+04	4.83E+04	4.09E+04	3.44E+04	2.87E+04	2.38E+04	1.97E+04
6	SCSUMI_Urbanr	1.34E+05	1.46E+05	1.57E+05	1.68E+05	1.80E+05	1.85E+05	1.92E+05	1.98E+05	2.04E+05	2.09E+05	2.15E+05	2.19E+05	2.24E+05	2.28E+05	2.32E+05	2.34E+05	2.34E+05	2.35E+05	2.34E+05	2.34E+05	2.33E+05	2.31E+05	2.29E+05	2.27E+05	2.25E+05
**6**	**TOTAL UMI URBAN SCS**	**4.92E+05**	**5.33E+05**	**5.71E+05**	**6.10E+05**	**6.51E+05**	**6.64E+05**	**6.89E+05**	**7.10E+05**	**7.31E+05**	**7.51E+05**	**7.69E+05**	**7.87E+05**	**8.04E+05**	**8.19E+05**	**8.33E+05**	**8.40E+05**	**8.41E+05**	**8.41E+05**	**8.40E+05**	**8.38E+05**	**8.35E+05**	**8.33E+05**	**8.31E+05**	**8.29E+05**	**8.26E+05**
**6**	**TOTAL GLOBAL SCS ANNUAL**	**2.14E+06**	**2.24E+06**	**2.33E+06**	**2.42E+06**	**2.51E+06**	**2.55E+06**	**2.58E+06**	**2.61E+06**	**2.64E+06**	**2.67E+06**	**2.69E+06**	**2.72E+06**	**2.74E+06**	**2.75E+06**	**2.76E+06**	**2.76E+06**	**2.74E+06**	**2.72E+06**	**2.70E+06**	**2.68E+06**	**2.66E+06**	**2.63E+06**	**2.61E+06**	**2.58E+06**	**2.56E+06**
**6**	**TOTAL GLOBAL SCS CUMULATIVE**	**2.14E+06**	**4.37E+06**	**6.70E+06**	**9.12E+06**	**1.16E+07**	**1.42E+07**	**1.68E+07**	**1.94E+07**	**2.20E+07**	**2.47E+07**	**2.74E+07**	**3.01E+07**	**3.28E+07**	**3.56E+07**	**3.84E+07**	**4.11E+07**	**4.39E+07**	**4.66E+07**	**4.93E+07**	**5.20E+07**	**5.46E+07**	**5.72E+07**	**5.99E+07**	**6.24E+07**	**6.50E+07**

Notes: Annual impact estimates in Disability-Adjusted Life Years (DALYs) for each year 2016 – 2040, by Plastics-to-Ocean system scenario (1 – 6), and by geographical archetype and plastic category. Estimated as the mean average of 300 Monte Carlo runs for each cell. Total Human Health impacts in Disability-Adjusted Life Years (DALYs) calculated as the sum of impacts for each year within each scenario, for all geographical archetypes and plastic categories. Abbreviations: System Scenarios: Business-as-Usual (BAU), Current Commitments (CCS), Collect and Dispose (CDS), Recycling (RES), Reduce and Substitute (RSS), System Change Scenario (SCS); Geographic archetypes: High-Income Rural (HIC_Rural), High-Income Urban (HIC_Urban), Upper Middle-Income Rural (UMC_Rural), Upper Middle-Income Urban (UMC_Urban), Lower Middle-Income Rural (LMC_Rural), Lower Middle-Income Urban (LMC_Urban), Low-Income Rural (LIC_Rural), Low-Income Urban (LIC_Urban); Plastic Category: Rigid Monomaterial (r), Flexible Monomaterial (f), Multilayer/Multimaterials (m).

Table S35. Human Health midpoint impact contributions to total Disability-Adjusted Life Years (DALYs) associated with system scenarios in 2016 and 2040

System Scenario (Year)	Global Warming (DALYs)	Ozone Depletion (DALYs)	Ionizing Radiation (DALYs)	Ozone Formation (DALYs)	Particulate Matter Formation (DALYs)	Carcinogenic Toxicity (DALYs)	Non-carcinogenic Toxicity (DALYs)	Water Consumption (DALYs)	Total (DALYs)
BAU (2016)	8.27E+05	3.86E+02	2.21E+02	1.49E+03	6.77E+05	4.16E+05	2.16E+05	9.55E+03	2.15E+06
BAU (2040)	1.80E+06	7.36E+02	4.41E+02	3.04E+03	1.46E+06	8.32E+05	4.08E+05	1.91E+04	4.52E+06
CCS (2040)	1.72E+06	7.13E+02	4.25E+02	2.91E+03	1.38E+06	8.10E+05	3.90E+05	1.82E+04	4.32E+06
CDS (2040)	1.60E+06	6.67E+02	4.26E+02	2.73E+03	1.12E+06	7.81E+05	4.16E+05	1.82E+04	3.94E+06
RES (2040)	1.47E+06	6.06E+02	4.28E+02	2.63E+03	1.17E+06	7.55E+05	3.41E+05	1.68E+04	3.76E+06
RSS (2040)	1.09E+06	6.49E+02	3.15E+02	2.02E+03	9.26E+05	5.24E+05	2.72E+05	1.76E+04	2.84E+06
SCS (2040)	9.68E+05	5.95E+02	3.31E+02	1.90E+03	8.00E+05	5.13E+05	2.55E+05	1.74E+04	2.56E+06

Notes: The total Disability-Adjusted Life Years (DALYs) for each midpoint impact category were calculated as the sum of impacts by category (both positive and negative) across plastic categories, geographical archetypes and life cycle stages for the years 2016 and 2040. Abbreviations: Business-as-Usual (BAU), System Change scenario (SCS).

Table S36. Sensitivity analysis of plastics mass substitution ratios by single-use paper-based substitutes and polylactide (PLA) alternatives on total Disability-Adjusted Life Years (DALYs) associated with relevant system scenarios in 2040

System Scenario (Year)	Primary modelled mass substitution ratio (all 1:1)	Low range substitution ratio (paper-based: 0.4:1, PLA: 0.8:1)	High range substitution ratio (paper-based: 9:1, PLA: 1.4:1)
RSS Absolute DALYs 2040	2.84E+06	2.72E+06	3.55E+06
RSS vs BAU (% reduction DALs)	-37%	-40%	-21%
SCS Absolute DALYs 2040	2.56E+06	2.44E+06	3.27E+06
SCS vs BAU (% reduction DALYs)	-43%	-46%	-28%

Notes: The substitution ratios are based on those identified in published literature, as detailed in section *3.2. Substitute Overview* in this Supplementary Material.

Table S37. Life cycle process contributions to total Disability-Adjusted Life Years (DALYs) associated with the Business-as-Usual system scenario in 2016 and 2040

System Scenario (Year)	Virgin Polymer Granulate Production	Mechanical Recycling	Mechanical Recycling Avoided Secondary Plastic Production	Chemical Recycling	Pyrolysis Avoided Fuel Production	Sorting and Transport	International Waste Transport	Industrial Incineration	Sanitary Landfill	Open Burning	Open Dumpsites	Terrestrial and Aquatic Pollution
BAU 2016	1.76E+06	1.39E+04	-1.65E+05	4.93E+03	-4.27E+03	2.17E+04	4.70E+03	8.02E+04	5.01E+04	3.23E+05	1.97E+04	3.89E+04
BAU 2040	3.45E+06	2.71E+04	-2.95E+05	8.98E+03	-7.71E+03	4.15E+04	8.75E+03	2.44E+05	4.19E+04	8.51E+05	3.88E+04	1.14E+05

Notes: The total Disability-Adjusted Life Years (DALYs) for each life cycle process were calculated as the sum of process impacts (both positive and negative) across plastic categories, geographical archetypes and midpoint impact categories for the years 2016 and 2040. Abbreviations: Business-as-Usual (BAU).

Table S38. Life cycle process contribution to total Disability-Adjusted Life Years (DALYs) associated with all system scenarios in 2040

System Scenario in 2040	Primary Production	Mechanical Recycling	Chemical Recycling (Pyrolysis)	Avoided Burdens Secondary Plastic Production	Avoided Burdens Fuel Production	Reuse Systems	Paper Substitutes	Compostables	Sorting and Transport	Industrial Incineration	Sanitary Landfill	Open Burning	Open Dumpsites	Terrestrial and Aquatic Pollution
BAU	3.45E+06	2.71E+04	1.27E+03	-2.95E+05	-7.71E+03	0.00E+00	0.00E+00	0.00E+00	5.02E+04	2.44E+05	4.19E+04	8.51E+05	3.88E+04	1.14E+05
CCS	3.29E+06	3.21E+04	1.31E+03	-2.83E+05	-7.74E+03	0.00E+00	0.00E+00	0.00E+00	7.24E+04	2.36E+05	4.05E+04	7.89E+05	3.95E+04	1.02E+05
CDS	3.29E+06	3.46E+04	3.32E+03	-2.84E+05	-1.20E+04	0.00E+00	0.00E+00	0.00E+00	4.94E+04	3.52E+05	1.13E+05	3.21E+05	2.25E+04	4.10E+04
RES	3.28E+06	6.19E+04	9.99E+04	-6.01E+05	-6.71E+04	0.00E+00	0.00E+00	0.00E+00	1.05E+05	1.81E+05	3.82E+04	4.91E+05	4.95E+04	5.44E+04
RSS	1.88E+06	2.60E+04	7.32E+02	-2.22E+05	-4.47E+03	9.75E+04	7.14E+04	3.52E+05	2.97E+04	1.42E+05	2.66E+04	3.61E+05	2.28E+04	4.56E+04

SCS	1.89E+06	3.98E+04	8.06E+04	-3.59E+05	-5.55E+04	9.75E+04	7.15E+04	3.55E+05	5.46E+04	1.25E+05	3.92E+04	1.39E+05	6.57E+03	2.12E+04

Notes: The total Disability-Adjusted Life Years (DALYs) for each life cycle process were calculated as the sum of process impacts (both positive and negative) across plastic categories, geographical archetypes and midpoint impact categories for the year 2040. Abbreviations: Business-as-Usual (BAU), Current Commitments (CCS), Collect and Dispose (CDS), Recycling (RES), Reduce and Substitute (RSS), System Change Scenario (SCS).

Table 39. Substance contributions to life cycle process, midpoint impacts and total Disability-Adjusted Life Years (DALYs) associated with the System Change scenario in 2040

**Contributions to global DALYs in System Change scenario 2040**						
**Contributing midpoint impact**	**Contributing substance emission**	**Contributing life cycle stage**	**Substance emission contribution to total DALYs in 2040 (%)**	**Midpoint contribution to total DALYs (%)**	**Process contribution to midpoint (%)**	**Substance contribution to process by midpoint (%)**
Global Warming	Carbon dioxide, fossil (Air)	Virgin Production	1.95E-01	3.79E-01	6.31E-01	8.16E-01
Global Warming	Methane, fossil (Air)	Virgin Production	4.24E-02	3.79E-01	6.31E-01	1.77E-01
**Global Warming**	**Other**	**Virgin Production**	**1.63E-03**	3.79E-01	6.31E-01	**6.83E-03**
Global Warming	Carbon dioxide, fossil (Air)	Compostable Substitution	3.20E-02	3.79E-01	1.04E-01	8.15E-01
Global Warming	Methane, fossil (Air)	Compostable Substitution	3.77E-03	3.79E-01	1.04E-01	9.63E-02
Global Warming	Dinitrogen monoxide (Air)	Compostable Substitution	3.36E-03	3.79E-01	1.04E-01	8.57E-02
**Global Warming**	**Other**	**Compostable Substitution**	**1.17E-04**	3.79E-01	1.04E-01	**2.98E-03**
Global Warming	Carbon dioxide, fossil (Air)	Industrial Incineration	3.49E-02	3.79E-01	9.24E-02	9.97E-01
**Global Warming**	**Other**	**Industrial Incineration**	**1.11E-04**	3.79E-01	9.24E-02	**3.16E-03**
Global Warming	Carbon dioxide, fossil (Air)	Open Burning	2.01E-02	3.79E-01	5.70E-02	9.30E-01
Global Warming	Methane, fossil (Air)	Open Burning	1.28E-03	3.79E-01	5.70E-02	5.93E-02
**Global Warming**	**Other**	**Open Burning**	**2.31E-04**	3.79E-01	5.70E-02	**1.07E-02**
**Global Warming**	Carbon dioxide, fossil (Air)	Chemical Recycling: Pyrolysis	1.40E-02	3.79E-01	3.99E-02	9.25E-01
**Global Warming**	Methane, fossil (Air)	Chemical Recycling: Pyrolysis	1.02E-03	3.79E-01	3.99E-02	6.78E-02

Contributions to global DALYs in System Change scenario 2040						
**Contributing midpoint impact**	**Contributing substance emission**	**Contributing life cycle stage**	**Substance emission contribution to total DALYs in 2040 (%)**	**Midpoint contribution to total DALYs (%)**	**Process contribution to midpoint (%)**	**Substance contribution to process by midpoint (%)**
**Global Warming**	**Other**	Chemical Recycling: Pyrolysis	**1.13E-04**	3.79E-01	3.99E-02	**7.44E-03**
**Global Warming**	**Other**	**Other**	**2.88E-02**			
Particulate Matter Formation	Sulfur dioxide (Air)	Virgin Production	8.69E-02	3.13E-01	6.12E-01	4.54E-01
Particulate Matter Formation	Particulates, < 2.5 um (Air)	Virgin Production	7.19E-02	3.13E-01	6.12E-01	3.75E-01
Particulate Matter Formation	Nitrogen oxides (Air)	Virgin Production	3.18E-02	3.13E-01	6.12E-01	1.66E-01
**Particulate Matter Formation**	**Other**	**Virgin Production**	**8.96E-04**	3.13E-01	6.12E-01	**4.68E-03**
Particulate Matter Formation	Particulates, < 2.5 um (Air)	Compostable Substitution	1.82E-02	3.13E-01	1.42E-01	4.09E-01
Particulate Matter Formation	Sulfur dioxide (Air)	Compostable Substitution	1.41E-02	3.13E-01	1.42E-01	3.17E-01
Particulate Matter Formation	Ammonia (Air)	Compostable Substitution	5.19E-03	3.13E-01	1.42E-01	1.17E-01
Particulate Matter Formation	Nitrogen oxides (Air)	Compostable Substitution	6.68E-03	3.13E-01	1.42E-01	1.50E-01
**Particulate Matter Formation**	**Other**	**Compostable Substitution**	**3.04E-04**	3.13E-01	1.42E-01	**6.85E-03**
Particulate Matter Formation	Particulates, < 2.5 um (Air)	Chemical Recycling: Pyrolysis	**1.13E-02**	3.13E-01	6.07E-02	5.96E-01
Particulate Matter Formation	Sulfur dioxide (Air)	Chemical Recycling: Pyrolysis	**5.65E-03**	3.13E-01	6.07E-02	2.98E-01
Particulate Matter Formation	Nitrogen oxides (Air)	Chemical Recycling: Pyrolysis	**1.97E-03**	3.13E-01	6.07E-02	1.04E-01
**Particulate Matter Formation**	**Other**	Chemical Recycling: Pyrolysis	**5.47E-05**	3.13E-01	6.07E-02	**2.88E-03**
Particulate Matter Formation	Particulates, < 2.5 um (Air)	Open Burning	**2.14E-02**	3.13E-01	7.39E-02	9.27E-01

**Contributions to global DALYs in System Change scenario 2040**						
**Contributing midpoint impact**	**Contributing substance emission**	**Contributing life cycle stage**	**Substance emission contribution to total DALYs in 2040 (%)**	**Midpoint contribution to total DALYs (%)**	**Process contribution to midpoint (%)**	**Substance contribution to process by midpoint (%)**
Particulate Matter Formation	Nitrogen Oxides (Air)	Open Burning	**1.39E-03**	3.13E-01	7.39E-02	6.03E-02
Particulate Matter Formation	Sulphur dioxide (Air)	Open Burning	**2.84E-04**	3.13E-01	7.39E-02	1.23E-02
**Particulate Matter Formation**	**Other**	**Open Burning**	**-1.24E-11**	3.13E-01	7.39E-02	**-5.38E-10**
**Particulate Matter Formation**	**Other**	**Other**	**3.49E-02**			
Carcinogenic Toxicity	Chromium VI (Groundwater, long term)	Virgin Production	1.48E-01	2.01E-01	7.58E-01	9.71E-01
**Carcinogenic Toxicity**	**Other**	**Virgin Production**	**4.44E-03**	2.01E-01	7.58E-01	**2.92E-02**
Carcinogenic Toxicity	Chromium VI (Groundwater, long term)	Compostable Substitution	1.91E-02	2.01E-01	9.93E-02	9.57E-01
**Carcinogenic Toxicity**	**Other**	**Compostable Substitution**	**8.48E-04**	2.01E-01	9.93E-02	**4.25E-02**
**Carcinogenic Toxicity**	Chromium VI (Groundwater, long term)	Chemical Recycling: Pyrolysis	**8.47E-03**	2.01E-01	4.38E-02	9.65E-01
**Carcinogenic Toxicity**	**Other**	Chemical Recycling: Pyrolysis	**3.11E-04**	2.01E-01	4.38E-02	**3.55E-02**
Carcinogenic Toxicity	Dioxin, 2,3,7,8 Tetrachlorodibenzo-p- (Air)	Open Burning	**1.47E-03**	2.01E-01	9.06E-03	8.09E-01
Carcinogenic Toxicity	PAH, polycyclic aromatic hydrocarbons (Air)	Open Burning	**2.83E-04**	2.01E-01	9.06E-03	1.56E-01
**Carcinogenic Toxicity**	**Other**	Open Burning	**6.45E-05**	2.01E-01	9.06E-03	**3.55E-02**
**Carcinogenic Toxicity**	**Other**	**Other**	**1.81E-02**			
Non-Carcinogenic Toxicity	Zinc (II) (Groundwater, long term)	Virgin Production	2.45E-02	9.98E-02	5.39E-01	4.56E-01

Contributions to global DALYs in System Change scenario 2040						
**Contributing midpoint impact**	**Contributing substance emission**	**Contributing life cycle stage**	**Substance emission contribution to total DALYs in 2040 (%)**	**Midpoint contribution to total DALYs (%)**	**Process contribution to midpoint (%)**	**Substance contribution to process by midpoint (%)**
Non-Carcinogenic Toxicity	Arsenic, ion (Groundwater, long term)	Virgin Production	1.70E-02	9.98E-02	5.39E-01	3.17E-01
Non-Carcinogenic Toxicity	Arsenic, ion (Air)	Virgin Production	3.36E-03	9.98E-02	5.39E-01	6.24E-02
**Non-Carcinogenic Toxicity**	**Other**	**Virgin Production**	**8.84E-03**	9.98E-02	5.39E-01	**1.64E-01**
Non-Carcinogenic Toxicity	Zinc (II) (Groundwater, long term)	Compostable Substitution	6.98E-03	9.98E-02	1.48E-01	4.74E-01
Non-Carcinogenic Toxicity	Arsenic, ion (Groundwater, long term)	Compostable Substitution	2.87E-03	9.98E-02	1.48E-01	1.95E-01
Non-Carcinogenic Toxicity	Vanadium (V) (Groundwater, long term)	Compostable Substitution	2.06E-03	9.98E-02	1.48E-01	1.40E-01
**Non-Carcinogenic Toxicity**	**Other**	**Compostable Substitution**	**2.82E-03**	9.98E-02	1.48E-01	**1.92E-01**
Non-Carcinogenic Toxicity	Zinc (Groundwater, long term)	Sanitary Landfill	1.01E-02	9.98E-02	1.16E-01	8.76E-01
Non-Carcinogenic Toxicity	Vanadium (V) (Groundwater, long term)	Sanitary Landfill	9.53E-04	9.98E-02	1.16E-01	8.26E-02
**Non-Carcinogenic Toxicity**	**Other**	**Sanitary Landfill**	**4.78E-04**	9.98E-02	1.16E-01	**4.14E-02**
Non-Carcinogenic Toxicity	Zinc (Groundwater, long term)	Industrial Incineration	2.53E-03	9.98E-02	5.08E-02	5.00E-01
Non-Carcinogenic Toxicity	Vanadium (V) (Groundwater, long term)	Industrial Incineration	1.72E-03	9.98E-02	5.08E-02	3.40E-01
Non-Carcinogenic Toxicity	Arsenic (River)	Industrial Incineration	3.28E-04	9.98E-02	5.08E-02	6.47E-02
**Non-Carcinogenic Toxicity**	**Other**	**Industrial Incineration**	**4.82E-04**	9.98E-02	5.08E-02	**9.52E-02**
Non-Carcinogenic Toxicity	Zinc (II) (Groundwater, long term)	Chemical Recycling: Pyrolysis	1.48E-03	9.98E-02	3.23E-02	4.61E-01
Non-Carcinogenic Toxicity	Arsenic, ion (Groundwater, long term)	Chemical Recycling: Pyrolysis	1.19E-03	9.98E-02	3.23E-02	3.68E-01

**Contributions to global DALYs in System Change scenario 2040**						
**Contributing midpoint impact**	**Contributing substance emission**	**Contributing life cycle stage**	**Substance emission contribution to total DALYs in 2040 (%)**	**Midpoint contribution to total DALYs (%)**	**Process contribution to midpoint (%)**	**Substance contribution to process by midpoint (%)**
Non-Carcinogenic Toxicity	Arsenic, ion (Air)	Chemical Recycling: Pyrolysis	6.38E-05	9.98E-02	3.23E-02	1.98E-02
Non-Carcinogenic Toxicity	**Other**	Chemical Recycling: Pyrolysis	**4.88E-04**	9.98E-02	3.23E-02	**1.51E-01**
Non-Carcinogenic Toxicity	**Vanadium (V) (Air)**	Open Burning	2.70E-04	9.98E-02	9.84E-03	2.75E-01
Non-Carcinogenic Toxicity	**Vanadium (V) (Soil)**	Open Burning	2.04E-04	9.98E-02	9.84E-03	2.08E-01
Non-Carcinogenic Toxicity	**Barium (II) (Soil)**	Open Burning	1.72E-04	9.98E-02	9.84E-03	1.76E-01
Non-Carcinogenic Toxicity	**Zinc (II) (Soil)**	Open Burning	1.18E-04	9.98E-02	9.84E-03	1.20E-01
Non-Carcinogenic Toxicity	**Antimony, ion (Soil)**	Open Burning	3.48E-05	9.98E-02	9.84E-03	3.54E-02
Non-Carcinogenic Toxicity	**Cadmium (II) (Soil)**	Open Burning	5.72E-05	9.98E-02	9.84E-03	5.83E-02
Non-Carcinogenic Toxicity	**Zinc (II) (Soil)**	Open Burning	5.09E-05	9.98E-02	9.84E-03	5.18E-02
Non-Carcinogenic Toxicity	**Other**	Open Burning	**7.42E-05**	9.98E-02	9.84E-03	**7.56E-02**
**Non-Carcinogenic Toxicity**	**Other**	**Other**	**1.05E-02**			
**Other**	**Other**	**Other**	**7.89E-03**			

***Notes**:* The table provides a detailed breakdown of process, activity, and substance contributions to the four midpoint impacts found to contribute most to Human Health Disability-Adjusted Life Years (DALYs) in the Plastics-to-Ocean (P_2O) *System Change* scenario in 2040. The *System Change* scenario combined reductions in plastic use, increased collection and disposal, material substitutions and reuse, and increased recycling, implemented globally.[1] Other categories of midpoint impacts were not included in this detailed breakdown because their contribution to total DALYs for the *System Change* scenario in 2040 were less than 5% of total impacts: Ozone Depletion (0.02%), Ionising Radiation (0.01%), Ozone Formation (0.07%), Water Consumption (0.67%).

Table 40. Sub-process activity contributions to total Disability-Adjusted Life Years (DALYs) associated with global system life cycle stages by midpoint impacts

PRIMARY POLYMER PRODUCTION

GLOBAL WARMING

	RM	FM	MM - HIC	MM - LMIC
Electricity production	25	29	24	28
Hydrocarbons production, steam cracking	14	21	17	19
Natural gas venting	11	12	11	12
Heat production	11	5	6	6
Methanol production, chemical feedstock	5	5	6	6
Sweet gas burning in turbine and waste flare	5	6	5	6
Transport (road, rail, inland waterways and ocean)	4	4	4	4
Reformate production, chemical feedstock	3	0	0	0
Diesel production and diesel burning in machinery	2	2	2	2
Xylenes production, chemical feedstock	2	0	0	0
Naptha production, chemical feedstock	1	2	2	2
Direct emissions from polymerisation	1	1	1	1
Ethylene oxide production, chemical feedstock	1	0	0	0
Synethetic fuel production	1	1	1	1
Polystyrene granulate production, black box	4	0	12	3
Other	10	11	10	11

PARTICULATE MATTER FORMATION

	RM	FM	MM - HIC	MM - LMIC
Electricity production	50	57	51	56
Transport (road, rail, inland waterways and ocean)	9	9	8	9
Heat production	9	5	6	6
Diesel production and diesel burning in machinery	3	4	3	4
Reformate production, chemical feedstock	4	0	0	0
Naphtha production, chemical feedstock	2	3	3	3
Synethetic fuel production	1	1	1	1
Copper smelting for chemical production	5	5	5	5
Petroleum and gas production	4	4	4	4
Mining operations, chemical feedstocks and infrastructure	2	2	2	2
Blasting for mining operations	1	1	1	1
Polystyrene granulate production, black box	3	0	9	2
Other	7	7	7	7

CARCINOGENIC TOXICITY

	RM	FM	MM - HIC	MM - LMIC
Residual material landfill	89	93	80	96
Tailings impoundment from mining operations	1	1	0	0
Surface landfill of mining spoil	9	6	17	3
Opencast refill treatment of lignite ash	1	0	1	0
Other	1	0	1	1

NON-CARCINOGENIC TOXICITY

	RM	FM	MM - HIC	MM - LMIC
Tailings impoundment from mine operations	35	35	34	35
Residual material landfill	27	25	25	25
Surface landfill of mining spoil	17	20	19	20
Copper smelting for chemical production	10	10	10	10
Treatment of bottom ash, from waste treatment of used transport vehicles	2	2	2	2
Opencast refill treatment of lignite ash	1	1	1	1
Direct emissions from polymer specific waste	0	0	0	0
Other	9	8	9	8

CHEMICAL RECYCLING

GLOBAL WARMING

	HIC	UMC	LMC	LIC
Electricity production	46	55	62	40
Direct emissions from pyrolysis	38	27	24	42
Heat production	4	3	2	4
Transport (road, rail, inland waterways and ocean)	3	2	2	3
Natural gas venting	3	2	2	3
Sweet gas burning in turbine and waste flare	1	1	1	2
Diesel production and diesel burning in machinery	1	1	1	1
Other	5	10	6	5

PARTICULATE MATTER FORMATION

	HIC	UMC	LMC	LIC
Electricity production	80	89	93	80
Transport (road, rail, inland waterways and ocean)	5	2	1	4
Diesel production and burning in machinery	3	2	1	3
Heat production	3	2	1	3
Mining operations, chemical feedstocks and infrastructure	1	1	2	1
Copper smelting for chemical production	1	1	0	1
Petroleum and natural gas production	1	0	0	2
Other	6	3	2	6

CARCINOGENIC TOXICITY

	HIC	UMC	LMC	LIC
Electricity production	80	89	93	80
Transport (road, rail, inland waterways and ocean)	5	2	1	4
Diesel production and burning in machinery	3	2	1	3
Heat production	3	2	1	3
Mining operations, chemical feedstocks and infrastructure	1	1	2	1
Copper smelting for chemical production	1	1	0	1
Petroleum and natural gas production	1	0	0	2
Other	6	3	2	6

NON-CARCINOGENIC TOXICITY

	HIC	UMC	LMC	LIC
Surface landfill of mining spoil	54	31	58	29
Residual material landfill	13	47	20	34
Tailings impoundment from mine operations	13	11	5	17
Opencast refill treatment of lignite ash from electricity and heat production	4	1	5	1
Copper smelting for chemical production	3	2	1	5
Treatment of bottom ash, from waste treatment of used transport vehicles	2	2	1	4
Direct emissions from polymer specific waste	0	0	0	0
Other	65	38	67	40

COMPOSTABLE PRODUCTION AND WASTE MANAGEMENT

GLOBAL WARMING

	HIC	UMC	LMC	LIC
Electricity production	39	39	39	39
Heat production	24	24	24	24
Direct field emissions from maize grain production	7	7	7	7
Hard coal and lignite mine operation, energy	4	4	4	4
Transport (tractor, road, rail, inland waterways and ocean)	4	4	4	4
Natural gas venting, energy	4	4	4	4
Land tenure, land conversion for annual crops	3	3	3	3
Sweet gas burning in turbine and waste flare, energy	2	2	2	2
Ammonia production, fertilizer	2	2	2	2
Diesel production and diesel burning in machinery	1	1	1	1
Direct emissions from field tillage	1	1	1	1
Other	7	7	7	7

PARTICULATE MATTER FORMATION

	HIC	UMC	LMC	LIC
Electricity production	55	55	55	55
Direct field emissions from maize grain production	11	11	11	11
Heat production	6	6	6	6
Land tenure, land conversion for annual crops	3	3	3	3
Transport (tractor, road, rail, inland waterways and ocean)	6	6	6	6
Copper smelting for chemical production	4	4	4	4
Direct emissions from field tillage	2	2	2	2
Direct emissions from combine harvesting	1	1	1	1
Direct emissions from PLA incineration	1	1	1	1
Other	10	10	10	10

CARCINOGENIC TOXICITY

	HIC	UMC	LMC	LIC
Electricity production	55	55	55	55
Direct field emissions from maize grain production	11	11	11	11
Heat production	6	6	6	6
Land tenure, land conversion for annual crops	3	3	3	3
Transport (tractor, road, rail, inland waterways and ocean)	6	6	6	6
Copper smelting for chemical production	4	4	4	4
Direct emissions from field tillage	2	2	2	2
Direct emissions from combine harvesting	1	1	1	1
Direct emissions from PLA incineration	1	1	1	1
Other	10	10	10	10

NON-CARCINOGENIC TOXICITY

	HIC	UMC	LMC	LIC
Treatment of bottom ash from PLA incineration	22	22	22	22
Residual material landfill	29	29	29	29
Tailings impoundment from mine operations	20	20	20	20
Surface landfill of mining spoil	14	14	14	14
Copper smelting for chemical production	6	6	6	6
Opencast refill treatment of lignite ash from electricity and heat production	1	1	1	1
Direct emissions from polymer specific waste	0	0	0	0
Other	8	8	8	8

INCINERATION

GLOBAL WARMING

	RM	FM	MM - HIC	MM - LMIC
Direct emissions	99	99	98	99
Other	1	1	2	1

PARTICULATE MATTER FORMATION

	RM	FM	MM - HIC	MM - LMIC
Direct emissions from incineration	71	68	56	63
Electricity production	10	11	18	14
Heat production	4	4	4	4
Transport (road, rail, inland waterways and ocean)	3	3	4	4
Clinker production, for cement for treatment of residues	2	2	3	3
Diesel production and burning in machinery	2	2	2	2
Mining operations, chemical feedstocks and infrastructure	1	1	1	1
Copper smelting for chemical production	1	1	1	1
Other	7	8	10	9

CARCINOGENIC TOXICITY

	RM	FM	MM - HIC	MM - LMIC
Residual material landfill	97	97	97	97
Tailings impoundment from mine operations	0	0	0	0
Surface landfill of mining spoil	0	0	1	1
Opencast refill treatment of lignite ash from electricity and heat production	0	0	0	0
Other	2	2	2	2

NON-CARCINOGENIC TOXICITY

	RM	FM	MM - HIC	MM - LMIC
Treatment of bottom ash from incineration	52	55	60	56
Residual material landfill	45	42	37	41
Tailings impoundment from mine operations	0	0	0	0
Surface landfill of mining spoil	0	0	0	0
Copper smelting for chemical production	0	0	0	0
Opencast refill treatment of lignite ash from electricity and heat production	0	0	0	0
Direct emissions from polymer specific waste	2	2	2	2
Other	2	2	2	2

Notes: Detailed sub-process activity analysis conducted for the life cycle stages responsible for 5% or more of Disability-Adjusted Life Years (DALYs) for each midpoint impact category contributing more than 5% to total Human Health DALYs associated with the System Change scenario in 2040. Sub-activity contributions themselves are not time-dependent as the same inventories were applied across time in the models. All data are presented as percentage contributions (%) to the total DALYs of each midpoint category (Global Warming, Particulate Matter Formation, Carcinogenic Toxicity, Non-Carcinogenic Toxicity) for each life cycle stage or system (Primary Polymer Production, Chemical Recycling, Compostable Production and Waste Management, Incineration). The results of the analysis are sub-divided by the P_2O plastic categories and/or geographical archetypes where relevant. Abbreviations: Rigid Monomaterials (RM), Flexible Monomaterials (FM), Multilayers/Multimaterials in High-Income Countries Archetype (MM-HIC), Multilayers/Multimaterials in Low- and Middle-Income Countries Archetypes (MM-LMC), all plastic categories (ALL), High-Income Countries Archetype (HIC), Upper Middle-Income Countries Archetype (UMC), Lower Middle-Income Countries Archetype (UMC), Low-Income Countries Archetype (LIC).

6. REFERENCES

1 Lau WWY, Shiran Y, Bailey RM, *et al.* Evaluating scenarios toward zero plastic pollution. *Science (1979)* 2020; **369**: 1455–61.

2 Pew Charitable Trusts, SYSTEMIQ. Breaking the plastic wave: a comprehensive assessment of pathways towards stopping ocean plastic pollution. Pew Charitable Trusts, SYSTEMIQ, 2020 https://www.systemiq.earth/breakingtheplasticwave/ (accessed Oct 20, 2025).

3 ecoinvent. ecoinvent Database [database]. ecoinvent. 2022. https://ecoinvent.org/the-ecoinvent-database/ (accessed Oct 30, 2025).

4 Mutel C. Geographies - ecoinvent. ecoinvent. https://ecoinvent.org/the-ecoinvent-database/geographies/ (accessed Oct 10, 2023).

5 Huijbregts MAJ, Steinmann ZJN, Eishout PMF, *et al.* ReCiPe 2016 v1.1. A harmonized life cycle impact assessment method at midpoint and endpoint level. Report I: Characterization. National Institute for Public Health and the Environment. Ministry of Health, Welfare and Sport, The Netherlands, 2017 https://www.rivm.nl/bibliotheek/rapporten/2016-0104.html (accessed Oct 19, 2025).

6 PRé Sustainability. SimaPro LCA software for informed change-makers. Simapro. 2022. https://www.simapro.co.uk/simapro (accessed Dec 5, 2021).

7 Kaza S, Yao LC, Bhada-Tata P, Van Woerden F. What a Waste 2.0: A Global Snapshot of Solid Waste Management to 2050. Washington DC: World Bank, 2018 https://openknowledge.worldbank.org/handle/10986/30317 (accessed Jan 24, 2022).

8 Organisation for Economic Co-operation and Development (OECD). Global Plastics Outlook: Economic Drivers, Environmental Impacts and Policy Options. OECD, 2022 https://www.oecd-ilibrary.org/environment/global-plastics-outlook_de747aef-en (accessed Sept 14, 2023).

9 Plastics Europe. Plastics—The Facts 2021: An analysis of European Plastics Production, Demand and Waste Data. Plastics Europe. 2021 https://plasticseurope.org/knowledge-hub/plastics-the-facts-2021/ (accessed Oct 20, 2025).

10 Cimpan C, Bjelle EL, Strømman AH. Plastic packaging flows in Europe: A hybrid input-output approach. *J Ind Ecol* 2021; **25**: 1572–87.

11 Waste & Resources Action Programme (WRAP). PlasticFlow 2025: Plastic Packaging Flow Data Report. WRAP, 2018 https://wrap.org.uk/sites/default/files/2020-11/WRAP-PlasticFlow 2025 Plastic Packaging Flow Data Report_0.pdf (accessed Oct 20, 2025).

12 Deloitte Sustainability. Blueprint for Plastics Packaging Waste: Quality Sorting & Recycling - Final report. Deloitte, 2017 https://www2.deloitte.com/content/dam/Deloitte/my/Documents/risk/my-risk-blueprint-plastics-packaging-waste-2017.pdf (accessed Oct 20, 2025).

13 Irby C. What is the Difference Between HDPE and PET? Plascene. 2021. https://www.plascene.com/whats-the-difference-between-hdpe-and-pet (accessed Aug 30, 2023).

14 Gall M, Schweighuber A, Buchberger W, Lang RW. Plastic bottle cap recycling - characterization of recyclate composition and opportunities for design for circularity. *Sustainability* 2020; **12**: 1–21.

15 International Life Sciences Institute (ILSI). Packaging Materials 4. Polyethylene for Food Packaging Applications. Brussels, Belgium: ILSI Europe, 2003

https://ilsi.eu/wp-content/uploads/sites/3/2016/06/R2003Pac_Mat.pdf (accessed Aug 30, 2023).
16 Euromonitor International. Rigid Plastic Packaging in Western Europe. Euromonitor International, 2021 https://www.euromonitor.com/rigid-plastic-packaging-in-western-europe/report (accessed Aug 30, 2023).
17 Copello L, Haut G, Maillot J, Mongodin F. Moving on from single-use plastics: How is Europe doing? Assessment of European countries' transposition of the Single Use Plastics Directive. Rethink Plastic Alliance, Break Free From Plastic, 2021 https://rethinkplasticalliance.eu/wp-content/uploads/2021/06/SUP-Assessment-Design-final.pdf (accessed Aug 30, 2023).
18 Materson V. Which countries have bans on single-use plastics? World Economic Forum: Plastics and the Environment. 2020; published online Oct. https://www.weforum.org/agenda/2020/10/canada-bans-single-use-plastics/ (accessed Aug 30, 2023).
19 European Commission. Energy C change, E. Single-use plastics. European Commission. 2019. https://environment.ec.europa.eu/topics/plastics/single-use-plastics_en (accessed Aug 30, 2023).
20 Gore-Langton L. EU declares blanket ban on harmful packaging chemicals but braces for petrochemical fightback. *Packaging Insights* 2022; published online April. https://www.packaginginsights.com/news/eu-declares-blanket-ban-on-harmful-packaging-chemicals-but-braces-for-petrochemical-fightback.html (accessed Aug 30, 2023).
21 United Nations Environment Programme (UNEP). Single-use plastic bags and their alternatives: Recommendations from Life Cycle Assessments. UNEP, 2020 https://www.lifecycleinitiative.org/wp-content/uploads/2020/04/Single-use-plastic-bags-and-alternatives-Recommendations-from-LCA-final.pdf (accessed Oct 20, 2025).
22 Industrial Netting. Materials: Plastic Netting: Polypropylene, Nylon, Polyethylene, Polyester & More. Industrial Netting. 2023. https://www.industrialnetting.com/materials.html (accessed Aug 30, 2023).
23 Mieth A, Hoekstra E, Simoneau C. Guidance for the identification of polymers in multilayer films used in food contact materials. Publications Office of the European Union, 2016 https://op.europa.eu/en/publication-detail/-/publication/12f4d00c-0203-11e6-b713-01aa75ed71a1/language-en (accessed Aug 30, 2023).
24 Unilever. Our solution for recycling plastic sachets takes another step forward. Unilever. 2018; published online Nov. https://www.unilever.com/news/news-search/2018/our-solution-for-recycling-plastic-sachets-takes-another-step-forward/ (accessed Aug 30, 2023).
25 Waste & Resources Action Programme (WRAP). Collection of food and drink cartons at the kerbside: Guidance for local authorities and waste contractors. WRAP, 2017 https://wrap.org.uk/sites/default/files/2020-09/WRAP_2923_Collection-food-drink-cartons-kerbside-guidance.pdf (accessed Aug 30, 2023).
26 Woeller KE, Hochwalt AE. Safety assessment of sanitary pads with a polymeric foam absorbent core. *Regulatory Toxicology and Pharmacology* 2015; **73**: 419–24.
27 Resource Futures. Mapping Economic, Behavioural and Social Factors within the Plastic Value Chain that lead to Marine Litter in Scotland. Menstrual products report. The Scottish Government, 2019 https://www.gov.scot/binaries/content/documents/govscot/publications/research-and-analysis/2020/02/mapping-economic-behavioural-social-factors-within-marine-plastic-value-chain-scotland/documents/summary-report/summary-report/govscot%3Adocument/summary-re (accessed Aug 30, 2023).

28 United Nations Environment Programme (UNEP). Single-use menstrual products and their alternatives: Recommendations from Life Cycle Assessments. UNEP, 2021 https://www.lifecycleinitiative.org/wp-content/uploads/2021/07/UNEP-LCI-Single-use-vs-reusable-Menstrual-Products-Meta-study.pdf (accessed Oct 20, 2025).
29 Environment Agency. An updated lifecycle assessment study for disposable and reusable nappies. Environment Agency, 2008 www.environment-agency.gov.uk (accessed Aug 30, 2023).
30 Kawecki D, Nowack B. Polymer-Specific Modeling of the Environmental Emissions of Seven Commodity Plastics As Macro- and Microplastics. *Environ Sci Technol* 2019; **53**: 9664–76.
31 Plastics Europe. Polyolefins. Plastics Europe. 2023. https://plasticseurope.org/plastics-explained/a-large-family/polyolefins-2/ (accessed Sept 1, 2023).
32 D W Plastics Ltd. What type of plastic to use? D W Plastics Ltd. 2023. https://www.dwplastics.co.uk/what-type-of-plastic-to-use/#pet (accessed Sept 1, 2023).
33 Plastics Europe. Eco-profiles set (Database). Plastics Europe. 2023. https://plasticseurope.org/sustainability/circularity/life-cycle-thinking/eco-profiles-set/ (accessed June 12, 2023).
34 The European Council of Vinyl Manufacturers (ECVM). The polymerisation process. ECVM. 2023. https://pvc.org/about-pvc/polymerisation-process/ (accessed Sept 15, 2023).
35 International Life Sciences Institute (ILSI). Packaging Materials 2. Polystyrene for Food Packaging Applications. ILSI Europe, 2017 www.ilsi.eu. (accessed Sept 15, 2023).
36 United Nations Statistics Division. UN Comtrade Database: Trade Data. United Nations Statistics Division, 2023 https://comtradeplus.un.org/TradeFlow?Frequency=A&Flows=M&CommodityCodes=3915&Partners=0&Reporters=all&period=2022&AggregateBy=none&BreakdownMode=plus (accessed Sept 9, 2023).
37 European Union. Eurostat: Transport database. European Union, 2023 https://ec.europa.eu/eurostat/web/transport/data/database (accessed Sept 6, 2023).
38 Kägi T, Zschokke M, Stettler C, Carbotech AG. Life Cycle Inventories for Swiss Recycling Processes. Part Carbotech: Recycling of Cardboard, Glass, PE, PET, Tinplate. Carbotech AG, 2017 https://carbotech.ch/cms/wp-content/uploads/Technical_Report_V1.0-2.pdf (accessed Sept 5, 2023).
39 Franklin Associates. Life Cycle Inventory of 100% Postconsumer HDPE and PET Recycled Resin from Postconsumer Containers and Packaging. Kansas, USA: Franklin Associates, 2011 https://www.americanchemistry.com/better-policy-regulation/plastics/resources/life-cycle-inventory-of-100-postconsumer-hdpe-and-pet-recycled-resin-from-postconsumer-containers-and-packaging (accessed Oct 27, 2025).
40 Tonini D, Garcia-Gutierrez P, Nessi S. Environmental effects of plastic waste recycling. Focus on Climate Change effects. European Union, 2021 https://publications.jrc.ec.europa.eu/repository/handle/JRC122455 (accessed Oct 27, 2025).
41 World Population Review. Plastic Pollution by Country 2023. World Population Review. 2023. https://worldpopulationreview.com/country-rankings/plastic-pollution-by-country (accessed June 12, 2023).
42 Jambeck JR, Geyer R, Wilcox C, *et al.* Plastic waste inputs from land into the ocean. *Science (1979)* 2015; **347**: 768–71.

43 Rigamonti L, Falbo A, Grosso M. Improvement actions in waste management systems at the provincial scale based on a life cycle assessment evaluation. *Waste Management* 2013; **33**: 2568–78.
44 Jambeck JR, Geyer R, Wilcox C, *et al.* Plastic waste inputs from land into the ocean. *Science (1979)* 2015; **347**: 768–71.
45 Environmental Investigation Agency (EIA). The Truth Behind Trash: The scale and impact of the international trade in plastic waste. 2021 https://eia-international.org/wp-content/uploads/EIA-The-Truth-Behind-Trash-FINAL.pdf (accessed Oct 27, 2025).
46 Ren Y, Shi L, Bardow A, Geyer R, Suh S. Life-cycle environmental implications of China's ban on post-consumer plastics import. *Resour Conserv Recycl* 2020; **156**: 104699.
47 sea-distances.org. Sea distances/port distances - online tool for calculation distances between sea ports. sea-distances.org. 2017. https://sea-distances.org/ (accessed June 12, 2023).
48 Cook E, Velis C. Global Review on Safer End of Engineered Life. Engineering X, founded by the Royal Academy of Engineering and the Lloyd's Register Foundation, 2020 https://engineeringx.raeng.org.uk/media/xf3bekhf/groseel_lr.pdf (accessed Oct 27, 2025).
49 World Ocean Day Youth Advisory Council. Coastal and Inland Cleanup Guide. World Ocean Day, 2023 https://worldoceanday.org/resources/coastal-and-inland-cleanup-guide/?gclid=EAIaIQobChMIxrzI5YD2gAMVxxOiAx3PFgWAEAAYAyAAEgJriPD_BwE.
50 The Ocean Cleanup. The Ocean Cleanup. The Ocean Cleanup. 2023. https://theoceancleanup.com/ (accessed Sept 9, 2023).
51 The Ocean Cleanup. The Ocean Cleanup Environmental Impact Assessment. The Ocean Cleanup, 2018 https://www.google.com/url?sa=t&rct=j&q=&esrc=s&source=web&cd=1&cad=rja&uact=8&ved=2ahUKEwj_1_XK8PnoAhX4QUEAHVGgBasQFjAAegQIAxAB&url=https%3A%2F%2Fassets.theoceancleanup.com%2Fapp%2Fuploads%2F2019%2F04%2FTOC_EIA_2018.pdf&usg=AOvVaw1-JkDEMzZVa_xRNceKb70P (accessed Oct 27, 2025).
52 The World Bank. World Bank Country and Lending Groups – World Bank Data Help Desk. The World Bank. 2022. https://datahelpdesk.worldbank.org/knowledgebase/articles/906519-world-bank-country-and-lending-groups (accessed Jan 30, 2022).
53 Klotz M, Haupt M. A high-resolution dataset on the plastic material flows in Switzerland. *Data Brief* 2022; **41**: 108001.
54 Somoza-Tornos A, Gonzalez-Garay A, Pozo C, Graells M, Espuña A, Guillén-Gosálbez G. Realizing the Potential High Benefits of Circular Economy in the Chemical Industry: Ethylene Monomer Recovery via Polyethylene Pyrolysis. *ACS Sustain Chem Eng* 2020; **8**: 3561–72.
55 Plastics Europe. Recycling technologies. Plastics Europe, 2023 https://plasticseurope.org/sustainability/circularity/recycling/recycling-technologies/ (accessed Sept 10, 2023).
56 Organisation for Economic Co-operation and Development (OECD) Environment Statistics (database). Global Plastics Outlook: Plastic waste by end-of-life fate and region - projections. OECD https://www.oecd-ilibrary.org/environment/data/global-plastic-outlook_c0821f81-en (accessed Oct 19, 2025).
57 Organisation for Economic Co-operation and Development (OECD). The current plastics lifecycle is far from circular. OECD. 2019.

https://www.oecd.org/environment/plastics/plastics-lifecycle-is-far-from-circular.htm (accessed Sept 11, 2023).
58 Plastics Recyclers Europe. Plastics recycling industry in Europe - Mapping of installed plastics recycling capacities. Plastics Recyclers Europe, 2022 https://www.plasticsrecyclers.eu/wp-content/uploads/2023/03/Statistics_2023_FINAL_V2.pdf (accessed Sept 11, 2023).
59 Greenpeace. Circular claims fall flat again: 2022 Update. Greenpeace, 2022.
60 Doka G. Life Cycle Inventories of Waste Treatment Services. Swiss Centre for Life Cycle Inventories, 2003 www.ecoinvent.ch (accessed Sept 2, 2023).
61 Doka G. Inventory parameters for regionalised mixes of municipal waste disposal in ecoinvent v3.5. Doka Life Cycle Assessments, 2018 http://www.doka.ch/publications.htm (accessed Sept 2, 2023).
62 International Solid Waste Association. A Roadmap for closing Waste Dumpsites: The World's most Polluted Places. International Solid Waste Association, 2021 https://www.iswa.org/closing-the-worlds-biggest-dumpsites-task-force/?v=79cba1185463#:~:text=ISWA's Roadmap,-Closing down a&text=The report "A Roadmap for,alternative sound waste management system. (accessed Oct 27, 2025).
63 Wiedinmyer C, Yokelson RJ, Gullett BK. Global emissions of trace gases, particulate matter, and hazardous air pollutants from open burning of domestic wastE. *Environ Sci Technol* 2014; **48**: 9523–30.
64 Velis CA, Cook E. Mismanagement of Plastic Waste through Open Burning with Emphasis on the Global South: A Systematic Review of Risks to Occupational and Public Health. Environ Sci Technol. 2021; **55**: 7186–207.
65 Wang X, Firouzkouhi H, Chow JC, Watson JG, Carter W, De Vos ASM. Characterization of gas and particle emissions from open burning of household solid waste from South Africa. *Atmos Chem Phys* 2023; **23**: 8921–37.
66 Chamas A, Moon H, Zheng J, *et al.* Degradation Rates of Plastics in the Environment. *ACS Sustain Chem Eng* 2020; **8**: 3494–511.
67 Van Sebille E. The oceans' accumulating plastic garbage. *Phys Today* 2015; **68**: 60–1.
68 Ellen MacArthur Foundation. The New Plastics Economy Global Commitment 2019: Progress Report. Cowes, United Kingdom: Ellen Macarthur Foundation, 2019 https://emf.thirdlight.com/link/d81jyzj5q3li-ico7uz/@/preview/1?o (accessed Sept 28, 2023).
69 United Nations Environment Programme (UNEP). Single-use plastics: a roadmap for sustainability. UNEP, 2018 https://www.unep.org/resources/report/single-use-plastics-roadmap-sustainability (accessed Oct 27, 2025).
70 European Parliament, Council of the European Union. Directive (EU) 2019/904 of the European Parliament and of the Council of 5 June 2019 on the reduction of the impact of certain plastic products on the environment. 2019 https://eur-lex.europa.eu/eli/dir/2019/904/oj (accessed Sept 28, 2023).
71 Cepi. Key Statistics 2021: European pulp & paper industry. Cepi, 2022 https://www.cepi.org/wp-content/uploads/2022/07/Key-Statistics-2021-Final.pdf (accessed June 12, 2023).
72 Joint Research Centre (European Commission), Nessi S, Sinkko T, *et al.* Life Cycle Assessment (LCA) of alternative feedstocks for plastics production. Part 1, the Plastics LCA method. Publications Office of the European Union, 2021 https://data.europa.eu/doi/10.2760/271095 (accessed June 12, 2023).
73 Green Source. Home - EU Green Source. Green Source. 2023. https://www.eugreensource.org/ (accessed Oct 3, 2023).

74 European Commission. Environmental impact assessments of innovative bio-based products. Task 1 of 'Study on Support to R&I Policy in the Area of Bio-based Products and Services'. European Union, 2019 https://op.europa.eu/en/publication-detail/-/publication/15bb40e3-3979-11e9-8d04-01aa75ed71a1 (accessed Nov 4, 2025).
75 CE Delft. Verpakkingen onder de SUP-richtlijn. CE Delft, 2020 https://cedelft.eu/publications/packaging-under-the-sup-directive-an-environmental-exploration-of-the-potential-impact-of-measures-for-several-illustrative-example-cases/ (accessed Nov 4, 2025).
76 The Danish Environmental Protection Agency. Life Cycle Assessment of grocery carrier bags. The Danish Environmental Protection Agency, 2018 https://www2.mst.dk/udgiv/publications/2018/02/978-87-93614-73-4.pdf (accessed Oct 20, 2025).
77 National Council for Air and Stream Improvement (NCASI). Review of life cycle assessments comparing paper and plastic products. White Paper (WP-20-09). NCASI, 2020 https://www.ncasi.org/resource/review-of-life-cycle-assessments-comparing-paper-and-plastic-products/ (accessed Nov 4, 2025).
78 Joint Research Centre (European Commission), Nessi S, Sinkko T, *et al.* Life Cycle Assessment (LCA) of alternative feedstocks for plastics production Part 2: illustrative case studies. Publications Office of the European Union, 2022 https://data.europa.eu/doi/10.2760/655230 (accessed June 12, 2023).
79 European Commission. Developer Environmental Footprint (EF). European Commission, 2022 https://eplca.jrc.ec.europa.eu/LCDN/developerEF.xhtml (accessed Sept 13, 2023).
80 Moya, J. A., Pavel CC. Energy efficiency and GHG emissions: Prospective scenarios for the pulp and paper industry. European Union, 2018 https://publications.jrc.ec.europa.eu/repository/handle/JRC111652 (accessed Sept 13, 2023).
81 World Bank. What a Waste Global Database. Country level dataset. World Bank. 2021. https://datacatalog.worldbank.org/search/dataset/0039597 (accessed June 12, 2023).
82 European Commission. EU policy framework on biobased, biodegradable and compostable plastics. European Commission. 2022. https://environment.ec.europa.eu/system/files/2022-12/COM_2022_682_1_EN_ACT_part1_v4.pdf (accessed Nov 6, 2025).
83 European Commission. Biobased, biodegradable and compostable plastics - Environment. European Commission. 2025. https://environment.ec.europa.eu/topics/plastics/biobased-biodegradable-and-compostable-plastics_en (accessed Nov 6, 2025).
84 NatureWorks. NatureWorks Home. NatureWorks. 2023. https://www.natureworksllc.com/ (accessed Sept 22, 2023).
85 Bagheri AR, Laforsch C, Greiner A, Agarwal S. Fate of So-Called Biodegradable Polymers in Seawater and Freshwater. *Global Challenges* 2017; **1**: 1700048.
86 Wichers E, Steffen Peiser . H. Atomic Weight. Encyclopedia Britannica. 2023; published online Oct. https://www.britannica.com/science/atomic-weight (accessed Sept 22, 2023).
87 Greenwood SC, Walker S, Baird HM, *et al.* Many Happy Returns: Combining insights from the environmental and behavioural sciences to understand what is required to make reusable packaging mainstream. *Sustain Prod Consum* 2021; **27**: 1688–702.
88 United Nations Environment Programme (UNEP). Single-use plastic bottles and their alternatives – Recommendations from Life Cycle Assessments. UNEP, 2020

https://www.lifecycleinitiative.org/wp-content/uploads/2020/07/UNEP_PLASTIC-BOTTLES-REPORT_29-JUNE-2020_final-low-res.pdf (accessed Oct 27, 2025).
89 United Nations Environment Programme (UNEP). Single-use plastic take-away food packaging and its alternatives: Recommendations from Life Cycle Assessments. UNEP, 2020 https://www.lifecycleinitiative.org/wp-content/uploads/2020/10/Take-Away-food-containers_REPORT_LR.pdf (accessed Oct 27, 2025).
90 United Nations Environment Programme (UNEP). Single-use plastic tableware and its alternatives – Recommendations from Life Cycle Assessments. UNEP, 2021 https://www.lifecycleinitiative.org/wp-content/uploads/2021/03/UNEP-D001-Tableware-Report_Lowres.pdf (accessed Oct 27, 2025).
91 United Nations Environment Programme (UNEP). Supermarket food packaging and its alternatives: Recommendations from Life Cycle Assessments. UNEP, 2022 https://www.lifecycleinitiative.org/wp-content/uploads/2023/03/UNEP-D010-Food-Packaging-Report.pdf (accessed Oct 20, 2025).
92 United Nations Environment Programme (UNEP). Single-use nappies and their alternatives: Recommendations from Life Cycle Assessments. UNEP, 2021 https://www.lifecycleinitiative.org/wp-content/uploads/2021/03/UNEP-D003-Nappies-Report_lowres.pdf (accessed Oct 27, 2025).
93 Amienyo D, Gujba H, Stichnothe H, Azapagic A. Life cycle environmental impacts of carbonated soft drinks. *Int J Life Cycle Assess* 2013; **18**: 77–92.
94 Postacchini L, Mazzuto G, Paciarotti C, Ciarapica FE. Reuse of honey jars for healthier bees: Developing a sustainable honey jars supply chain through the use of LCA. *J Clean Prod* 2018; **177**: 573–88.
95 Stefanini R, Borghesi G, Ronzano A, Vignali G. Plastic or glass: a new environmental assessment with a marine litter indicator for the comparison of pasteurized milk bottles. *International Journal of Life Cycle Assessment* 2021; **26**: 767–84.
96 Gallego-Schmid A, Mendoza JMF, Azapagic A. Improving the environmental sustainability of reusable food containers in Europe. *Science of The Total Environment* 2018; **628–629**: 979–89.
97 Youngblood K, Brooks A, Das N, *et al.* Rapid Characterization of Macroplastic Input and Leakage in the Ganges River Basin. *Environ Sci Technol* 2022; **56**: 4029–38.
98 Schencking LTF, Stamminger R. What science knows about our daily dishwashing routine. *Tenside, Surfactants, Detergents* 2022; **59**: 205–20.
99 Berkholz P, Kobersky V, Stamminger R. Comparative analysis of global consumer behaviour in the context of different manual dishwashing methods. *Int J Consum Stud* 2013; **37**: 46–58.
100 Gallego-Schmid A, Mendoza JMF, Azapagic A. Environmental impacts of takeaway food containers. *J Clean Prod* 2019; **211**: 417–27.
101 ecoinvent. Electricity. ecoinvent, 2023 https://ecoinvent.org/the-ecoinvent-database/sectors/electricity/ (accessed Sept 18, 2023).
102 Mendoza Beltran A, Cox B, Mutel C, *et al.* When the Background Matters: Using Scenarios from Integrated Assessment Models in Prospective Life Cycle Assessment. *J Ind Ecol* 2020; **24**: 64–79.
103 Ritchie H, Roser M. Electricity Mix. Our World in Data. 2023. https://ourworldindata.org/electricity-mix (accessed Sept 18, 2023).
104 International Energy Agency (IEA). Electricity Market Report – December 2020. IEA Publications, 2020 https://iea.blob.core.windows.net/assets/a695ae98-cec1-43ce-9cab-c37bb0143a05/Electricity_Market_Report_December_2020.pdf (accessed Oct 4, 2023).
